# Photocatalytic synthesis of CF_3_-containing *β*-amino alcohols via covalent metal–organic frameworks

**DOI:** 10.1093/nsr/nwaf463

**Published:** 2025-10-29

**Authors:** Xu Chen, Ri-Qin Xia, Yucong Huang, Ji Zheng, Yu-Mei Wang, Xilin Jia, Yu Han, Zhongxin Chen, Guo-Hong Ning, Dan Li

**Affiliations:** College of Chemistry and Materials Science, Guangdong Provincial Key Laboratory of Supramolecular Coordination Chemistry, Jinan University, Guangzhou 510632, China; College of Chemistry and Materials Science, Guangdong Provincial Key Laboratory of Supramolecular Coordination Chemistry, Jinan University, Guangzhou 510632, China; School of Science and Engineering, The Chinese University of Hong Kong, Shenzhen 518172, China; College of Chemistry and Materials Science, Guangdong Provincial Key Laboratory of Supramolecular Coordination Chemistry, Jinan University, Guangzhou 510632, China; College of Chemistry and Materials Science, Guangdong Provincial Key Laboratory of Supramolecular Coordination Chemistry, Jinan University, Guangzhou 510632, China; Center for Electron Microscopy, School of Emergent Soft Matter, and State Key Laboratory of Pulp and Paper Engineering, South China University of Technology, Guangzhou 510640, China; Center for Electron Microscopy, School of Emergent Soft Matter, and State Key Laboratory of Pulp and Paper Engineering, South China University of Technology, Guangzhou 510640, China; School of Science and Engineering, The Chinese University of Hong Kong, Shenzhen 518172, China; College of Chemistry and Materials Science, Guangdong Provincial Key Laboratory of Supramolecular Coordination Chemistry, Jinan University, Guangzhou 510632, China; College of Chemistry and Materials Science, Guangdong Provincial Key Laboratory of Supramolecular Coordination Chemistry, Jinan University, Guangzhou 510632, China

**Keywords:** covalent metal–organic frameworks, CF_3_-containing β-amino alcohols, photocatalysis, continuous flow

## Abstract

The construction of CF_3_-containing β-amino alcohols represents a promising approach for drug discovery due to their enhanced metabolic stability and tunable pharmacological properties. However, the development of non-noble metal heterogeneous photocatalysts for the efficient one-step synthesis of trifluoromethyl (CF_3_)-containing β-amino alcohol motifs under mild conditions poses significant challenges. In this work, we successfully synthesized two photoactive covalent metal–organic frameworks capable of photocatalysing the one-step conversion of allylamines into CF_3_-containing β-amino alcohols via trifluoromethylation and photooxidation. Notably, the catalytic reaction can be performed under continuous-flow conditions, reducing the reaction time from 12 h to 50 min while achieving a product yield of 6.2 g of CF_3_-containing β-amino alcohol. Additionally, under a CO_2_ atmosphere, CF_3_-containing oxazolidinones can be efficiently obtained through the oxytrifluoromethylation of alkenes. Both catalytic reactions demonstrate high yields under natural sunlight illumination, further highlighting the practicality and sustainability of this approach.

## Teaser

A novel trinuclear copper-based covalent metal—organic framework has been synthesized and demonstrated to function as an efficient photocatalyst for the one-step synthesis of CF3-containing β-amino alcohols under either air or carbon dioxide atmosphere.

## INTRODUCTION

**Figure 1.**
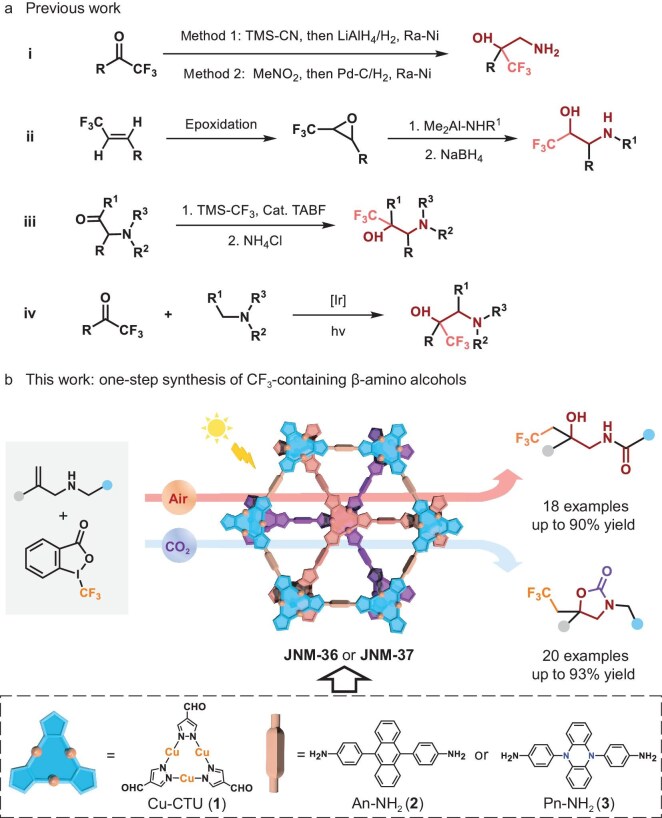
(a) Previous work for synthesis of β-amino alcohols. (b) Photosynthesis of CF_3_-containing β-amino alcohols using **JNM-36** or **JNM-37**, and schematic illustration for synthesis of **JNM-36** or **JNM-37**.

β-Amino alcohols are a prevalent structural motif found in natural products, active pharmaceutical ingredients (APIs) and various chemical entities [1–3]. These compounds are integral to numerous small-molecule drugs, such as formoterol, salmeterol, betanis, ventolin and epinephrine, which are listed among the top-selling medications (Scheme S1) [4]. Furthermore, β-amino alcohols can be incorporated into oxazolidinone rings [5], serving as key pharmacophores in APIs such as rivaroxaban, an anticoagulant, and cytoxazone, an immunomodulator (Scheme S1). Various methods have been employed to synthesize β-amino alcohols, including the reduction of amino acids [6], ring-opening reactions of epoxides or aziridines [7,8], nucleophilic addition of α-hydroxy imine or α-amino carbonyls [9–11], aminohydroxylation of olefins [12,13], radical addition[14] and photocatalysis [15,16]. On the other hand, trifluoromethyl (CF_3_)-substituted organic compounds have wide applications in pharmaceuticals, functional materials and agrochemicals, as the group imparts enhanced lipophilicity, metabolic stability and electronegativity—key properties for optimizing bioactivity and material performance [17,18]. The construction of CF_3_-containing β-amino alcohols is particularly promising for drug discovery, as they offer enhanced metabolic stability and tunable pharmacological properties. However, limited methods have been developed, such as the reduction of cyanohydrins derived from trifluoromethyl ketones [19], the Henry reaction and subsequent reduction of β-nitro alcohols [20], ring opening of trifluoromethyl oxiranes with *N*-nucleophiles [21], nucleophilic trifluoromethylation of α-amino carbonyls [22] and photocatalytic radical–radical cross-coupling from trifluoromethyl ketones and tertiary amines (Fig. 1a) [23]. Despite significant advances, the employment of expensive transition metals (i.e. Pd and Ir) and harsh conditions, low yields, as well as the reusability issues of these homogeneous catalysts have limited their further applications. Moreover, most of the reported approaches required multiple-step synthesis, not only for the starting substrate (i.e. trifluoromethyl ketones and trifluoromethyl oxiranes), but also for the product (i.e. CF_3_-containing β-amino alcohol). Therefore, the development of non-noble-metal, highly efficient heterogeneous photocatalysts for the one-step synthesis of CF_3_-containing β-amino alcohol skeletons under mild conditions is highly desirable, yet remains unexplored.

Metal–organic frameworks (MOFs) possess ordered crystalline architectures, intrinsic high porosity, tunable photophysical and photochemical properties, and abundant active sites. Notably, photocatalytic active sites can be strategically incorporated into MOFs, such as organic linkers (e.g. via photosensitizer integration), metal nodes (e.g. through metal exchange) or pores (e.g. through photoactive metal complexes encapsulation), making them promising heterogeneous photocatalysts for organic transformations, including the tandem transformation of terminal olefins, C–H oxidation and the oxidation of sulfides and photocatalytic coupling reactions such as C–C, C–N, C–O and CF_3_–C coupling [24–33]. Recently, we have developed a novel class of crystalline porous materials, covalent metal–organic frameworks (CMOFs), by integrating trinuclear coinage metal-based cyclic units (M-CTUs, M = Cu, Ag, Au) through dynamic covalent bonding. These CMOFs uniquely combine the metal sites of MOFs with the exceptional stability of covalent organic frameworks [34–38]. Impressively, our studies have demonstrated that these photosensitizing CMOFs can efficiently catalyse the conversion of terminal alkenes and alkynes into primary alcohols via a one-pot tandem reaction. This process synergistically integrates copper-catalysed hydroboration and photocatalysed aerobic oxidation, showcasing the versatility and potential of CMOFs in catalytic applications [39]. Previously, Singaram has reported the preparation of β-amino alcohols via the hydroboration and oxidation of enamines [40]. Inspired by these findings, we envisioned that the simultaneous trifluoromethylation and oxidation of allylamine by using CMOFs as photocatalysts would enable the one-step synthesis of CF_3_-containing β-amino alcohols (Fig. 1b).

Herein, we synthesized two photoactive Cu-CTUs based CMOFs, denoted as **JNM-36** and **JNM-37** (**JNM** = Jinan materials), through the Schiff-base condensation reaction. Interestingly, these CMOFs can photocatalyse allylamines to give CF_3_-containing β-amino alcohols via the simultaneous trifluoromethylation and oxidation of alkenes (e.g. yields of ≤90% with 18 examples) under ambient conditions (Fig. 1b). Impressively, this catalytic reaction can be performed under continuous-flow conditions with a reaction time shortened to 50 min and 6.2 g of CF_3_-containing β-amino alcohol is synthesized, demonstrating the practicality and scale-up potential of our method. Moreover, under a CO_2_ atmosphere, CF_3_-containing oxazolidinones as β-amino alcohol derivatives are readily obtained through the difunctionalization of alkenes utilizing the **JNM**s as photocatalysts (e.g. yields of ≤93% with 20 examples). Interestingly, these two catalytic reactions can be conducted under natural sunlight irradiation with good yields. Our work paves a new way for the rational design of noble-metal-free MOFs as heterogeneous photocatalysts for the synthesis of CF_3_-containing β-amino alcohols and oxazolidinones.

## RESULTS AND DISCUSSION

### Synthesis and characterization

Cu-CTC (**1**) has been prepared according to our reported procedures (Fig. S1) [41]. A mixture of 1-butanol (*n*-BuOH), 1,2-dichlorobenzene (*o*-DCB) and 6 M aqueous trifluoroacetic acid containing **1** and organic linker 4,4′-(anthracene-9,10-diyl)dianiline (**2**) or 4,4′-(phenazine-5,10-diyl)dianiline (**3**) through the imine condensation reaction would provide highly crystalline powders of **JNM-36** or **JNM-37** (Fig. 1b, Figs S2 and S3). The chemical structures of the **JNM**s were confirmed through Fourier transform infrared (FT-IR) spectroscopy and solid-state ^13^C cross polarization/magic angle spinning nuclear magnetic resonance (^13^C CP/MAS NMR) analyses. As shown in Figs S4 and S5, the formation of imine bonds was confirmed by the appearance of the C=N stretching band at ∼1622 and ∼1629 cm^−1^ for **JNM-36** and **JNM-37**, respectively. The solid-state ^13^C CP-MAS NMR spectra of **JNM-36** and **JNM-37** showed typical resonance peaks of imine carbons at ∼150 ppm—further evidence of the existence of imine linkages (Fig. S6). The oxidation states of Cu in the **JNM**s were investigated by utilizing X-ray photoelectron spectroscopy (XPS) analysis. Specifically, characteristic peaks attributed to Cu(I) 2p and Cu(II) 2p were observed, indicating the presence of both Cu^+^ and Cu^2+^ ions in the **JNM**s (Fig. S7). Scanning electron microscopy images of **JNM-36** and **JNM-37** revealed that they both featured a 2D flake morphology (Fig. S8). Energy-dispersive X-ray spectroscopy elemental mappings conducted on the **JNM**s demonstrated the uniform distribution of Cu, C and N elements within the structure (Figs S9 and S10).

**Figure 2.**
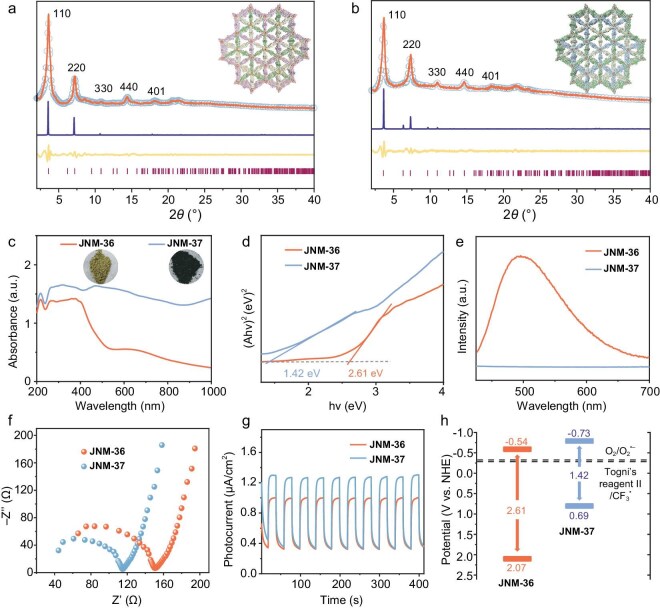
PXRD structural analysis of (a) **JNM-36** and (b) **JNM-37** (experimental curve, orange line; Pawley refinement data, pale-blue hollow circle; simulated profile of ABC packing mode, blue line; difference plot, faint yellow line; Bragg peak positions, brownish line). Inset: refined ABC-stacking model of **JNM-36** and **JNM-37**. (c) Solid-state UV–vis diffuse reflectance spectroscopy of **JNM-36** and **JNM-37**. Inset: photographs of powder samples of **JNM-36** and **JNM-37**. (d) Tauc plot curves of **JNM-36** and **JNM-37**. (e) PL spectra, (f) Nyquist plots of electrochemical impedance spectroscopy, (g) photocurrent response curves and (h) energy-level diagrams of **JNM-36** and **JNM-37**.

The crystal structures of **JNM-36** and **JNM-37** were confirmed by using powder X-ray diffraction (PXRD) analyses combined with structural simulations (Fig. 2a and b). The observed PXRD patterns for **JNM-36** showed two intense peaks at 3.60° and 7.26°, accompanied by three relatively weak peaks at 10.82°, 14.35° and 18.20°, which corresponded to the (110), (220), (330), (440) and (401) reflections, respectively (Fig. 2a, orange line). Similarly, the observed PXRD patterns for **JNM-37** (Fig. 2b, orange line) exhibited five peaks at 3.68°, 7.37°, 10.99°, 14.64° and 18.41°, respectively. In addition, structural modeling and geometry optimization were performed through Materials Studio 2019 (see Supplementary information for details). Comparison of the experimental curves and the calculated PXRD patterns, including eclipsed stacking (AA) as well as staggered stacking (AB and ABC) models, revealed that the stacking modes of **JNM-36** and **JNM-37** were both ABC-stacking models (Figs S11–S14 and Tables S1 and S2). Furthermore, Pawley refinement in the *R*–3 space group with unit cell parameters of *a* = *b* = 49.15, *c* = 5.45 Å (**JNM-36**) and *a* = *b* = 48.26, *c* = 5.49 Å (**JNM-37**) reproduced an experimental curve with good residual factors (*R*_p_ = 2.26% and *R*_wp_ = 3.28% for **JNM-36**; *R*_p_ = 1.68% and *R*_wp_ = 2.63% for **JNM-37**) (Figs S11–S14 and Tables S1 and S2).

The porosity of the **JNM**s was evaluated by taking nitrogen adsorption–desorption measurements at 77 K. The Brunauer–Emmett–Teller surface areas of **JNM-36** and **JNM-37** were determined to be 149.6 and 108.9 m^2^ g^−1^, respectively (Figs S15 and S16). The pore-size distribution of **JNM-36** and **JNM-37** was centered at ∼0.9 nm when nonlocal density functional theory (DFT) was used, in good agreement with the theoretical value of 0.8 nm from the staggered ABC-stacking models (Figs S15 and S16). The high-resolution transmission electron microscopy (HR-TEM) images show lattice fringe spacings of 2.34 and 2.30 nm for **JNM-36** and **JNM-37**, respectively. These values correspond well with the *d*-spacing of the (110) reflection predicted for **JNM** models with ABC stacking (2.41 and 2.40 nm for **JNM-36** and **JNM-37**, respectively) (Figs S17 and S18). Thermogravimetric analysis results indicated that the **JNM**s exhibited commendable thermal stability, with no obvious weight loss when heated to 200°C under a nitrogen atmosphere (Fig. S19). In addition, after 3-day immersion in various solvents (e.g. *N,N*-dimethylformamide (DMF), acetone and other common solvents), water and base (i.e. aqueous NaOH (15 M)) under ambient conditions, **JNM-36** and **JNM-37** maintained structural integrity, as evidenced by the PXRD patterns being identical to those of the pristine samples, highlighting good chemical stability with pronounced resistance under basic conditions (Fig. S20). However, they were found to be unstable under acidic conditions, such as in 0.1 M HCl (Fig. S20).

### Photophysical properties

The photophysical properties of the **JNM**s were evaluated to determine their appropriateness for photocatalysis. As shown in Fig. 2c, the solid-state diffuse reflectance ultraviolet (UV)–visible spectroscopy of **JNM-37** exhibited a strong absorption band ranging from 200 to 800 nm, whereas the absorption edge of **JNM-36** was located at 509 nm. The optical band gaps (*E*_g_) of **JNM-36** and **JNM-37** were estimated to be 2.61 and 1.42 eV, respectively, from the Tauc plots obtained by employing the Kubelka–Munk function (Fig. 2d). These results revealed that the incorporation of dihydrophenazine motifs significantly enhanced the visible-light absorption capacity and narrowed the *E*_g_ of **JNM-37**, leading to the boosting of photocatalytic performance [42]. Based on the Mott–Schottky experiments, the average flat-band potentials of **JNM-36** and **JNM-37** were determined to be −0.54 and −0.73 V vs. normal hydrogen electrode (NHE), respectively, which are also equivalent to their conduction-band (CB) potentials (Fig. S21). Subsequently, the valence-band potentials of **JNM-36** and **JNM-37** were calculated to be 2.07 and 0.73 V vs. NHE, respectively (Fig. 2h). Notably, the CB values of the **JNM**s were both more negative than the reduction potential of Togni reagent II (i.e. −0.32 V vs NHE) [43], indicating that the reduction of Togni reagent II to give the •CF_3_ radical by the **JNM**s under light irradiation was thermodynamically favorable (Fig. 2h). The photoelectrochemical properties of the **JNM**s were further assessed by using photoluminescence (PL) spectra, transient photocurrent and electrochemical impedance spectroscopy analyses (Fig. 2e–g). As shown in Fig. 2e, **JNM-37** was non-emissive, while **JNM-36** exhibited a strong emission peak located at ∼495 nm, suggesting a superior separation efficiency of photoinduced electron–hole pairs and faster charge-carrier dynamics of **JNM-37** compared with **JNM-36** [44]. Moreover, **JNM-37** exhibited the lowest charge-transfer (CT) resistance and the highest photocurrent intensity (Fig. 2f and g), further demonstrating its superior interfacial charge-migration capability and photogenerated charge-carrier separation efficiency.

### Catalytic investigation

#### Hydroxy trifluoromethylation of allylamines

The visible-light-catalysed hydroxy trifluoromethylation of allylamines under air was initially investigated by using the **JNM**s as the heterogeneous photocatalysts. Firstly, the hydroxy trifluoromethylation of **4a** was used as a model reaction for optimizing the reaction conditions. As shown in Table 1 (entry 1), the CF_3_-containing β-amino alcohol product **5a** could be obtained in an isolated yield of 85% when the reaction was carried out in the presence of 1 mol% of **JNM-37** as the photocatalyst, 1,8-diazabicyclo[5.4.0]undec-7-ene (DBU) as the base, DMF as the solvent, Togni reagent II and a 30-W white light-emitting diode (LED) as the light source, under air. In the absence of **JNM-37**, DBU, light source or air, the reaction was difficult to proceed (Table 1, entries 2–6). Replacing the DBU with K_2_CO_3_, triethylenediamine (DABCO) or 7-methyl-1,5,7-triazabicyclo[4.4.0]dec-5-ene (MTBD) reduced the yields to 56%, 77% or 46%, respectively (Table 1, entries 7–9). Using different solvents in place of the DMF will notably decrease the reaction yield (Table 1, entries 10–13). Notably, the substitution of the catalyst from **JNM-37** to **JNM-36** remarkably reduced the yield to 47%, indicating a lower photocatalytic efficiency of **JNM-36** compared with **JNM-37** (Table 1, entry 14). Such results are consistent with the above-mentioned superior photophysical properties of **JNM-37**. When Cu-CTC **1, 3**, Cu_2_O or Cu(NO_3_)_2_ was employed as a catalyst instead of **JNM-37**, this resulted in significantly reduced yields (Table 1, entries 15–18). Notably, **5a** can be reduced to the amine (**5a-re**) by using NaBH_4_ as a reducing agent (Fig. S22). The catalytic kinetics of the **JNM**s were evaluated by graphing the NMR yield of **5a** versus reaction time. As shown in Fig. S23a, the product was detected after 1 h in a yield of 16% by using **JNM-37** as the photocatalyst, which further elevated to 87% at 12 h. However, **JNM-36** exhibited a slower catalytic rate and lower yield for the hydroxy trifluoromethylation of **4a** under air. The determination of the rate constants led to *k***_JNM-37_**/*k***_JNM-36_** = 2.7 (Fig. S23b).

**Table 1.** Condition optimization of **JNM-37**-photocatalytic hydroxy trifluoromethylation of allylamines.^a^

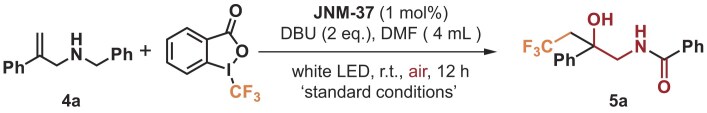		
Entry	Change from the ‘standard conditions’	Yield (%)^b^
1	None	87 (81)^c^
2	No **JNM-37**	15
3	No light	18
4	No DBU	<1
5	N_2_ instead of air	<1
6	O_2_ instead of air	92
7	K_2_CO_3_ instead of DBU	56
8	DABCO instead of DBU	77
9	MTBD instead of DBU	46
10	CH_3_CN instead of DMF	53
11	DMF:H_2_O (19:1) instead of DMF	73
12	DMF:H_2_O (1:1) instead of DMF	12
13	CH_3_OH instead of DMF	47
14	**JNM-36** instead of **JNM-37**	47
15	**1** instead of **JNM-37**	42
16	**3** instead of **JNM-37**	31
17	Cu_2_O instead of **JNM-37**	56
18	Cu(NO_3_)_2_ instead of **JNM-37**	45

a Reaction condition: **4a** (0.4 mmol), DBU (0.8 mmol), solvent (4 mL), white LED, room temperature. ^b^NMR yield with ^19^F NMR analysis using 1,4-difluorobenzene as internal standard. ^c^The yield was determined by using the isolated product. DABCO, triethylenediamine; MTBD, 7-methyl-1,5,7-triazabicyclo[4.4.0]dec-5-ene.

#### Oxytrifluoromethylation of allylamines with CO_2_

Given the prevalence of amino alcohol moieties in oxazolidinones, the visible-light-catalysed oxytrifluoromethylation of allylamines with CO_2_ was also tested by using the **JNM**s as heterogeneous photocatalysts. The oxytrifluoromethylation of **4a** with CO_2_ was used as the model reaction for optimizing the reaction conditions (Table S3). Interestingly, the employment of 1 mol% of **JNM-37** as the photocatalyst combined with a 30-W white light LED as the illumination source enabled the reaction to proceed under ambient CO_2_ pressure, resulting in the generation of the desired CF_3_-containing 2-oxazolidone product **6a** in a good isolated yield of 81% (Table S3, entry 1). In the absence of **JNM-37**, DBU, light source or CO_2_, the reaction was difficult to proceed (Table S3, entries 2–5). Using alternative bases instead of DBU or different solvents in place of DMF will notably decrease the reaction yield (Table S3, entries 6–11). Replacing **JNM-37** with **JNM-36, 1, 3**, Cu_2_O or Cu(NO_3_)_2_ led to reduced yields, consistently with the oxytrifluoromethylation of allylamines with air above (Table S3, entries 12–16). The catalytic kinetics of the **JNM**s were assessed through plotting the NMR yield of **6a** versus reaction time. As shown in Fig. S34a, **JNM-37** exhibited a faster catalytic rate and higher yield for the oxytrifluoromethylation of **4a** with CO_2_ and the determination of the rate constants led to *k***_JNM__-Y_**/*k***_JNM__-X_** = 2.5 (Fig. S34b).

### Reusability test

The reusability of **JNM-37** was investigated. The yields were nearly constant after five catalytic cycles for the hydroxy trifluoromethylation of the allylamines (Fig. S24a), whereas, for the oxytrifluoromethylation of the allylamines with CO_2_, the yields decreased slightly from 86% to 80% after five catalytic cycles (Fig. S35a). The recovered **JNM-37** still maintained its structural integrity after the catalytic cycles for these two reactions, confirmed by using PXRD patterns and XPS measurements (Figs S24b, S25, S35b and S36), suggesting that **JNM-37** is a promising heterogeneous photocatalyst with good recyclability. An inductively coupled plasma atomic emission spectroscopy (ICP-AES) analysis revealed that only 1.1 and 0.9 wt% of Cu were leached from **JNM-37** after the reactions under air and under a CO_2_ atmosphere, respectively.

### Substrate scope

**Figure 3.**
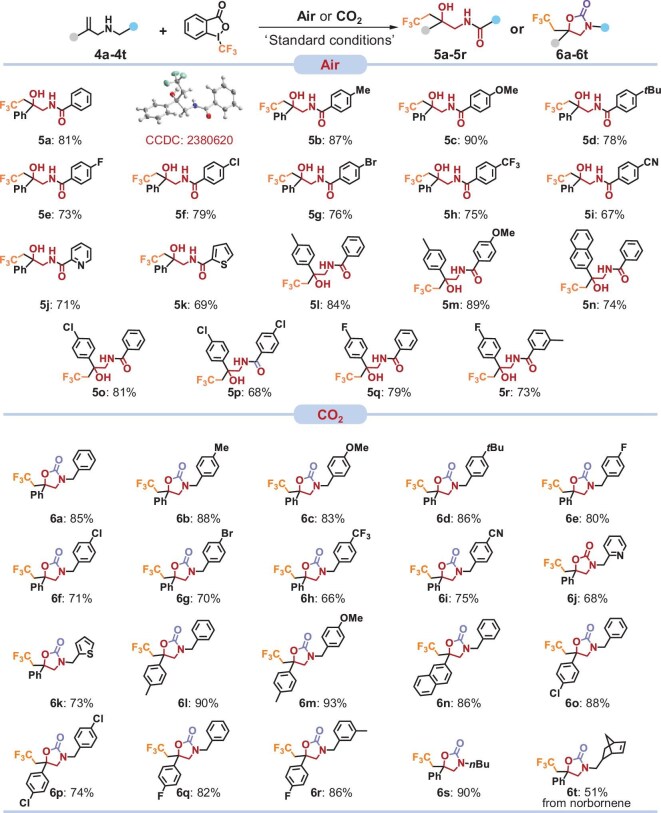
Substrate scope of hydroxy trifluoromethylation or oxytrifluoromethylation of allylamine with air or CO_2_. The optimal reaction conditions are based on Table 1 and Table S3. All yields are determined from isolated products.

Under the optimal reaction conditions, we next sought to explore the substrate scope of allylamines with different substituents. As shown in Fig. 3, electron-donating (**4a**–**4d**), electron-withdrawing (**4e**–**4i**) and heterocycle (**4j** and **4k**) substituents at the amine nitrogen were well tolerated. The corresponding CF_3_-containing β-amino alcohols were obtained in high isolated yields of 67%–90%. The vinyl aromatic groups with different substituents also afforded the desired CF_3_-containing β-amino alcohols (**5l**–**5r**) in moderate to good yields. In addition, we explored the substrate scope of the oxytrifluoromethylation of allylamines with CO_2_. The *N*-benzyl groups with both electron-donating and electron-withdrawing substituents at the para-position were well tolerated, providing the corresponding oxazolidinones in yields of ≤88% (**6a**–**6d, 6e**–**6i**). The allylamines with heterocycle substitutes or alkyl groups were also obtained in high isolated yields ranging from 68% to 90% (**6j, 6k** and **6s**). Moreover, the biologically active molecule derived from norbornene also formed the desired product **6t** in 51% yield. Interestingly, when the vinyl aromatic substituent was attached to an electron-donating group, the corresponding product (**6l**–**6o**) was formed in higher yields (≤93%) compared with its variants with an electron-withdrawing group (**6q** and **6r**), which revealed that the electron-donating effect may promote the reaction. Overall, a wide range of functional groups are compatible with the catalytic reaction conditions, regardless of their electronic nature and substitution patterns, demonstrating that **JNM-37** is a robust photocatalyst for the synthesis of CF_3_-containing β-amino alcohols.

### Mechanistic investigations

**Figure 4.**
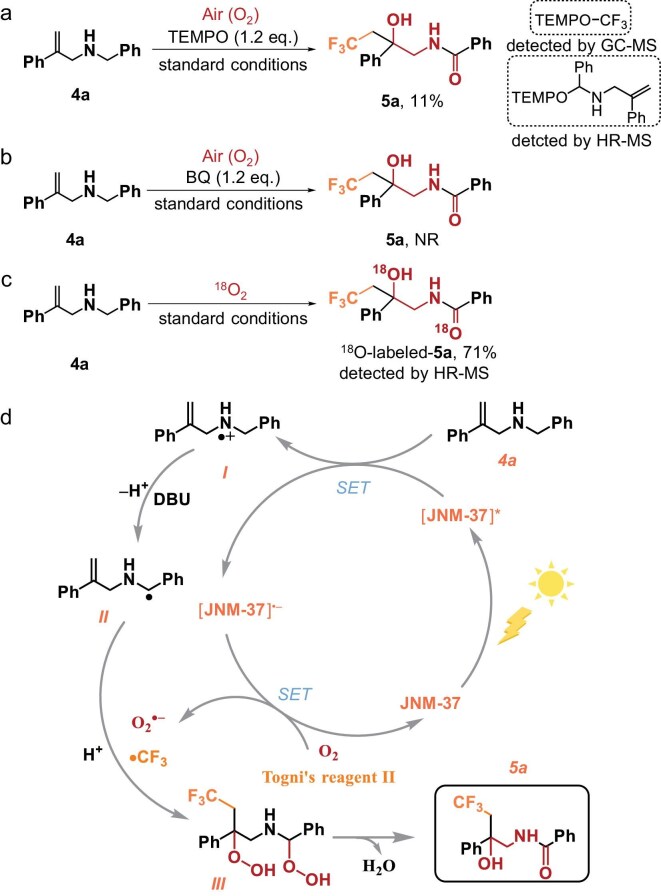
(a and b) Radical trapping experiments for hydroxy trifluoromethylation reaction. (c) ^18^O-isotope-labeling experiments. (d) Possible mechanism of hydroxy trifluoromethylation reaction. BQ: 1,4-benzoquinone; HR-MS: high-resolution mass spectrum.

To gather insights into this hydroxy trifluoromethylation reaction, radical scavengers, such as 2,2,6,6-tetramethyl-1-piperinedinyloxy (TEMPO) or butylated hydroxytoluene (BHT), were employed in the reaction (Fig. 4a and Figs S26a and S27). The reaction was difficult to proceed and free-radical coupling adducts TEMPO-CF_3_, TEMPO-**4a** and BHT-CF_3_ were detected, indicating that the reaction may have proceeded via the radical pathway. Subsequently, the addition of a ^1^O_2_ quencher such as sodium azide (NaN_3_) or an O_2_^•−^ quencher such as *p*-benzoquinone markedly reduced the yield of the amide product (Fig. 4b and Fig. S26b). The generation of O_2_^•−^ and ^1^O_2_ under visible-light irradiation was proved by using electron paramagnetic resonance (EPR) experiments. As shown in Fig. S33, in the presence of an O_2_^•−^ trapping agent (5,5-dimethyl-1-pyrroline *N*-oxide, (DMPO)), the absorption signals of the DMPO–O_2_^•−^ adduct were observed for the **JNM**s under visible-light irradiation. Meanwhile, the production of ^1^O_2_ was validated through the analysis of EPR spectra by utilizing an ^1^O_2_ scavenger (2,2,6,6-tetramethylpiperidine). These results suggest that O_2_^•−^ and ^1^O_2_ play a crucial role in the hydroxy trifluoromethylation process. Moreover, ^18^O-labeling studies were carried out and the ^18^O-labeled product was observed under an ^18^O_2_ atmosphere, indicating that the O sources for the CF_3_-containing β-amino alcohol product had originated from oxygen molecules (Fig. 4c and Figs S26c and S28). Based on these control experiments and the previously reported work [45], a plausible reaction mechanism was proposed, as shown in Fig. 4d. Initially, the photocatalyst **JNM-37** was excited by visible-light irradiation and engaged in a single-electron transfer (SET) process with *N*-benzyl-2-phenylprop-2-en-1-amine (**4a**). The primary function of the base within our system entailed the deprotonation of radical intermediate I to generate radical species II. The photocatalyst radical anion can be oxidized by O_2_ and Tognis reagent II to generate O_2_^•−^ and •CF_3_. Subsequently, the radical species V reacted with the O_2_^•−^ and •CF_3_ to generate intermediate III. Finally, intermediate III transformed into product **5a** with the formation of water.

Similarly, we also investigated the mechanism of the oxytrifluoromethylation of allylamine with CO_2_. The radical capture experiment was explored, indicating that •CF_3_ radical was involved during the catalytic reaction (Fig. S39a and b). Furthermore, isotope-labeling studies were carried out employing ^13^CO_2_, followed by analysis using gas chromatography-mass spectrometry (GC-MS) and ^13^C NMR spectroscopy to elucidate the formation of the ester motif in the oxazolidinones originating from CO_2_ (Figs S37, S38 and S39c). Based on the above experimental results, a possible reaction mechanism was proposed, as shown in Fig. S39d. Upon white-LED light irradiation, the photoexcited **JNM-37** may engage in a process of SET with Tognis reagent II, resulting in the generation of a •CF_3_ radical. Afterward, the addition of the •CF_3_ radical to carbamate anion (IV), which was generated from **4a** and CO_2_, gave the benzylic radical (V). Finally, oxidation of II by excited [**JNM-37]^•+^** species was used to generate a stable carbocation (VI) and regenerate **JNM-37**. The desired product **6a** was formed after the intramolecular cyclization of VI.

DFT calculations were carried out to explore the electronic structures of the **JNM**s (see Supplementary information for details) by utilizing **JNM** fragments as model molecules and further clarifying the difference between their photocatalytic performances. Electrostatic potential surface maps of **JNM-36** and **JNM-37** revealed that the dihydrophenazine unit has a stronger electron-donating ability compared with the anthracene unit (Fig. S41). In addition, a molecular orbital analysis of the **JNM**s was carried out (Fig. S42) and the highest occupied molecular orbital (HOMO) and lowest unoccupied molecular orbital (LUMO) of **JNM-36** displayed a high degree of overlap, in which both were primarily distributed on the anthracene unit. However, the HOMO of **JNM-37** was mainly located in the dihydrophenazine units and the LUMO was primarily located at the Schiff-base bond and pyrazol unit of the CTUs. These results reveal that **JNM-37** shows better charge-separation ability compared with **JNM-36** (Fig. S42), which is consistent with the photocatalysis experiment and electrochemical measurement results. Moreover, to further discuss the charge-separation properties of the excited state, time-dependent DFT calculations for the **JNM**s were performed to investigate the possible electronic excitation transition and the CT mode (Fig. S43). The first single excited state (S_1_) was chosen due to its relatively strong light-absorbing capacity and the electron density difference map based on S_1_ was depicted to visualize the excited-state assignment. Typically, the positive and negative centroids of **JNM-37** were primarily localized on the dihydrophenazine segment and the pyrazole-Schiff-base region, respectively, confirming the formation of donor and acceptor structures within the frameworks, in agreement with molecular orbital analysis. Furthermore, the CT mode would reflect the efficient separation of electrons and holes, which largely affects the photocatalytic performance. Based on the hole–electron theory, the CT efficiency can be identified by using the *S*/*D* value, where *S* represents the overlap degree of the electron and the hole, and *D* represents the centroid distance between the electron and the hole [46]. Usually, a smaller *S*/*D* value suggests a higher CT efficiency. For the **JNM**s, the *S*/*D* value of **JNM-37** (0.27) was much smaller than that of **JNM-36** (54.31), demonstrating that **JNM-37** is more beneficial for CT. The above calculation results suggest that the charge separation and transfer efficiency are remarkably improved by introducing the dihydrophenazine units into the frameworks (Table S4), leading to the enhancement of the photocatalytic performance of **JNM-37** compared with that of **JNM-36** bearing anthracene units.

### Upscaling in a continuous-flow reactor

**Figure 5.**
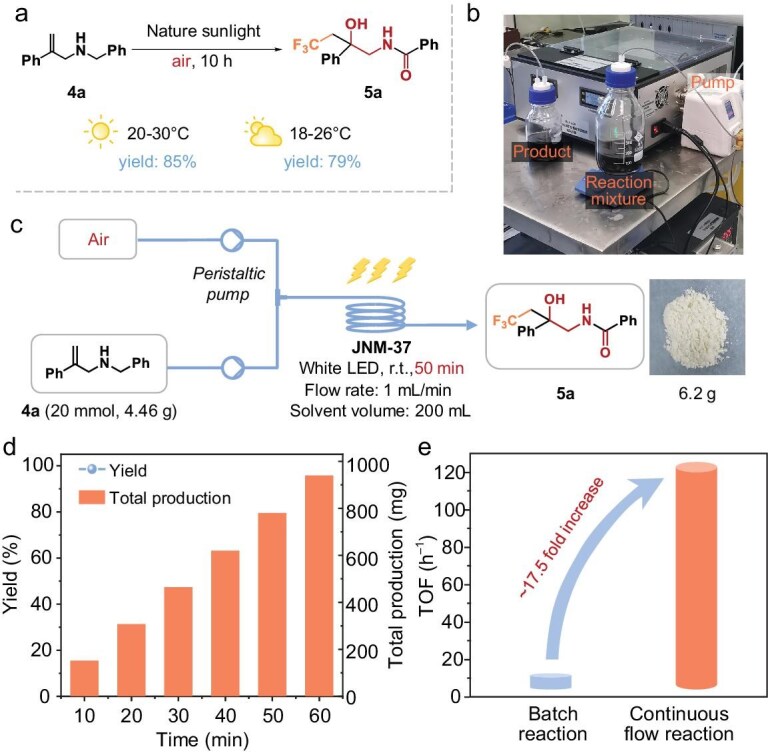
(a) Photosynthesis of CF_3_-containing β-amino alcohols using **JNM-37** under natural sunlight: under different weather conditions. (b) Continuous-flow photoreactor device diagram. (c) Reaction scale-up with a continuous-flow photoreactor. (d) Durability and production rate for photosynthesis of **5a**. (e) Comparison of TOF for hydroxy trifluoromethylation of **4a** in batch and continuous-flow systems.

To demonstrate the practicality of our procedure utilizing **JNM-37** as a photocatalyst, catalytic experiments under natural sunlight irradiation and continuous-flow experiments were conducted (Fig. 5 and Figs S29–S32 and S38). As shown in Fig. 5a, the yields of the hydroxy trifluoromethylation of **4a** can reach >85% under clear weather conditions, which is comparable to white LED. Even under cloudy weather conditions, the reactions can achieve high yields of 79% (Fig. 5a). To reinforce its practicality, a gram-scale photocatalytic reaction (20 mmol) was performed by using a continuous-flow photoreactor (Fig. 5b–e and Figs S30–S32). Remarkably, with the use of **JNM-37** as the photocatalyst, the flow rate of 1 mL/min, and the residence time of 50 min (batch time: 12 h), 6.2 g of the target product was obtained in a yield of 95% (Fig. 5c). We subsequently investigated the durability of this continuous-flow synthesis of CF_3_-containing β-amino alcohols from allylamines. The yield of the continuous-flow reaction was consistently monitored; it exhibited a steady level of ∼95% throughout the run of 1 h and the cumulative production reached 940 mg/h (Fig. 5d). Notably, the continuous-flow system remarkably improved the catalytic efficiency, with a turnover frequency (TOF) of 118 h^−1^, which is >17.5 times higher than that in the batch system (Fig. 5e). The high efficiency of the continuous-flow reaction not only confirms the practicality of our material, but also highlights its potential for future industrial applications.

## CONCLUSION

In summary, we successfully synthesized two photoactive CMOFs, designated as **JNM-36** and **JNM-37**, via the Schiff-base condensation reaction. These **JNM**s serve as efficient photocatalysts for the one-step conversion of allylamines into CF_3_-containing β-amino alcohols. Remarkably, the catalytic reaction can be readily conducted under continuous-flow conditions, significantly reducing the reaction time from 12 h (batch reaction) to 50 min, while yielding 6.2 g of CF_3_-containing β-amino alcohol. Furthermore, under a CO_2_ atmosphere, CF_3_-containing oxazolidinones, as derivatives of β-amino alcohols, can be synthesized through the oxytrifluoromethylation of allylamines using the **JNM**s as photocatalysts. Notably, both catalytic reactions can proceed with good yields under natural sunlight irradiation. The reaction mechanisms were thoroughly investigated by using radical trapping experiments, isotope-labeling experiments and DFT calculations. This work establishes a promising blueprint for the development of noble-metal-free MOF-based heterogeneous photocatalysts, enabling the efficient synthesis of CF_3_-containing β-amino alcohols and oxazolidinones. This strategy has the potential to significantly expand the application of heterogeneous catalysis in various fields.

## Supplementary Material

nwaf463_Supplemental_File
