# Supplementary material for: Photocatalytic synthesis of CF3-containing β-amino alcohols via covalent metal–organic frameworks
Source: Natl Sci Rev. 2025 Oct 29;13(2):nwaf463. doi: 10.1093/nsr/nwaf463 (PMC12839521; doi:10.1093/nsr/nwaf463)
Supplement: nwaf463_Supplemental_File [file nwaf463_supplemental_file.pdf]

Supplementary Information

Photocatalytic synthesis of CF_3-containing β-amino alcohols via covalent metal-organic frameworks

Xu Chen[1], Ri-Qin Xia[1], Yucong Huang[2], Ji Zheng[1], Yu-Mei Wang[1], Xilin Jia[3], Yu Han[3], Zhongxin Chen[2], Guo-Hong Ning[1,*], and Dan Li[1,*]

[1]College of Chemistry and Materials Science, Guangdong Provincial Key Laboratory of Functional Supramolecular Coordination Materials and Applications, Jinan University, Guangzhou 510632, China.

[2]China School of Science and Engineering, The Chinese University of Hong Kong, Shenzhen, Guangdong 518172, China.

[3]Center for Electron Microscopy, South China University of Technology, Guangzhou 510640, China; School of Emergent Soft Matter, South China University of Technology, Guangzhou 510640, China; State Key Laboratory of Pulp and Paper Engineering, South China University of Technology, Guangzhou 510640, China.

Corresponding Author

*E-mail: guohongning@jnu.edu.cn, danli@jnu.edu.cn

Contents

1. Characterizations
2. Representative biologically active β-amino alcohols
3. Synthesis
    3.1 Synthesis of CTC
    3.2 Synthesis of PN-NH_2
    3.3 Synthesis of **JNMs**
4. Fourier-transform infrared (FT-IR) spectra
5. Solid-state ^{13}C CP/MAS NMR spectra of **JNMs**
6. X-ray photoelectron spectroscopy (XPS)
7. Scanning electron microscopy (SEM)
8. Energy Dispersive X-ray Spectroscopy (EDS)
9. Structural simulation
10. Nitrogen adsorption-desorption measurements
11. Transmission electron microscopy (TEM)
12. Thermogravimetric analysis (TGA)
13. Stability in various solvents
14. Mott-Schottky plots
15. Photocatalytic Applications
  15.1 Air system
    15.1.1 Synthesis of substrates
    15.1.2 General procedure for hydroxytrifluoromethylation of allylamines
    15.1.3 The catalytic kinetics
    15.1.4 General procedure for the catalytic cycle test
    15.1.5 Mechanistic Studies
    15.1.6 Catalytic experiment under natural sunlight
    15.1.7 Continuous flow experiments
    15.1.8 EPR experiments
  15.2 CO_2 system
    15.2.1 Optimization of reaction conditions
    15.2.2 The catalytic kinetics
    15.2.3 General procedure for the catalytic cycle test
    15.2.4 isotope labeling experiments
    15.2.5 Mechanistic Studies
    15.2.6 Catalytic experiment under natural sunlight
16. Computational Results
17. ^{1}H and ^{13}C NMR spectra for products

1. Characterizations

All reagents and solvents were purchased from commercial sources and used as received without further purification. Powder X-ray diffraction (PXRD) data were collected on Rigaku Ultima IV diffractometer (40 kV, 40 mA, Cu Kα, λ = 1.5418 Å) from 1.5 ° to 30 ° with a step of 0.02 ° at a scan speed of 1 ° min^{-1}. Thermogravimetric analysis was performed on a Mettler-Toledo (TGA/DSC1) thermal analyzer. Measurement was made on approximately 5 mg of dried samples under a N_2 flow with a heating rate of 10 ℃/min. The morphology characterizations were taken on TEM (JEM-F200) and SEM (COXEM/EM 30AX PLUS). Energy dispersive X-ray spectroscopy (EDS) analyses were performed on COXEM/EM 30AX PLUS. Inductively coupled plasma atomic emission spectroscopy (ICP-AES) tests were performed on ThermoiCAP 7000 SERIES. Fourier-transform infrared (FTIR) was recorded on Nicolet Avatar 360. X-ray photoelectron spectroscopy (XPS) experiments were performed by a Thermo ESCALAB 250XI system. ^{1}H NMR (400 MHz) spectra were recorded on a Bruker AVANCE III HD 400 spectrometer using $CDCl_3$ (1H, δ = 7.26) as deuterated solvent. ^{13}C NMR (100 MHz) spectra on a Bruker Avance 400 spectrometer using $CDCl_3$ (δ = 77) as deuterated solvent. ^{19}F NMR spectra (376 M) were recorded on a Bruker Avance 400 spectrometer. The following abbreviations were used to explain the multiplicities: s = singlet, d = doublet, t = triplet, q = quartet, dd = doublet of doublet, ddd = doublet of doublet of doublet, dt = doublet of triplet, m = multiplet, s br = single broad. Flash column chromatography was performed using Macklin silica gel 60 with solvents. The solid-state ^{13}C CP/MAS NMR spectra were recorded on Bruker AVANCE III 600M. Gas sorption analyses were conducted using Quantachrome Instruments Autosorb-iQ-MP-MP(2 Stat.) (Boynton Beach, Florida USA) with extrahigh pure gases.

2. Representative biologically active β-amino alcohols

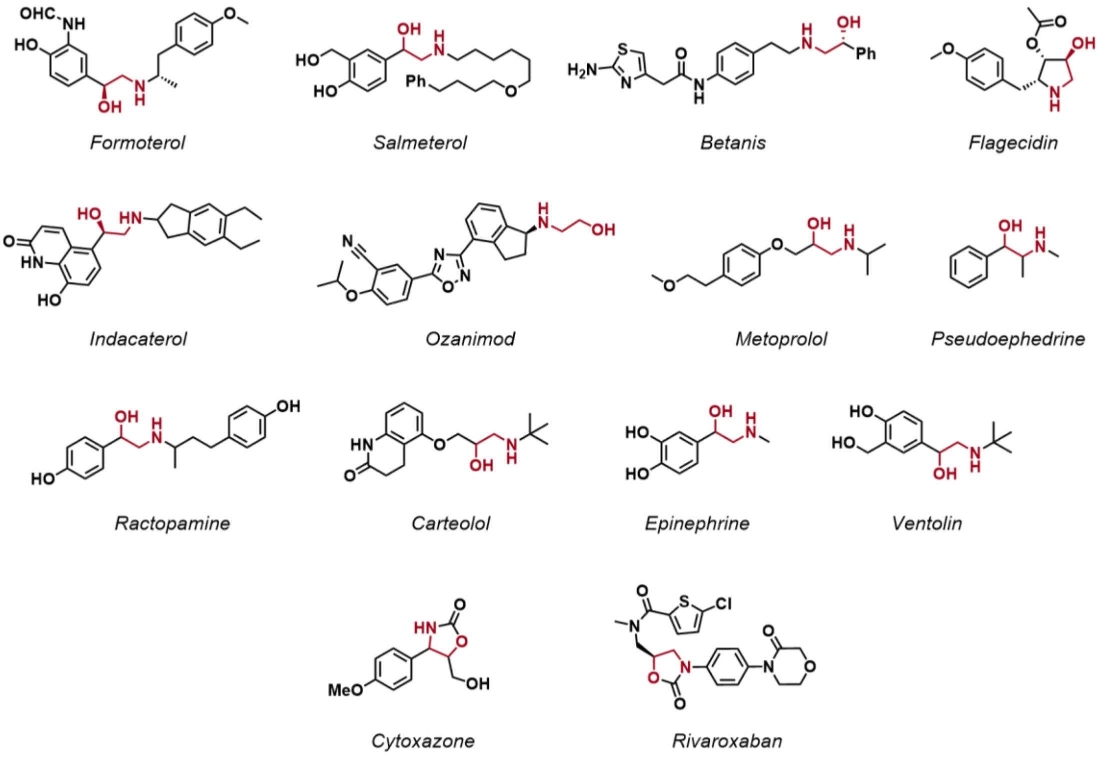

Scheme S1. Representative biologically active β-amino alcohols among the top-selling drugs in 2024.

3. Synthesis

3.1 Synthesis of CTC

The Cu-CTC (**1**) was synthesized according to previously reported procedures.[1] $Cu(NO_3)_2 \cdot 3H_2O$ (20 mg, 0.08 mmol) and 1*H*-pyrazole-4-carbaldehyde (HPyCA, 9.6 mg, 0.1 mmol) were dissolved in a solution comprising 0.5 mL of H_2O, 0.7 mL of DMF, and 0.7 mL of C_2H_5OH. The resulting mixture was placed in an 8 mL Pyrex tube, sealed with a flame, and subjected to heating at 100 ℃ in an oven for a duration of 24 hours. The colorless crystals were collected, washed with ethanol, and dried under vacuum for 6 h (11 mg, 69% based on Cu).

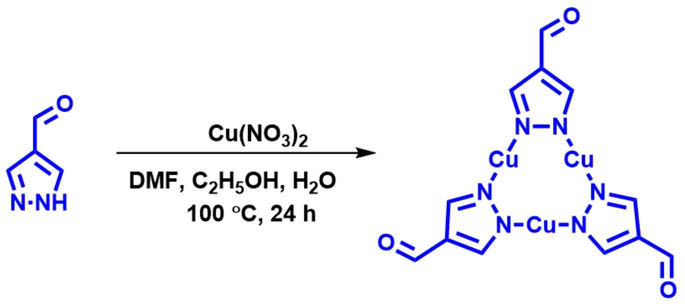

Cu-CTC (**1**)

Figure S1. The synthesis route of Cu-CTC (**1**).

3.2 Synthesis of PN-NH_2

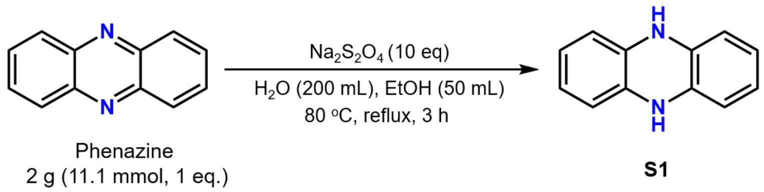

The **S1** was synthesized according to the literature method.[1] Add C_2H_5OH (50 mL) and H_2O (200 mL) to a 500 mL double-necked flask and then sparged with N_2 for 30 min. Phenazine (2.00 g, 11.1 mmol) and $Na_2S_2O_4$ (23.3 g, 111 mmol) were added under N_2 atmosphere, then it was heated to reflux under an N_2 atmosphere for 3 h. The light green powder was separated by filtration, washed with deoxygenated water, and dried under vacuum (1.45 g,71%). The **S1** was stored under an N_2 atmosphere.

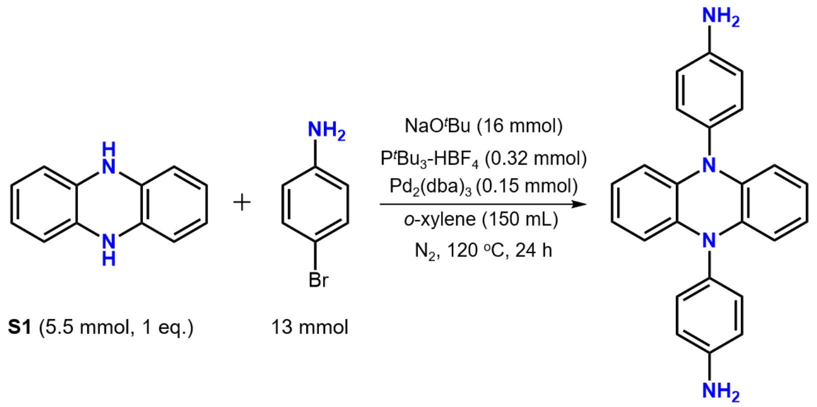

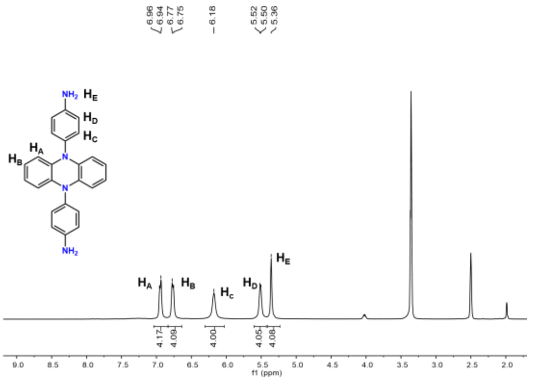

To a 350 mL Schlenk tube were added **S1** (1 g, 5.5 mmol), 4- bromoaniline (2.2 g, 13 mmol), $Pd_2(dba)_3$ (137 mg, 0.15 mmol), P^tBu_3-HBF_4 (92 mg, 0.32 mmol), NaO^tBu (1.53 g, 16 mmol) and anhydrous o-xylene (150 mL). The mixture was degassed by freeze-pump-thaw cycles three times, then it was heated to 140 ℃ for 16 h. After cooling to ambient temperature, the mixture was filtered, and the insoluble material was washed with dichloromethane. The combined organic phase was washed with water, and then the solvent was removed under reduced pressure. The resulting crude was purified by column chromatography

(petroleum ether/EtOAc, 2:1) to give PN-NH_2 (**3**) as purple powder (0.52 g, 25%). ^{1}H NMR (400 MHz, DMSO-d_6, 298 K) δ = 5.36 (4H, s), 5.50-5.52 (4H, m), 6.18 (4H, s), 6.76 (4H, d, *J* = 8.4 Hz), 6.94 (4H, d, *J* = 8.4 Hz) [ppm]. QTOF-HRMS: m/z calcd for $[M+H]^+$ $C_{24}H_{21}N_4^+$, 365.1768; found 365.1761.

3.3. Synthesis of JNMs

3.3.1 Synthesis of JNM-36

An *o*-DCB/*n*-BuOH/6 M aqueous trifluoroacetic acid (1/1/0.2 by vol.; 1.2 mL) mixture of Cu-CTC (**1**) (23.7 mg, 0.05 mmol) and AN-NH_2 (**2**) (27 mg, 0.075 mmol) was degassed in a Schlenk tube (10 mL) by three freeze-pump-thaw cycles. The tube was heated at 120 ℃ for 72 h, yielding a tawny solid. The solid was separated by filtration, washed with EtOH, DMF, and acetone, and dried under vacuum at 100 ℃ for 12 h (32.1 mg, 64%).

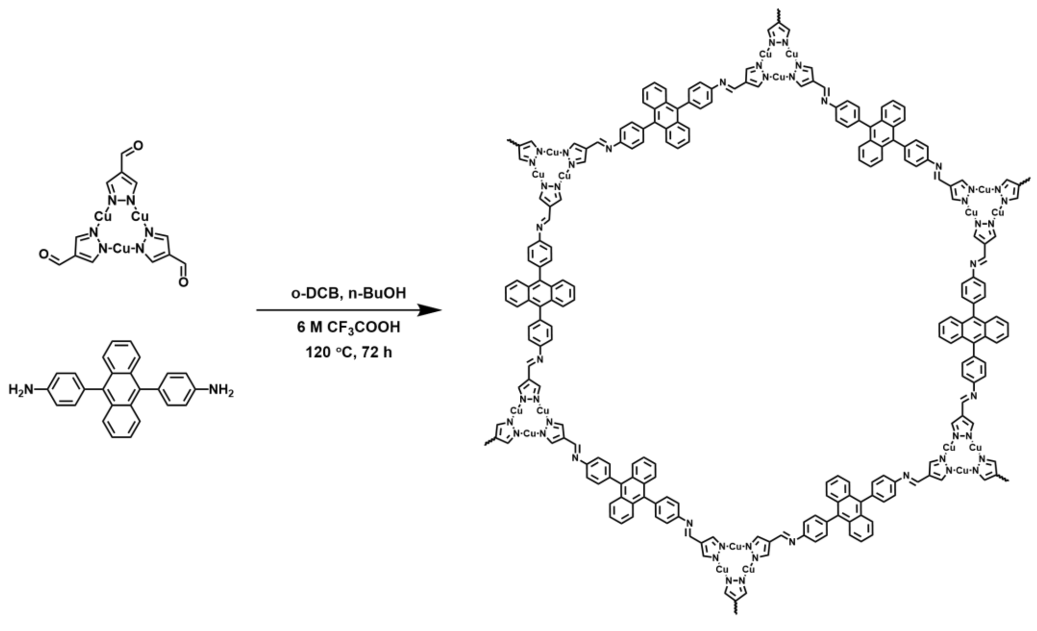

Figure S2. The synthesis route of **JNM-36**.

3.3.2 Synthesis of JNM-37

An *o*-DCB/*n*-BuOH/6 M aqueous trifluoroacetic acid (1/1/0.2 by vol.; 1.2 mL) mixture of Cu-CTC (**1**) (23.7 mg, 0.05 mmol) and PN-NH_2 (**3**) (27.3 mg, 0.075 mmol) was degassed in a Schlenk tube (10 mL) by three freeze-pump-thaw cycles. The tube was heated at 120 ℃ for 72 h, yielding an atropurpureus solid. The solid was separated by filtration, washed with EtOH, DMF, and acetone, and dried under vacuum at 100 ℃ for 12 h (30.3 mg, 60%).

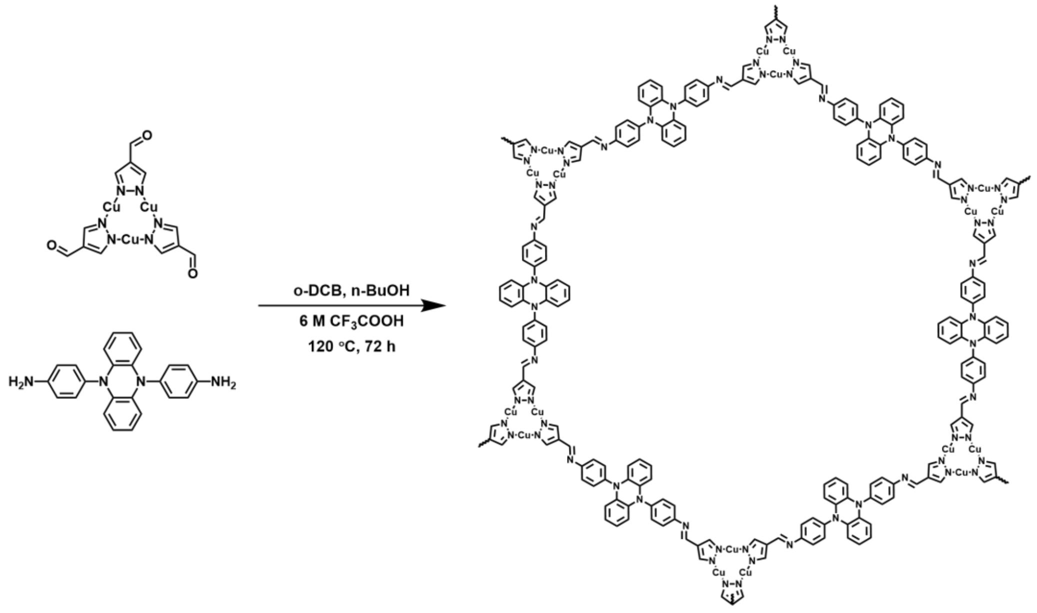

Figure S3. The synthesis route of **JNM-37**.

4. FT-IR spectra of JNMs.

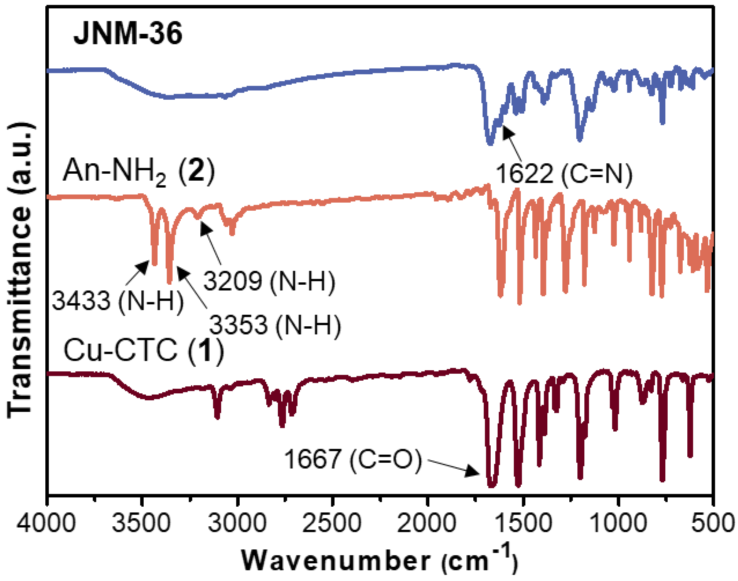

Figure S4. FT-IR spectra for **1**, **2,** and **JNM-36**.

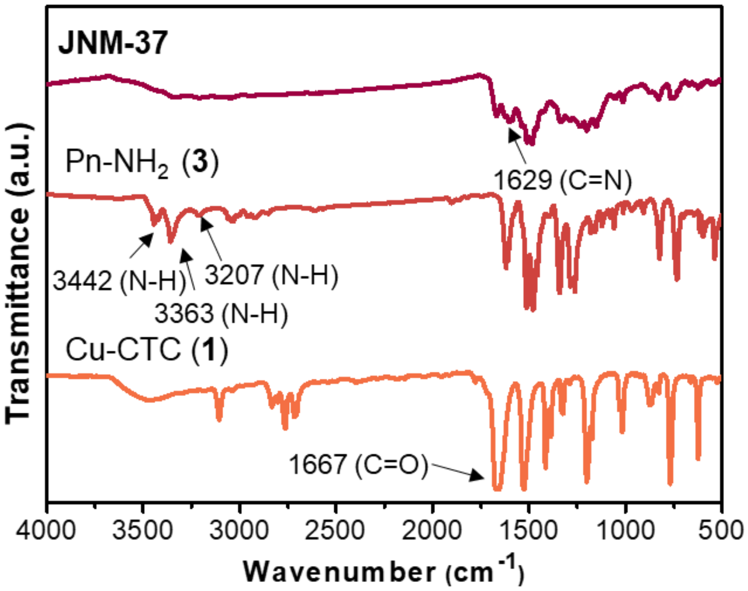

Figure S5. FT-IR spectra for **1**, **3,** and **JNM-37**.

5. Solid-state ^{13}C CP/MAS NMR spectra of JNMs.

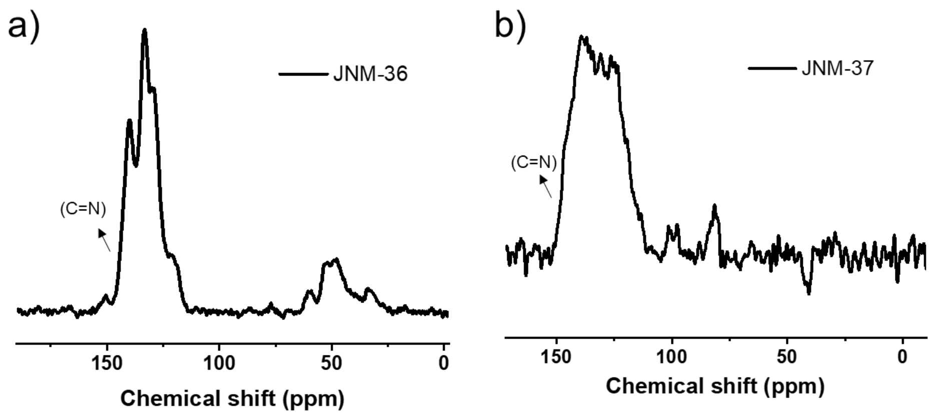

Figure S6. ^{13}C NMR spectra of (a) **JNM-36** and (b) **JNM-37**.

6. X-ray photoelectron spectroscopy (XPS)

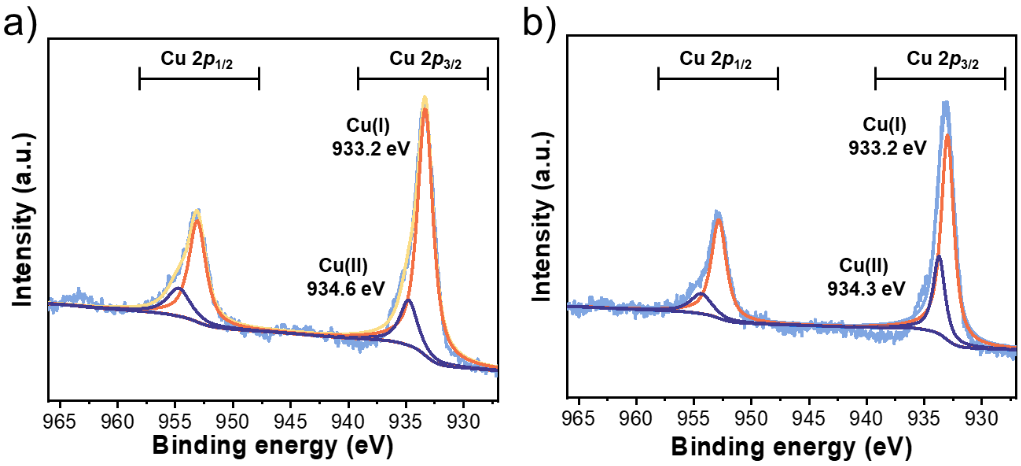

Figure S7. XPS for (a) **JNM-36** and (b) **JNM-37**.

7. Scanning electron microscopy (SEM)

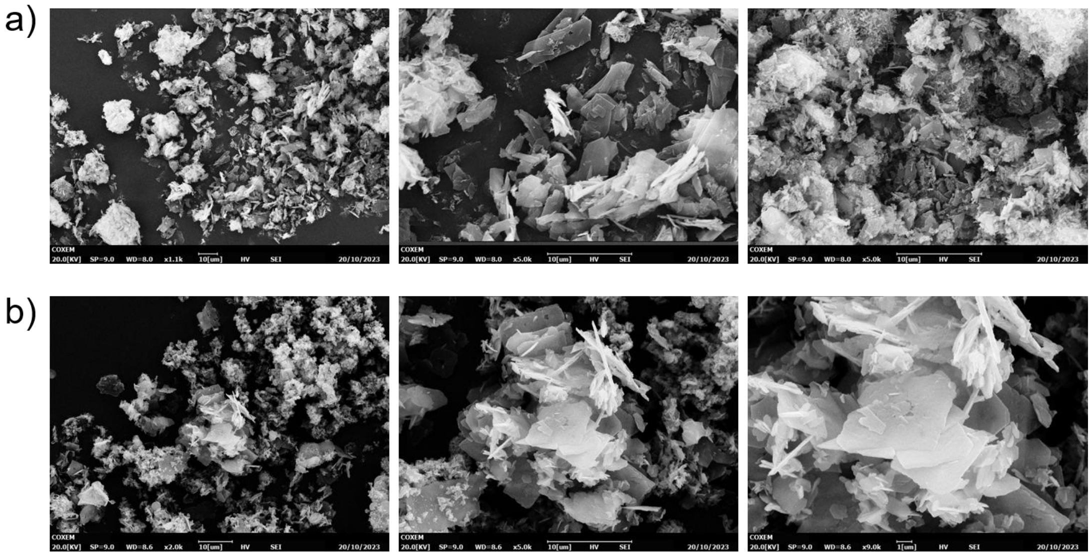

Figure S8. SEM images of (a) **JNM-36** and (b) **JNM-37.**

8. Energy dispersive X-ray spectroscopy (EDS)

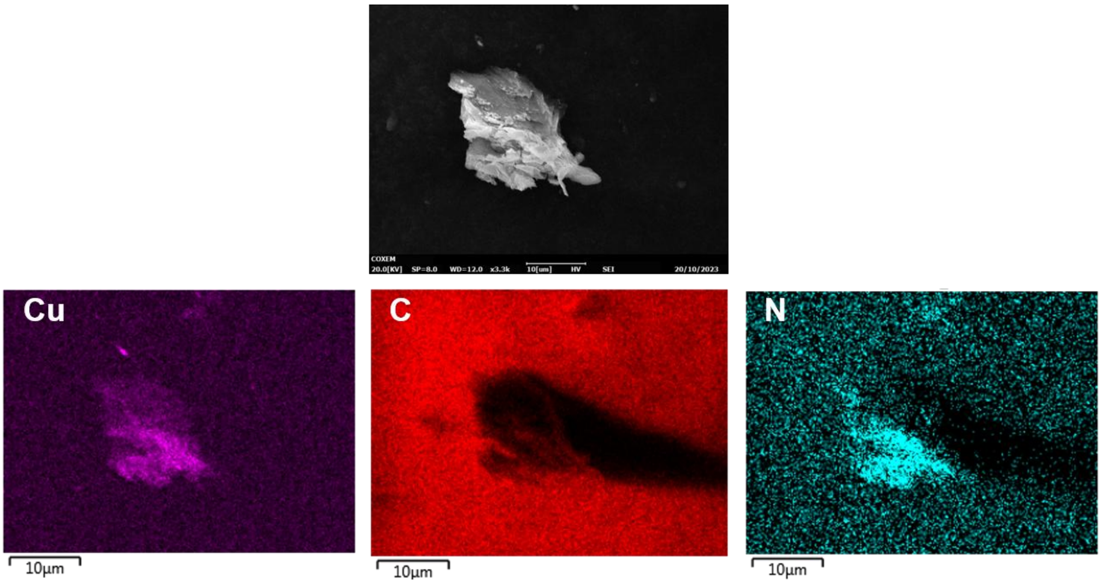

Figure S9. EDS mapping of **JNM-36**.

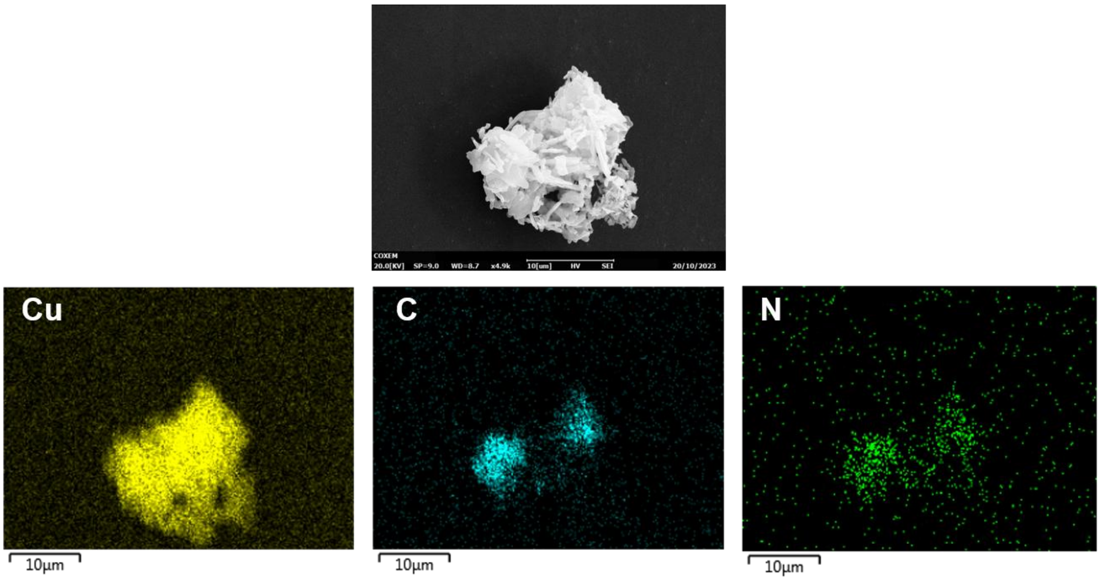

Figure S10. EDS mapping of **JNM-37**.

9. Structural simulation

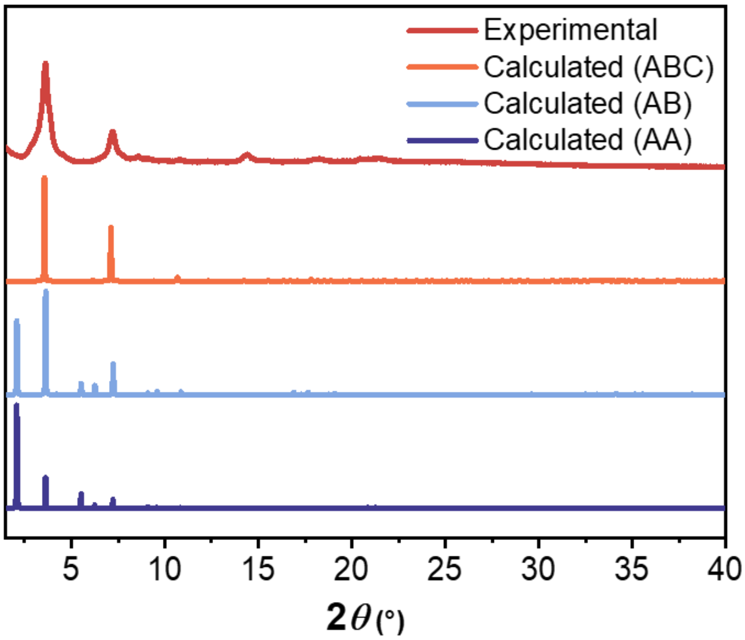

Figure S11. PXRD patterns of **JNM-36** with the experimental profiles in reddish brown and calculated profiles of AA (blue purple), AB (sky blue), and ABC (orange) packing modes.

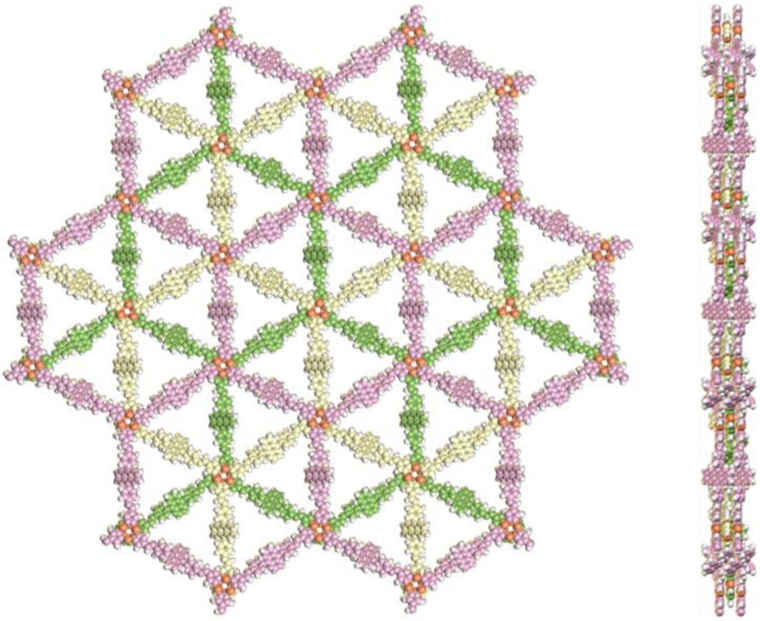

Figure. S12. Space-filling mode of **JNM-36** in the ABC stacking model viewed from (left) *c* axis and (right) *a* axis.

Table S1. Atomic coordinates of the ABC-stacking mode of **JNM-36**.

Space group: *R*-3<br>$a = b = 49.15$ Å, and $c = 5.45$ Å<br>$\alpha = \beta = 90°$, and $\gamma = 120°$<br>residual factors: $R_p = 2.26\%$, $R_{wp} = 3.28\%$.			
	X	Y	Z
C1	-0.23835	-0.60862	0.07813
N2	-0.26954	-0.62942	0.08344
N3	-0.27416	-0.65845	0.08523
C4	-0.24609	-0.65732	0.08215
C5	-0.2227	-0.62576	0.07652
Cu6	-0.31653	-0.69529	0.08442
C7	-0.81151	-0.38665	-0.06584
C8	-0.47483	-0.49174	-0.167
N9	-0.83067	-0.41678	-0.03685
C10	-0.8817	-0.41832	-0.11948
C11	-0.86459	-0.43217	-0.02532
C12	-0.88114	-0.46258	0.07564
C13	-0.91473	-0.43464	-0.10933
C14	-0.93118	-0.46503	-0.00719
C15	-0.91417	-0.47887	0.08594
C16	-0.45881	-0.01664	0.50449
C17	-0.96721	-0.51593	-0.34756
C18	-0.98327	-0.50788	-0.17156
C19	-0.96636	-0.48279	-0.00398
C20	-0.96685	-0.44998	0.33704
C21	-0.98383	-0.54078	-0.51033
H22	-0.22795	-0.58316	0.07447
H23	-0.24296	-0.67776	0.08102
H24	-0.82011	-0.3701	-0.07445
H25	-0.86987	-0.39529	-0.20534
H26	-0.86833	-0.47369	0.1465
H27	-0.92746	-0.42364	-0.18208
H28	-0.92647	-0.50241	0.16316
H29	-0.45216	-0.02931	0.63121
H30	-0.94169	-0.50311	-0.36216
H31	-0.9413	-0.43634	0.34286
H32	-0.97122	-0.54688	-0.64215

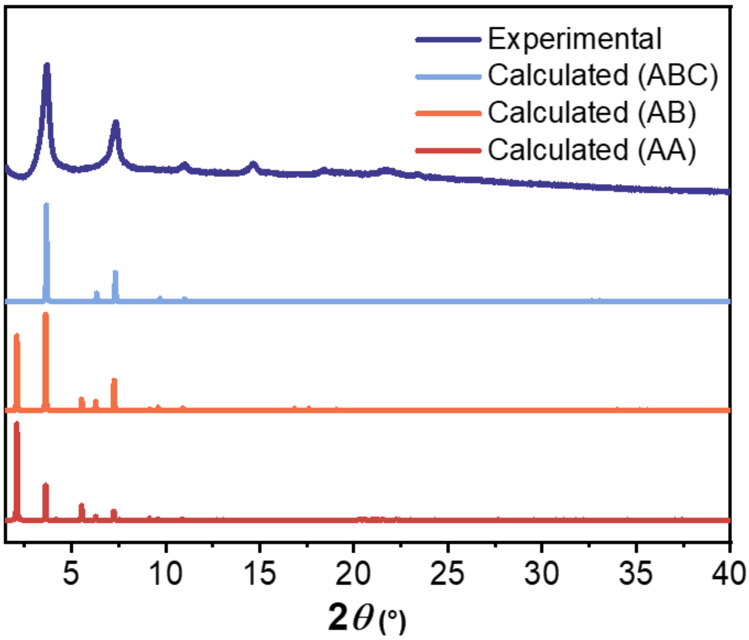

Figure S13. PXRD patterns of **JNM-37** with the experimental profiles in purple and calculated profiles of AA (reddish brown), AB (orange), and ABC (sky blue) packing modes.

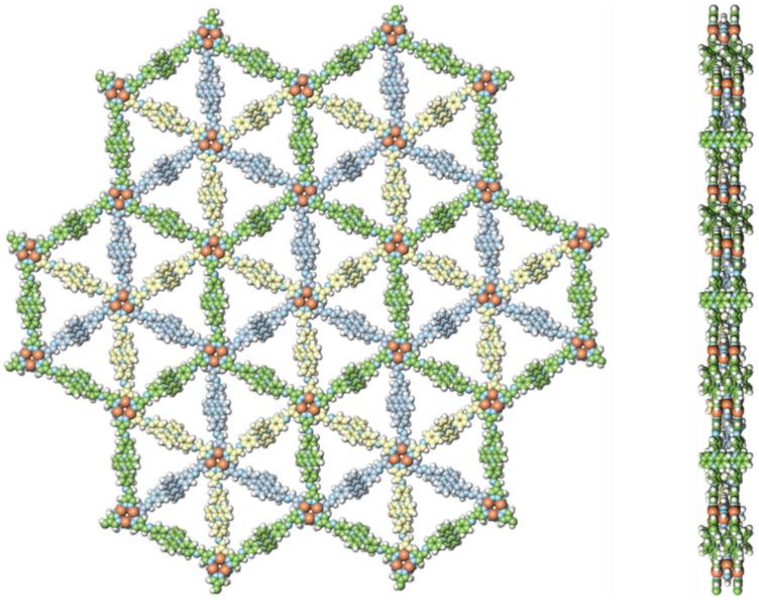

Figure. S14. Space-filling mode of **JNM-37** in the ABC stacking model viewed from (left) *c* axis and (right) *a* axis.

Table S2. Atomic coordinates of the ABC-stacking mode of **JNM-37**.

Space group: *R*-3<br>$a = b$ = 48.26 Å, and c = 5.49 Å<br>$\alpha = \beta$ = 90 °, and γ = 120 °<br>residual factors: R_p = 1.68%, R_{wp} = 2.63%.			
	X	Y	Z
C1	-0.23809	-0.60857	0.07842
N2	-0.26937	-0.62939	0.0834
N3	-0.27404	-0.65851	0.08514
C4	-0.24592	-0.65743	0.08231
C5	-0.22243	-0.6258	0.07703
Cu6	-0.31654	-0.69541	0.08436
C7	-0.81186	-0.38661	-0.06724
C8	-0.47409	-0.49099	-0.16743
N9	-0.83113	-0.41682	-0.0394
C10	-0.8822	-0.41824	-0.12285
C11	-0.86513	-0.43221	-0.02882
C12	-0.88181	-0.46273	0.07118
C13	-0.91532	-0.43454	-0.11315
C14	-0.93188	-0.46498	-0.01143
C15	-0.91493	-0.47897	0.08099
C16	-0.45802	-0.01679	0.50263
C17	-0.96728	-0.51572	-0.35493
C18	-0.98338	-0.50808	-0.17714
N19	-0.96608	-0.48215	-0.00839
C20	-0.96673	-0.44883	0.33334
C21	-0.98393	-0.54101	-0.51422
H22	-0.22763	-0.58304	0.07494
H23	-0.24282	-0.67795	0.08131
H24	-0.82045	-0.36998	-0.07567
H25	-0.87027	-0.39513	-0.20807
H26	-0.86905	-0.47394	0.14172
H27	-0.92809	-0.42352	-0.1854
H28	-0.92741	-0.50259	0.15718
H29	-0.45103	-0.02952	0.62604
H30	-0.94175	-0.50226	-0.37234
H31	-0.94113	-0.43476	0.33378
H32	-0.97132	-0.54692	-0.64724

10. Nitrogen adsorption-desorption measurements

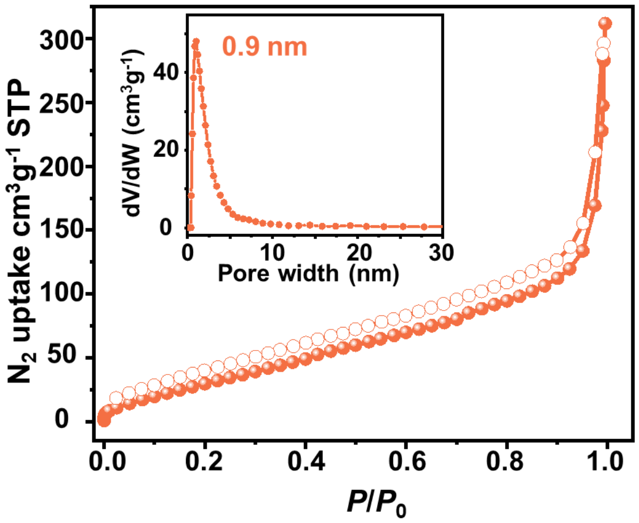

Figure S15. Nitrogen adsorption-desorption isotherms of **JNM-36** at 77 K. Inset, showing pore size distribution profiles of **JNM-36**

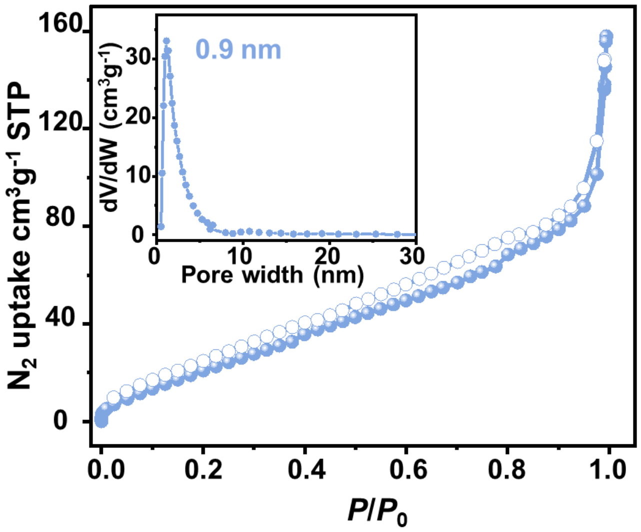

Figure S16. Nitrogen adsorption-desorption isotherms of **JNM-37** at 77 K. Inset, showing pore size distribution profiles of **JNM-37**

11. Transmission electron microscopy (TEM)

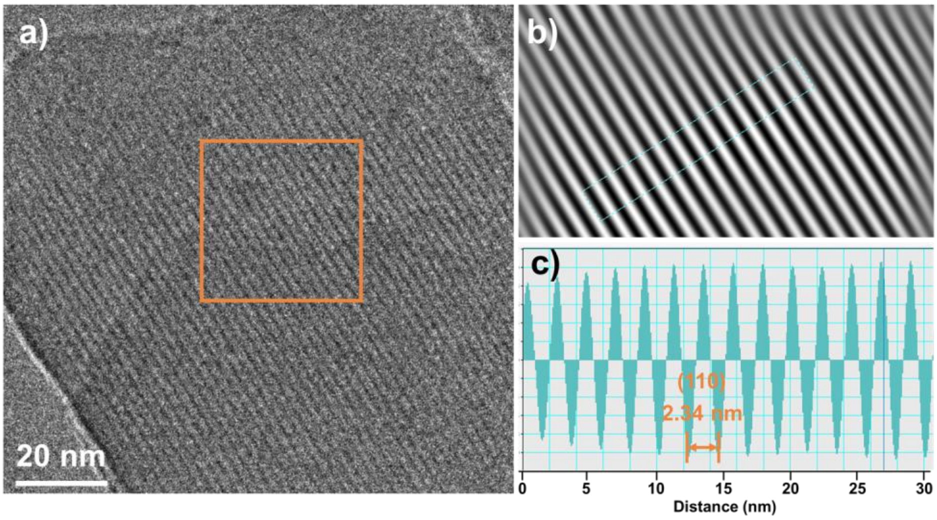

Figure S17. HR-TEM images of **JNM-36**.

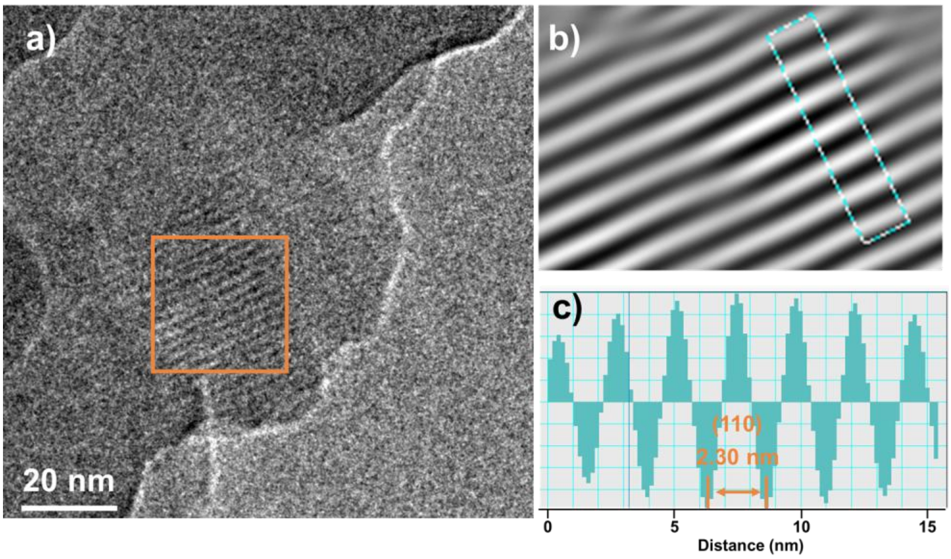

Figure S18. HR-TEM images of **JNM-37**.

12. Thermogravimetric analysis (TGA).

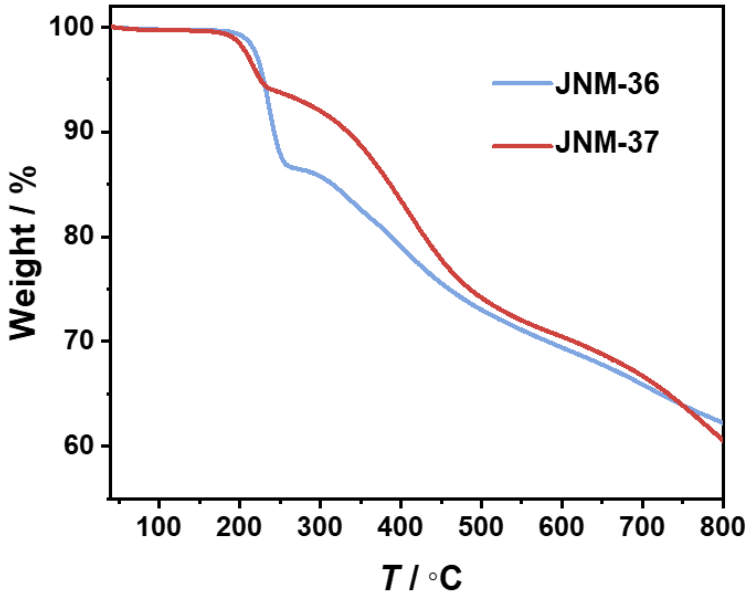

Figure S19. TGA curves of **JNM-36** and **JNM-37** under N_2 atmosphere.

13. Stability in various solvents.

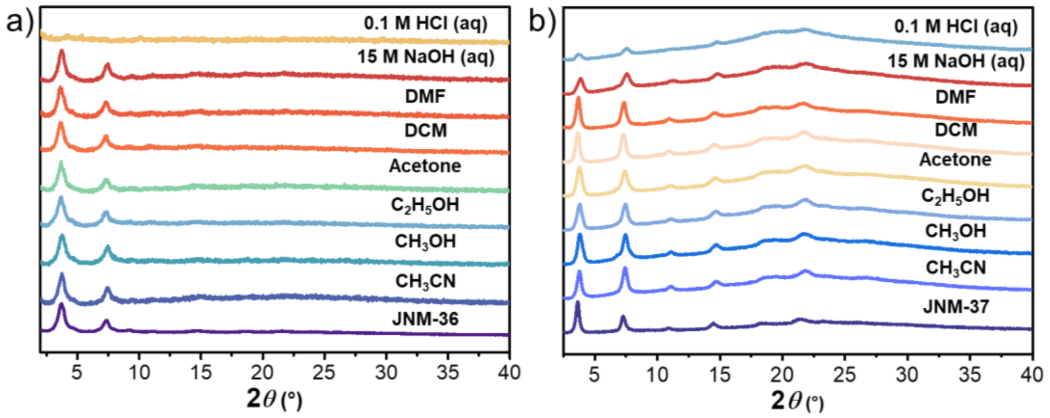

Figure S20. (a-b) PXRD patterns of **JNM-36** and **JNM-37** soaked in different organic solvents and different acid/base solutions.

14. Mott-Schottky plots.

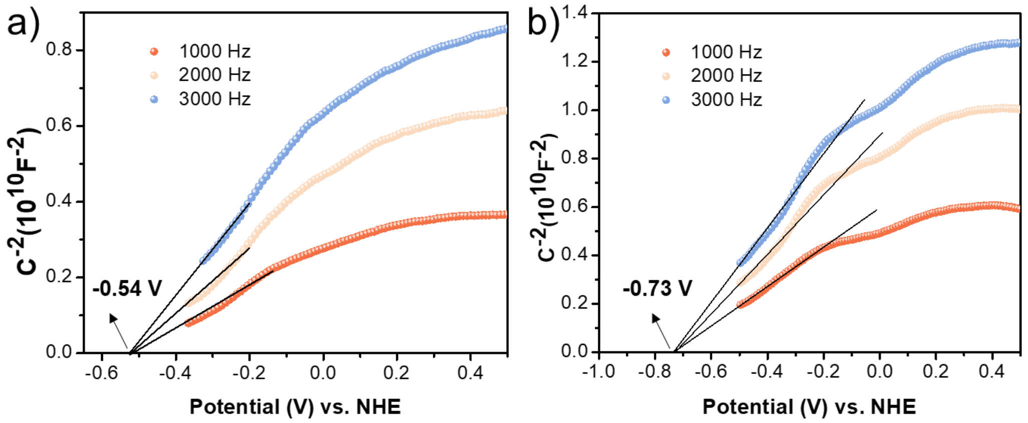

Figure S21. Mott-Schottky plots for (a) **JNM-36** and (b) **JNM-37** in 0.5 M solution of Na_2SO_4.

15. Photocatalytic application

15.1 Air system

15.1.1 Synthesis of substrates

General procedure 1

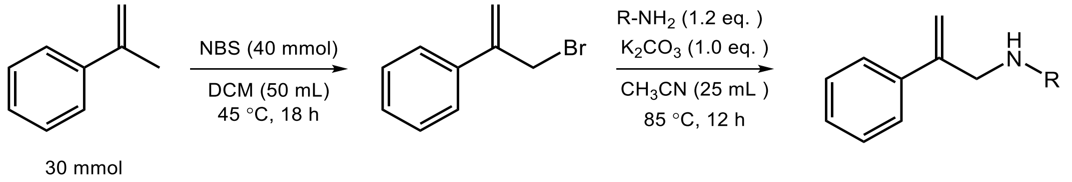

To a solution of prop-1-en-2-ylbenzene (1 equiv., 30 mmol) in DCM (50 mL) was added NBS (1.33 equiv., 40 mmol) at room temperature. After stirring for 18 hours at 45 ℃ and then the solvent was evaporated under reduced pressure. The residue was purified by column chromatography (petroleum ether /EtOAc, 40:1) to obtain a light yellow liquid.

The mixture of (3-bromoprop-1-en-2-yl)-benzene (3.0 mmol, 1.0 equiv.), primary amine (3.6 mmol, 1.2 equiv.), and K_2CO_3 (3.0 mmol, 1.0 equiv.) in 20 mL CH_3CN were refluxed overnight. After the solvent was evaporated under reduced pressure and then the mixture was extracted with DCM (50 mL × 3). The product was purified by column chromatography (petroleum ether /EtOAc, 10:1). The NMR data of all substrates were consistent with the literature previously.[2, 3]

General procedure 2

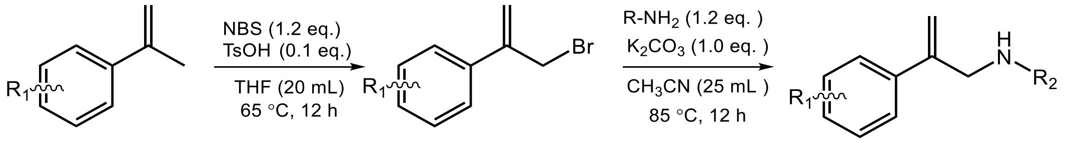

To a solution of prop-1-en-2-ylbenzene with substituent (1 equiv., 1.3 mmol) in THF (20 mL) were added NBS (1.2 equiv., 1.56 mmol), and TsOH (0.13 mmol, 0.1 equiv.) at room temperature. After stirring for 18 hours at 65 ℃ and then the solvent was evaporated under reduced pressure. The residue was purified by column chromatography (petroleum ether /EtOAc, 40:1).

The mixture of (3-bromoprop-1-en-2-yl)-benzene with substituent (3.0 mmol, 1.0 equiv.), primary amine (3.6 mmol, 1.2 equiv.), and K_2CO_3 (3.0 mmol, 1.0 equiv.) in 20 mL CH_3CN were refluxed overnight. After the solvent was evaporated under reduced pressure and then the mixture was extracted with DCM (50 mL × 3). The product was purified by column chromatography (petroleum ether /EtOAc, 10:1). The NMR data of all substrates were consistent with the literature previously.[2, 3]

15.1.2 General procedure for hydroxytrifluoromethylation of allylamines

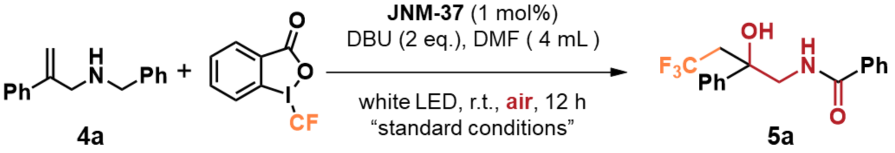

4a (89.2 mg, 0.4 mmol), Togni's reagent II (139 mg, 0.44 mmol), DBU (120 µL, 0.8 mmol), and the corresponding amount of catalyst (1 mol% based on Cu) were added to an oven-dried Schlenk tube (10 mL). The reaction was stirred under 30 W white LED for 12 h at room temperature. Then, the resulting mixture was filtered and concentrated in vacuo. 1,4-difluorobenzene (0.4 mmol) was added as an internal standard, and the reaction mixture was analyzed by ^{19}F NMR. Finally, the resulting crude was purified by column chromatography on silica gel (petroleum ether/EtOAc, 5:1) to give the CF_3-containing β-amino alcohols product.

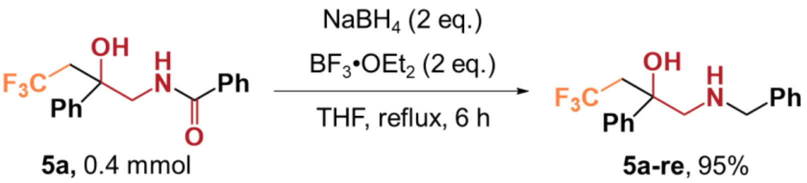

Figure S22. Reduction of the amide of **5a** to the amine by using $NaBH_4$-BF_3•OEt_2.

15.1.3 The catalytic kinetics

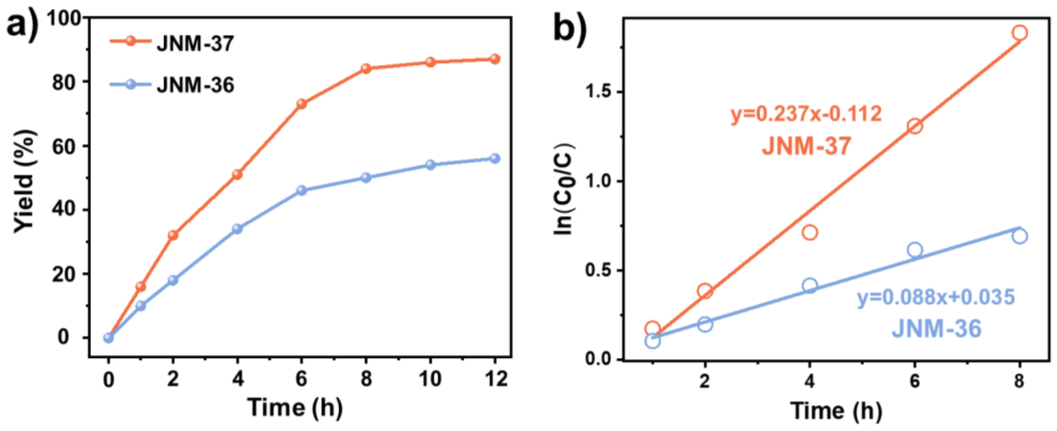

Figure S23. (a) Plots of yield vs reaction time for **JNM-36** and **JNM-37**. (b) Estimation of rates for the reaction by using different catalysts.

15.1.4 General procedure for the catalytic cycle test

The catalytic recyclability of **JNM-37** catalyst was studied detailedly after five cycles. After each catalytic run, the mixture was separated by centrifuging the mixture, and the liquid was analyzed for the product yield by ^{19}F NMR, and the solid was washed 3 times with C_2H_5OH and DCM. Then it was dried in a vacuum at 100 ℃ for 3 h and used for the next run.

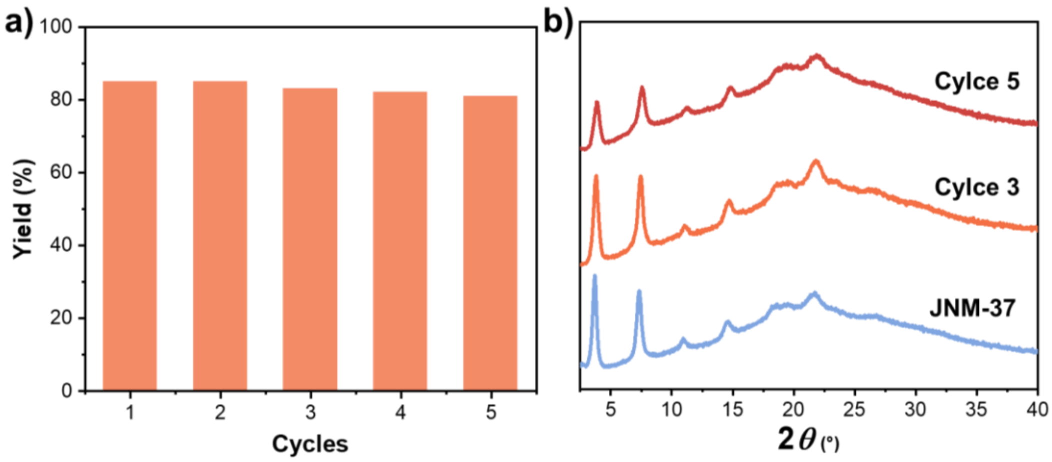

Figure S24. (a) Recyclability of **JNM-37**-catalyzed hydroxytrifluoromethylation of **4a** with O_2. The reported yield is based on ^{19}F NMR analysis. (b) PXRD patterns of **JNM-37** after five catalytic cycles.

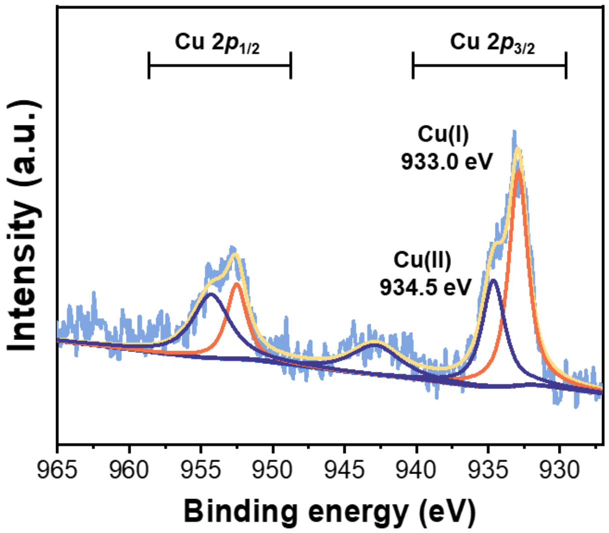

Figure S25. XPS for **JNM-37** after catalyst reaction.

15.1.5 Mechanistic Studies

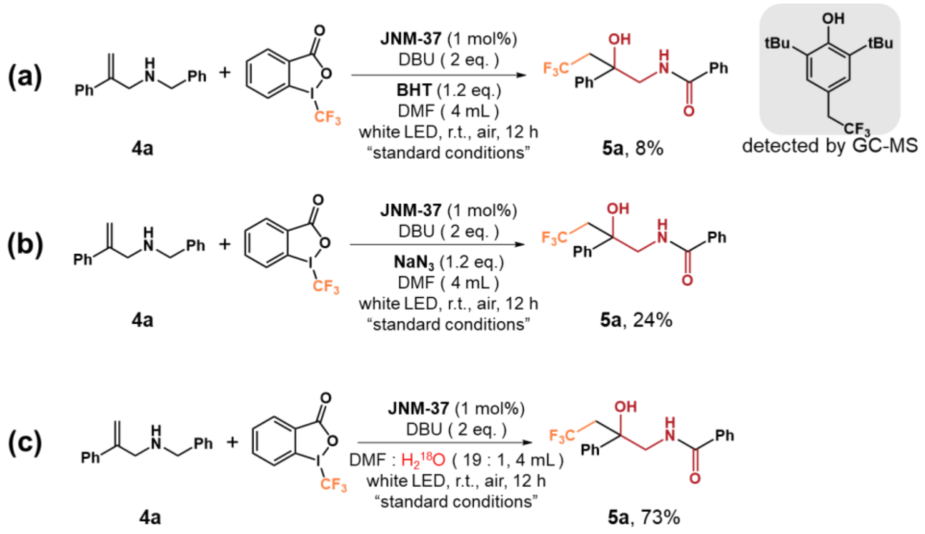

Figure S26. Adding a free radical scavenger to the reaction. (a) 2,6-di-tert-butyl-4-methylphenol, BHT. (b) $^1O_2$ quencher such as sodium azide (NaN_3). (c) ^{18}O-labeling studies.

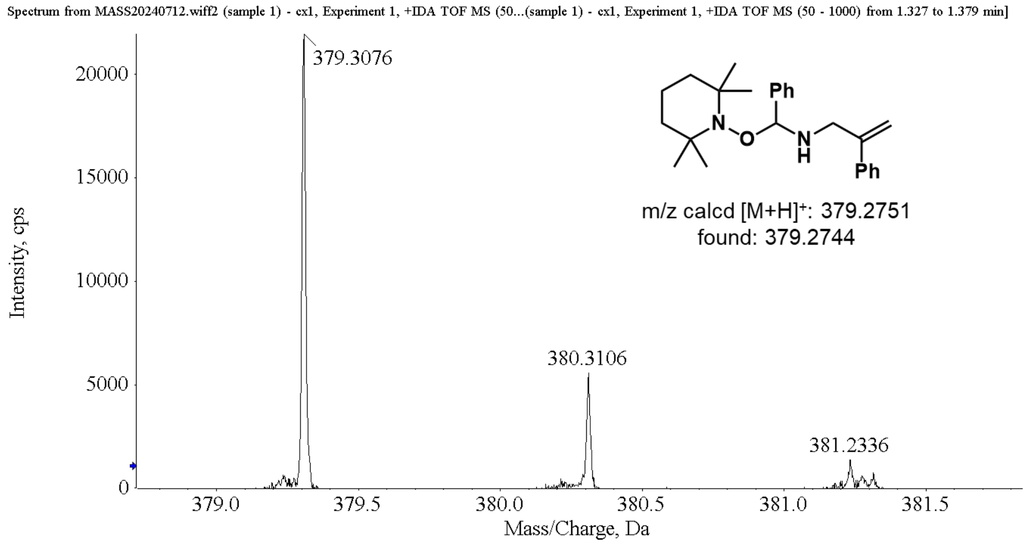

Figure S27. QTOF-HRMS spectra for TEMPO-**4a**.

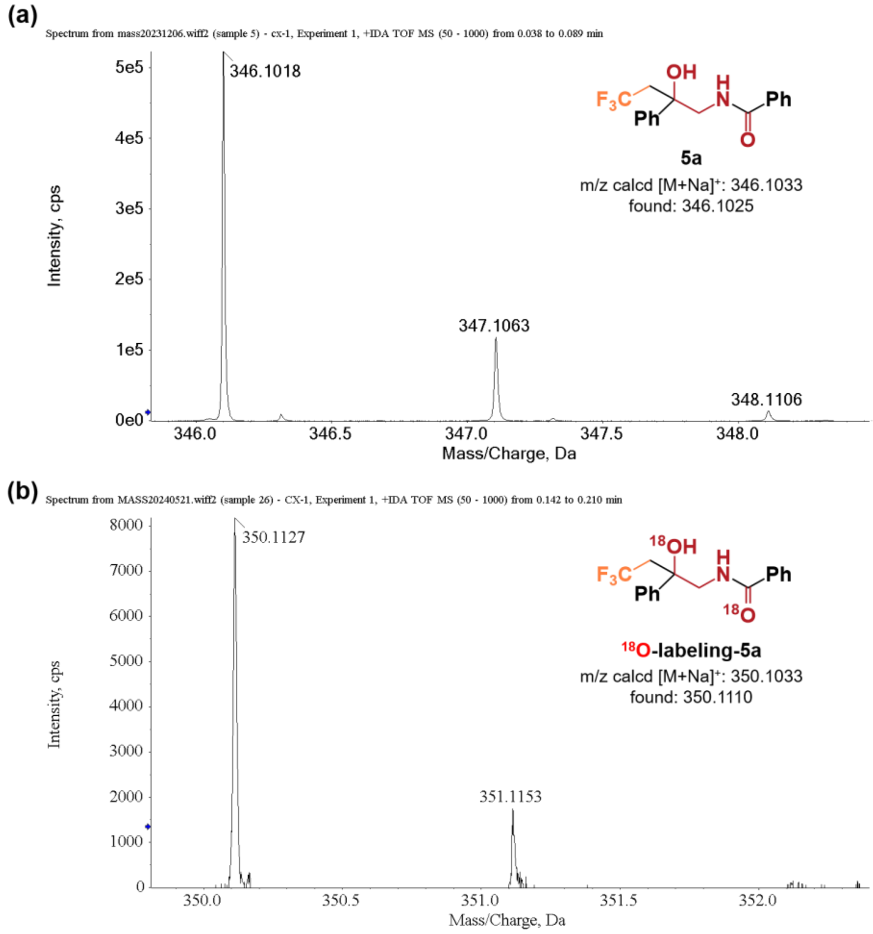

Figure S28. QTOF-HRMS spectra for (a) **5a** and (b) ^{18}O-labeling-**5a**.

15.1.6 Catalytic experiment under natural sunlight

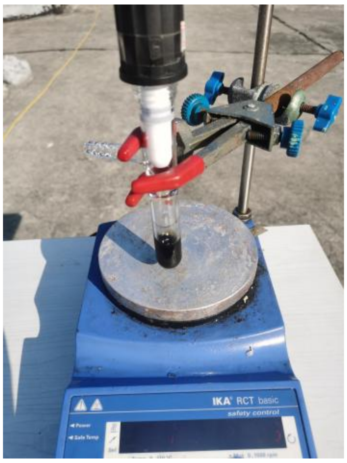

Figure S29. Pictures of catalytic experiments under natural sunlight.

15.1.7 Continuous flow experiments

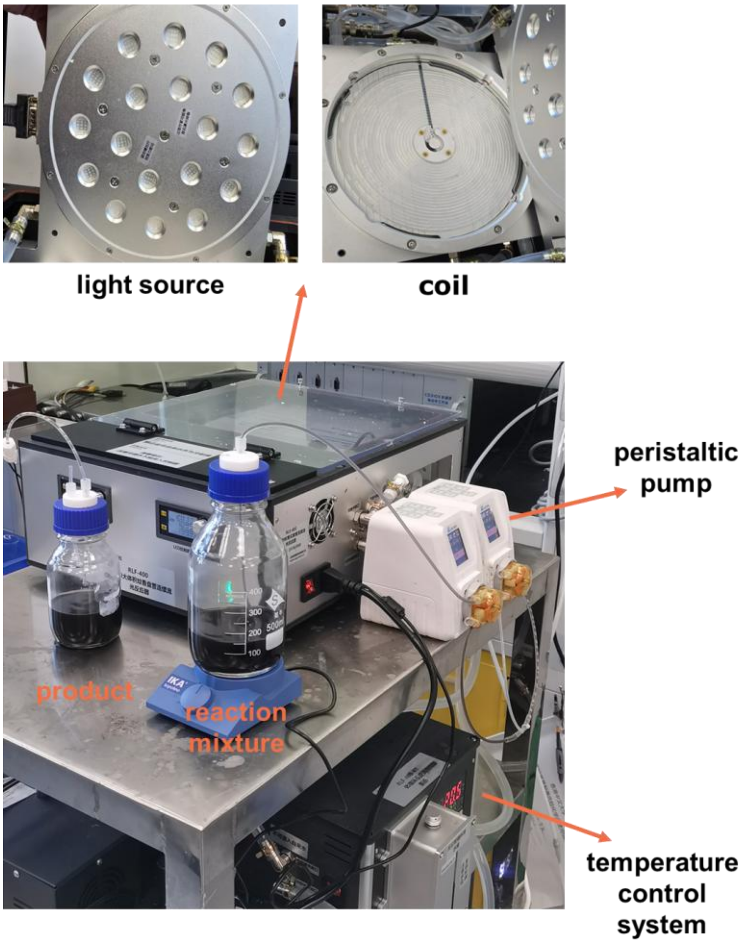

Figure S30. Demonstration diagram of continuous flow experiment.

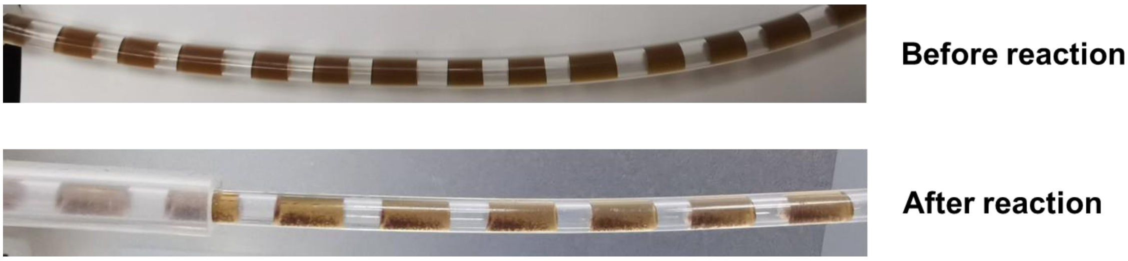

Figure S31. Images of the droplets before and after the reaction.

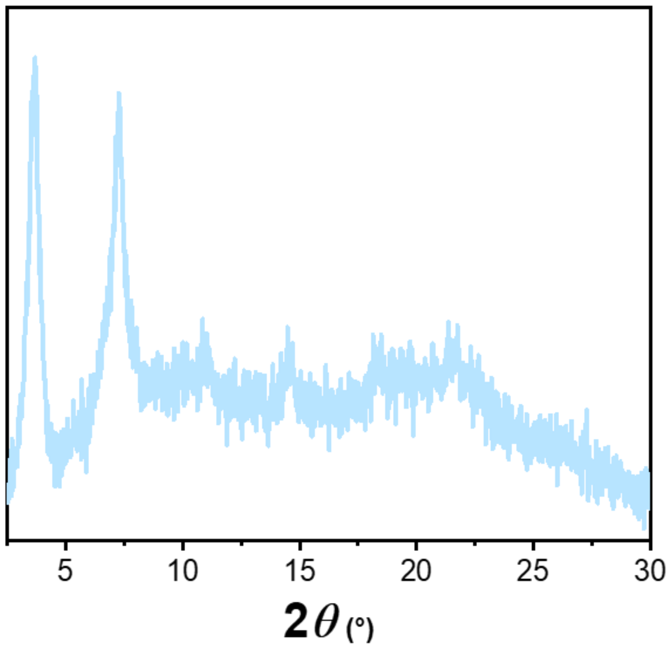

Figure S32. PXRD patterns of **JNM-37** after continuous flow experiments.

15.1.8. EPR experiments

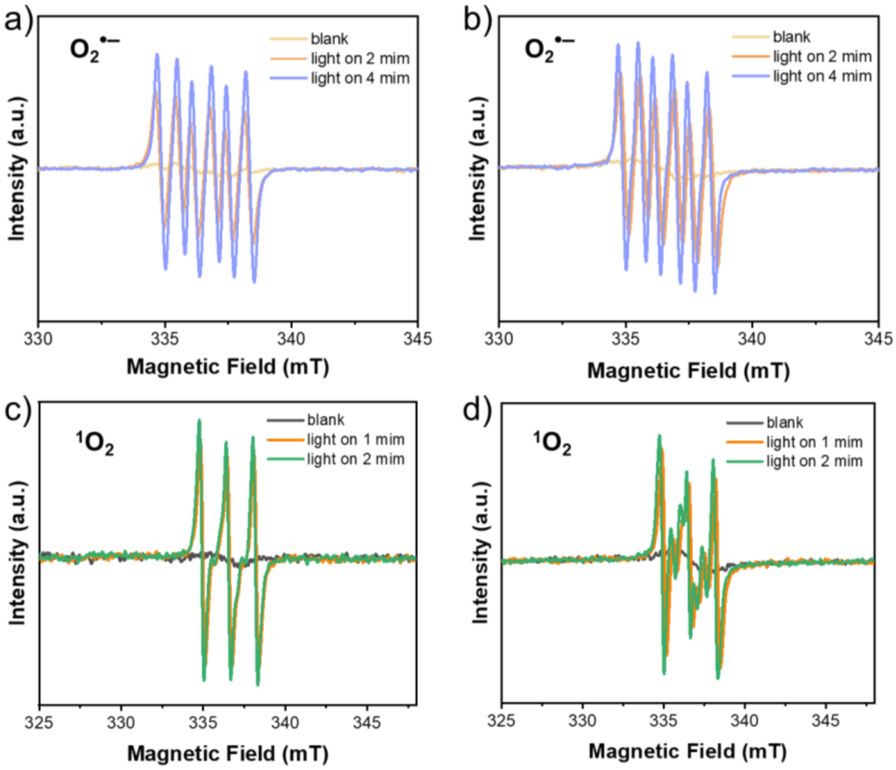

Figure S33. EPR spectra of **JNM-36** (a, c) and **JNM-37** (b, d) in the presence of DMPO (a-b) and TEMP (c-d) under white light irradiation.

15.2 CO_2 system

15.2.1 Optimization of reaction conditions

Table S3. Condition optimization of JNM-37-catalyzed oxytrifluoromethylation of allylamines with CO_2.

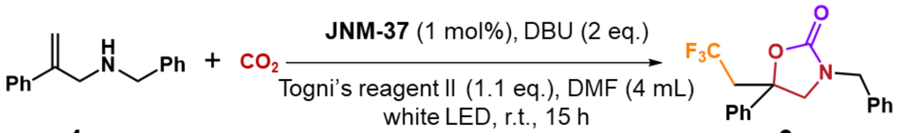

4a 6a

entry	change from the "standard conditions"	yield[b] (%)
1	none	90 (85)[c]
2	no **JNM-37**	7
3	no light	5
4	no DBU	12
5	N_2 instead of CO_2	<1
6	K_2CO_3 instead of DBU	39
7	DABCO instead of DBU	81
8	MTBD instead of DBU	28
9	DMF : H_2O (10 : 1) instead of DMF	48
10	CH_3CN instead of DMF	60
11	CH_3OH instead of DMF	72
12	**JNM-36** instead of **JNM-37**	59
13	**1** instead of **JNM-37**	8
14	**3** instead of **JNM-37**	13
15	Cu_2O instead of **JNM-37**	18
16	$Cu(NO_3)_2$ instead of **JNM-37**	68

[a] Reactions condition: **4a** (0.4 mmol), Togni's reagent II (1.1 eq.), CO_2 (0.1 MPa), DBU (2 eq.), solvent (4 mL), white LED, r.t. [b] NMR yield with ^{19}F NMR analysis using 1,4-difluorobenzene as internal standard. [c] isolated yield.

15.2.2 The catalytic kinetics

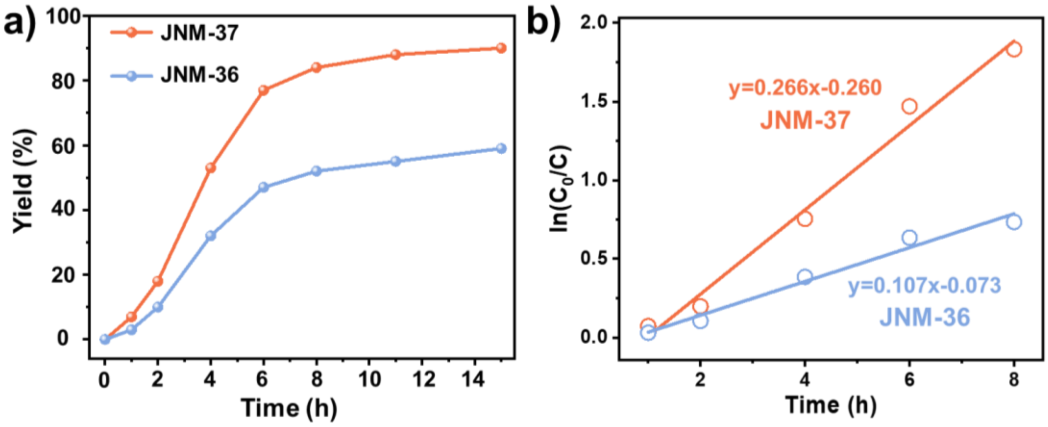

Figure S34. (a) Plots of yield vs reaction time for **JNM-36** and **JNM-37**. (b) Estimation of rates for the reaction by using different catalysts.

15.2.3 General procedure for the catalytic cycle test

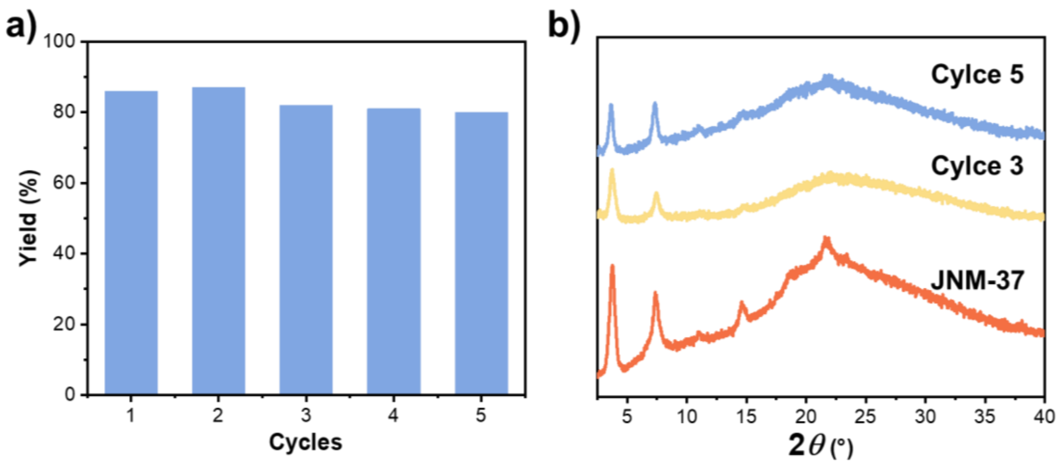

Figure S35. (a) Recyclability of **JNM-37**-catalyzed oxytrifluoromethylation of **4a** with CO_2. The reported yield is based on ^{19}F NMR analysis. (b) PXRD patterns of **JNM-37** after five catalytic cycles.

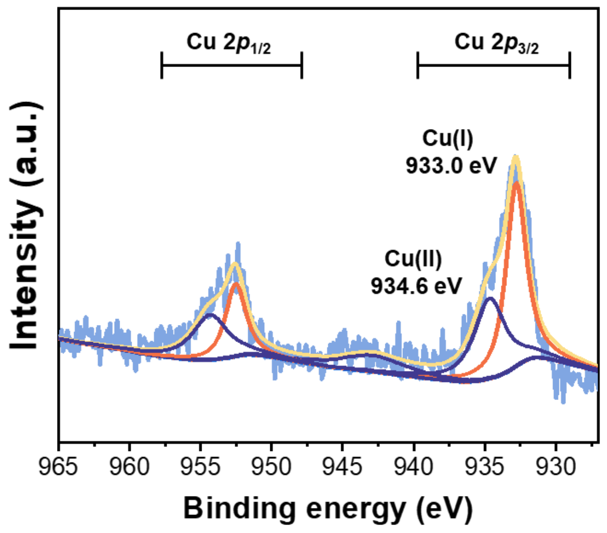

Figure S36. XPS for **JNM-37** after catalyst reaction.

15.2.4. isotope labeling experiments

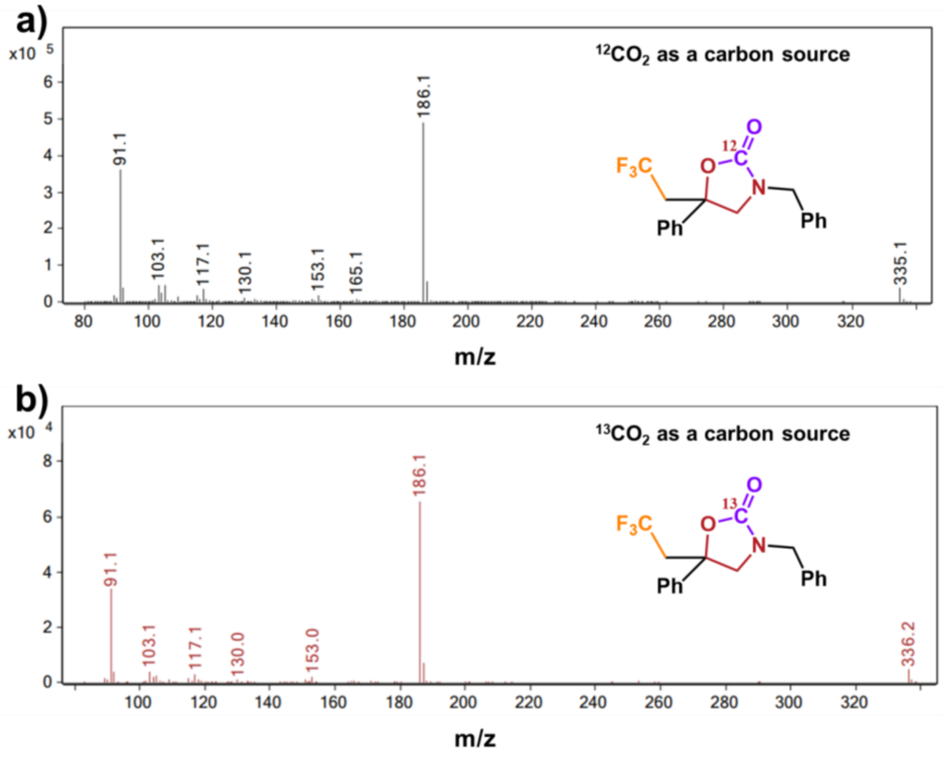

Figure S37. GC-MS spectra of (a) unlabeled **6a** and (b) ^{13}C-labeled **6a**.

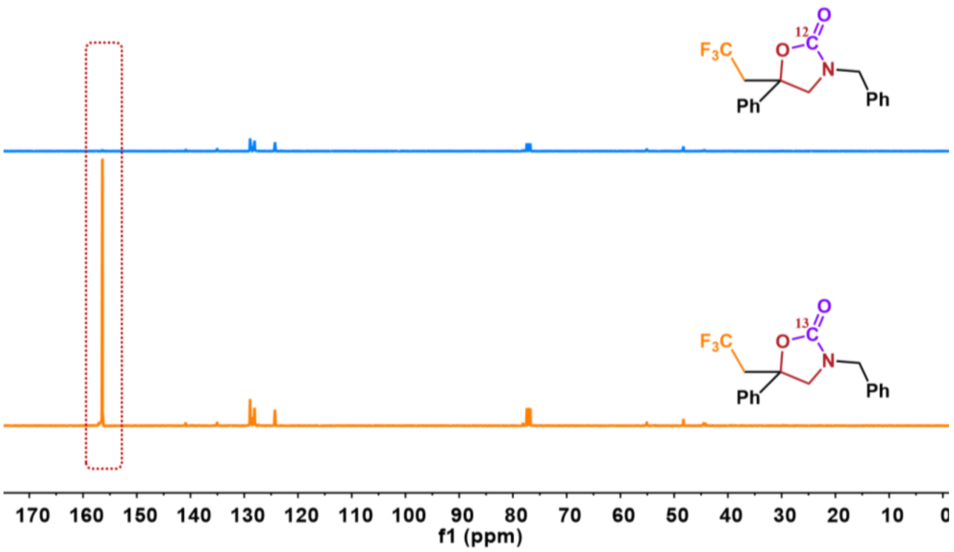

Figure S38. ^{13}C NMR (101 MHz, 298 K, $CDCl_3$) spectras of (top) unlabeled **6a** and (bottom) ^{13}C-labeled **6a**.

15.2.5 Mechanistic Studies

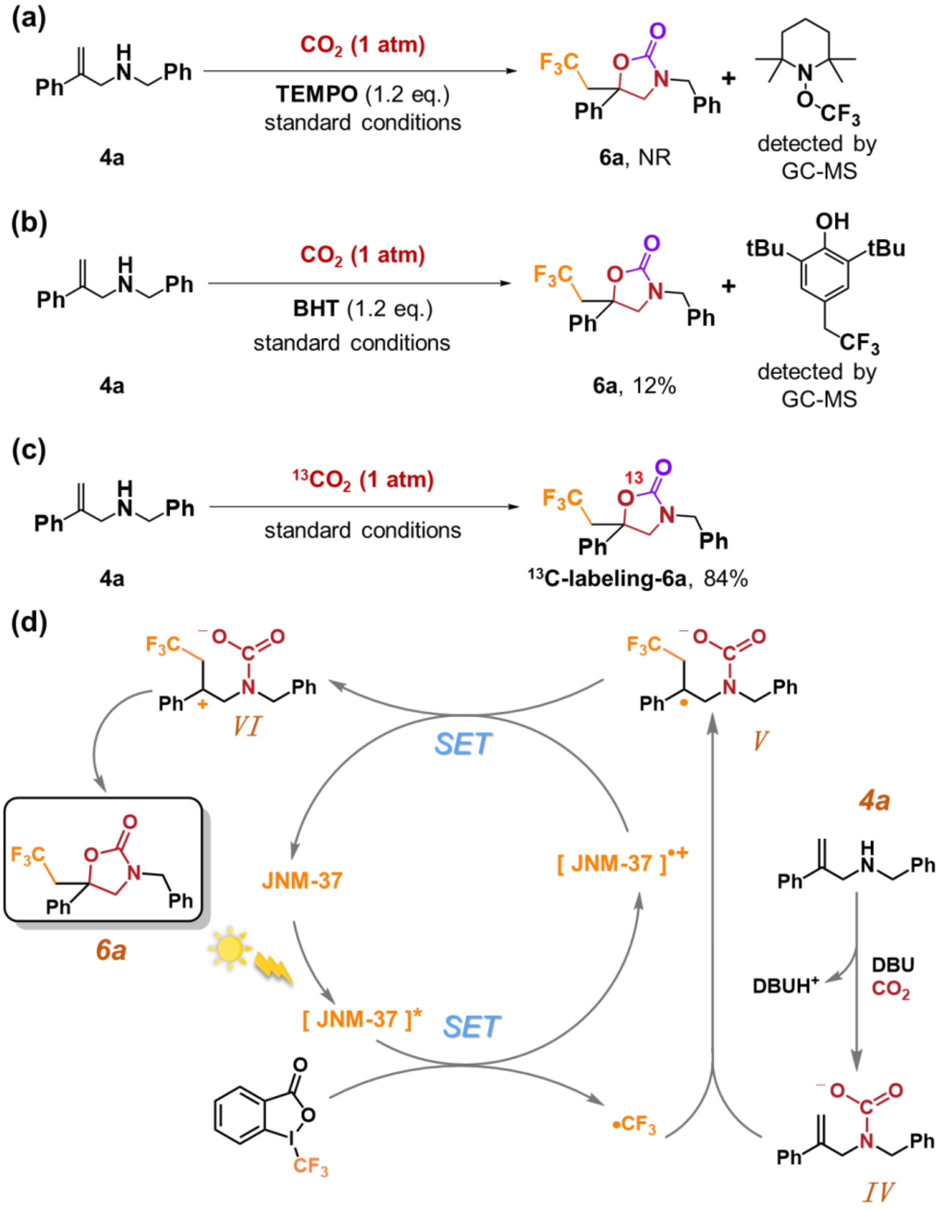

Figure S39. (a-b) Radical trapping experiments for oxytrifluoromethylation of allylamines with CO_2. (c) ^{13}C-isotope labeling experiments. (d) Possible mechanism of oxytrifluoromethylation of allylamines with CO_2.

15.2.6 Catalytic experiment under natural sunlight

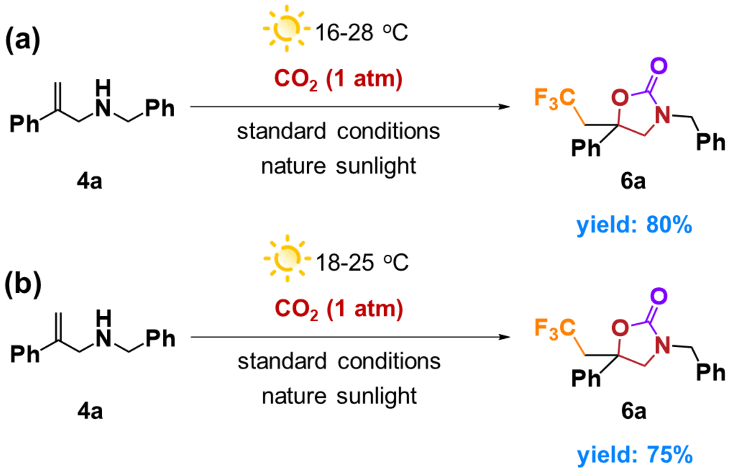

Figure S40. (a-b) The oxytrifluoromethylation of **4a** with CO_2 under different natural sunlight irradiations;

16. Computational Results

Density functional theory (DFT) as well as time-dependent density functional theory (TDDFT) computations were performed for **JNM-36** and **JNM-37** by using Gaussian 09E software.[4] The following level of theory was adopted in all the calculations: (1) Functional: the hybrid Perdew, Burke, and Ernzerhof functional (PBE0)[5] in conjunction with D3(BJ) dispersion correlation;[6, 7] (2) Basis sets: the LANL2DZ[8, 9] effective core potential (ECP) was used for Cu and the 6-31G(d,p)[10] basis set was used for the other atoms. Firstly, the geometrical optimization was performed for **JNM-36** and **JNM-37** to obtain their stable ground-state (S_0) geometries by the restricted B3LYP method, confirmed by the absent of imaginary frequencies (NIMG = 0). Then, TDDFT calculations were performed for the S_1 singlet-triplet spin-forbidden transitions were calculated based on the optimized S_0 structures. By using Multiwfn program,[11] the TDDFT results were extracted from the Gaussian output files (log files), and the cub files for drawing the electron density differences (EDD) maps were obtained by the hole-electron analysis function[12] of Multiwfn software after inputting the logfiles and the formatted checkpoint (fchk) files, the isovalue of the EDD maps was 0.001 a.u. In addition, Visual Molecular Dynamics (VMD)[13] after inputting the cube (cub) files. Electrostatic potential (ESP) surfaces were drawn for the monomer of **JNM-36** and **JNM-37**, by mapping ESP on the van der Walls isosurfaces of the model with isovalue = 0.0004 a.u.

To visualize the charge transfer more clearly, another intuitive representation of charge density difference has been presented.[14] Two centroids of charges (C_+ and C_-) associated with the positive and negative density regions are defined. The S index represents the overlap integral of hole-electron distribution, evaluated using the following equation: $S_{index} = \int (\rho^{hole}(r)\rho^{ele}(r))^{1/2}\, dr$[15] Where ρ^{hole} and ρ^{ele} stand for the density distribution of hole and electron, respectively. The D_{index} reflects the distance between the centroid of hole and electron, measured using the following equation: $D_x = |X_{ele} - X_{hole}|$[16] (1), $D_y = |Y_{ele} - Y_{hole}|$ (2), $D_z = |Z_{ele} - Z_{hole}|$ (3), $D_{index} = ((D_x)^2 + (D_y)^2 + (D_z)^2)^{1/2}$ (4), Where X_{hole} corresponds to the X coordinate of the centroid of electron obtained

through multiplying the ρ^{hole} function by the X coordinate variable and integrating in the whole space.

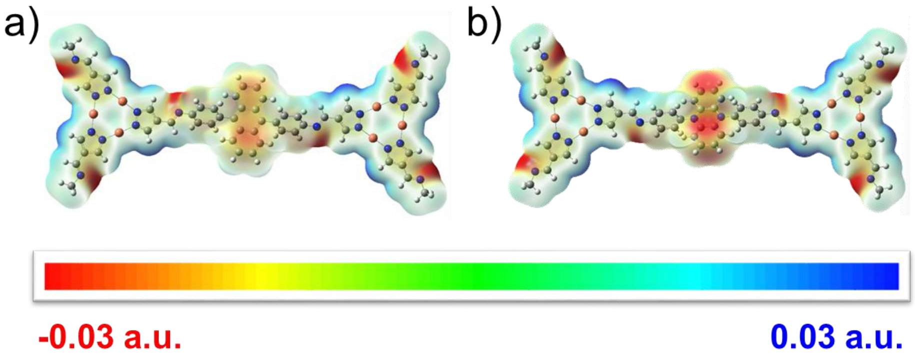

Figure S41. The electrostatic potential (ESP) of (a) **JNM-36** and (b) **JNM-37** was mapped on the van der Waals surfaces (isovalue = 0.0004 a.u.).

.

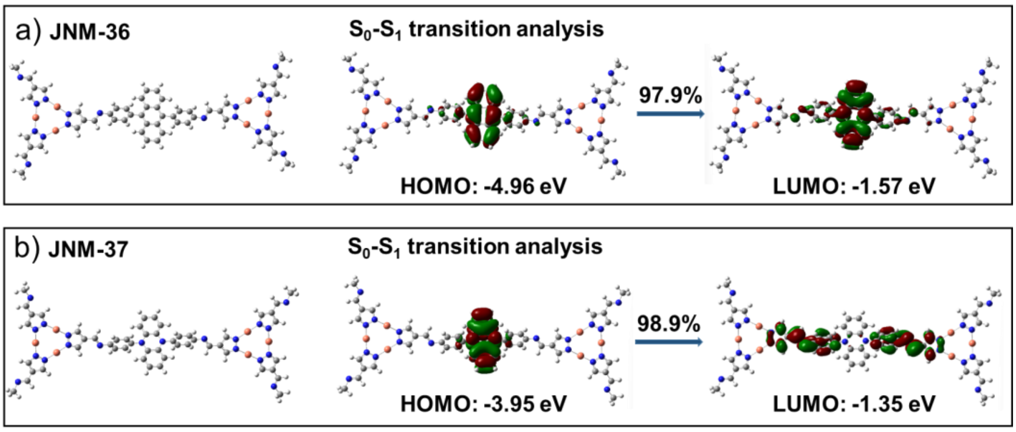

Figure S42. The optimized geometries, calculated frontier molecular orbitals, and molecular orbital contribution of the S_0-S_1 transition analysis of (a) **JNM-36** and (b) **JNM-37** (isovalue = 0.02 a.u.).

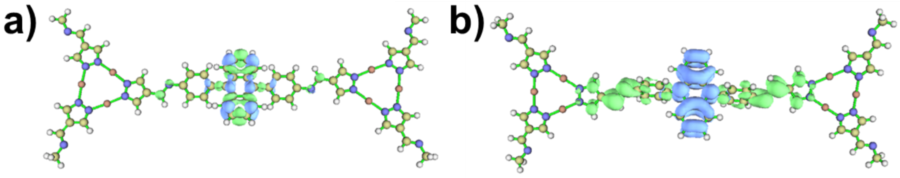

Figure S43. The electron density differences (EDD) maps of S_1 state for (a) **JNM-36** and (b) **JNM-37** (isovalue = 0.001 a.u.).

Table S4. Calculated S_r indexes, D indexes, and S_r/D values of the $S_0 \rightarrow S_1$ transitions of (a) **JNM-36** and (b) **JNM-37**.

	S_r (a.u.)	D (Å)	S_r/D
**JNM-36**	**0.869**	**0.016**	**54.312**
**JNM-37**	**0.112**	**0.421**	**0.266**

17. ^{1}H and ^{13}C NMR spectra for products

N-(4,4,4-trifluoro-2-hydroxy-2-phenylbutyl)benzamide (**5a**)

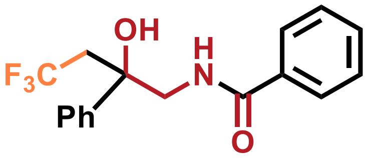

Yield: 81% (104.6 mg). **^{1}H NMR** (400 MHz, $CDCl_3$, 298 K) δ = 2.69-2.87 (2H, m), 3.61 (1H, dd, *J* = 14.4 Hz, *J* = 5.2 Hz), 4.18 (1H, dd, *J* = 14.4 Hz, *J* = 7.2 Hz), 4.22 (1H, s), 6.39 (1H, s), 7.28-7.41 (5H, m), 7.48-7.51 (3H, m), 7.61-7.63 (2H, m) [ppm]; **^{13}C NMR** (100 MHz, $CDCl_3$, 298 K) δ = 29.84, 43.51 (q, *J* = 26 Hz), 50.75, 75.26 (d, *J* = 1.3 Hz), 125.19, 125.91 (d, *J* = 277.1 Hz), 127.08, 127.85, 128.75(d, *J* = 7.9 Hz), 132.11, 133.64, 142.36, 169.57 [ppm]; **^{19}F NMR** (376 MHz, $CDCl_3$, 298 K). δ = -58.97[ppm]. **QTOF-HRMS**: m/z calcd for $[M+Na]^+$ $C_{17}H_{16}NaF_3NO_2^+$, 346.1033; found: 346.1025.

Table S5. Crystal data and structure refinement parameters for **5a**

Parameter	**5a**
CCDC Number	2380620
Chemical formula	$C_{17}H_{16}F_3NO_2$
Formula weight	323.31
Crystal system	monoclinic
Space group	$P2_1/n$
a (Å)	5.6641(1)
b (Å)	12.4927(2)
c (Å)	23.3950(4)
α (deg)	90.00
β (deg)	93.845(2)
γ (deg)	90.00
V (Å^3)	1651.70(5)
Z	4
ρ_{calcd}(g cm^{-3})	1.300
μ (mm^{-1})	0.924
Reflections collected	8289
Unique reflections	3361
R_{int}	0.0448
Goodness-of-fit on F^2	1.067
R_1[a] [I > 2σ(I)]	0.0521
wR_2[b] [I > 2σ(I)]	0.1275
R_1[a] [all refl.]	0.0622
wR_2[b] [all refl.]	0.1340

[a] $R_1=\sum(||F_0|-|F_c||)/\sum|F_0|$; [b] $wR_2=[\sum w(F_0^2-F_c^2)^2/\sum w(F_0^2)^2]$

4-methyl-*N*-(4,4,4-trifluoro-2-hydroxy-2-phenylbutyl)benzamide (**5b**)

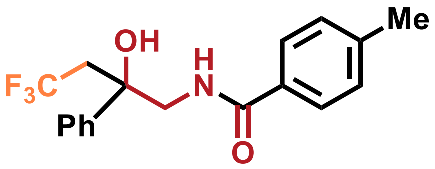

Yield: 87% (117.3 mg). **^{1}H NMR** (400 MHz, $CDCl_3$, 298 K) δ = 2.34 (3H, s), 2.74 (2H, q, *J* = 2.7 Hz), 3.63 (1H, dd, *J* = 14.4 Hz, *J* = 5.2 Hz), 4.07 (1H, dd, *J* = 14.4 Hz, *J* = 6.8 Hz), 4.80 (1H, s), 6.85 (1H, s), 7.11-7.13 (2H, m), 7.27-7.37 (3H, m), 7.46-7.51 (4H, m) [ppm]; **^{13}C NMR** (100 MHz, $CDCl_3$, 298 K) δ = 21.48, 29.79, 43.35 (q, *J* = 26 Hz), 50.79, 75.07 (d, *J* = 2 Hz), 125.23, 125.88 (d, *J* = 276.8 Hz), 127.13, 127.66 , 128.53, 129.53, 130.69, 142.44, 142.59, 169.72 [ppm]; **^{19}F NMR** (376 MHz, $CDCl_3$, 298 K). δ = -58.94 [ppm]. **QTOF-HRMS**: m/z calcd for $[M+Na]^+$ $C_{18}H_{18}NaF_3NO_2^+$, 360.1190; found: 360.1182.

4-methoxy-*N*-(4,4,4-trifluoro-2-hydroxy-2-phenylbutyl)benzamide (**5c**)

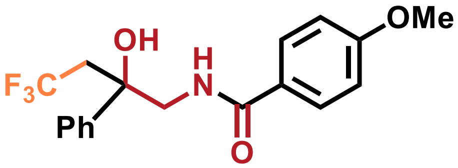

Yield: 90% (127.1 mg). **^{1}H NMR** (400 MHz, $CDCl_3$, 298 K) δ = 2.68-2.81 (2H, m), 3.59 (1H, dd, *J* = 14.4 Hz, *J* = 5.6 Hz), 3.81 (3H, s), 4.12 (1H, dd, *J* = 14.4 Hz, *J* = 6.8 Hz), 4.58 (1H, s), 6.49 (1H, s), 6.84 (2H, d, *J* = 8.4 Hz), 7.28-7.30 (1H, m), 7.36 (2H, t, *J* = 7.2 Hz), 7.46-7.48 (2H, m), 7.58 (2H, d, *J* = 8.4 Hz) [ppm]; **^{13}C NMR** (100 MHz, $CDCl_3$, 298 K) δ = 29.84, 43.48 (q, *J* = 26 Hz), 50.84, 55.53, 75.24(d, *J* = 1.9 Hz), 113.95, 125.24, 125.72, 125.93 (d, *J* = 276.1 Hz), 127.74, 128.64, 129.02, 142.54, 162.69, 169.20 [ppm]; **^{19}F NMR** (376 MHz, $CDCl_3$, 298 K). δ = -58.95 [ppm]. **QTOF-HRMS**: m/z calcd for $[M+Na]^+$ $C_{18}H_{18}NaF_3NO_3^+$, 376.1139; found: 376.1131.

4-(tert-butyl)-*N*-(4,4,4-trifluoro-2-hydroxy-2-phenylbutyl)benzamide (**5d**)

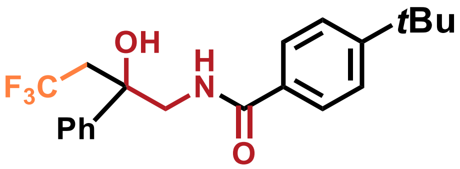

Yield: 78% (118.2 mg). **^{1}H NMR** (400 MHz, $CDCl_3$, 298 K) δ = 1.31 (9H, s), 2.71-2.83 (2H, m), 3.60 (1H, dd, *J* = 14.4 Hz, *J* = 5.2 Hz), 4.17 (1H, dd, *J* = 14.4 Hz, *J* = 7.2 Hz), 4.37 (1H, s), 6.40 (1H, s), 7.29-7.32 (1H, m), 7.36-7.42 (4H, m), 7.47-7.50 (2H, m), 7.55-7.58 (2H, m) [ppm]; **^{13}C NMR** (100 MHz, $CDCl_3$, 298 K) δ = 29.82, 31.22, 35.06, 43.50 (q, *J* = 26 Hz), 50.77, 75.22(d, *J* = 2 Hz), 125.24, 125.71, 125.92(d, *J* = 276.7 Hz), 127.01, 127.46, 128.64, 130.69, 142.49, 155.72, 169.55 [ppm]; **^{19}F NMR** (376 MHz, $CDCl_3$, 298 K). δ = -58.97[ppm]. **QTOF-HRMS**: m/z calcd for $[M+Na]^+$ $C_{21}H_{24}NaF_3NO_2^+$, 402.1659; found: 402.1651.

4-fluoro-*N*-(4,4,4-trifluoro-2-hydroxy-2-phenylbutyl)benzamide (**5e**)

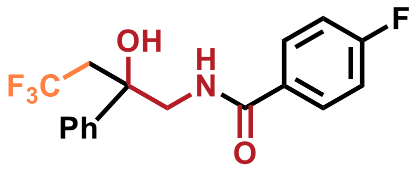

Yield: 73% (99.6 mg). **^{1}H NMR** (400 MHz, $CDCl_3$, 298 K) δ = 2.71-2.84 (2H, m), 3.62 (1H, dd, *J* = 14.4 Hz, *J* = 5.2 Hz), 4.14 (1H, dd, *J* = 14.4 Hz, *J* = 7.2 Hz), 6.43 (1H, s), 7.02-7.08 (2H, m), 7.28-7.48 (5H, m), 7.60-7.63 (2H, m) [ppm]; **^{13}C NMR** (100 MHz, $CDCl_3$, 298 K) δ = 29.72, 43.39 (q, *J* = 26 Hz), 50.56, 75.06(d, *J* = 2.4 Hz), 115.72(d, *J* = 21.8 Hz), 125.05, 127.15, 127.78, 128.61, 129.36 (d, *J* = 9.1 Hz), 129.71 (d, *J* = 3 Hz), 142.13, 164.95 (d, *J* = 251.2 Hz), 168.28 [ppm]; **^{19}F NMR** (376 MHz, $CDCl_3$, 298 K). δ = -58.97, 107.20 [ppm]. **QTOF-HRMS**: m/z calcd for $[M+H]^+$ $C_{17}H_{16}F_4NO_2^+$, 342.1119; found: 342.1113.

4-chloro-*N*-(4,4,4-trifluoro-2-hydroxy-2-phenylbutyl)benzamide (**5f**)

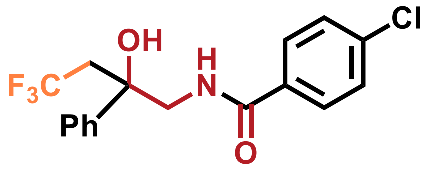

Yield: 79% (112.8 mg). **^{1}H NMR** (400 MHz, $CDCl_3$, 298 K) δ = 2.71-2.81 (2H, m), 3.65 (1H, dd, *J* = 14.4 Hz, *J* = 5.2 Hz), 4.08 (1H, dd, *J* = 14.4 Hz, *J* = 6.8 Hz), 4.39 (1H, s), 6.67 (1H, s), 7.27-7.31 (3H, m), 7.37 (2H, t, *J* = 7.2 Hz), 7.45-7.49 (4H, m) [ppm]; **^{13}C NMR** (100 MHz, $CDCl_3$, 298 K) δ = 29.82, 43.49 (q, *J* = 26 Hz), 50.63, 75.05(d, *J* = 2.1 Hz), 125.15, 125.84 (d, *J* = 276.9 Hz), 127.89, 128.49, 128.68,

128.95, 132.00, 138.32, 142.16, 169.43 [ppm]; ^{19}**F NMR** (376 MHz, $CDCl_3$, 298 K). δ = -58.96[ppm]. **QTOF-HRMS**: m/z calcd for $[M+H]^+$ $C_{17}H_{16}ClF_3NO_2^+$, 358.0823; found: 358.0816.

4-bromo-*N*-(4,4,4-trifluoro-2-hydroxy-2-phenylbutyl)benzamide (**5g**)

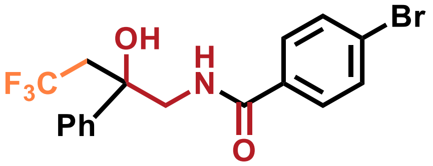

Yield: 76% (121.9 mg). ^{1}**H NMR** (400 MHz, $CDCl_3$, 298 K) δ = 2.72-2.85 (2H, m), 3.62 (1H, dd, *J* = 14.4 Hz, *J* = 5.2 Hz), 4.04 (1H, s), 4.15 (1H, dd, *J* = 14.4 Hz, *J* = 6.8 Hz), 6.36 (1H, s), 7.28-7.32 (1H, m), 7.39 (2H, t, *J* = 7.6 Hz), 7.46-7.53 (6H, m) [ppm]; ^{13}**C NMR** (100 MHz, $CDCl_3$, 298 K) δ = 29.84, 43.52 (q, *J* = 26.2 Hz), 50.64, 75.18 (d, *J* = 2.3 Hz), 125.14, 126.06 (d, *J* = 276.8 Hz), 126.83, 127.94, 128.72 (d, *J* = 8.6 Hz), 132.01, 132.51, 142.17, 168.43 [ppm]; ^{19}**F NMR** (376 MHz, $CDCl_3$, 298 K). δ = -58.96[ppm]. **QTOF-HRMS**: m/z calcd for $[M+H]^+$ $C_{17}H_{15b}rF_3NO_2^+$, 402.0318; found: 402.0311.

N-(4,4,4-trifluoro-2-hydroxy-2-phenylbutyl)-4-(trifluoromethyl)benzamide (**5h**)

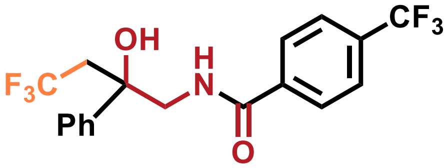

Yield: 75% (117.3 mg). ^{1}**H NMR** (400 MHz, $CDCl_3$, 298 K) δ = 2.72-2.89 (2H, m), 3.67 (1H, dd, *J* = 14.4 Hz, *J* = 5.2 Hz), 3.93 (1H, s), 4.15 (1H, dd, *J* = 14.4 Hz, *J* = 6.8 Hz), 6.47 (1H, s), 7.29-7.49 (5H, m), 7.63-7.71 (4H, m) [ppm]; ^{13}**C NMR** (100 MHz, $CDCl_3$, 298 K) δ = 29.72, 43.43 (q, *J* = 26 Hz), 50.46, 75.05 (q, *J* = 1.8 Hz), 124.98, 125.71 (q, *J* = 3.9 Hz), 127.42, 127.92, 128.70, 133.44, 136.94, 141.88, 167.84 [ppm]; ^{19}**F NMR** (376 MHz, $CDCl_3$, 298 K). δ = -58.98, -63.08 [ppm]. **QTOF-HRMS**: m/z calcd for $[M+H]^+$ $C_{18}H_{16}F_6NO_2^+$, 392.1087; found: 392.1080.

4-cyano-*N*-(4,4,4-trifluoro-2-hydroxy-2-phenylbutyl)benzamide (**5i**)

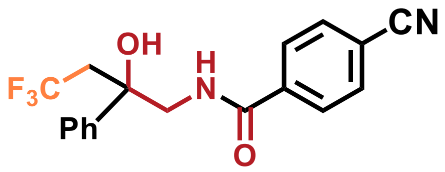

Yield: 67% (93.2 mg). **^{1}H NMR** (400 MHz, $CDCl_3$, 298 K) δ = 2.72-2.85 (2H, m), 3.69 (1H, dd, *J* = 14.4 Hz, *J* = 5.2 Hz), 4.10 (1H, dd, *J* = 14.4 Hz, *J* = 5.6 Hz), 6.62 (1H, s), 7.28-7.48 (5H, m), 7.63-7.69 (4H, m) [ppm]; **^{13}C NMR** (100 MHz, $CDCl_3$, 298 K) δ = 29.71, 43.42 (q, *J* = 26 Hz), 50.42, 75.94 (d, *J* = 2.1 Hz), 115.35, 117.85, 124.99, 125.69 (d, *J* = 277 Hz), 127.68, 127.96, 128.69, 132.46, 137.64, 141.79, 167.29 [ppm]; **^{19}F NMR** (376 MHz, $CDCl_3$, 298 K). δ = -58.96 [ppm]. **QTOF-HRMS**: m/z calcd for $[M+H]^+$ $C_{18}H_{16}F_3N_2O_2^+$, 349.1166; found: 349.1158.

N-(4,4,4-trifluoro-2-hydroxy-2-phenylbutyl)picolinamide (**5j**)

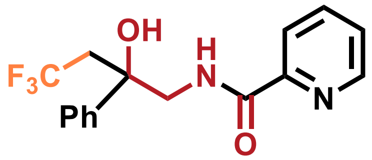

Yield: 71% (92.1 mg). **^{1}H NMR** (400 MHz, $CDCl_3$, 298 K) δ = 2.74-2.85 (2H, m), 3.67 (1H, dd, *J* = 14.4 Hz, *J* = 5.2 Hz), 4.12 (1H, dd, *J* = 14.4 Hz, *J* = 7.2 Hz), 4.42 (1H, s), 6.39 (1H, s), 7.28-7.42 (4H, m), 7.50-7.53 (2H, m), 7.79-7.84 (1H, m), 7.12-7.15 (1H, m), 8.35-8.38 (1H, m), 8.46-8.47 (1H, m) [ppm]; **^{13}C NMR** (100 MHz, $CDCl_3$, 298 K) δ = 43.31 (q, *J* = 26 Hz), 50.56, 75.19 (d, *J* = 1.8 Hz), 122.63, 125.27, 125.93 (d, *J* = 276.8 Hz), 126.68, 127.72, 128.58, 137.56, 142.48, 148.34, 149.04, 166.42 [ppm]; **^{19}F NMR** (376 MHz, $CDCl_3$, 298 K). δ = -59.00 [ppm]. **QTOF-HRMS**: m/z calcd for $[M+Na]^+$ $C_{16}H_{15}NaF_3N_2O_2^+$, 347.0986; found: 347.0978.

N-(4,4,4-trifluoro-2-hydroxy-2-phenylbutyl)thiophene-2-carboxamide (**5k**)

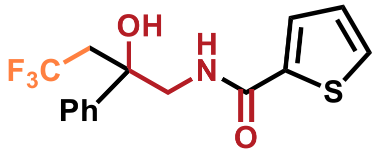

Yield: 69% (90.8 mg). **^{1}H NMR** (400 MHz, $CDCl_3$, 298 K) δ = 2.73-2.84 (2H, m), 3.60 (1H, dd, *J* = 14.4 Hz, *J* = 5.2 Hz), 4.07 (1H, dd, *J* = 14.4 Hz, *J* = 7.2 Hz), 4.46 (1H, s), 6.76 (1H, s), 6.99 (1H, t, *J* = 4.4 Hz), 7.28-7.29 (1H, m), 7.36 (2H, t, *J* = 8.4

Hz), 7.41-7.49 (4H, m) [ppm]; ^{13}**C NMR** (100 MHz, $CDCl_3$, 298 K) δ = 29.73, 43.21 (q, *J* = 21 Hz), 50.70, 75.94 (d, *J* = 2.2 Hz), 125.20, 125.87 (d, *J* = 276.8 Hz), 127.78, 127.87, 128.60, 128.92, 130.90, 137.78, 142.18, 163.82 [ppm]; ^{19}**F NMR** (376 MHz, $CDCl_3$, 298 K). δ = -58.89 [ppm].

N-(4,4,4-trifluoro-2-hydroxy-2-(p-tolyl)butyl)benzamide (**5l**)

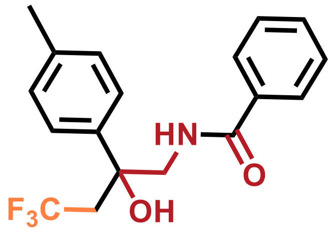

Yield: 84% (113.2 mg). ^{1}**H NMR** (400 MHz, $CDCl_3$, 298 K) δ = 2.34 (3H, s), 2.69-2.85 (2H, m), 3.59 (1H, dd, *J* = 14.4 Hz, *J* = 5.2 Hz), 4.15 (1H, dd, *J* = 14.4 Hz, *J* = 7.2 Hz), 4.18 (1H, s), 6.51 (1H, s), 7.17-7.19 (2H, m), 7.35-7.40 (4H, m), 7.46-7.50 (1H, m), 7.62-7.64 (2H, m) [ppm]; ^{13}**C NMR** (100 MHz, $CDCl_3$, 298 K) δ = 21.01, 29.72, 43.35 (q, *J* = 26 Hz), 50.60, 75.99 (d, *J* = 1.0 Hz), 124.97, 127.01, 128.65, 129.28, 131.93, 133.61, 137.40, 139.22, 169.30 [ppm]; ^{19}**F NMR** (376 MHz, $CDCl_3$, 298 K). δ = -58.95 [ppm]. **QTOF-HRMS**: m/z calcd for $[M+Na]^+$ $C_{18}H_{18}NaF_3NO_2^+$, 360.1190; found: 360.1182.

4-methoxy-*N*-(4,4,4-trifluoro-2-hydroxy-2-(p-tolyl)butyl)benzamide (**5m**)

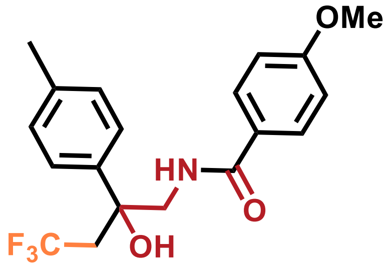

Yield: 89% (130.6 mg). ^{1}**H NMR** (400 MHz, $CDCl_3$, 298 K) δ = 2.34 (3H, s), 2.69-2.78 (2H, m), 3.55 (1H, dd, *J* = 14.4 Hz, *J* = 5.2 Hz), 3.82 (3H, s), 4.13 (1H, dd, *J* = 14.4 Hz, *J* = 7.2 Hz), 4.15 (1H, s), 6.87 (2H, d, *J* = 8.8 Hz), 7.26 (4H, dd, *J* = 70.8 Hz, *J* = 8.4 Hz), 7.60-7.63 (2H, m), 7.61-7.63 (2H, m) [ppm]; ^{13}**C NMR** (100 MHz, $CDCl_3$, 298 K) δ = 21.01, 29.72, 43.36 (q, *J* = 26 Hz), 50.67, 55.44, 75.05 (d, *J* = 1.5 Hz), 113.84, 114.40, 125.00, 125.70, 128.92, 129.25, 137.31, 139.40, 162.57, 168.90 [ppm]; ^{19}**F NMR** (376 MHz, $CDCl_3$, 298 K). δ = -58.94 [ppm]. **QTOF-HRMS**: m/z

calcd for [M+Na]$^+$ $C_{19}H_{20}NaF_3NO_3^+$, 390.1295; found: 390.1287.

N-(4,4,4-trifluoro-2-hydroxy-2-(naphthalen-2-yl)butyl)benzamide (**5n**)

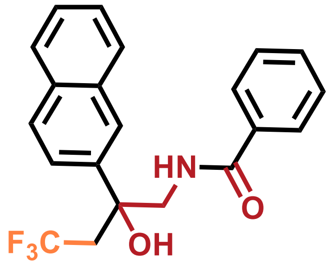

Yield: 74% (110.4 mg). ^{1}**H NMR** (400 MHz, $CDCl_3$, 298 K) δ = 2.80-2.94 (2H, m), 3.69 (1H, dd, *J* = 14.4 Hz, *J* = 5.2 Hz), 4.28 (1H, dd, *J* = 14.4 Hz, *J* = 7.2 Hz), 4.49 (1H, s), 6.46 (1H, s), 7.33-7.37 (2H, m), 7.44-7.61 (6H, m), 7.83-7.87 (3H, m), 8.04 (1H, s) [ppm]; ^{13}**C NMR** (100 MHz, $CDCl_3$, 298 K) δ = 29.72, 43.39 (q, *J* = 26 Hz), 50.64, 75.38 (d, *J* = 1.5 Hz), 122.87, 124.50, 126.42 (d, *J* = 11.5 Hz), 126.99, 127.59, 128.36 (d, *J* = 11.7 Hz), 128.67, 132.02, 132.65, 133.28 (d, *J* = 19.7 Hz), 139.63, 169.58 [ppm]; ^{19}**F NMR** (376 MHz, $CDCl_3$, 298 K). δ = -58.95 [ppm]. **QTOF-HRMS**: m/z calcd for [M+Na]$^+$ $C_{21}H_{18}NaF_3NO_2^+$, 396.1190; found: 396.1182.

N-(2-(4-chlorophenyl)-4,4,4-trifluoro-2-hydroxybutyl)benzamide (**5o**)

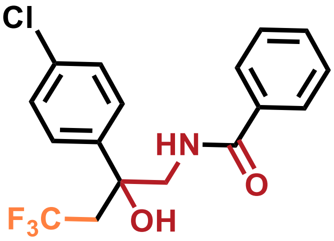

Yield: 81% (115.6 mg). ^{1}**H NMR** (400 MHz, $CDCl_3$, 298 K) δ = 2.69-2.81 (2H, m), 3.60 (1H, dd, *J* = 14.4 Hz, *J* = 5.2 Hz), 4.13 (1H, dd, *J* = 14.4 Hz, *J* = 7.2 Hz), 4.50 (1H, s), 6.44 (1H, s), 7.34-7.53 (7H, m), 7.63-7.66 (2H, m) [ppm]; ^{13}**C NMR** (100 MHz, $CDCl_3$, 298 K) δ = 29.72, 43.39 (q, *J* = 26 Hz), 50.71, 75.01 (d, *J* = 2 Hz), 126.73, 127.00, 128.73, 128.75, 132.18, 133.23, 133.66, 140.91, 169.63 [ppm]; ^{19}**F NMR** (376 MHz, $CDCl_3$, 298 K). δ = -58.96 [ppm]. **QTOF-HRMS**: m/z calcd for [M+Na]$^+$ $C_{17}H_{15}NaClF_3NO_2^+$, 376.0643; found: 376.0636.

4-chloro-*N*-(2-(4-chlorophenyl)-4,4,4-trifluoro-2-hydroxybutyl)benzamide (**5p**)

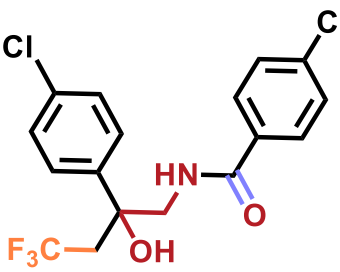

Yield: 68% (106.4 mg). **^{1}H NMR** (400 MHz, $CDCl_3$, 298 K) δ = 2.68-2.79 (2H, m), 3.61 (1H, dd, *J* = 14.4 Hz, *J* = 5.2 Hz), 4.09 (1H, dd, *J* = 14.4 Hz, *J* = 7.2 Hz), 4.38 (1H, s), 6.49 (1H, s), 7.33-7.42 (6H, m), 7.55-7.57 (2H, m) [ppm]; **^{13}C NMR** (100 MHz, $CDCl_3$, 298 K) δ = 29.72, 43.38 (q, *J* = 26 Hz), 50.59, 75.90 (d, *J* = 1 Hz), 125.60 (d, *J* = 276.9 Hz), 126.67, 128.42, 128.76, 129.00, 131.62, 133.77, 138.48, 140.68, 168.41 [ppm]; **^{19}F NMR** (376 MHz, $CDCl_3$, 298 K). δ = -58.95 [ppm]. **QTOF-HRMS**: m/z calcd for $[M+Na]^+$ $C_{17}H_{14}NaCl_2F_3NO_2^+$, 414.0254; found: 414.0246.

N-(4,4,4-trifluoro-2-(4-fluorophenyl)-2-hydroxybutyl)benzamide (**5q**)

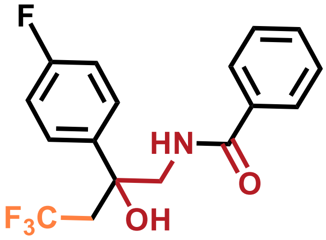

Yield: 79% (107.8 mg). **^{1}H NMR** (400 MHz, $CDCl_3$, 298 K) δ = 2.71-2.79 (2H, m), 3.61 (1H, dd, *J* = 14.4 Hz, *J* = 5.2 Hz), 4.13 (1H, dd, *J* = 14.4 Hz, *J* = 7.2 Hz), 4.42 (1H, s), 6.45 (1H, s), 7.04-7.08 (2H, m), 7.38-7.50 (5H, m), 7.63-7.65 (2H, m) [ppm]; **^{13}C NMR** (100 MHz, $CDCl_3$, 298 K) δ = 29.72, 43.48 (q, *J* = 26 Hz), 50.76, 75.96(d, *J* = 1 Hz), 115.33, 115.55, 126.97, 127.04, 128.74, 132.12, 133.32, 138.03 (d, *J* = 3.3 Hz), 169.53 [ppm]; **^{19}F NMR** (376 MHz, $CDCl_3$, 298 K). δ = -58.99, -114.93 [ppm]. **QTOF-HRMS**: m/z calcd for $[M+Na]^+$ $C_{17}H_{15}NaF_4NO_2^+$, 364.0939; found: 364.0931.

3-methyl-*N*-(4,4,4-trifluoro-2-(4-fluorophenyl)-2-hydroxybutyl)benzamide (**5r**)

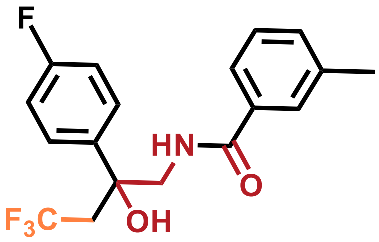

Yield: 73% (103.7 mg). **^{1}H NMR** (400 MHz, $CDCl_3$, 298 K) δ = 2.36 (3H, s), 2.72-2.81 (2H, m), 3.63 (1H, dd, *J* = 14.4 Hz, *J* = 5.2 Hz), 4.12 (1H, dd, *J* = 14.4 Hz, *J* = 7.2 Hz), 4.62 (1H, s), 6.57 (1H, s), 7.05-7.09 (2H, m), 7.28-7.34 (2H, m), 7.40-7.50 (4H, m) [ppm]; **^{13}C NMR** (100 MHz, $CDCl_3$, 298 K) δ = 21.29, 29.72, 43.45 (q, *J* = 26 Hz), 50.81, 75.91 (d, *J* = 2.5 Hz), 115.27, 115.49, 123.89, 124.31, 126.99, 127.07, 127.80, 127.85, 128.57, 132.87, 133.27, 138.07 (d, *J* = 4.8 Hz), 138.69, 162.16 (d, *J* = 245 Hz), 169.84 [ppm]; **^{19}F NMR** (376 MHz, $CDCl_3$, 298 K). δ = -59.00, -115.02 [ppm]. **QTOF-HRMS**: m/z calcd for $[M+Na]^+$ $C_{18}H_{17}NaF_4NO_2^+$, 378.1095; found: 378.1088.

N-(4,4,4-trifluoro-2-hydroxy-2-phenylbutyl)benzamide (**5a-re**)

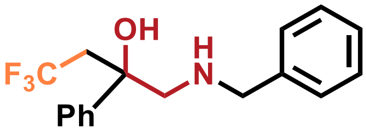

Yield: 95% (104.6 mg). **^{1}H NMR** (400 MHz, $CDCl_3$, 298 K) δ = 2.58-2.68 (2H, m), 3.06 (2H, dd, *J* = 117.2 Hz, *J* = 12 Hz), 3.75 (2H, s), 7.22-7.49 (10H, m) [ppm]; **^{13}C NMR** (100 MHz, $CDCl_3$, 298 K) δ = 29.77, 44.04 (q, *J* = 26 Hz), 53.81, 57.88, 72.28 (q, *J* = 4 Hz), 125.30, 125.70 (d, *J* = 277.1 Hz), 127.38 (d, *J* = 10 Hz), 128.01, 128.55(d, *J* = 7 Hz), 139.58, 143.57 [ppm]; **^{19}F NMR** (376 MHz, $CDCl_3$, 298 K). δ = -59.43[ppm]. **QTOF-HRMS**: m/z calcd for $[M+H]^+$ $C_{17}H_{16}NaF_3NO_2^+$, 310.1420; found: 310.1413.

3-benzyl-5-phenyl-5-(2,2,2-trifluoroethyl)oxazolidin-2-one (**6a**)

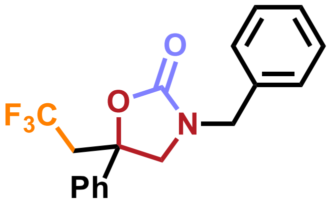

Yield: 85% (113.9 mg). **^{1}H NMR** (400 MHz, $CDCl_3$, 298 K) δ = 2.74-2.84 (2H, m), 3.68 (2H, dd, *J* = 61.9 Hz, *J* = 9.2 Hz), 4.43 (2H, q, *J* = 14.4 Hz), 7.19-7.21 (2H, m), 7.29-7.39 (8H, m) [ppm]; **^{13}C NMR** (100 MHz, $CDCl_3$, 298 K) δ = 44.42 (q, *J* = 27.5 Hz), 48.33, 55.17, 78.18(q, *J* = 1.6 Hz), 123.12, 124.35, 125.89, 128.14, 128.24, 128.74, 128.97, 128.99, 135.10, 140.94, 156.46 [ppm]; **^{19}F NMR** (376 MHz, $CDCl_3$, 298 K). δ = -60.54[ppm].

3-(4-methylbenzyl)-5-phenyl-5-(2,2,2-trifluoroethyl)oxazolidin-2-one (**6b**)

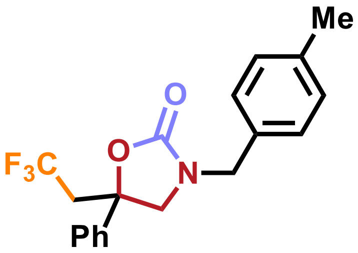

Yield: 88% (112.8 mg). **^{1}H NMR** (400 MHz, $CDCl_3$, 298 K) δ = 2.35 (3H, s), 2.79-2.87 (2H, m), 3.70 (2H, dd, *J* = 64.4 Hz, *J* = 8.4 Hz), 4.42 (2H, q, *J* = 14.4 Hz), 7.11-7.16 (4H, m), 7.35-7.44 (5H, m) [ppm]; **^{13}C NMR** (100 MHz, $CDCl_3$, 298 K) δ = 21.11, 44.28 (q, *J* = 27.5 Hz), 47.97, 55.24, 78.09(q, *J* = 2.4 Hz), 123.16, 124.30, 125.93, 128.08, 128.62, 128.89, 129.59, 132.03, 137.92, 140.97, 156.41 [ppm]; **^{19}F NMR** (376 MHz, $CDCl_3$, 298 K). δ = -60.44[ppm].

3-(4-methoxybenzyl)-5-phenyl-5-(2,2,2-trifluoroethyl)oxazolidin-2-one (**6c**)

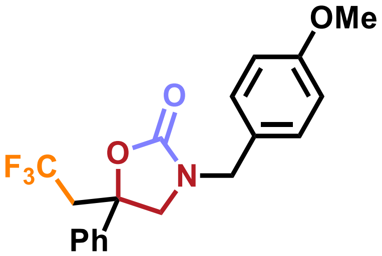

Yield: 83% (121.1 mg). **^{1}H NMR** (400 MHz, $CDCl_3$, 298 K) δ = 2.75-2.83 (2H, m), 3.65 (2H, dd, *J* = 65.6 Hz, *J* = 9.2 Hz), 3.76 (3H, s), 4.36 (2H, q, *J* = 14.8 Hz),

6.82-6.84 (2H, m), 7.11-7.13 (2H, m), 7.31-7.45 (5H, m) [ppm]; ^{13}**C NMR** (100 MHz, $CDCl_3$, 298 K) δ = 44.28 (q, *J* = 27.7 Hz), 47.65, 55.10, 55.26, 78.05(q, *J* = 2.4 Hz), 114.25, 123.13, 124.27, 125.89, 127.07, 128.61, 128.88, 129.48, 140.96, 156.32, 156.47 [ppm]; ^{19}**F NMR** (376 MHz, $CDCl_3$, 298 K). δ = -60.49[ppm].

3-(4-(tert-butyl)benzyl)-5-phenyl-5-(2,2,2-trifluoroethyl)oxazolidin-2-one (**6d**)

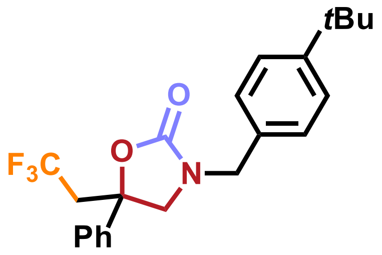

Yield: 86% (134.5 mg). ^{1}**H NMR** (400 MHz, $CDCl_3$, 298 K) δ = δ = 1.32 (9H, s), 2.77-2.88 (2H, m), 3.71 (2H, dd, *J* = 61.6 Hz, *J* = 9.2 Hz), 4.42 (2H, q, *J* = 14.8 Hz), 7.14-7.16 (2H, m), 7.33-7.40 (7H, m) [ppm]; ^{13}**C NMR** (100 MHz, $CDCl_3$, 298 K) δ = 31.33, 34.59, 44.38 (q, *J* = 27.4 Hz), 47.93, 55.33, 78.13(q, *J* = 2.3 Hz), 123.15, 124.36, 125.84, 125.92, 127.84, 128.64, 128.90, 132.42, 140.96, 151.18, 156.42 [ppm]; ^{19}**F NMR** (376 MHz, $CDCl_3$, 298 K). δ = -60.45[ppm].

3-(4-fluorobenzyl)-5-phenyl-5-(2,2,2-trifluoroethyl)oxazolidin-2-one (**6e**)

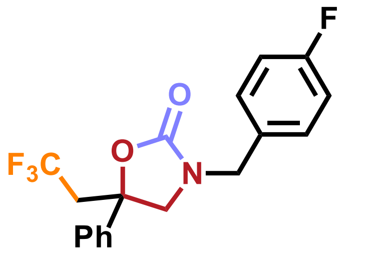

Yield: 80% (112.9 mg). ^{1}**H NMR** (400 MHz, $CDCl_3$, 298 K) δ = 2.71-2.89 (2H, m), 3.68 (2H, dd, *J* = 68 Hz, *J* = 9.2 Hz), 4.40 (2H, q, *J* = 15.2 Hz), 6.97-7.19 (5H, m), 7.34-7.39 (4H, m) [ppm]; ^{13}**C NMR** (100 MHz, $CDCl_3$, 298 K) δ = 44.36 (q, *J* = 27 Hz), 47.56, 54.91, 54.92, 55.17, 78.12(q, *J* = 2.3 Hz), 115.74, 115.95, 123.04, 124.23, 125.81, 128.74, 128.94, 129.84 (d, *J* = 8.1 Hz), 130.90 (d, *J* = 3.1 Hz), 140.90, 156.28, 162.55 (d, *J* = 245.4 Hz) [ppm]; ^{19}**F NMR** (376 MHz, $CDCl_3$, 298 K). δ = -60.59, -113.82 [ppm].

3-(4-chlorobenzyl)-5-phenyl-5-(2,2,2-trifluoroethyl)oxazolidin-2-one (**6f**)

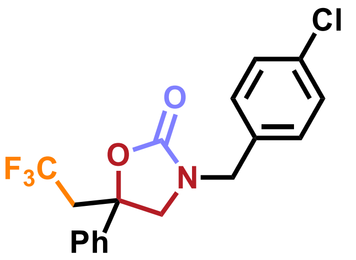

Yield: 71% (104.7 mg). **^{1}H NMR** (400 MHz, $CDCl_3$, 298 K) δ = 2.76-2.88 (2H, m), 3.71 (2H, dd, *J* = 67.6 Hz, *J* = 8.8 Hz), 4.43 (2H, q, *J* = 15.2 Hz), 7.14-7.16 (2H, m), 7.28-7.43 (7H, m) [ppm]; **^{13}C NMR** (100 MHz, $CDCl_3$, 298 K) δ = 44.38 (q, *J* = 27.7 Hz), 47.62, 54.96, 78.17(q, *J* = 2.4 Hz), 123.02, 124.22, 125.79, 128.77, 128.96, 129.11, 129.42, 133.59, 134.10, 140.84, 156.31 [ppm]; **^{19}F NMR** (376 MHz, $CDCl_3$, 298 K). δ = -60.58[ppm].

3-(4-bromobenzyl)-5-phenyl-5-(2,2,2-trifluoroethyl)oxazolidin-2-one (**6g**)

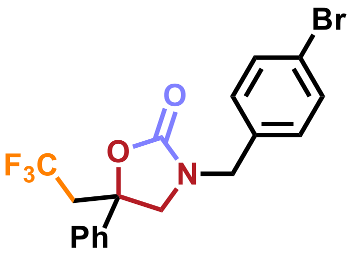

Yield: 70% (115.6 mg). **^{1}H NMR** (400 MHz, $CDCl_3$, 298 K) δ = 2.76-2.85 (2H, m), 3.69 (2H, dd, *J* = 71.6 Hz, *J* = 9.2 Hz), 4.37 (2H, q, *J* = 15.2 Hz), 7.05-7.08 (2H, m), 7.34-7.43 (7H, m) [ppm]; **^{13}C NMR** (100 MHz, $CDCl_3$, 298 K) δ = 44.27 (q, *J* = 27.5 Hz), 47.63, 55.11, 78.19(q, *J* = 1.9 Hz), 122.15, 124.21, 125.85, 128.76, 128.97, 129.74, 131.39(d, *J* = 10 Hz), 132.08, 134.18, 140.86, 156.35 [ppm]; **^{19}F NMR** (376 MHz, $CDCl_3$, 298 K). δ = -60.36[ppm].

5-phenyl-5-(2,2,2-trifluoroethyl)-3-(4-(trifluoromethyl)benzyl)oxazolidin-2-one (**6h**)

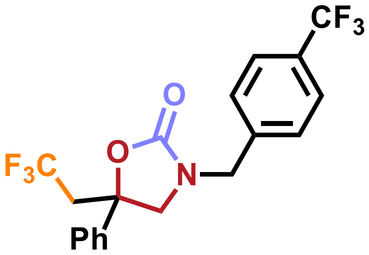

Yield: 66% (106.4 mg). **^{1}H NMR** (400 MHz, $CDCl_3$, 298 K) δ = 2.78-2.90 (2H, m), 3.74 (2H, dd, *J* = 70.4 Hz, *J* = 9.2 Hz), 4.52 (2H, q, *J* = 15.6 Hz), 7.06-7.12 (1H, m),

7.28-7.44 (6H, m), 7.51-7.61 (2H, m) [ppm]; ^{13}**C NMR** (100 MHz, $CDCl_3$, 298 K) δ = 44.37 (q, *J* = 27.7 Hz), 47.84, 55.08, 78.55(q, *J* = 1.4 Hz), 124.20, 125.90(d, *J* = 3.7 Hz) 128.25, 128.84, 128.97, 129.00, 139.16, 140.81, 156.37 [ppm]; ^{19}**F NMR** (376 MHz, $CDCl_3$, 298 K). δ = -60.54, -62.66[ppm].

4-((2-oxo-5-phenyl-5-(2,2,2-trifluoroethyl)oxazolidin-3-yl)methyl)benzonitrile (**6i**)

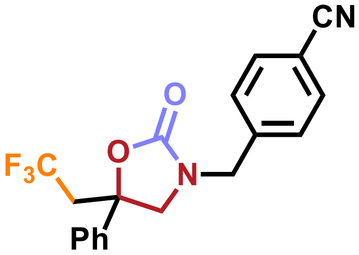

Yield: 75% (108.0 mg). ^{1}**H NMR** (400 MHz, $CDCl_3$, 298 K) δ = 2.75-2.88 (2H, m), 3.73 (2H, dd, *J* = 78.8 Hz, *J* = 9.2 Hz), 4.48 (2H, q, *J* = 15.6 Hz), 7.27-7.29 (2H, m), 7.34-7.41 (5H, m), 7.59-7.61 (2H, m) [ppm]; ^{13}**C NMR** (100 MHz, $CDCl_3$, 298 K) δ = 44.33 (q, *J* = 28 Hz), 47.89, 55.09, 78.33(q, *J* = 1.6 Hz), 112.20, 118.35, 124.15, 125.77, 128.53, 128.91, 129.05, 132.72, 140.57, 140.81, 156.35 [ppm]; ^{19}**F NMR** (376 MHz, $CDCl_3$, 298 K). δ = -60.59[ppm].

5-phenyl-3-(pyridin-2-ylmethyl)-5-(2,2,2-trifluoroethyl)oxazolidin-2-one (**6j**)

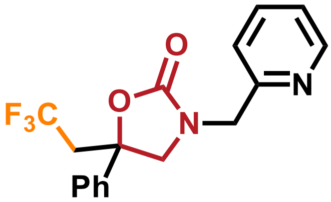

Yield: 68% (91.4 mg). ^{1}**H NMR** (400 MHz, $CDCl_3$, 298 K) δ = 2.85 (2H, q, *J* = 10 Hz), 3.91 (2H, dd, *J* = 78 Hz, *J* = 9.2 Hz), 4.55 (2H, q, *J* = 15.6 Hz), 7.19-7.24 (2H, m), 7.33-7.42 (5H, m), 7.63-7.67 (1H, m), 8.52 (1H, d, *J* = 4.8 Hz) [ppm]; ^{13}**C NMR** (100 MHz, $CDCl_3$, 298 K) δ = 44.50 (q, *J* = 27.7 Hz), 49.63, 55.96 (d, *J* = 2 Hz), 78.49(q, *J* = 2.3 Hz), 122.55, 123.06, 123.17, 124.45, 125.94, 128.74, 128.98, 137.38, 140.92, 149.44, 155.34, 156.59 [ppm]; ^{19}**F NMR** (376 MHz, $CDCl_3$, 298 K). δ = -60.57[ppm].

5-phenyl-3-(thiophen-2-ylmethyl)-5-(2,2,2-trifluoroethyl)oxazolidin-2-one (**6k**)

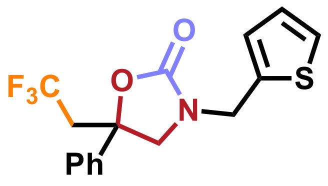

Yield: 73% (99.5 mg). ^{1}**H NMR** (400 MHz, $CDCl_3$, 298 K) δ = 2.83 (2H, q, *J* = 10 Hz), 3.78 (2H, dd, *J* = 61.2 Hz, *J* = 8.8 Hz), 4.64 (2H, q, *J* = 15.6 Hz), 6.96-6.98 (2H, m), 7.27-7.28 (1H, m), 7.37-7.44 (5H, m) [ppm]; ^{13}**C NMR** (100 MHz, $CDCl_3$, 298 K) δ = 42.78, 44.31 (q, *J* = 27.5 Hz), 54.99 (d, *J* = 2 Hz), 78.23(q, *J* = 2.3 Hz), 123.03, 124.31, 125.80, 126.18, 127.20 (d, *J* = 14.5 Hz), 128.69, 128.89, 137.31, 140.61, 155.90 [ppm]; ^{19}**F NMR** (376 MHz, $CDCl_3$, 298 K). δ = -60.51[ppm].

3-benzyl-5-(p-tolyl)-5-(2,2,2-trifluoroethyl)oxazolidin-2-one (**6l**)

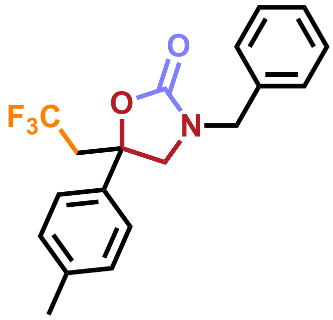

Yield: 90% (125.6 mg). ^{1}**H NMR** (400 MHz, $CDCl_3$, 298 K) δ = 2.35 (3H, s), 2.74-2.82 (2H, m), 3.66 (2H, dd, *J* = 57.6 Hz, *J* = 9.2 Hz), 4.43 (2H, q, *J* = 14.8 Hz), 7.18-7.25 (6H, m), 7.29-7.32 (3H, m) [ppm]; ^{13}**C NMR** (100 MHz, $CDCl_3$, 298 K) δ = 21.06, 44.39 (q, *J* = 27.5 Hz), 48.29, 55.10 (d, *J* = 1.9 Hz), 78.13(q, *J* = 2.5 Hz), 123.09, 124.21, 125.86, 128.09, 128.14, 128.91, 128.97, 129.52, 135.11, 137.92, 138.55, 156.46 [ppm]; ^{19}**F NMR** (376 MHz, $CDCl_3$, 298 K). δ = -60.54[ppm].

3-(4-methoxybenzyl)-5-(p-tolyl)-5-(2,2,2-trifluoroethyl)oxazolidin-2-one (**6m**)

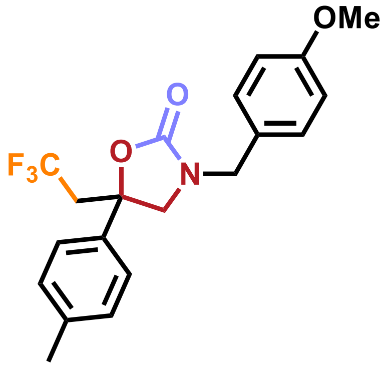

Yield: 93% (141.0 mg). ^{1}**H NMR** (400 MHz, $CDCl_3$, 298 K) δ = δ = 2.34 (3H, s),

2.72-2.80 (2H, m), 3.63 (2H, dd, *J* = 58.8 Hz, *J* = 9.2 Hz), 3.78 (3H, s), 4.36 (2H, q, *J* = 14.4 Hz), 6.82-6.85 (2H, m), 7.12-7.23 (6H, m) [ppm]; **^{13}C NMR** (100 MHz, $CDCl_3$, 298 K) δ = 21.05, 44.38 (q, *J* = 27.5 Hz), 47.70, 54.99, 55.28, 78.07(q, *J* = 2.4 Hz), 114.24, 123.10, 124.21, 125.87, 127.11, 129.50, 137.95, 138.50, 156.38, 159.45 [ppm]; **^{19}F NMR** (376 MHz, $CDCl_3$, 298 K). δ = -60.54[ppm].

3-benzyl-5-(naphthalen-2-yl)-5-(2,2,2-trifluoroethyl)oxazolidin-2-one (**6n**)

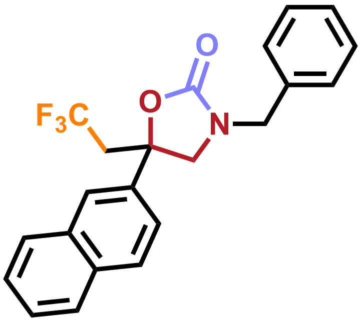

Yield: 86% (132.4 mg). **^{1}H NMR** (400 MHz, $CDCl_3$, 298 K) δ = 2.86-2.96 (2H, m), 3.76 (2H, dd, *J* = 50.4 Hz, *J* = 8.8 Hz), 4.45 (2H, q, *J* = 14.4 Hz), 7.19-7.21 (5H, m), 7.52-7.54 (2H, m), 7.83-8.03 (5H, m) [ppm]; **^{13}C NMR** (100 MHz, $CDCl_3$, 298 K) δ = 44.20 (q, *J* = 16.1 Hz), 48.34, 55.05, 78.24(q, *J* = 2.1 Hz), 121.73, 123.62, 126.93, 127.04, 128.09, 128.18, 128.30, 128.40, 128.94, 129.05, 129.08, 132.88, 132.99, 135.03, 137.91, 156.41 [ppm]; **^{19}F NMR** (376 MHz, $CDCl_3$, 298 K). δ = -60.49[ppm].

3-benzyl-5-(4-chlorophenyl)-5-(2,2,2-trifluoroethyl)oxazolidin-2-one (**6o**)

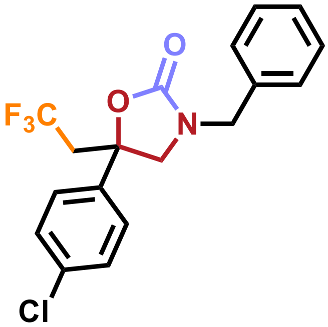

Yield: 88% (129.9 mg). **^{1}H NMR** (400 MHz, $CDCl_3$, 298 K) δ = 2.76-2.84 (2H, m), 3.67 (2H, dd, *J* = 68.4 Hz, *J* = 9.2 Hz), 4.46 (2H, q, *J* = 14.8 Hz), 7.20-7.23 (2H, m), 7.30-7.40 (7H, m) [ppm]; **^{13}C NMR** (100 MHz, $CDCl_3$, 298 K) δ = 44.29 (q, *J* = 27.7 Hz), 48.30, 55.13, 78.73(q, *J* = 1.9 Hz), 122.90, 125.67, 125.90, 128.06, 128.26, 128.97, 129.11, 134.74, 134.90, 139.21, 156.09 [ppm]; **^{19}F NMR** (376 MHz, $CDCl_3$, 298 K). δ = -60.46[ppm].

3-(4-chlorobenzyl)-5-(4-chlorophenyl)-5-(2,2,2-trifluoroethyl)oxazolidin-2-one (**6p**)

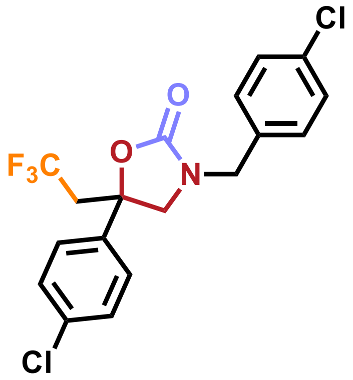

Yield: 74% (119.3 mg). **^{1}H NMR** (400 MHz, $CDCl_3$, 298 K) δ = 2.73-2.82 (2H, m), 3.64 (2H, dd, *J* = 73.6 Hz, *J* = 9.2 Hz), 4.39 (2H, q, *J* = 15.2 Hz), 7.11-7.13 (2H, m), 7.27-7.39 (6H, m) [ppm]; **^{13}C NMR** (100 MHz, $CDCl_3$, 298 K) δ = 44.33 (q, *J* = 27.8 Hz), 47.67, 54.98, 78.78(q, *J* = 2.1 Hz), 125.62, 125.82, 129.18, 129.24, 129.44, 133.42, 134.24, 134.88, 139.14, 156.02 [ppm]; **^{19}F NMR** (376 MHz, $CDCl_3$, 298 K). δ = -60.50[ppm].

3-benzyl-5-(4-fluorophenyl)-5-(2,2,2-trifluoroethyl)oxazolidin-2-one (**6q**)

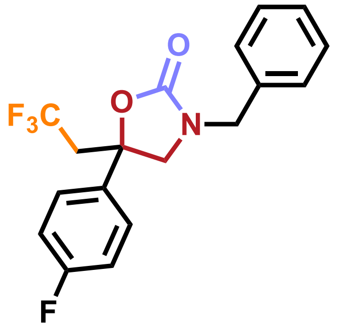

Yield: 82% (115.8 mg). **^{1}H NMR** (400 MHz, $CDCl_3$, 298 K) δ = 2.78 (2H, q, *J* = 10 Hz), 3.65 (2H, dd, *J* = 62.8 Hz, *J* = 9.2 Hz), 4.43 (2H, q, *J* = 15.2 Hz), 7.05-7.09 (2H, m), 7.17-7.20 (2H, m), 7.29-7.35 (5H, m) [ppm]; **^{13}C NMR** (100 MHz, $CDCl_3$, 298 K) δ = 44.45 (q, *J* = 27.6 Hz), 48.29, 55.18, 78.80(q, *J* = 2.3 Hz), 115.77, 115.99, 122.93, 125.70, 126.33, 126.41, 128.06, 128.24, 128.96, 134.95, 136.48, 136.51, 156.16, 162.63 (d, *J* = 246.9 Hz) [ppm]; **^{19}F NMR** (376 MHz, $CDCl_3$, 298 K). δ = -60.53, -112.86 [ppm].

5-(4-fluorophenyl)-3-(3-methylbenzyl)-5-(2,2,2-trifluoroethyl)oxazolidin-2-one (**6r**)

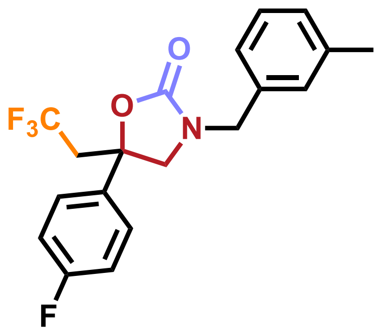

Yield: 86% (126.3 mg). **^{1}H NMR** (400 MHz, $CDCl_3$, 298 K) δ = 2.29 (3H, s), 2.78 (2H, q, J = 10 Hz), 3.65 (2H, dd, J = 63.2 Hz, J = 9.2 Hz), 4.39 (2H, q, J = 14.8 Hz), 6.69-6.98 (2H, m), 7.05-7.11 (3H, m), 7.18-7.22 (1H, m), 7.32-7.36 (2H, m) [ppm]; **^{13}C NMR** (100 MHz, $CDCl_3$, 298 K) δ = 21.30, 44.41 (q, J = 27.5 Hz), 48.17, 55.16, 78.81(q, J = 2.1 Hz), 115.75, 115.97, 122.95, 125.06, 125.71, 126.41 (d, J = 8.3 Hz), 128.78 (t, J = 14.8 Hz), 134.86, 136.52 (d, J = 3.3 Hz), 138.76, 156.17 162.63 (d, J = 246.9 Hz) [ppm]; **^{19}F NMR** (376 MHz, $CDCl_3$, 298 K). δ = -60.52, -112.91[ppm].

3-butyl-5-phenyl-5-(2,2,2-trifluoroethyl)oxazolidin-2-one (**6s**)

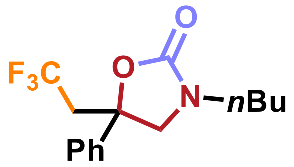

Yield: 90% (108.4 mg). **^{1}H NMR** (400 MHz, $CDCl_3$, 298 K) δ = 0.90 (3H, q, J = 7.2 Hz), 1.22-1.33 (2H, m), 1.43-1.53 (2H, m), 2.78-2.88 (2H, m), 3.17-3.34 (2H, m), 3.84 (2H, dd, J = 80.4 Hz, J = 9.2 Hz), 7.33-7.43 (5H, m) [ppm]; **^{13}C NMR** (100 MHz, $CDCl_3$, 298 K) δ = 13.75, 19.86, 29.35, 43.97, 44.59 (q, J = 27.5 Hz), 55.70 (d, J = 1.9 Hz), 78.87(q, J = 2.4 Hz), 123.26, 124.38, 126.02, 128.74, 129.02, 141.32, 156.36 [ppm]; **^{19}F NMR** (376 MHz, $CDCl_3$, 298 K). δ = -60.59[ppm].

3-(((1R,4R)-bicyclo[2.2.1]hept-5-en-2-yl)methyl)-5-phenyl-5-(2,2,2-trifluoroethyl)oxazolidin-2-one (**6t**)

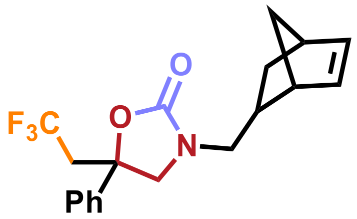

Yield: 51% (71.6 mg). **^{1}H NMR** (400 MHz, $CDCl_3$, 298 K) δ = 1.37-1.45 (1H, m),

1.78-1.84 (1H, m), 2.23-2.32 (1H, m), 2.60 (1H, s), 2.79-2.81 (1H, m), 2.89-3.06 (3H, m), 3.16-3.39 (1H, m), 3.87 (2H, dd, *J* = 71.6Hz, *J* = 8.8 Hz), 5.94-5.96 (1H, m), 6.16-6.18 (1H, m), 7.35-7.44 (5H, m) [ppm]; ^{13}**C NMR** (100 MHz, $CDCl_3$, 298 K) δ = 29.71, 30.01, 37.23, 42.34, 44.34 (t, *J* = 6.4 Hz), 44.54, 48.23, 48.30, 49.53, 56.13, 78.81(q, *J* = 2.2 Hz), 123.15, 124.26, 126.38(d, *J* = 92.5 Hz), 128.65, 128.92, 132.02, 132.09, 136.06, 137.04(d, *J* = 8.3 Hz), 137.84, 141.26, 156.12 [ppm]; ^{19}**F NMR** (376 MHz, $CDCl_3$, 298 K). δ = -60.54[ppm]. **QTOF-HRMS**: m/z calcd for $[M+H]^+$ $C_{19}H_{21}F_3NO_2^+$, 352.1526; found: 352.1519.

^{1}H NMR (400 MHz, $CDCl_3$) spectrum of **5a**

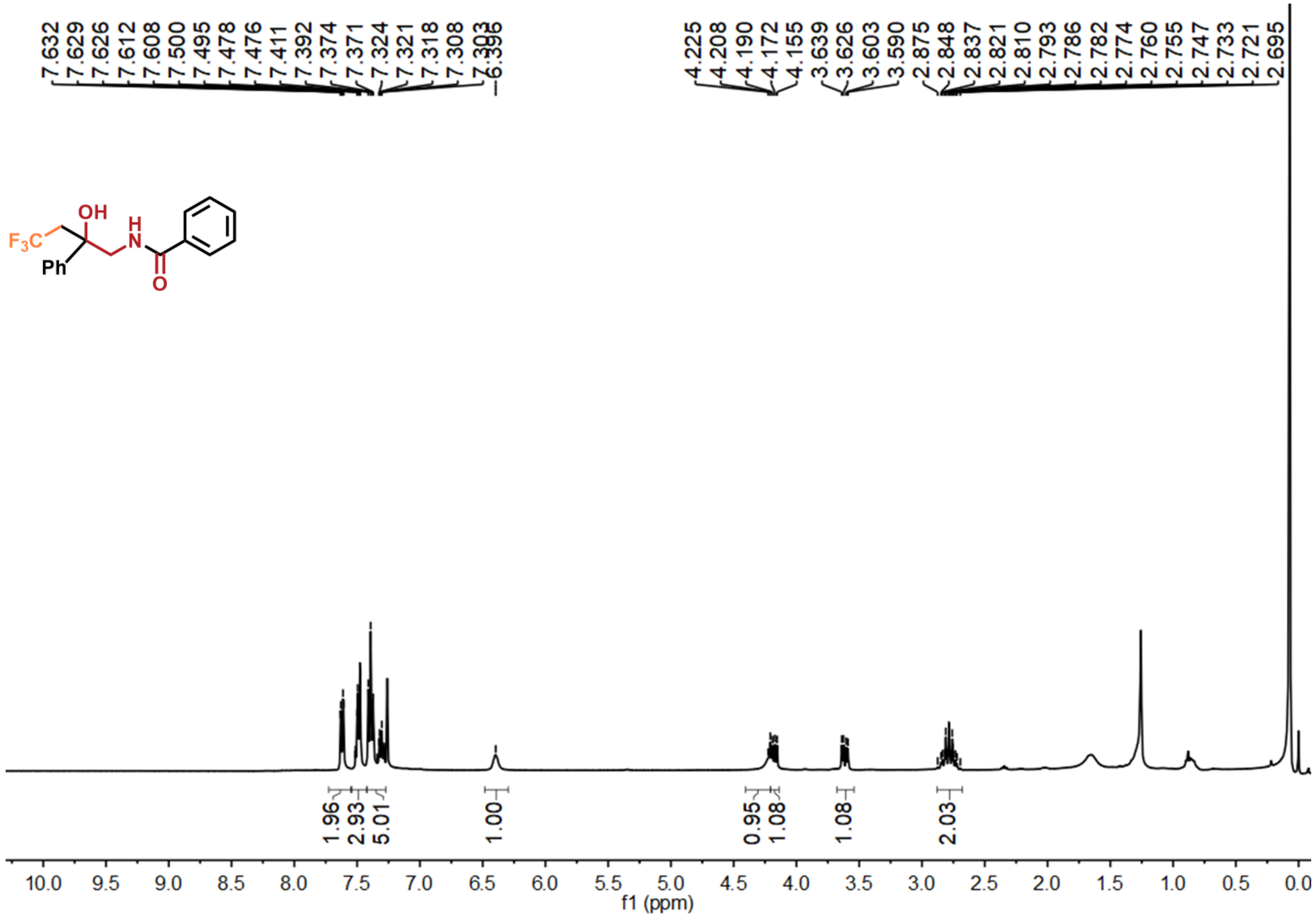

^{13}C NMR (100 MHz, $CDCl_3$) spectrum of **5a**

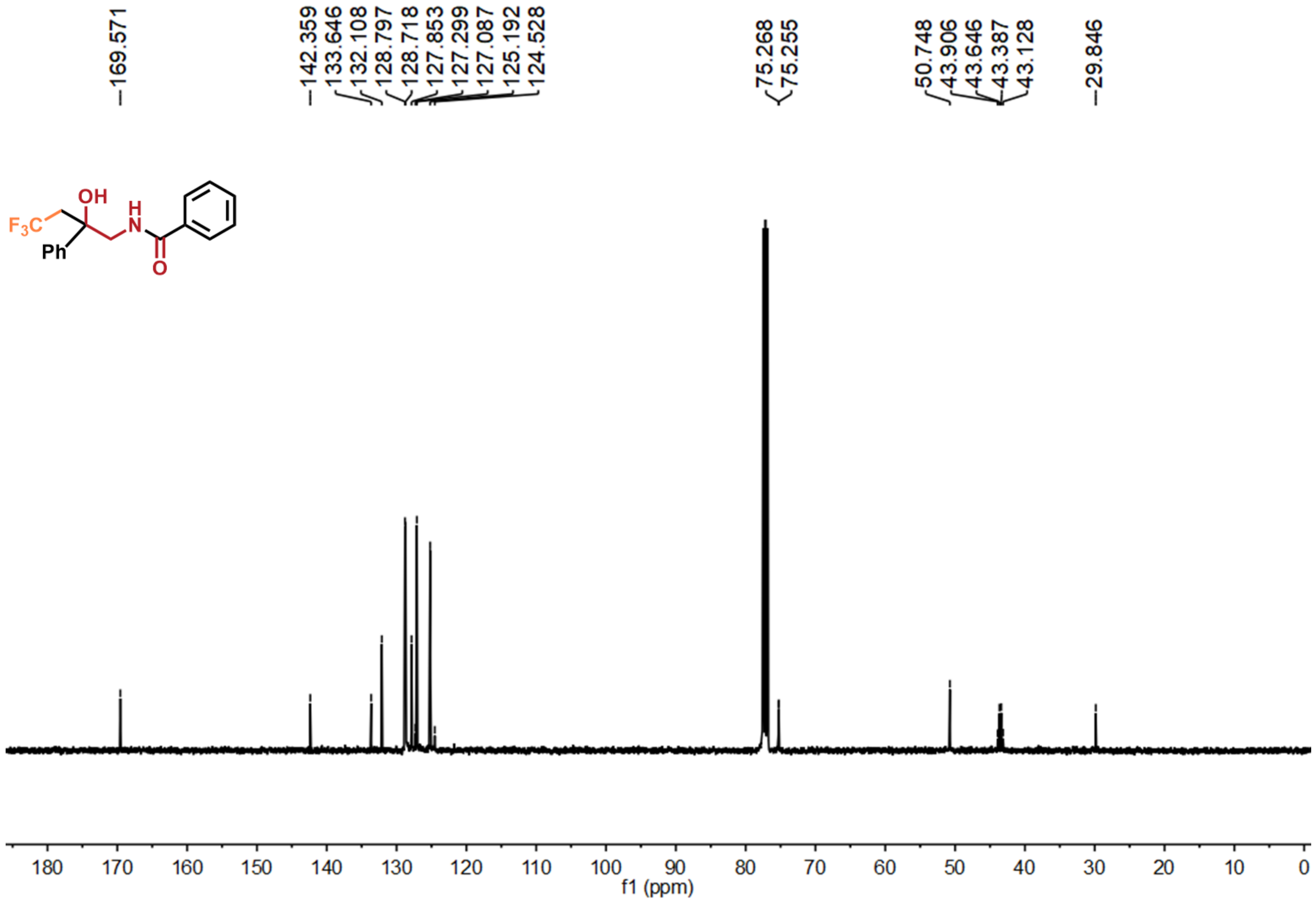

^{19}F NMR (376 MHz, $CDCl_3$) spectrum of **5a**

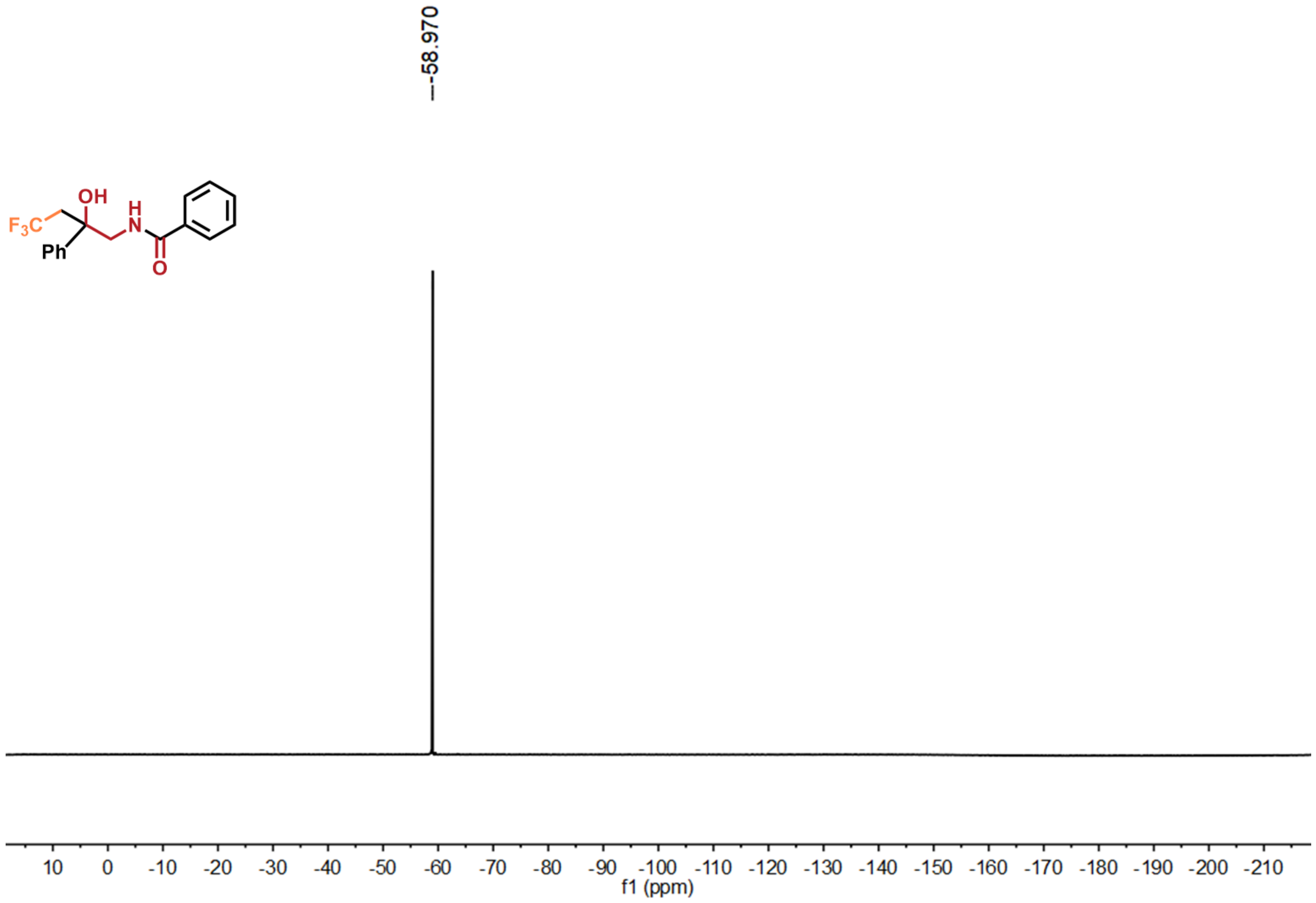

^{1}H NMR (400 MHz, $CDCl_3$) spectrum of **5b**

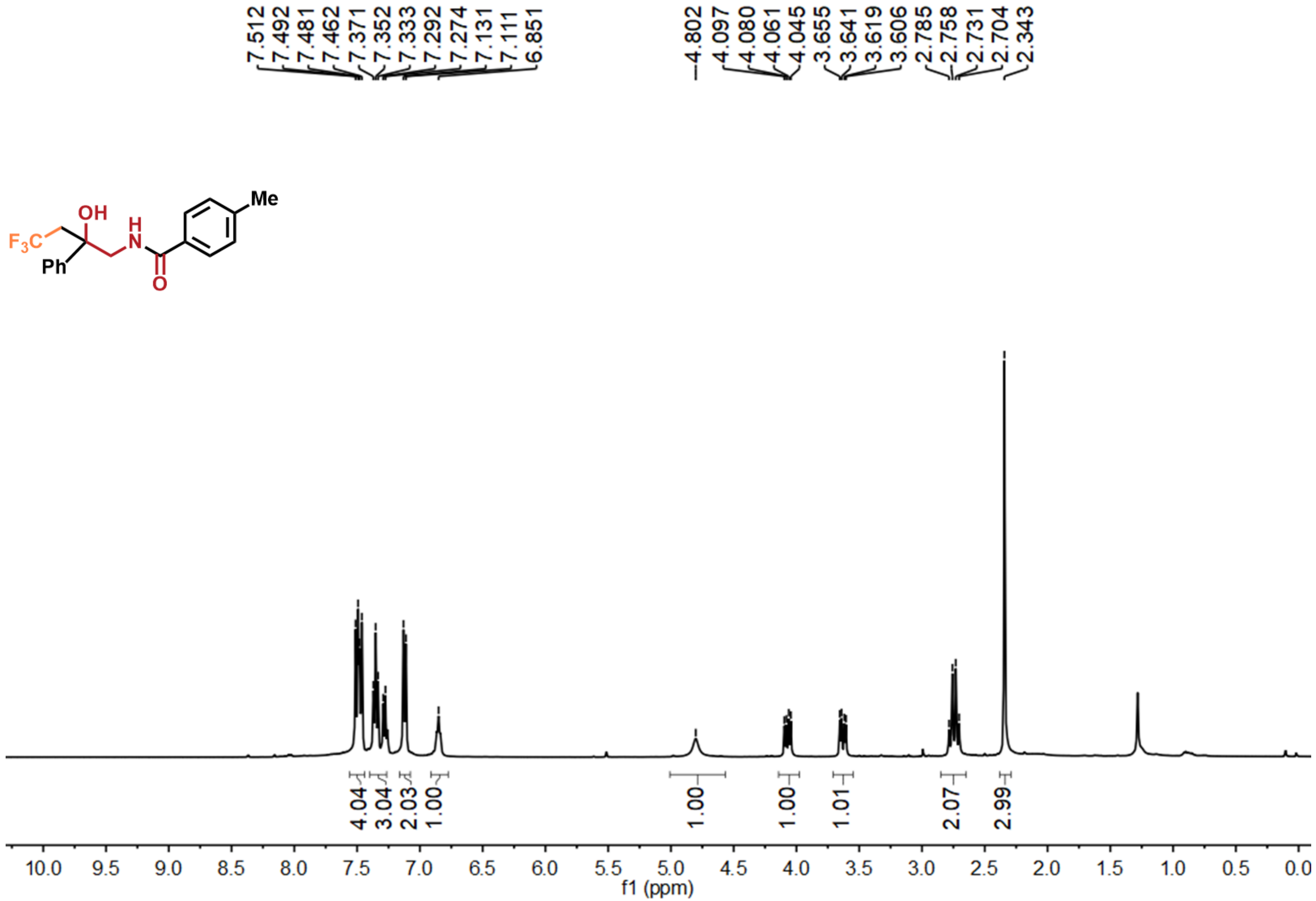

^{13}C NMR (100 MHz, $CDCl_3$) spectrum of **5b**

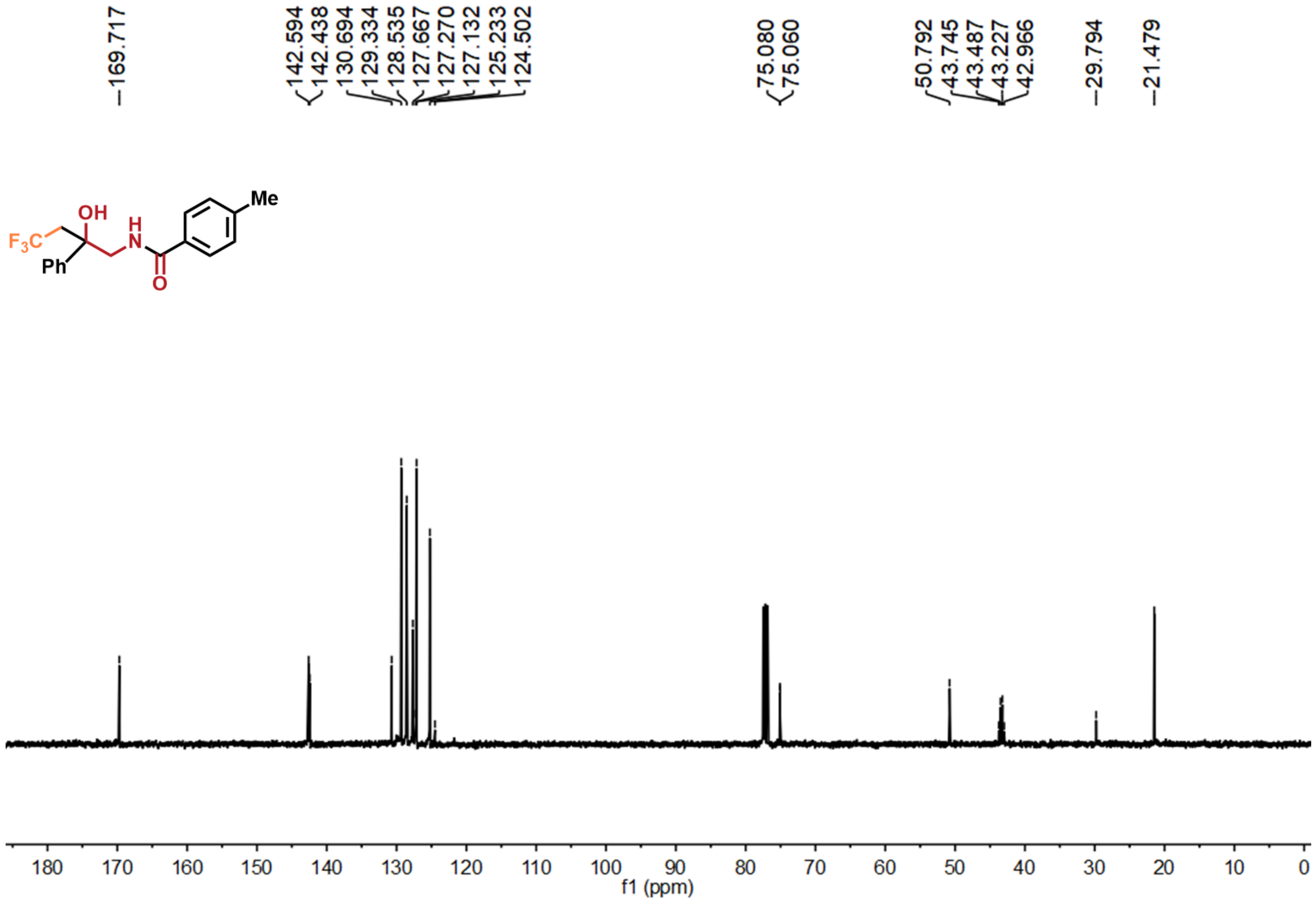

^{19}F NMR (376 MHz, $CDCl_3$) spectrum of **5b**

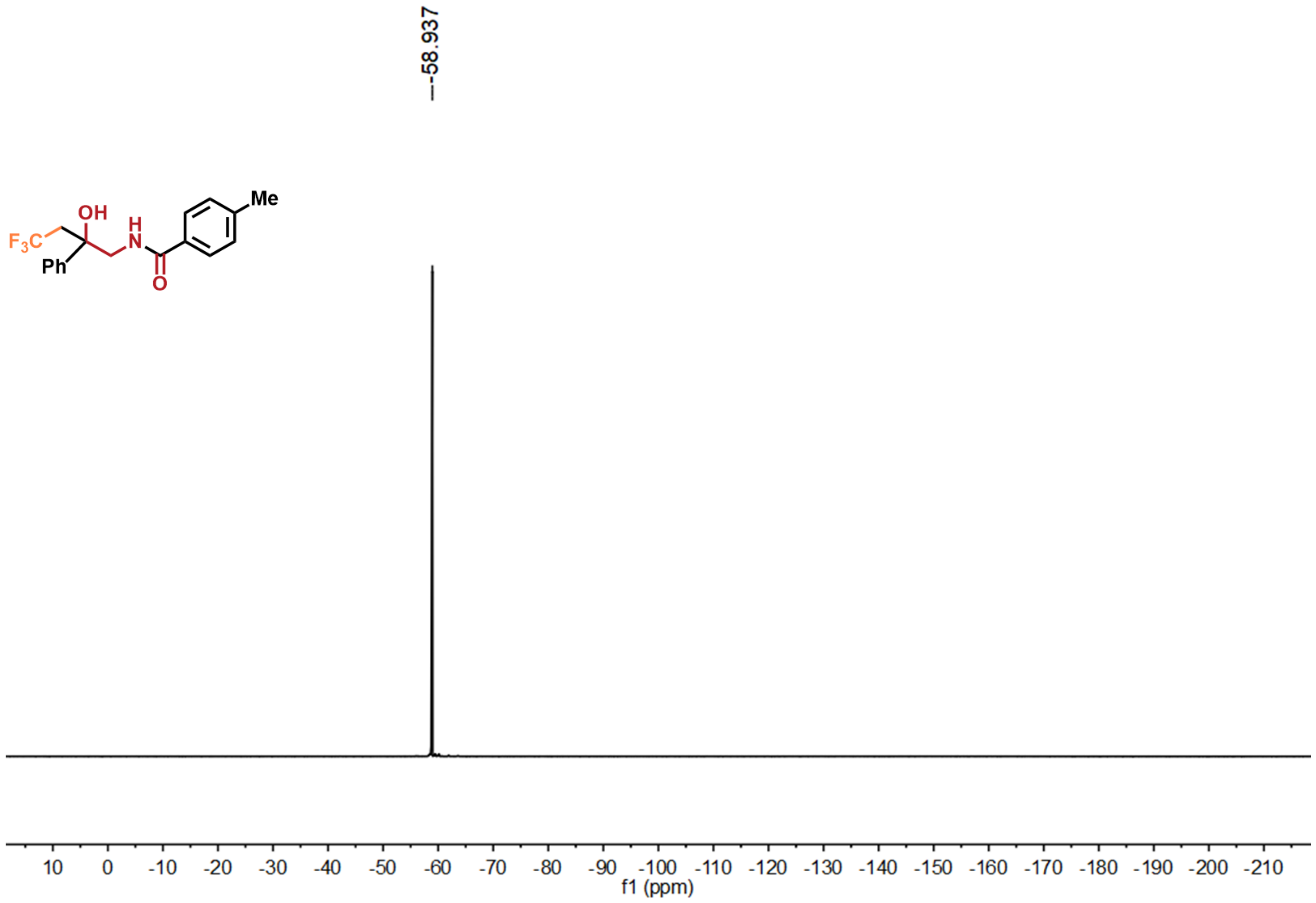

^{1}H NMR (400 MHz, $CDCl_3$) spectrum of **5c**

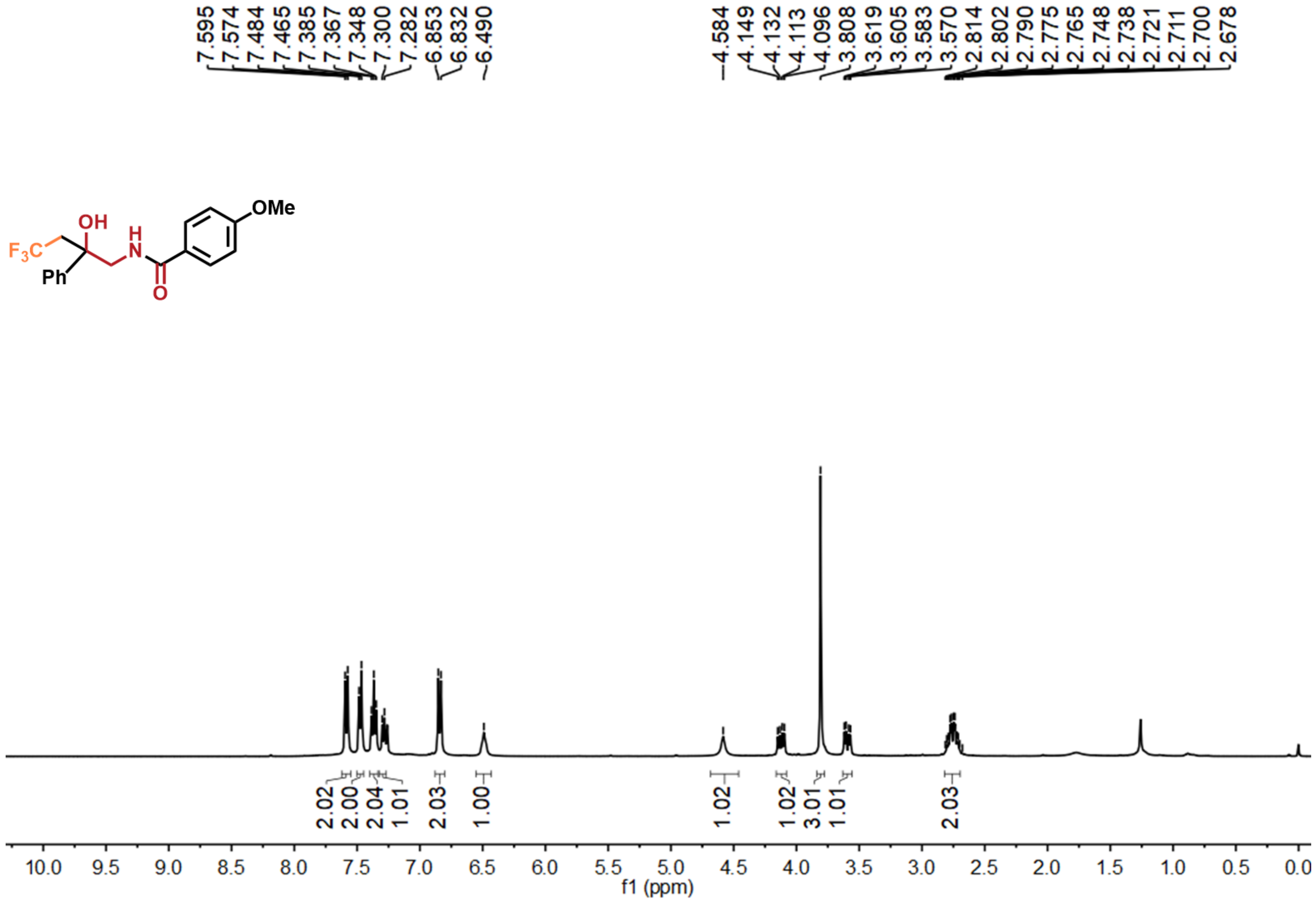

^{13}C NMR (100 MHz, $CDCl_3$) spectrum of **5c**

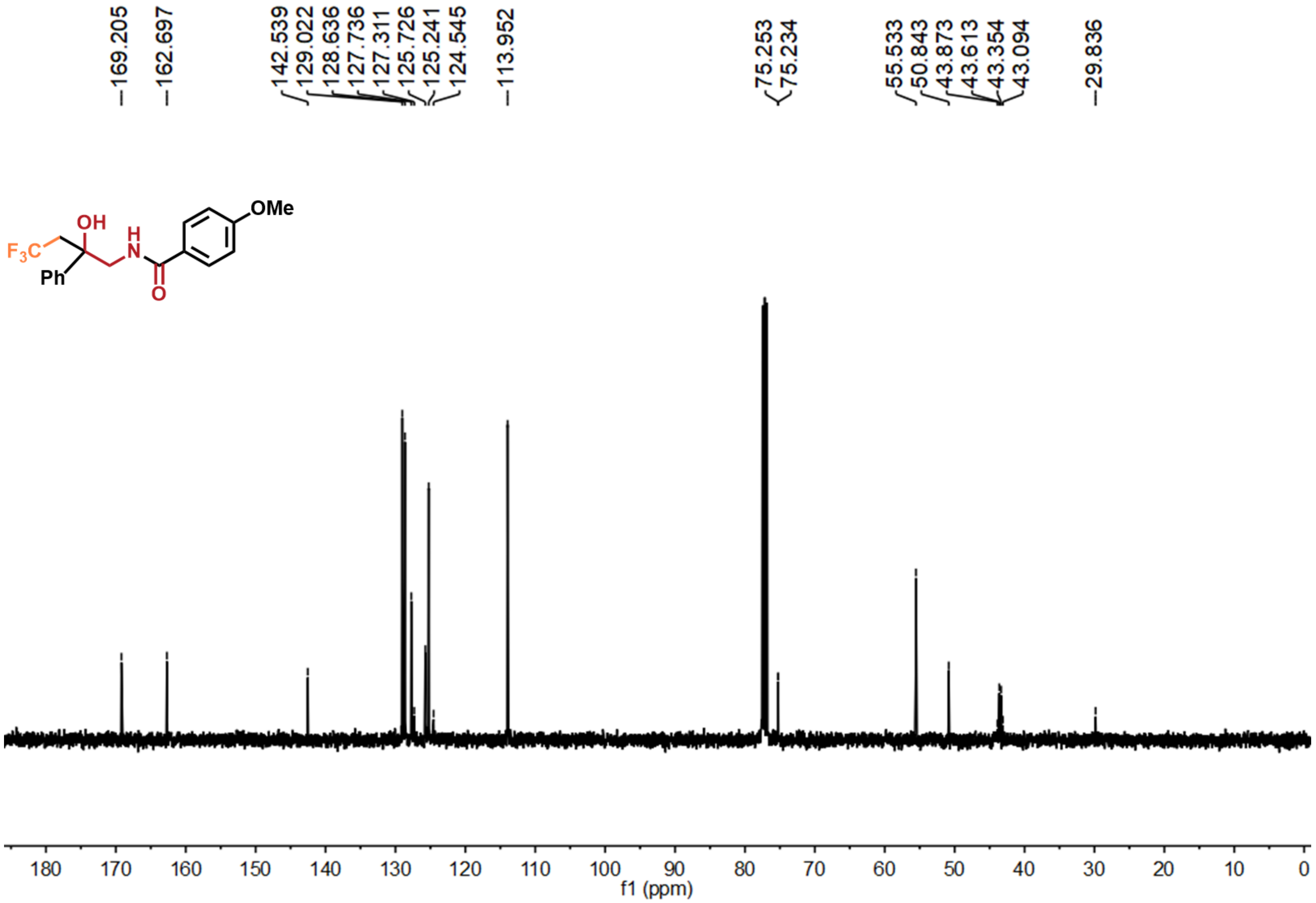

^{19}F NMR (376 MHz, $CDCl_3$) spectrum of **5c**

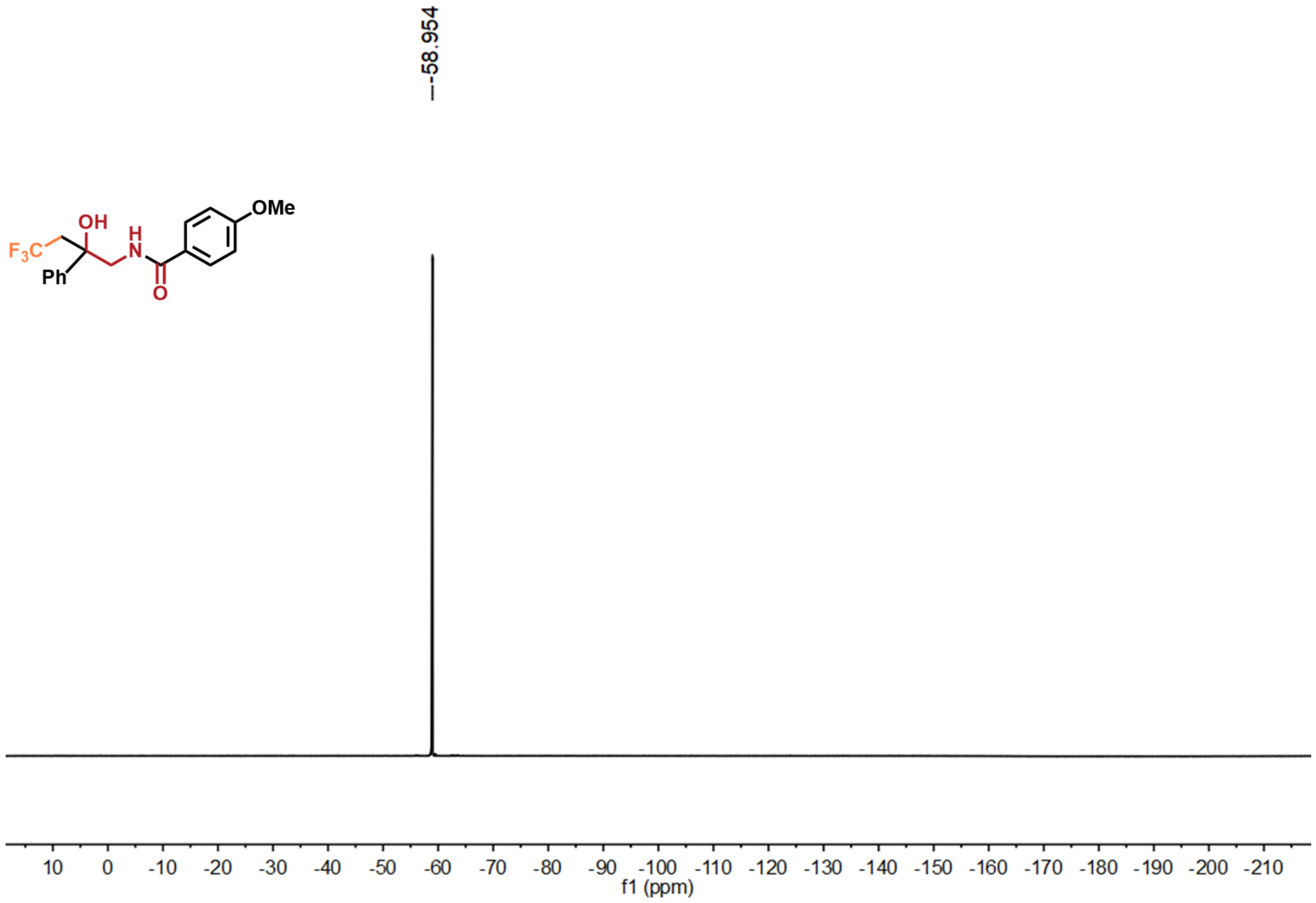

^{1}H NMR (400 MHz, $CDCl_3$) spectrum of **5d**

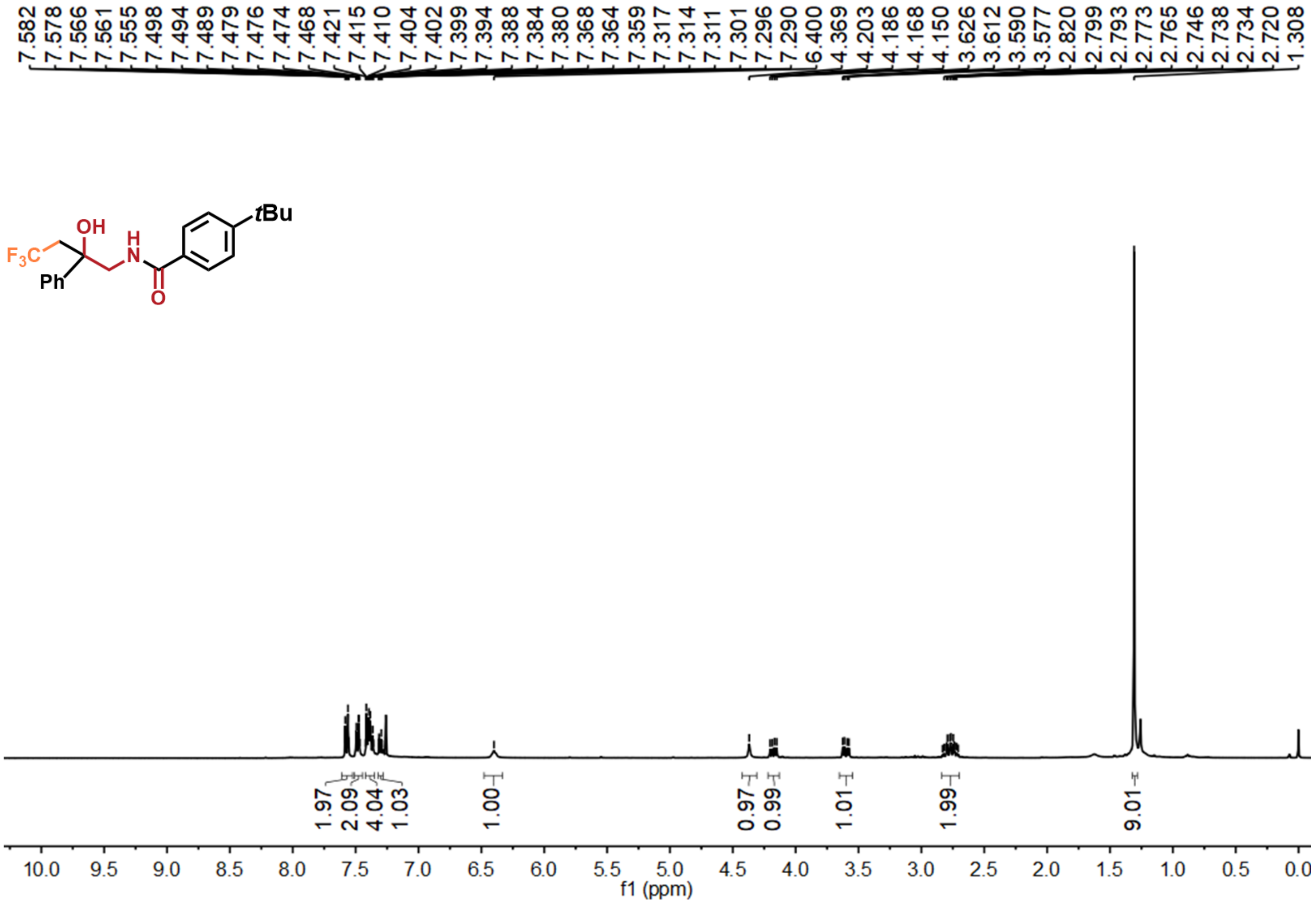

^{13}C NMR (100 MHz, $CDCl_3$) spectrum of **5d**

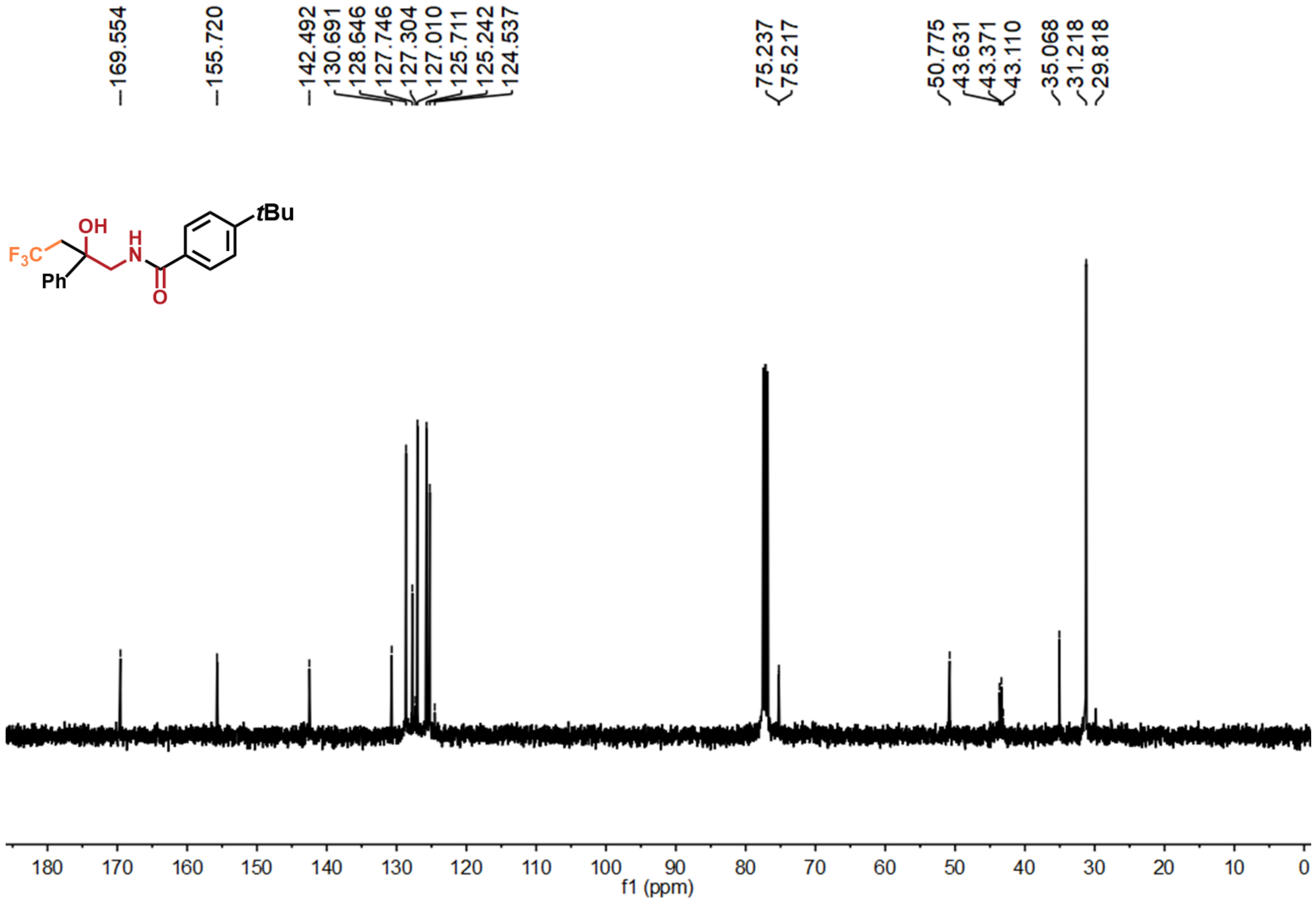

^{19}F NMR (376 MHz, $CDCl_3$) spectrum of **5d**

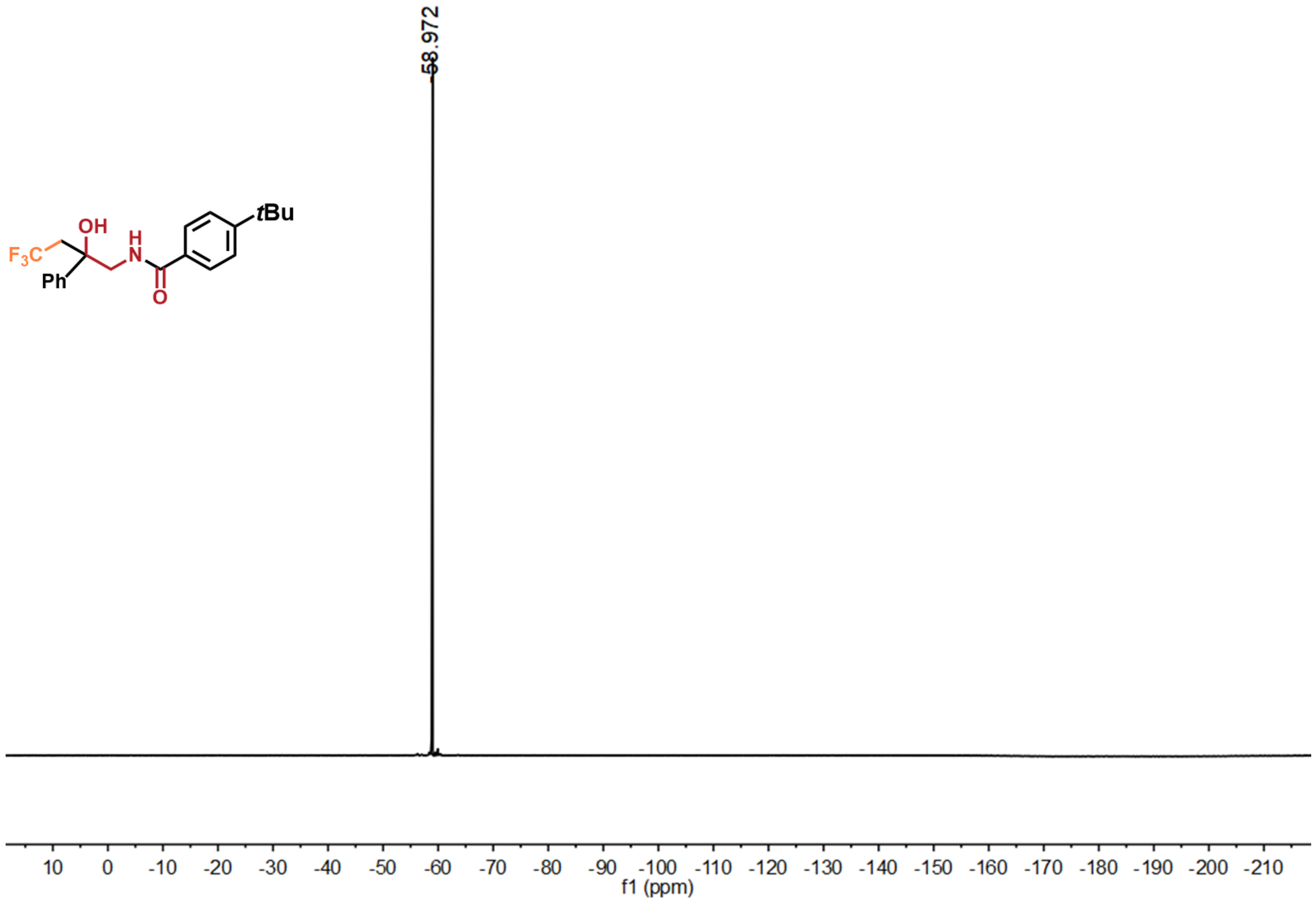

^{1}H NMR (400 MHz, $CDCl_3$) spectrum of **5e**

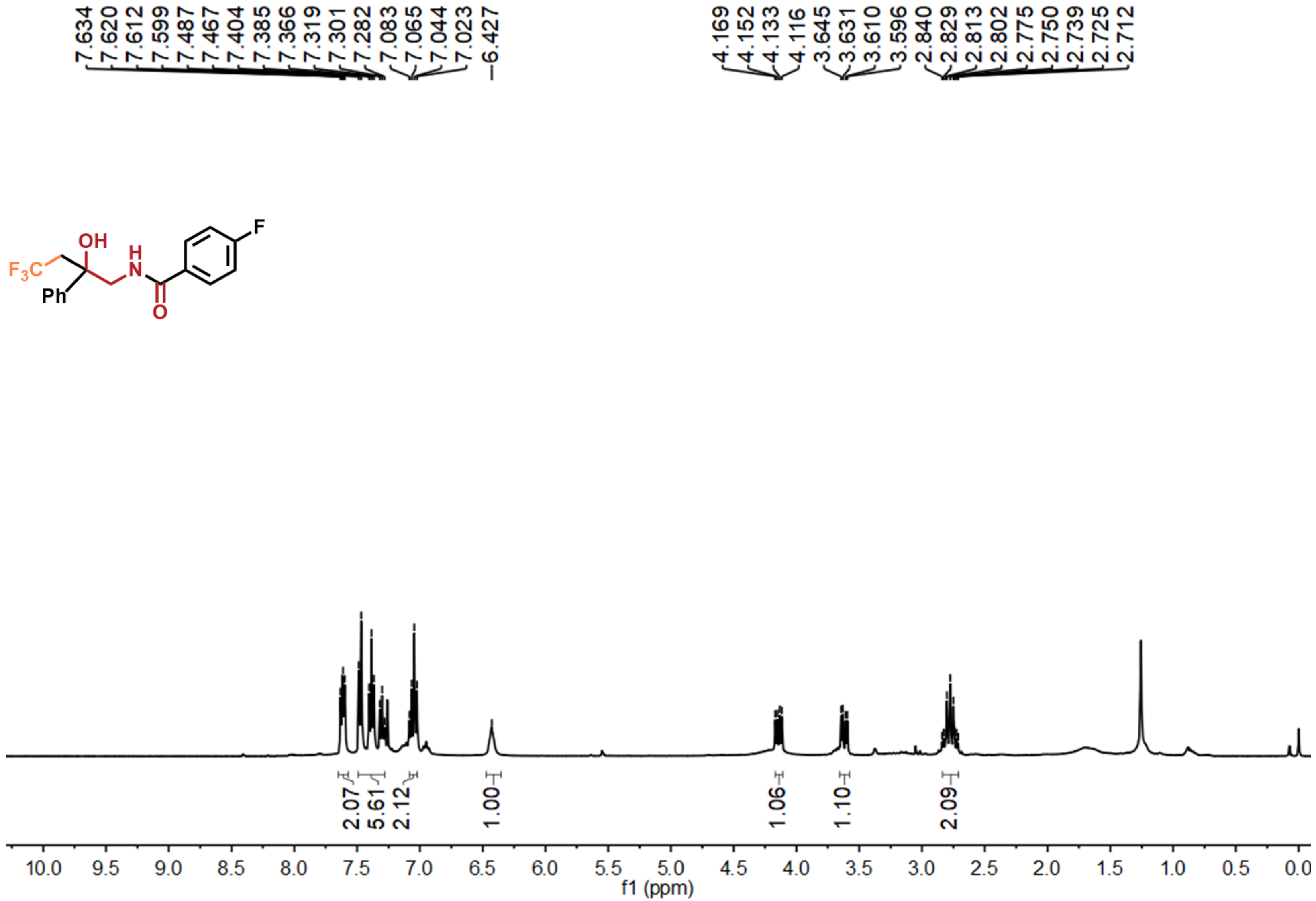

^{13}C NMR (100 MHz, $CDCl_3$) spectrum of **5e**

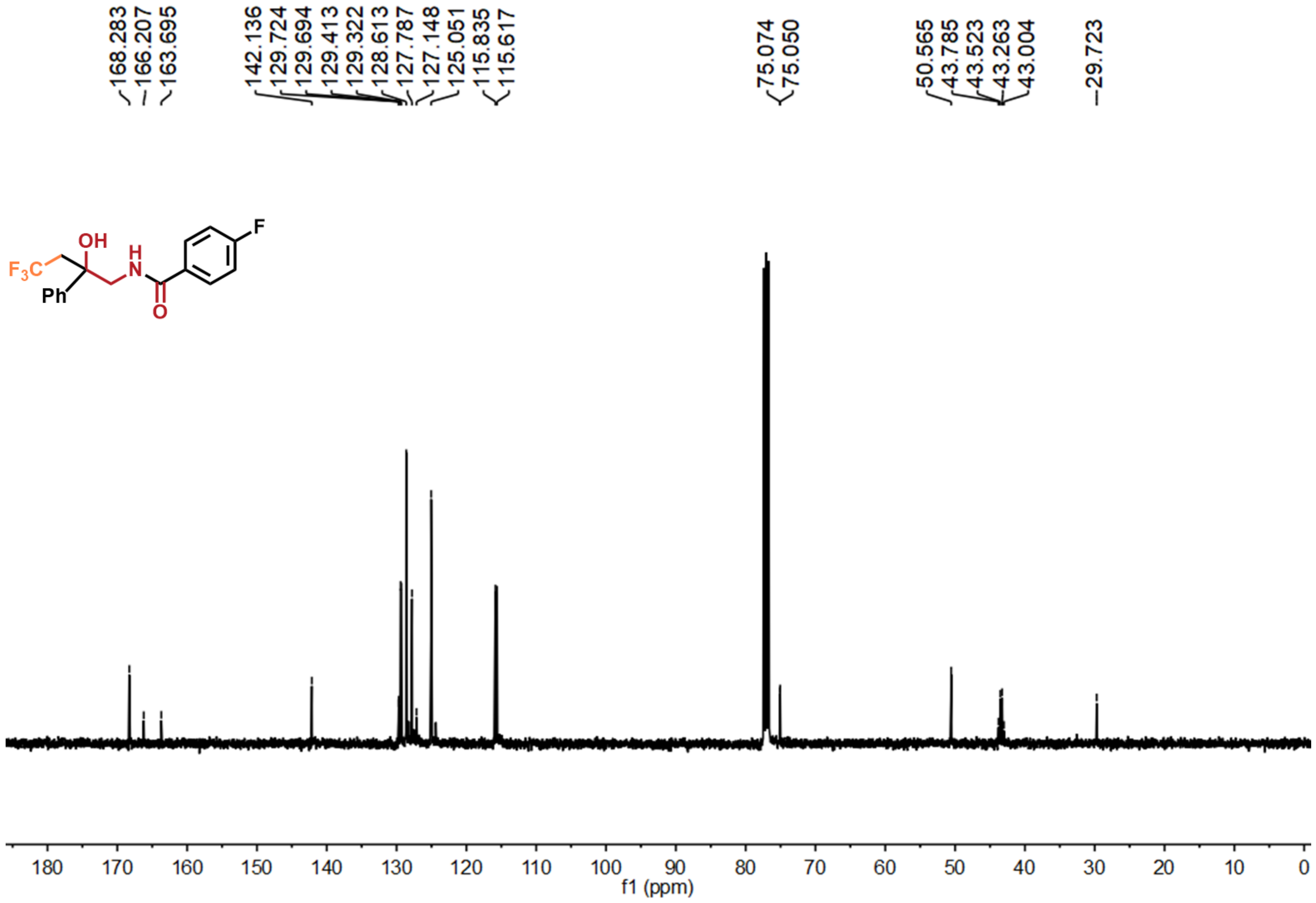

^{19}F NMR (376 MHz, $CDCl_3$) spectrum of **5e**

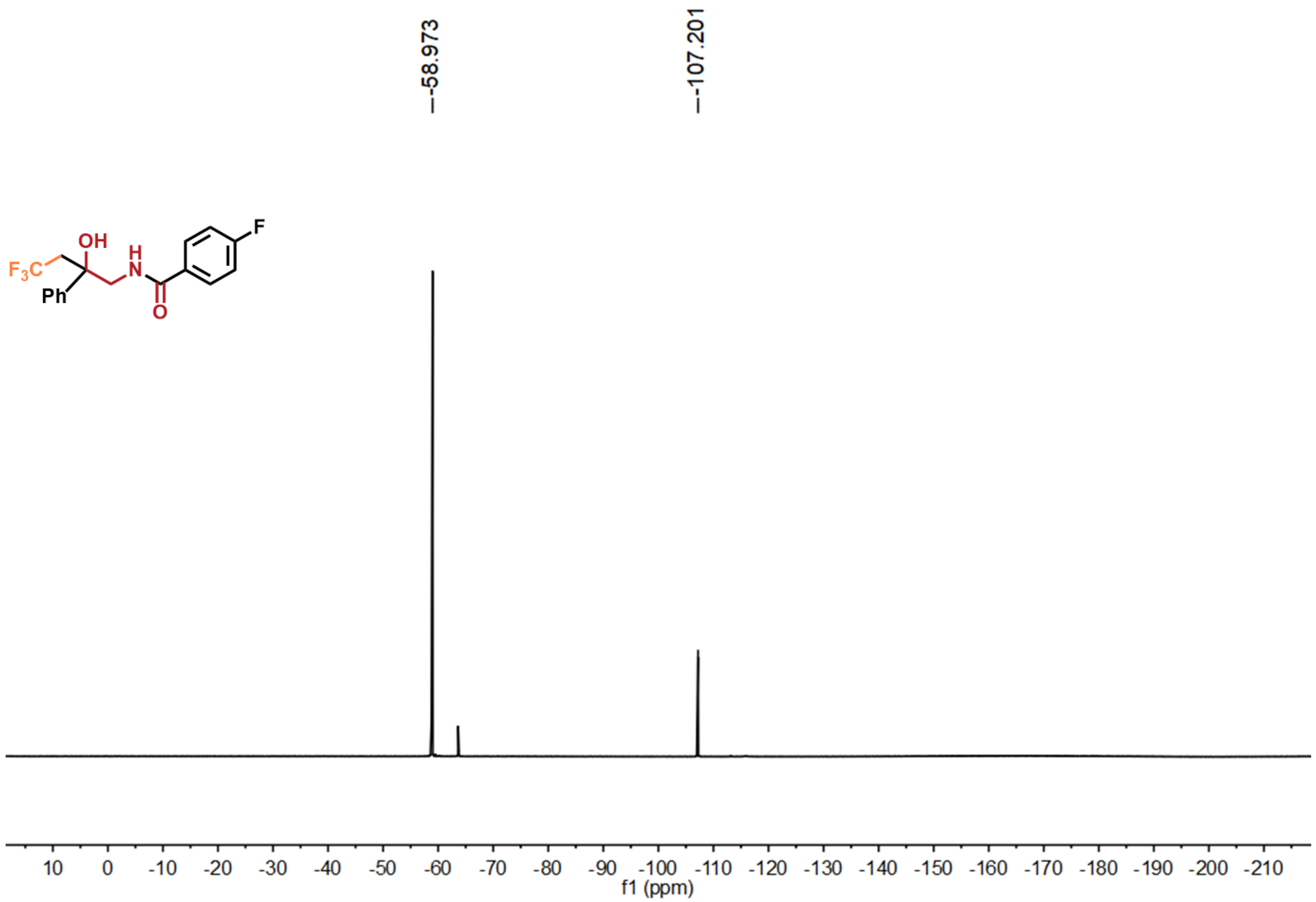

^{1}H NMR (400 MHz, $CDCl_3$) spectrum of **5f**

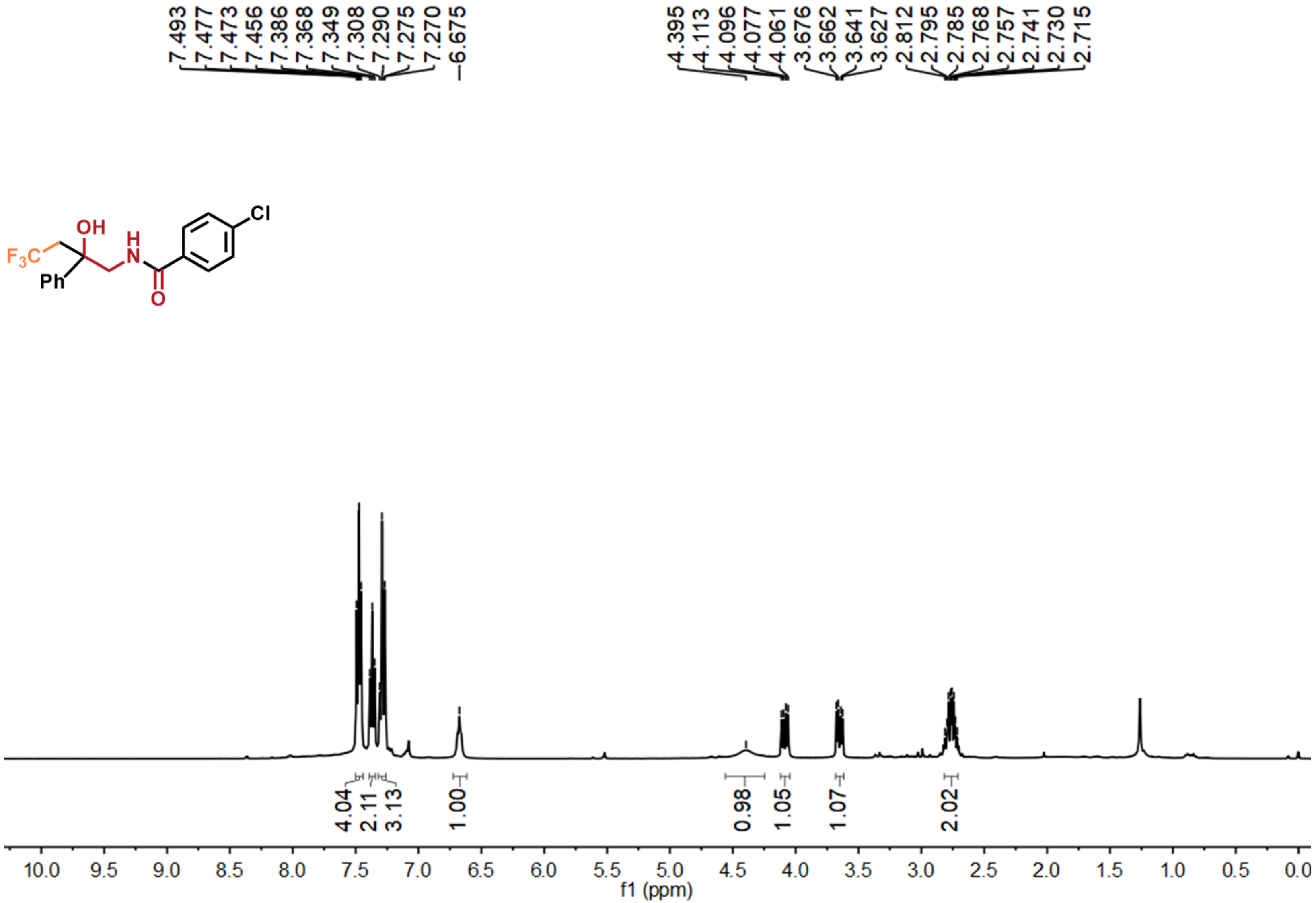

^{13}C NMR (100 MHz, $CDCl_3$) spectrum of **5f**

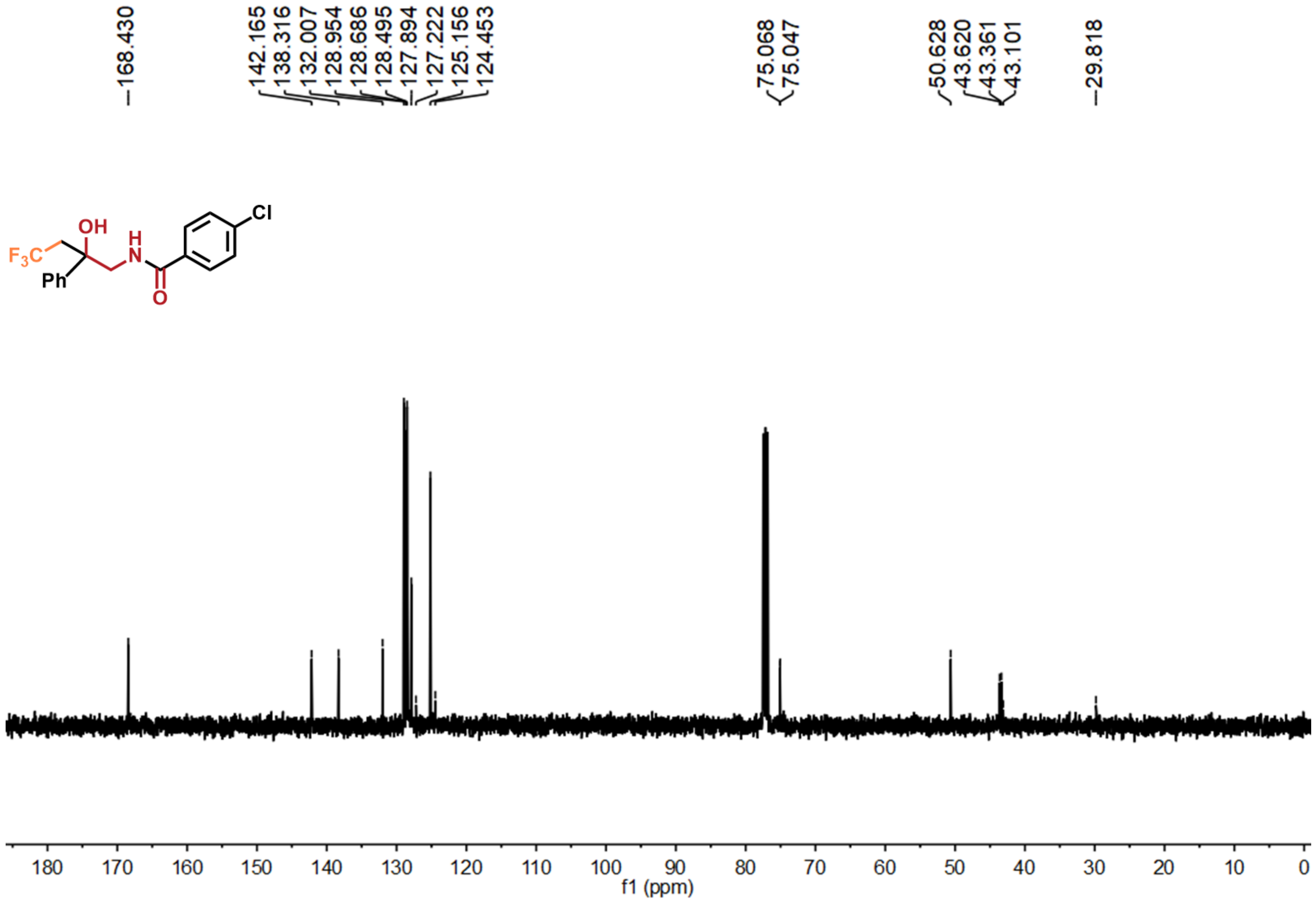

^{19}F NMR (376 MHz, $CDCl_3$) spectrum of **5f**

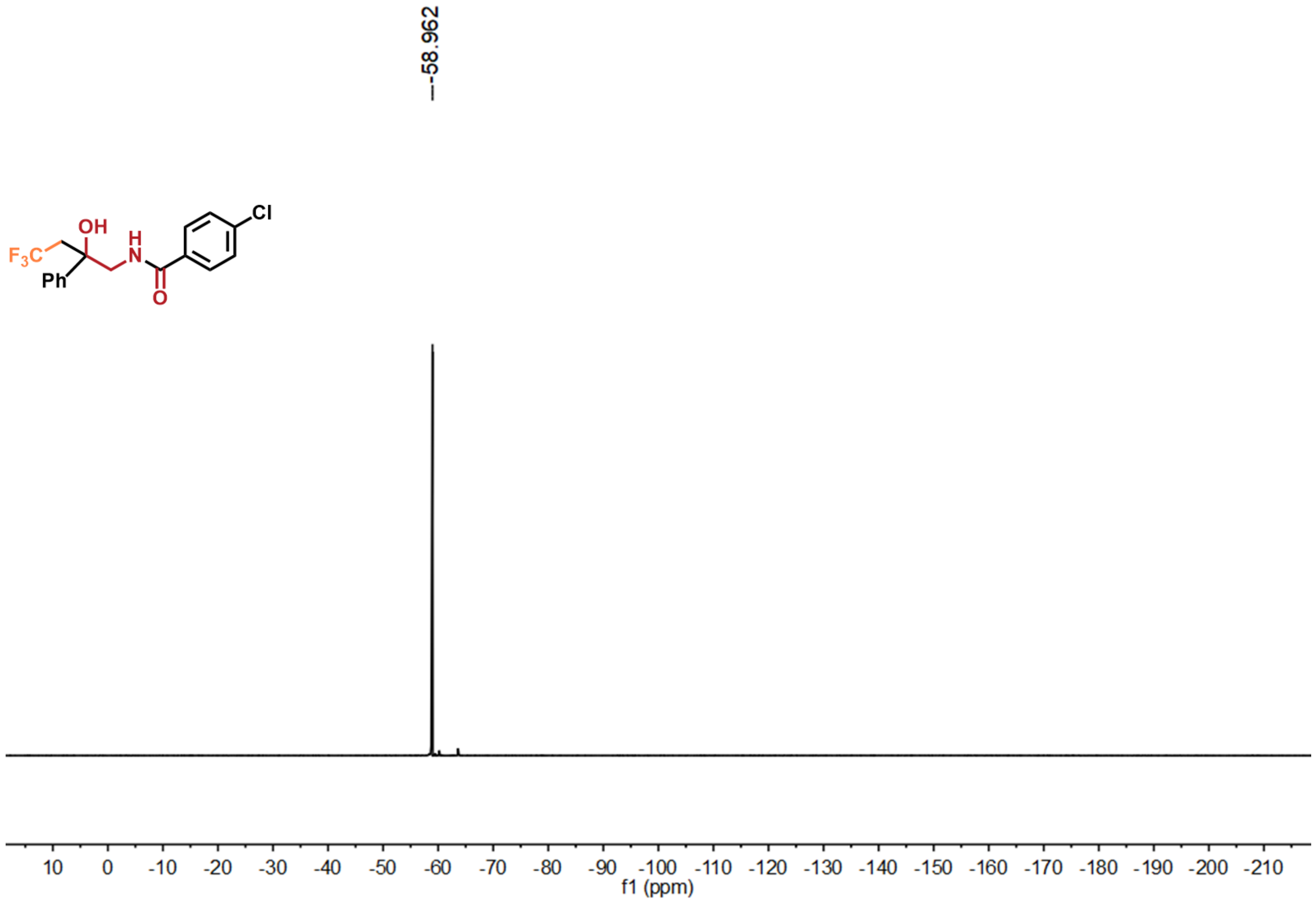

^{1}H NMR (400 MHz, $CDCl_3$) spectrum of **5g**

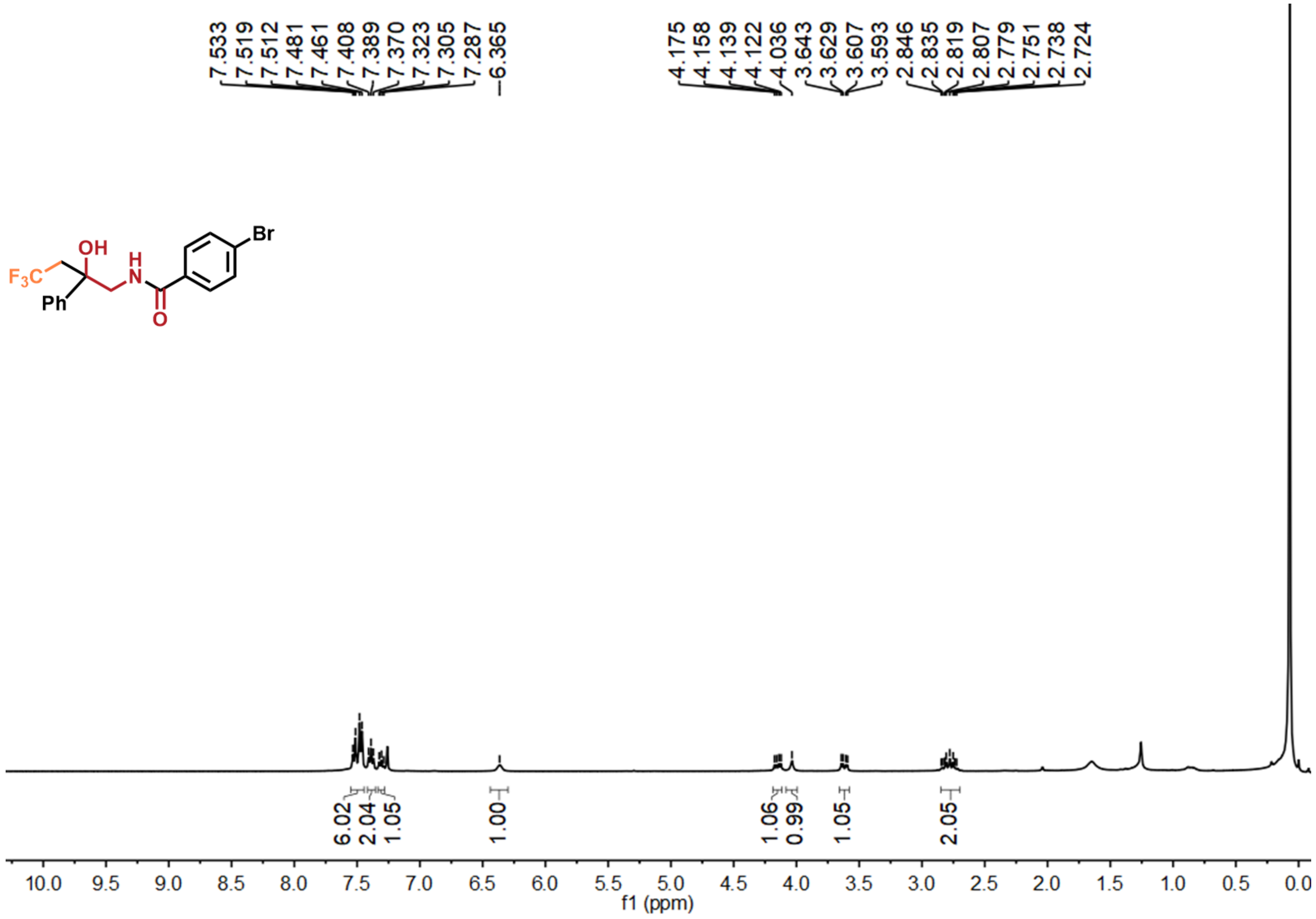

^{13}C NMR (100 MHz, $CDCl_3$) spectrum of **5g**

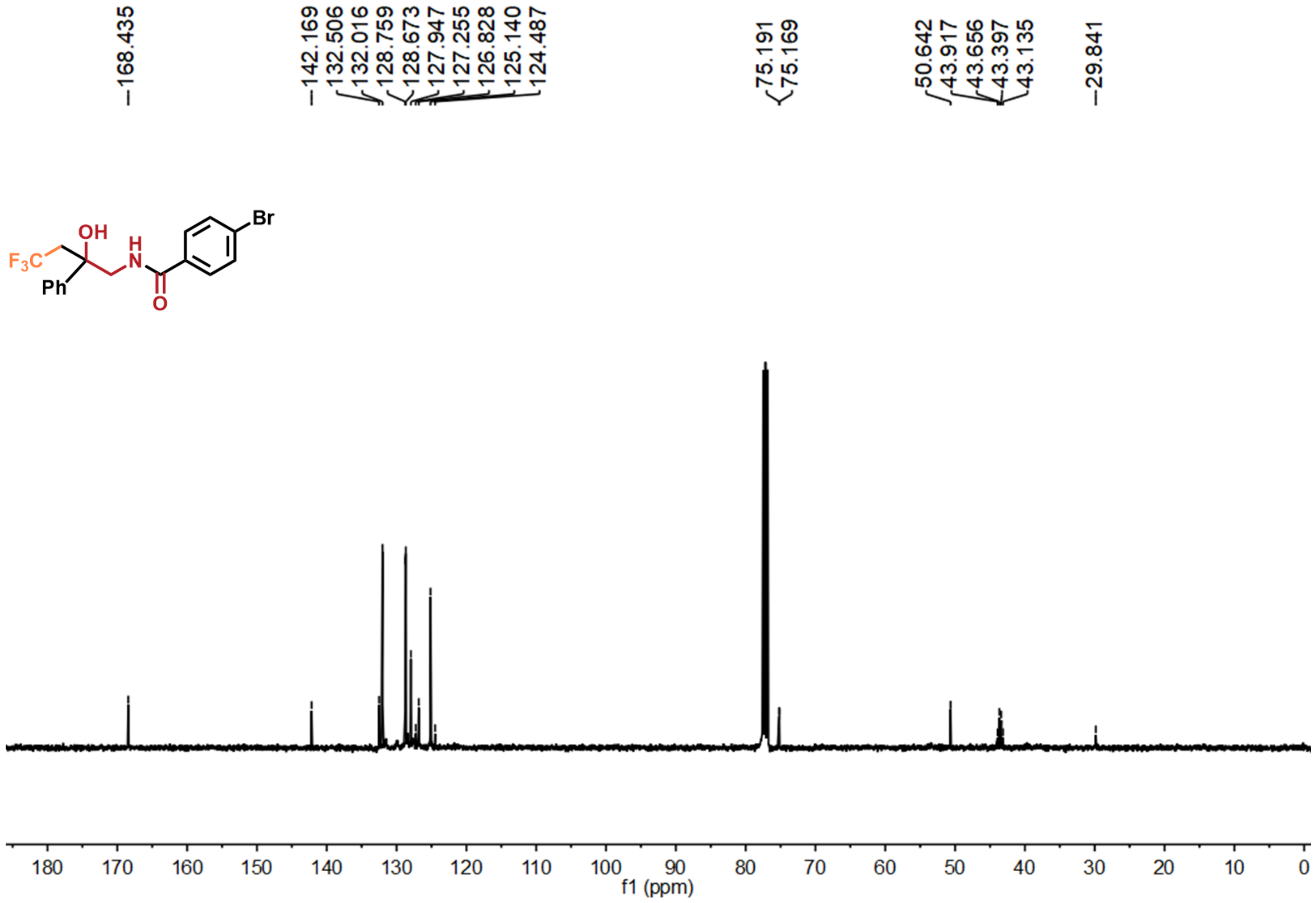

^{19}F NMR (376 MHz, $CDCl_3$) spectrum of **5g**

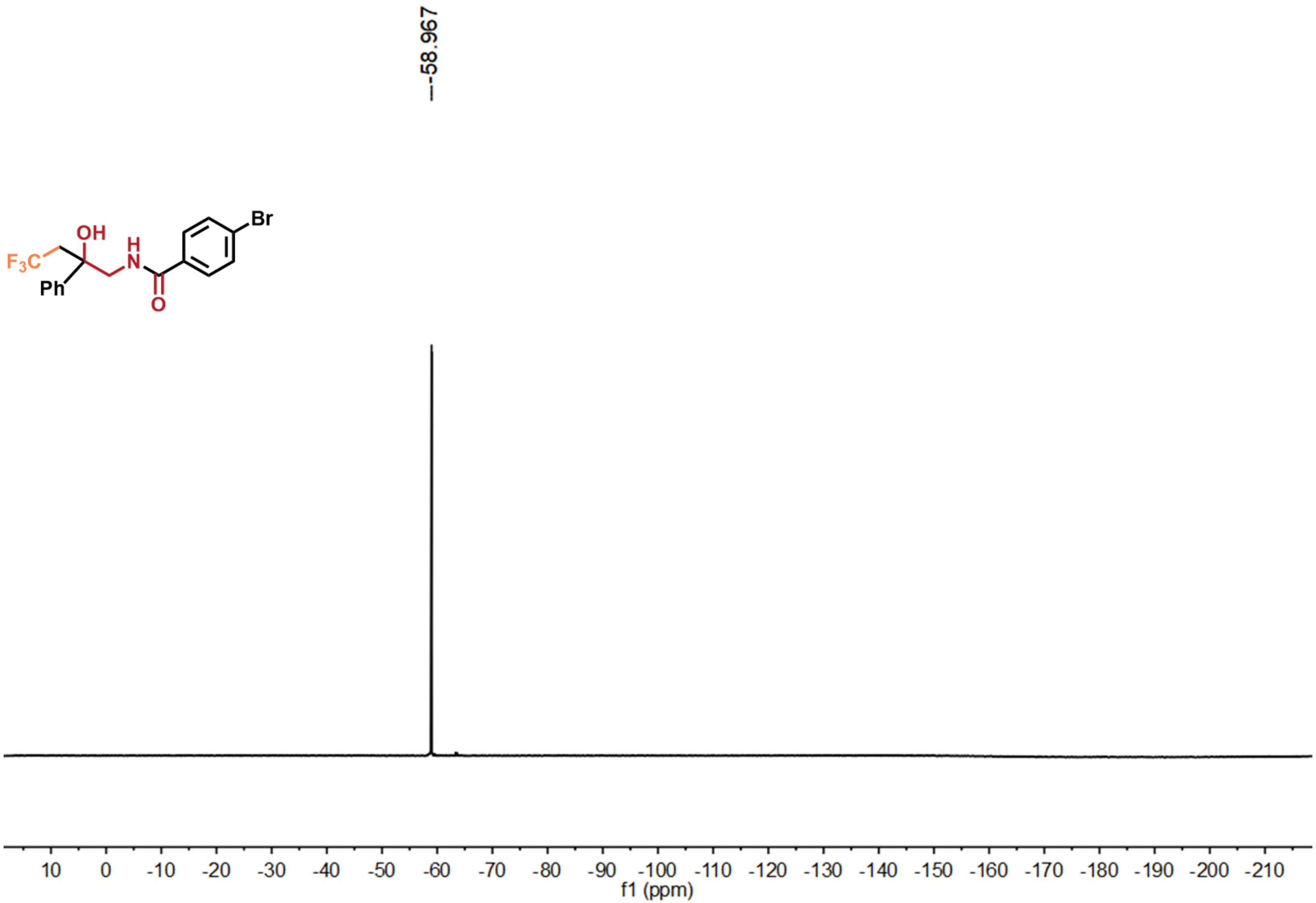

^{1}H NMR (400 MHz, $CDCl_3$) spectrum of **5h**

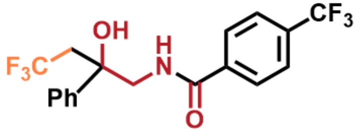

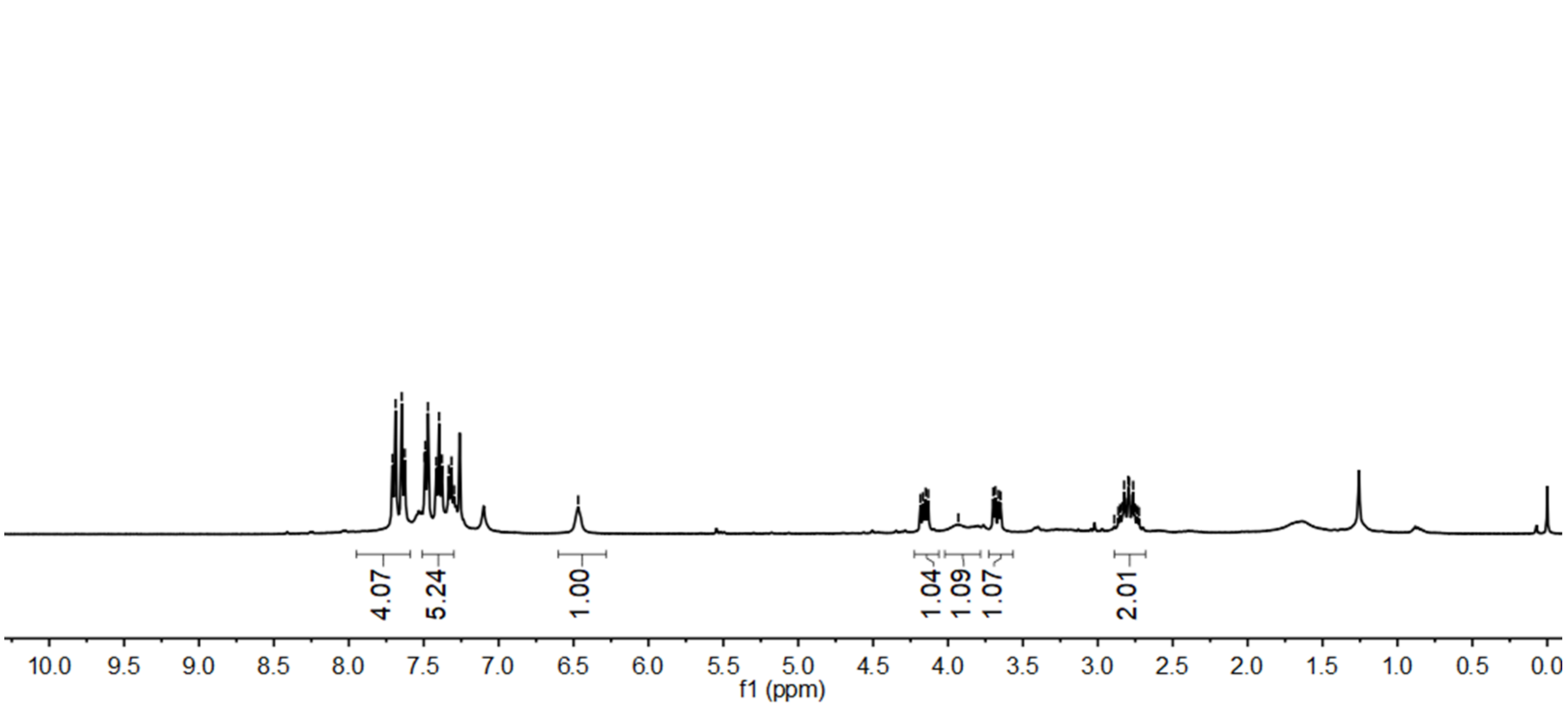

^{13}C NMR (100 MHz, $CDCl_3$) spectrum of **5h**

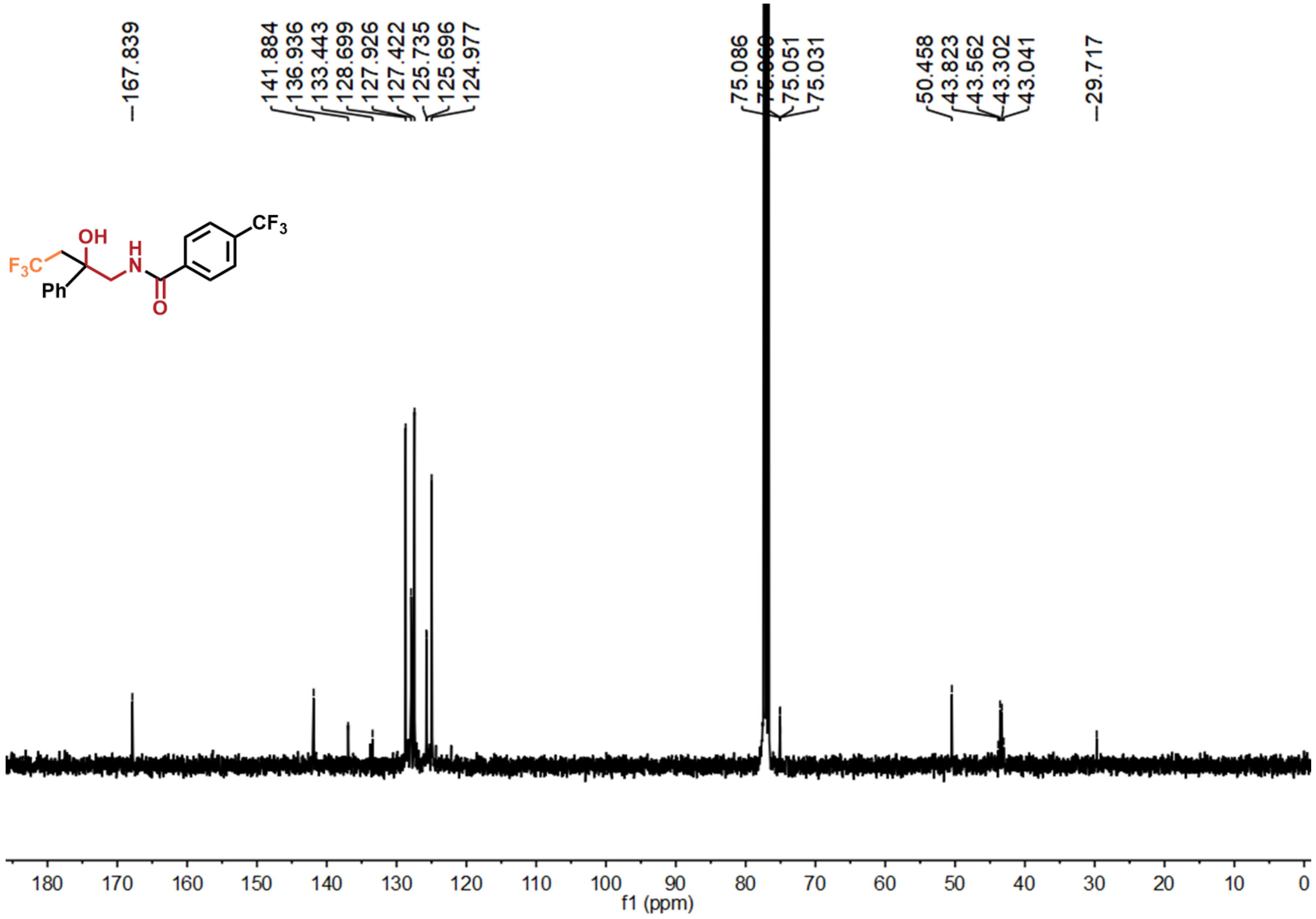

^{19}F NMR (376 MHz, $CDCl_3$) spectrum of **5h**

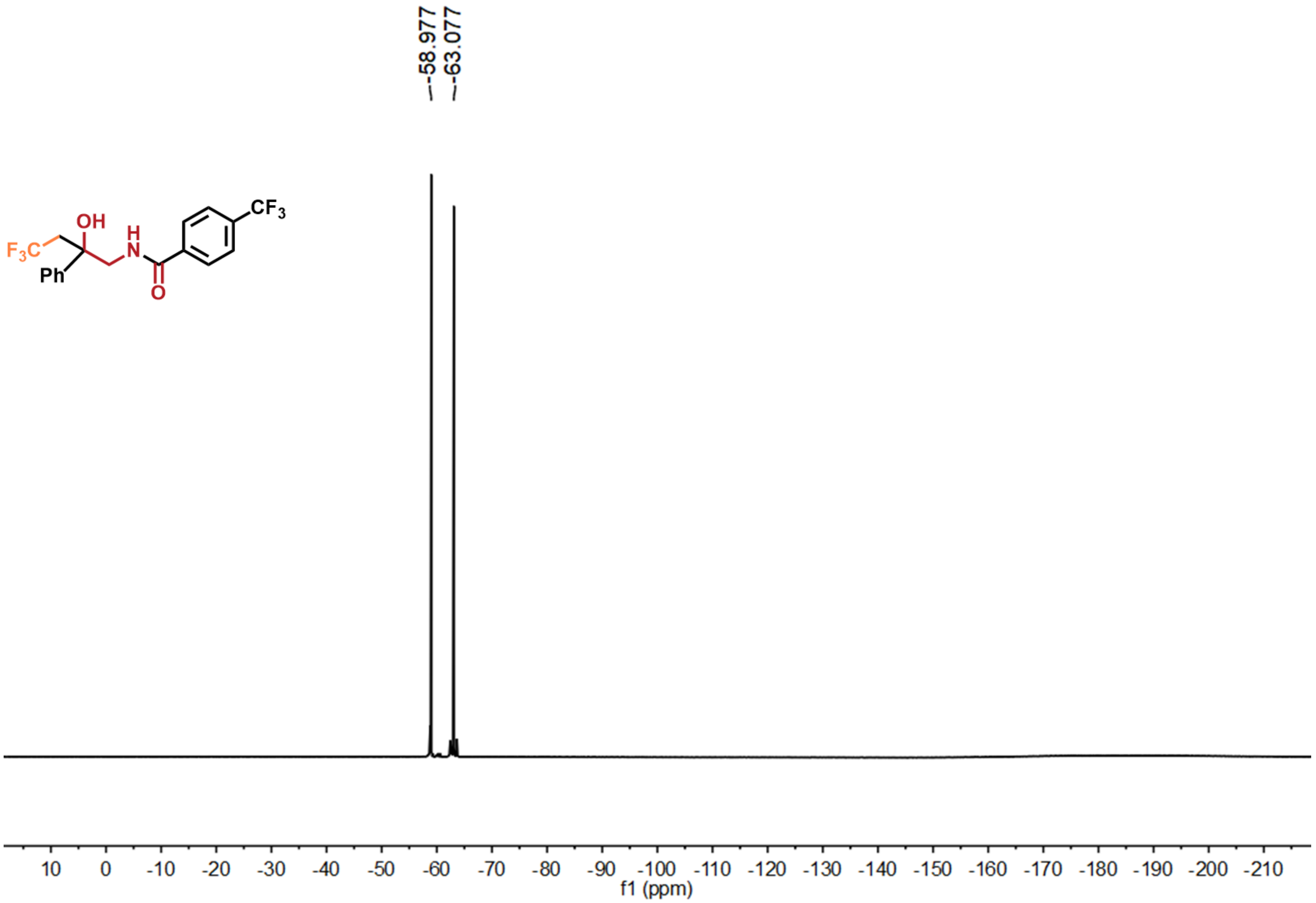

^{1}H NMR (400 MHz, $CDCl_3$) spectrum of **5i**

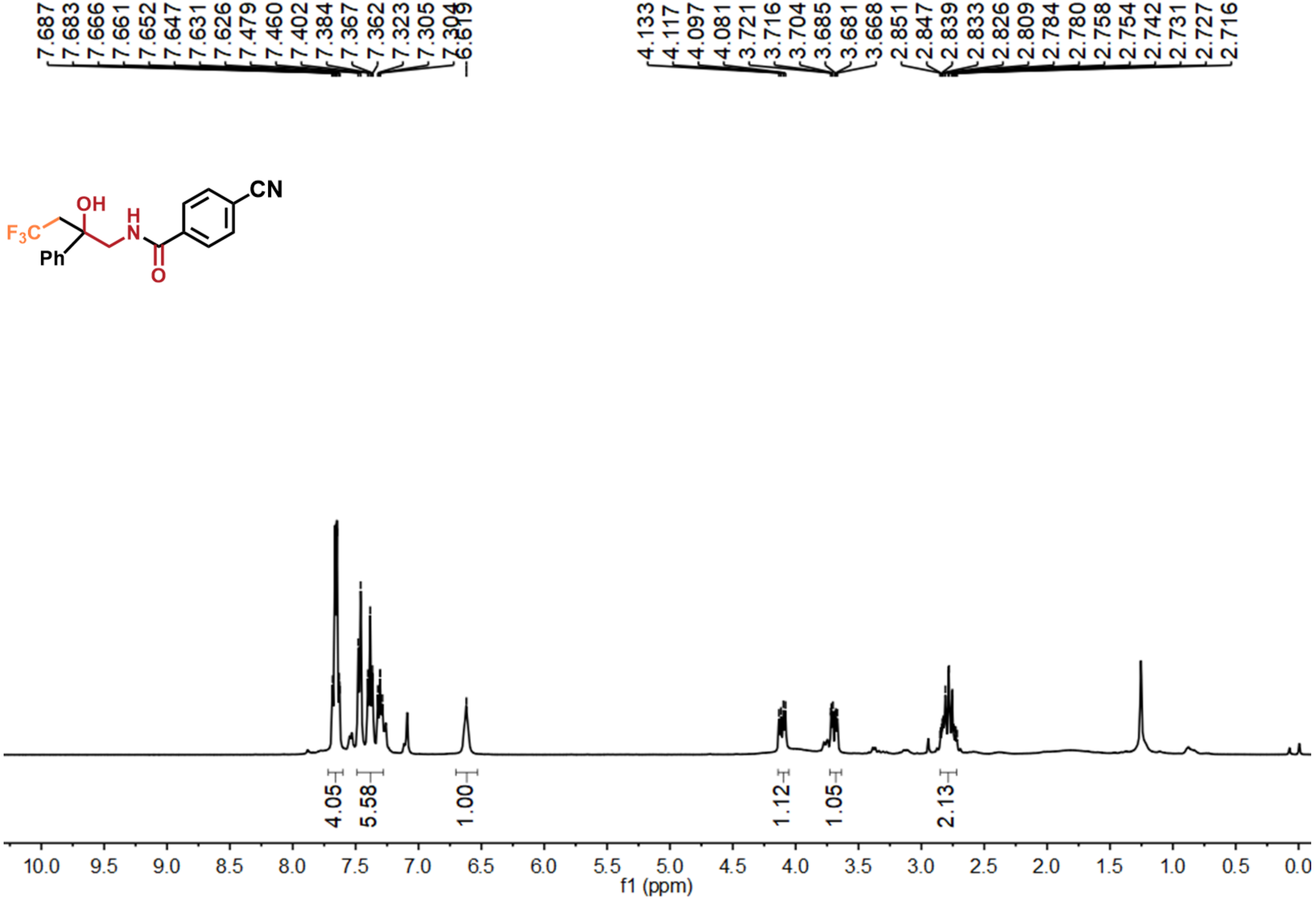

^{13}C NMR (100 MHz, $CDCl_3$) spectrum of **5i**

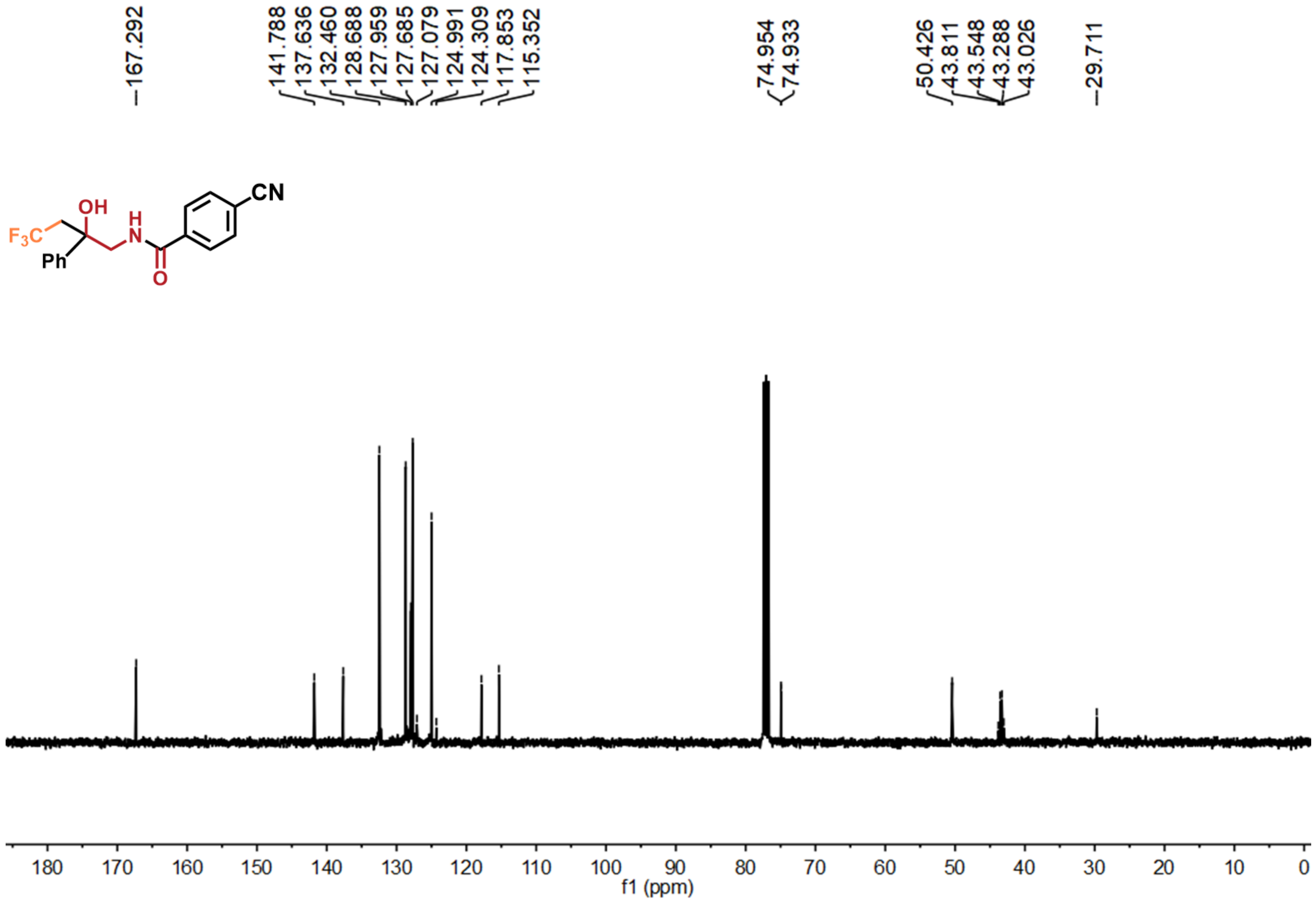

^{19}F NMR (376 MHz, $CDCl_3$) spectrum of **5i**

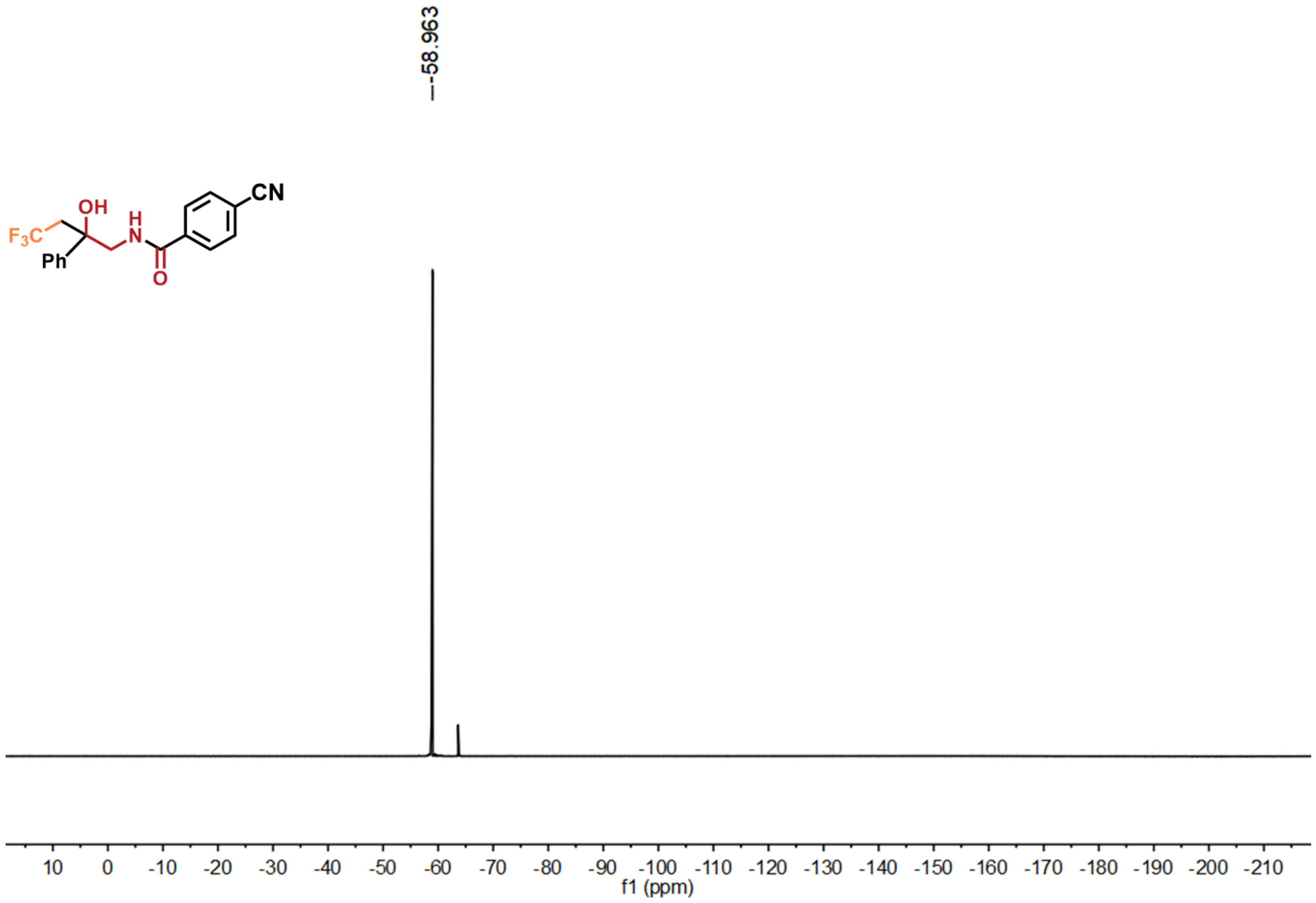

^{1}H NMR (400 MHz, $CDCl_3$) spectrum of **5j**

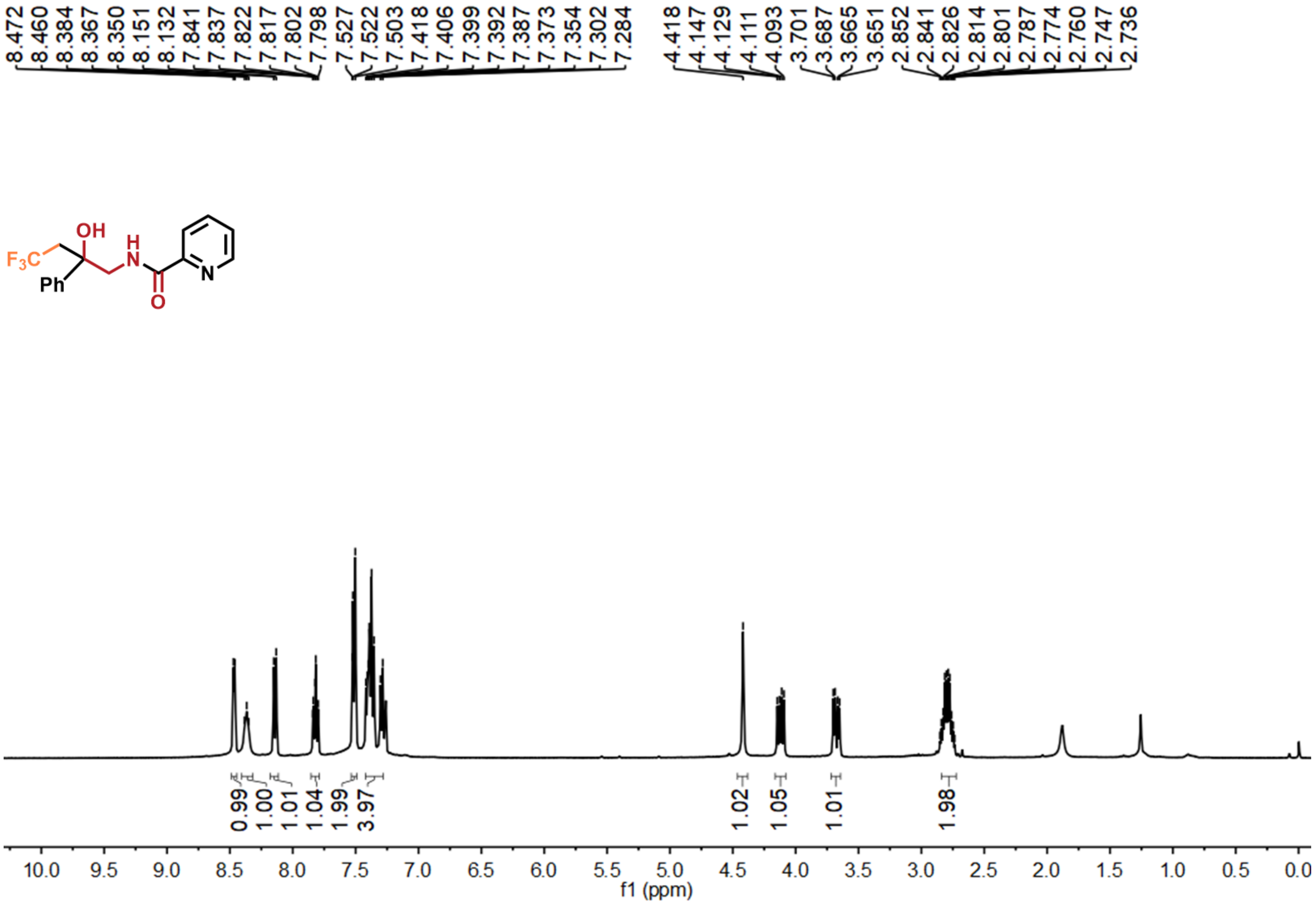

^{13}C NMR (100 MHz, $CDCl_3$) spectrum of **5j**

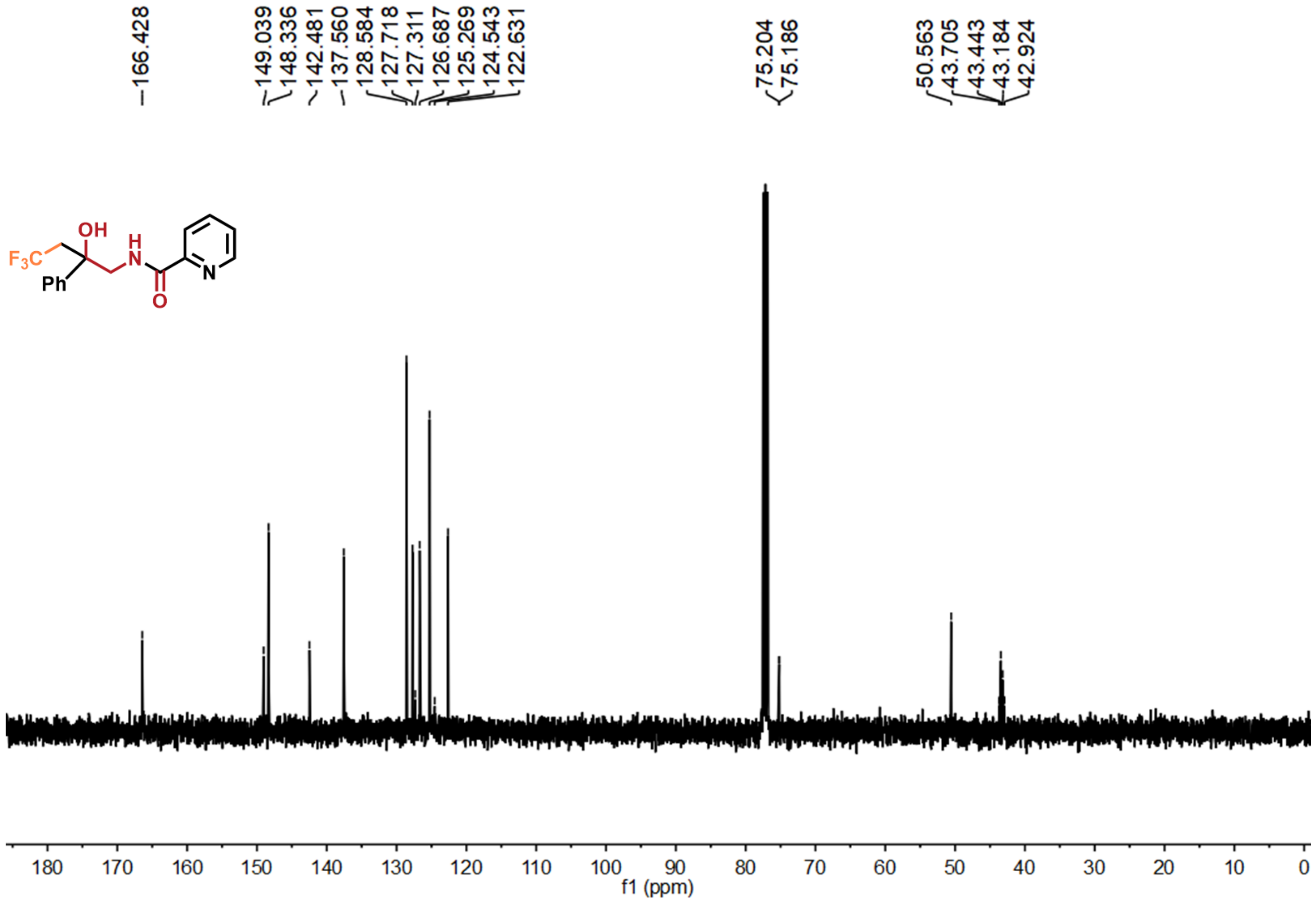

^{19}F NMR (376 MHz, $CDCl_3$) spectrum of **5j**

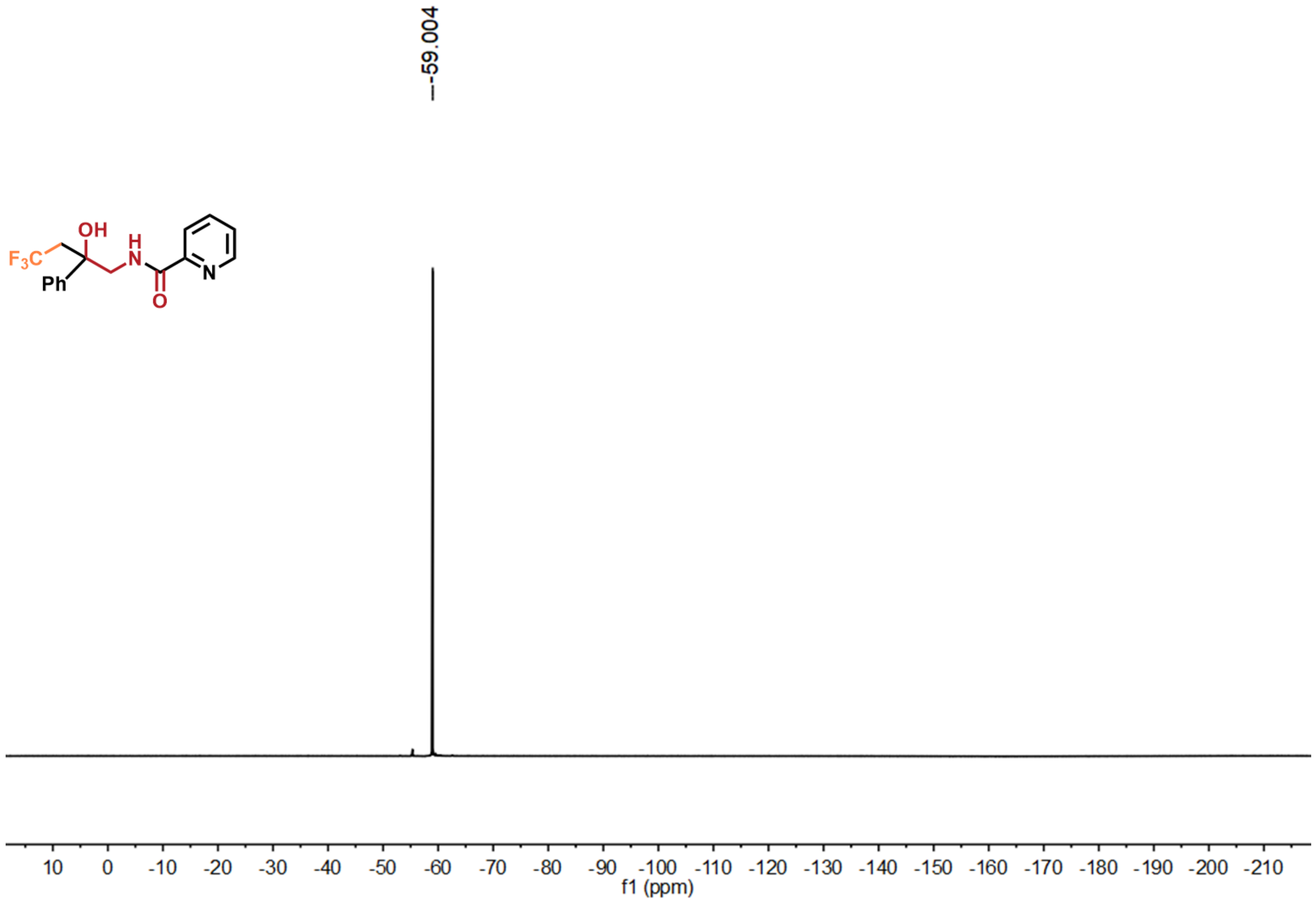

^{1}H NMR (400 MHz, $CDCl_3$) spectrum of **5k**

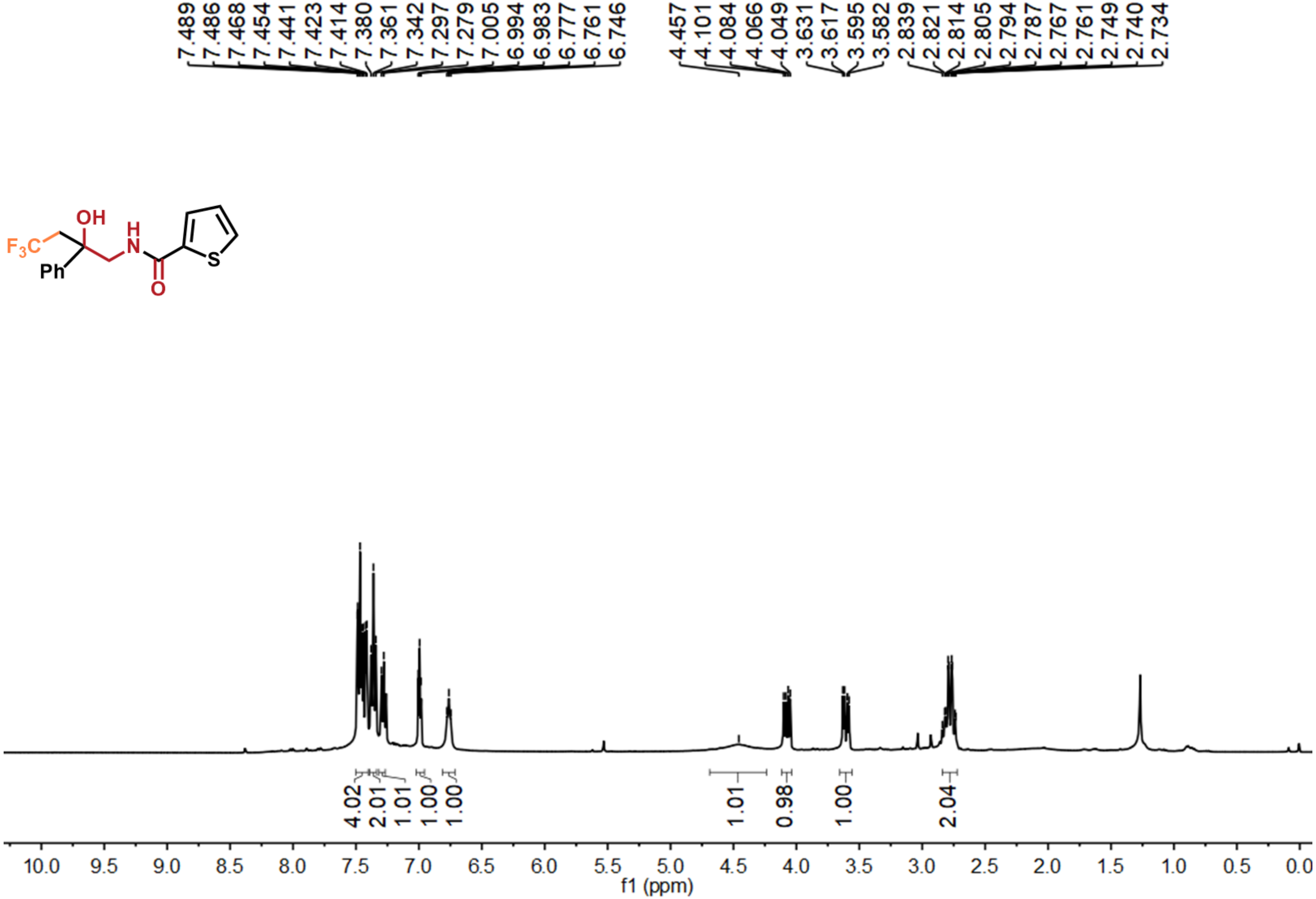

^{13}C NMR (100 MHz, $CDCl_3$) spectrum of **5k**

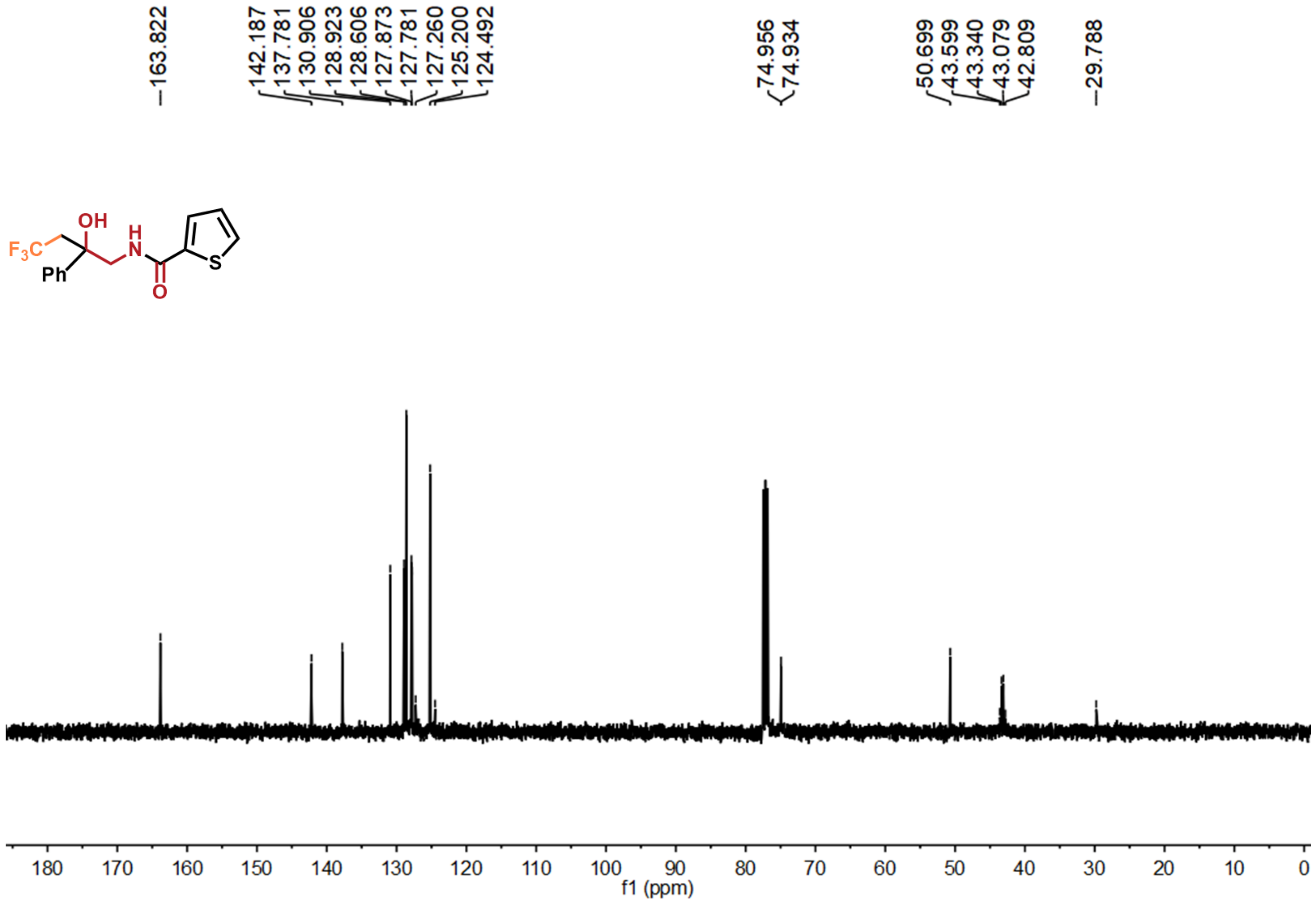

^{19}F NMR (376 MHz, $CDCl_3$) spectrum of **5k**

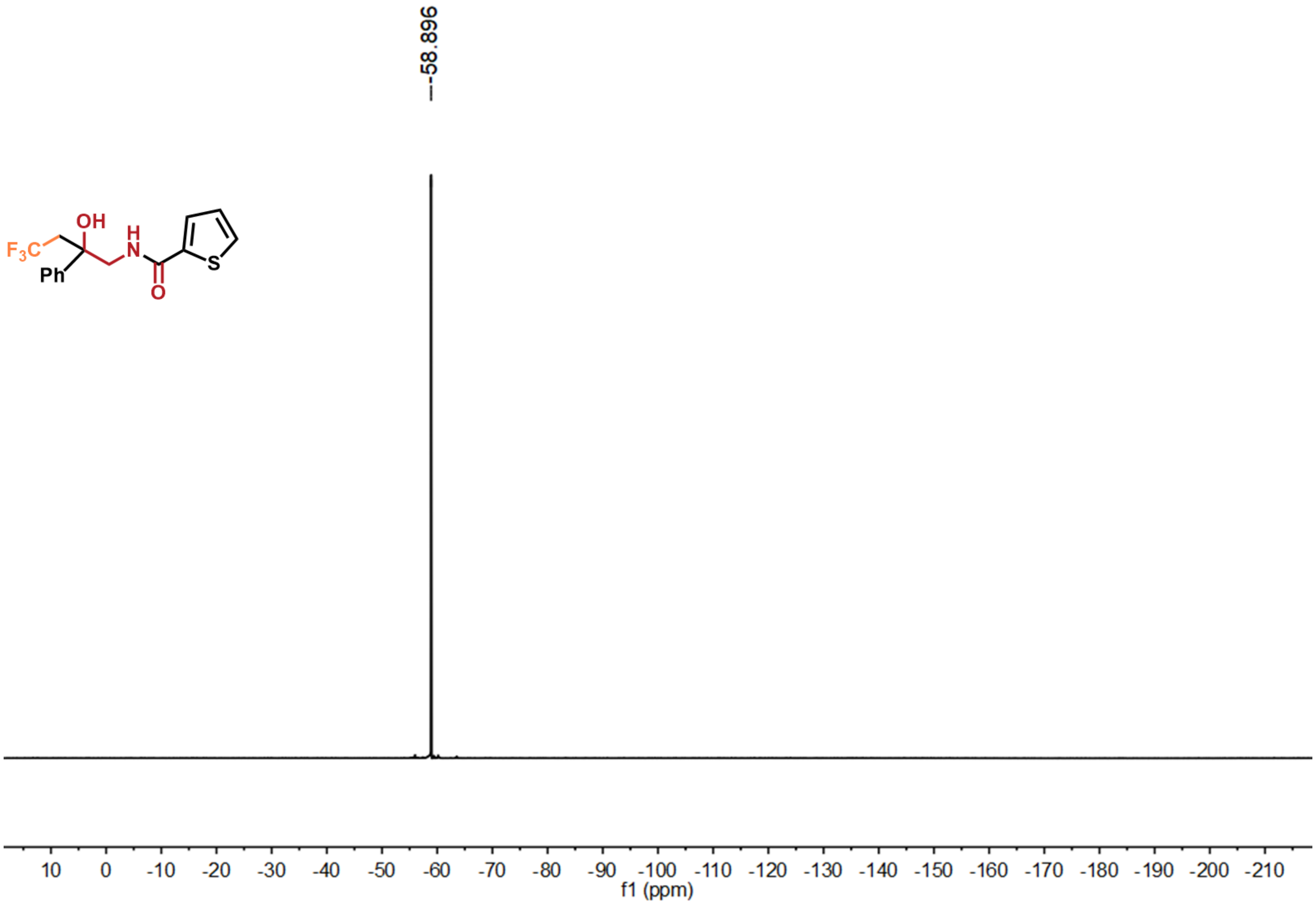

^{1}H NMR (400 MHz, $CDCl_3$) spectrum of **5l**

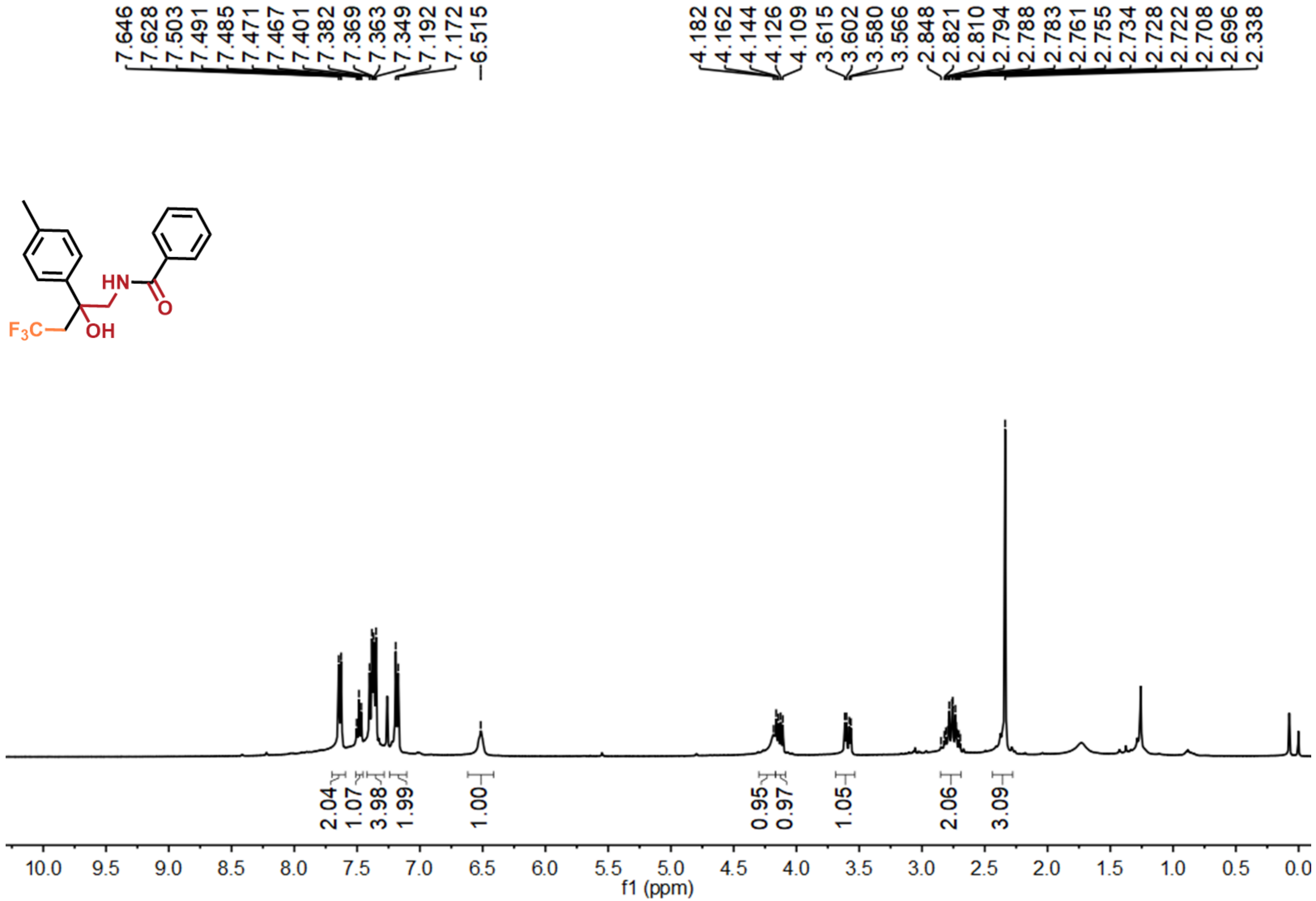

^{13}C NMR (100 MHz, $CDCl_3$) spectrum of **5l**

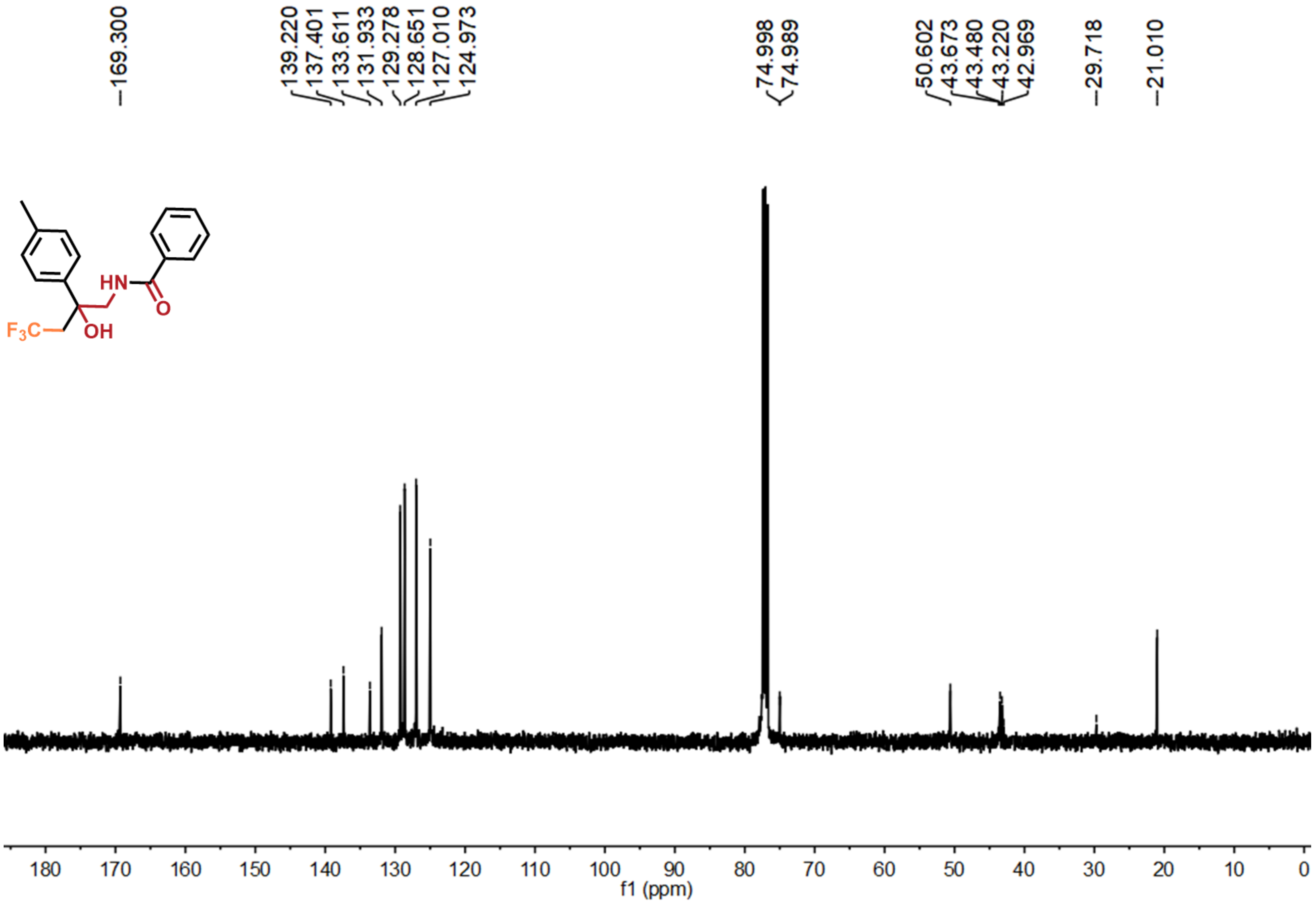

^{19}F NMR (376 MHz, $CDCl_3$) spectrum of **5l**

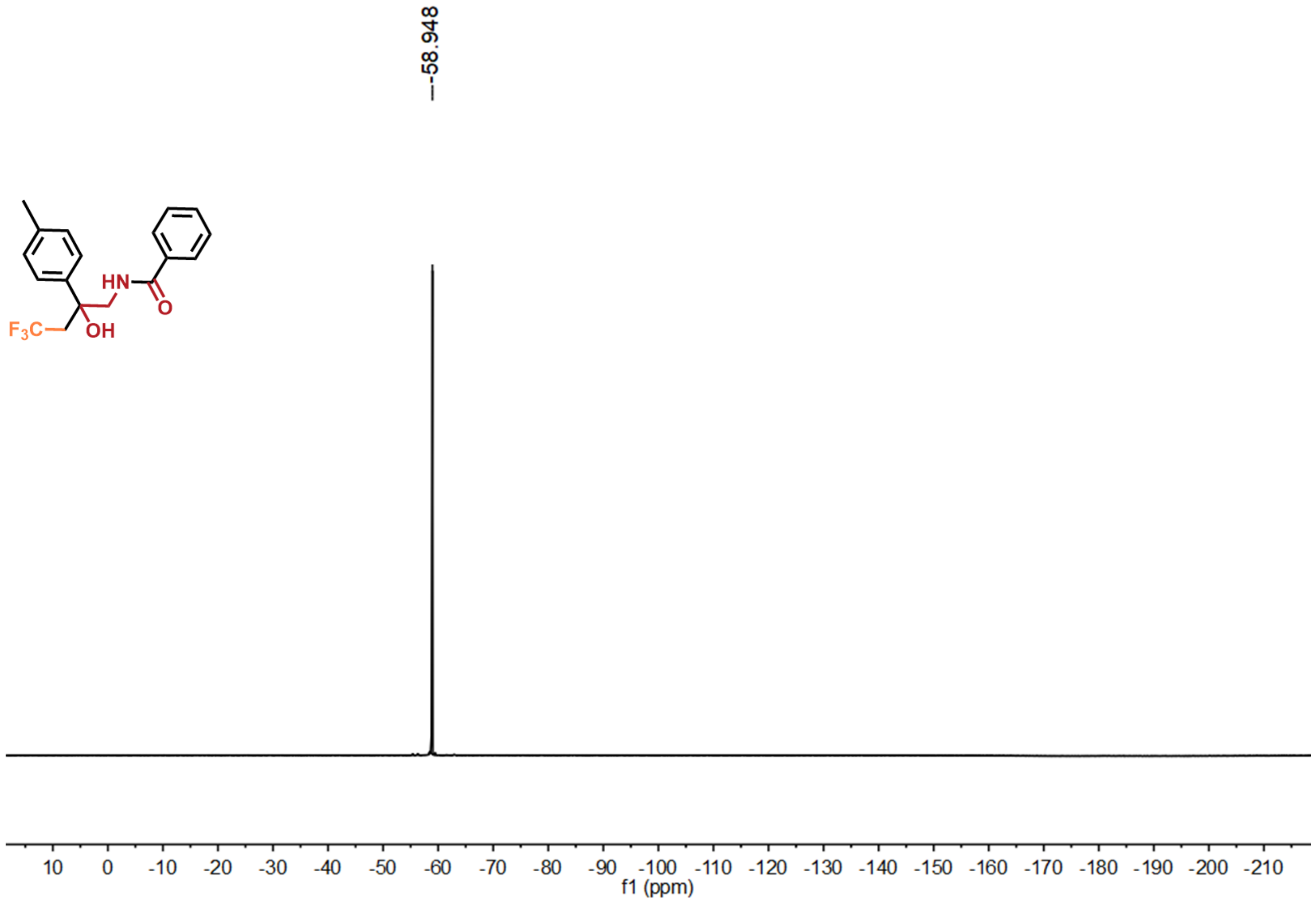

^{1}H NMR (400 MHz, $CDCl_3$) spectrum of **5m**

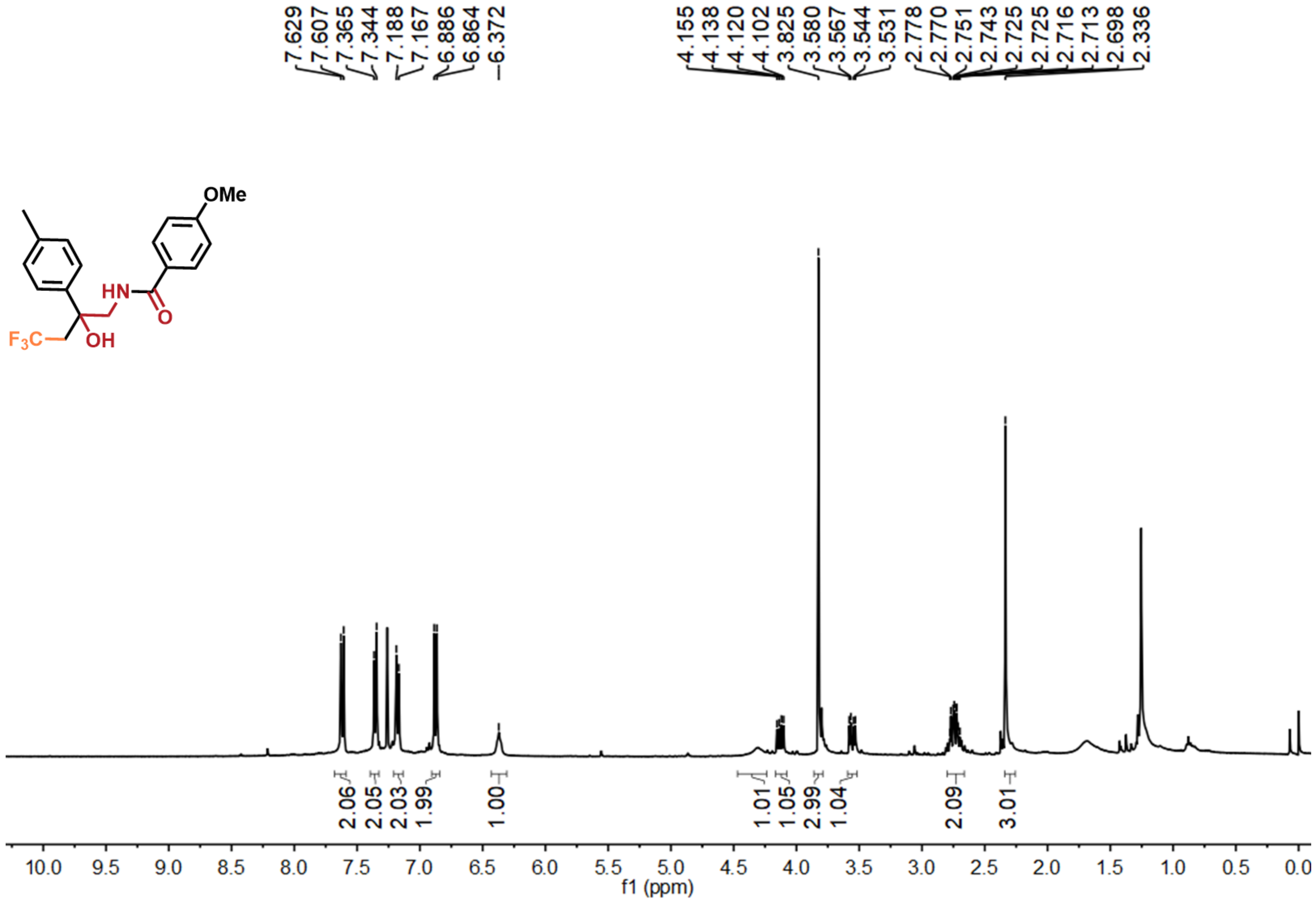

^{13}C NMR (100 MHz, $CDCl_3$) spectrum of **5m**

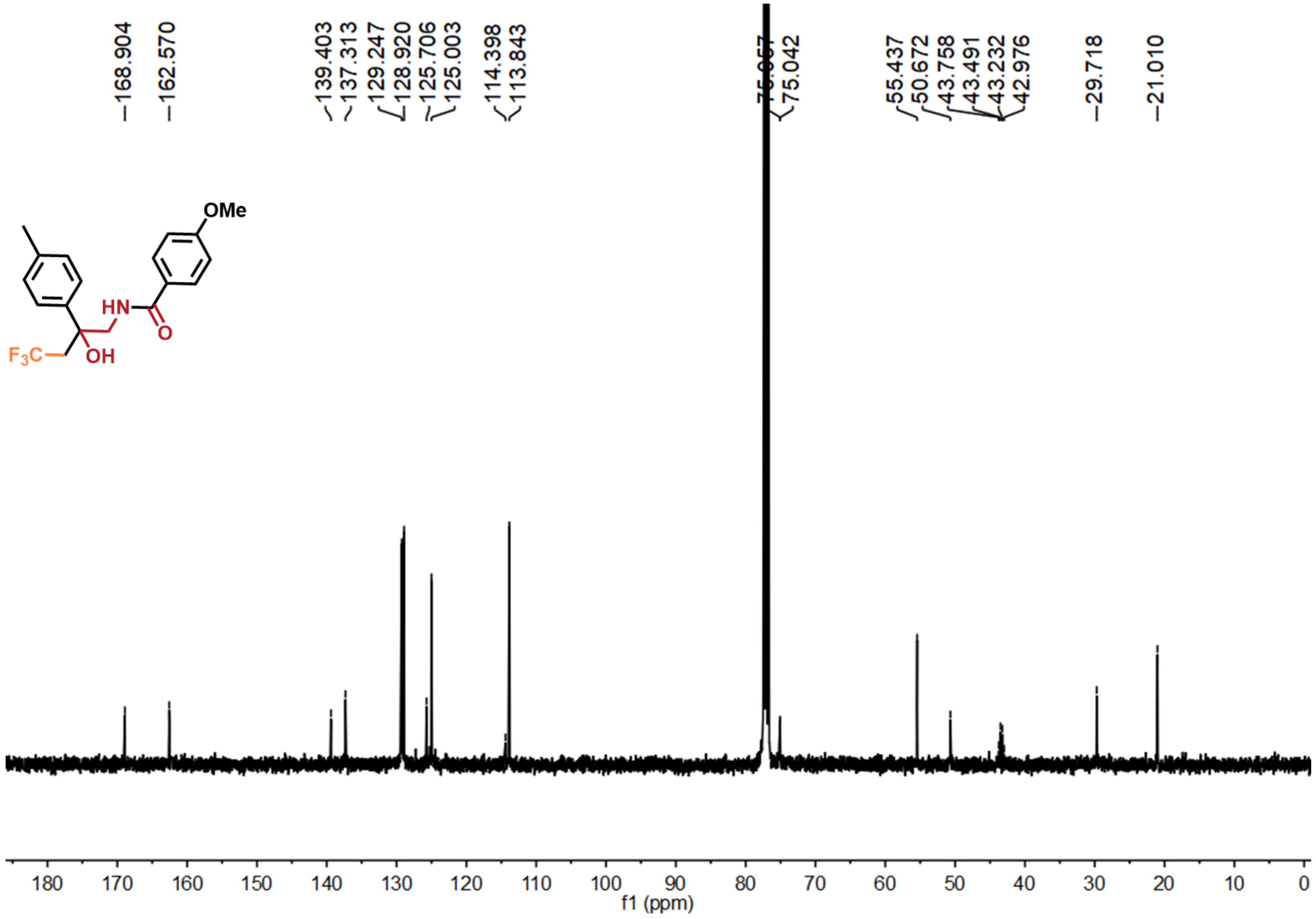

^{19}F NMR (376 MHz, $CDCl_3$) spectrum of **5m**

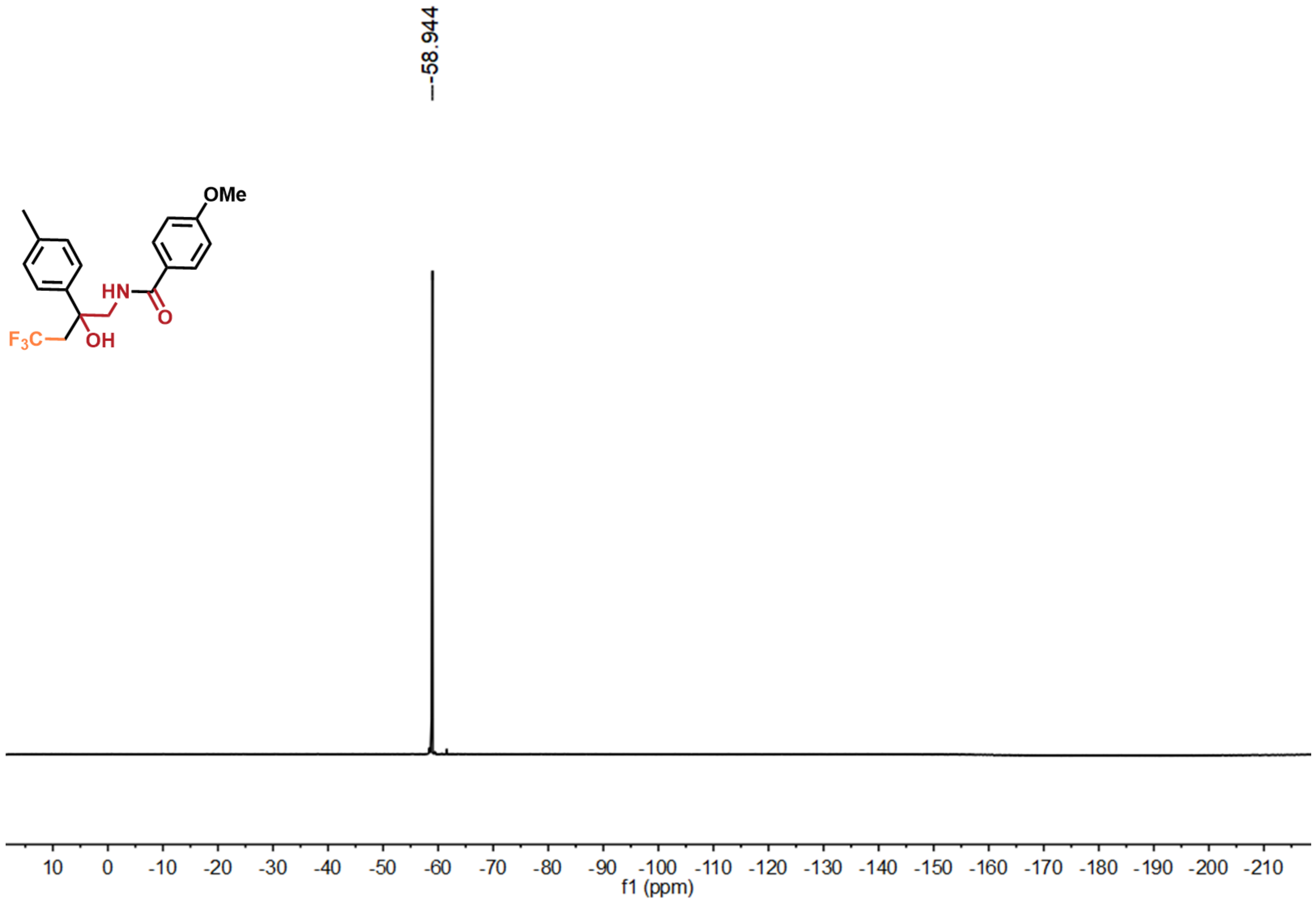

^{1}H NMR (400 MHz, $CDCl_3$) spectrum of **5n**

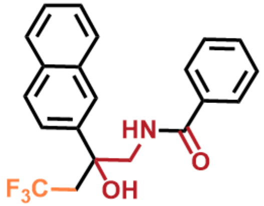

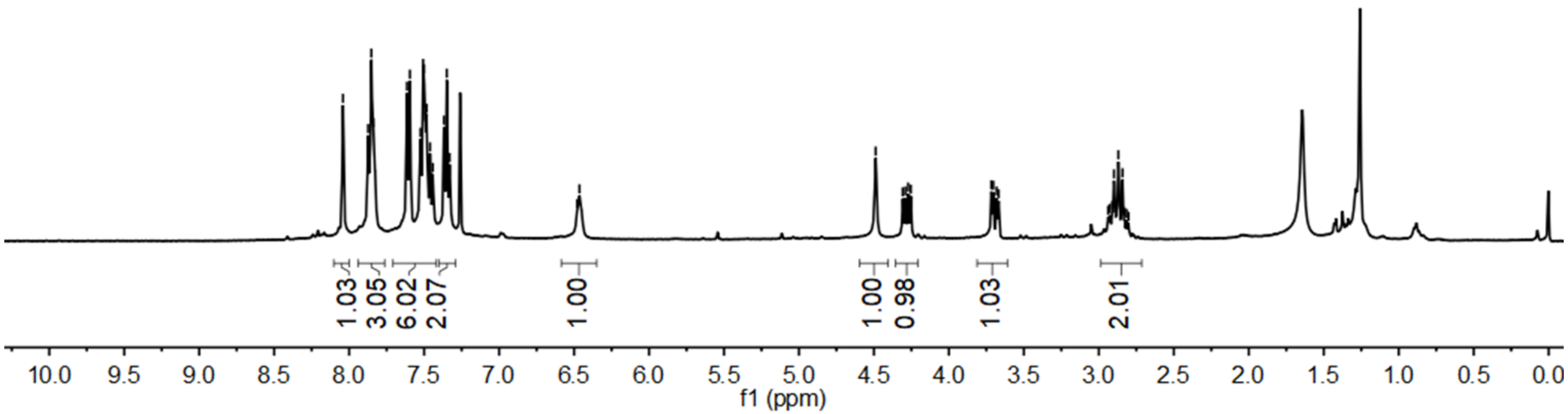

^{13}C NMR (100 MHz, $CDCl_3$) spectrum of **5n**

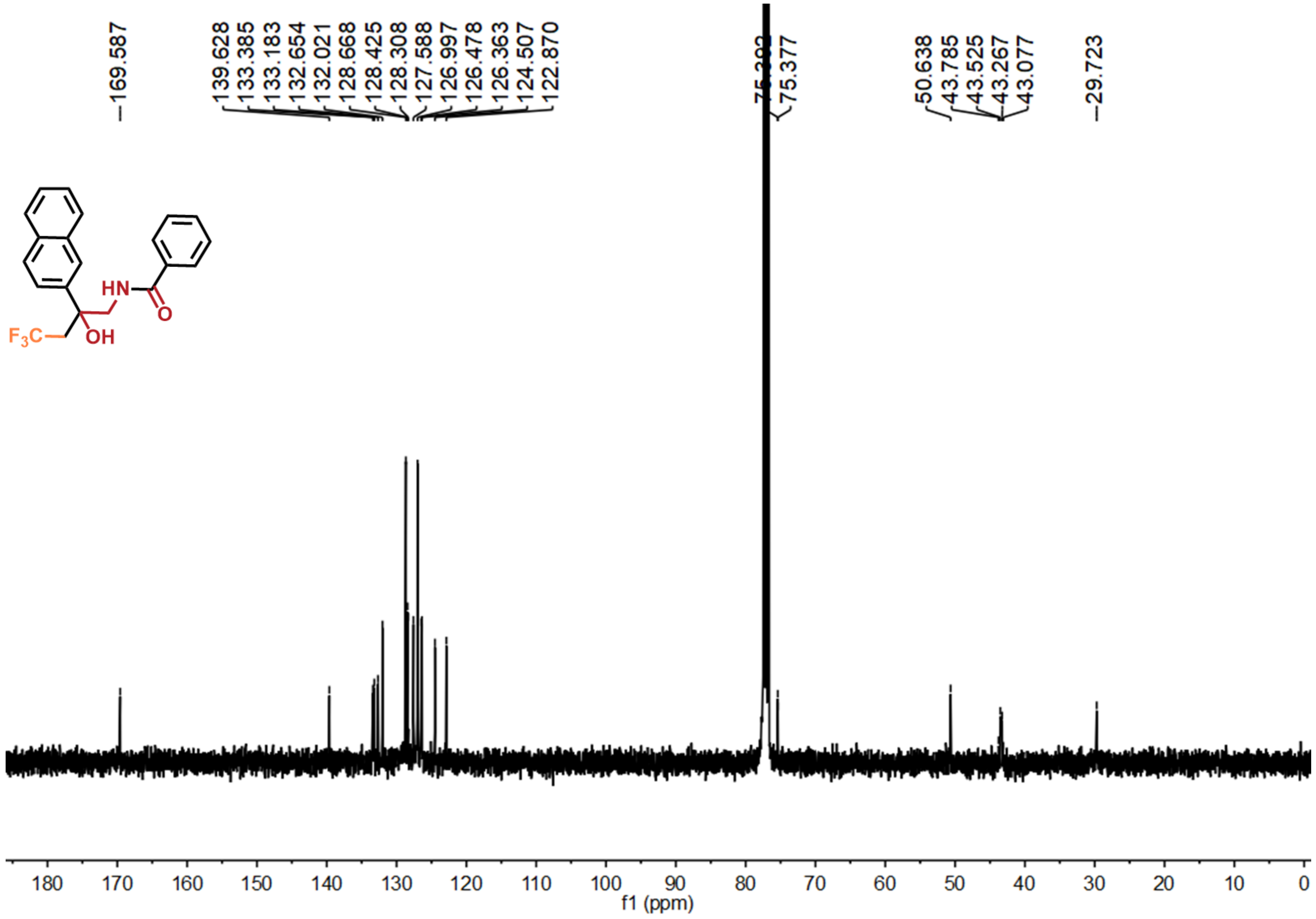

^{19}F NMR (376 MHz, $CDCl_3$) spectrum of **5n**

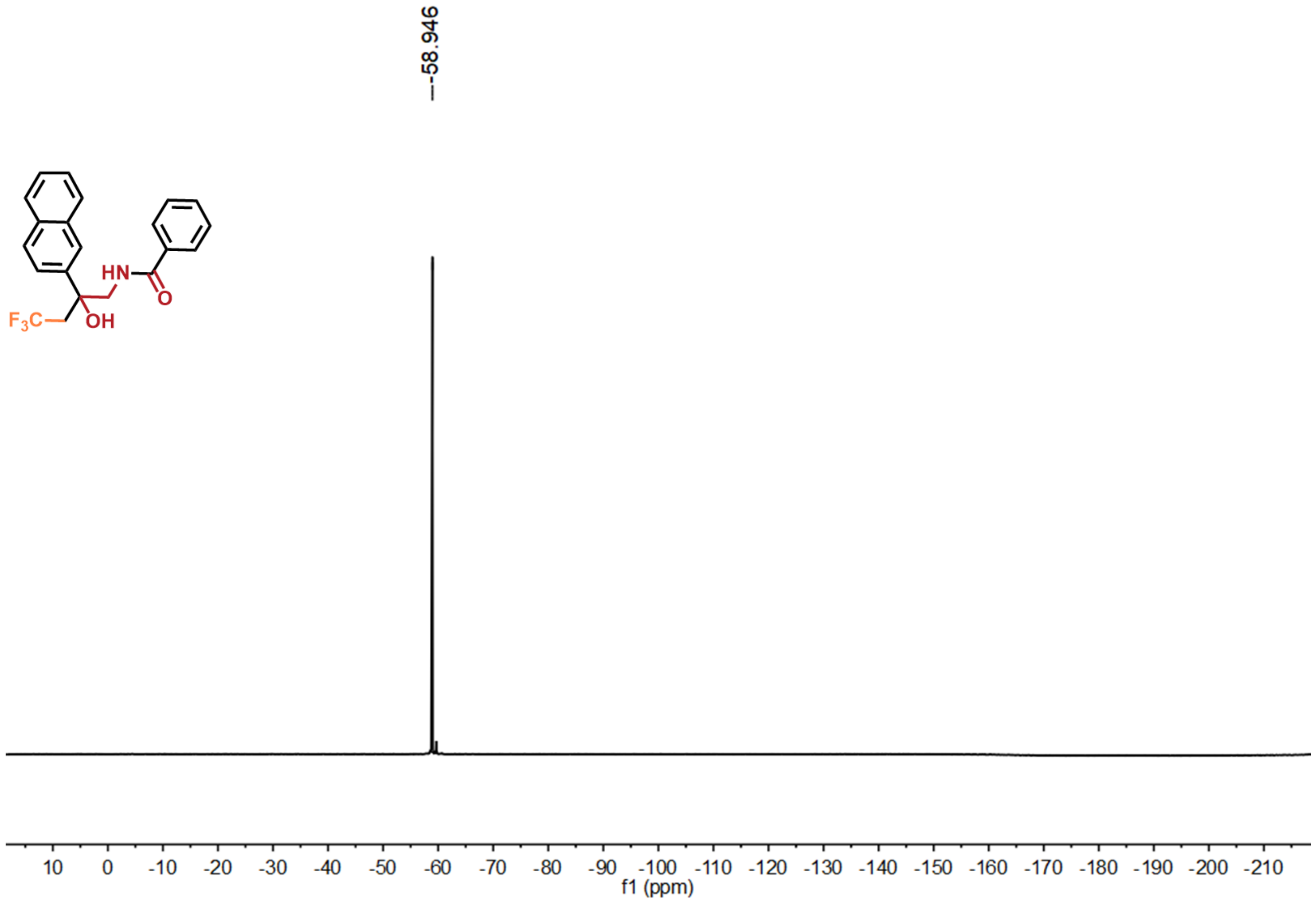

^{1}H NMR (400 MHz, $CDCl_3$) spectrum of **5o**

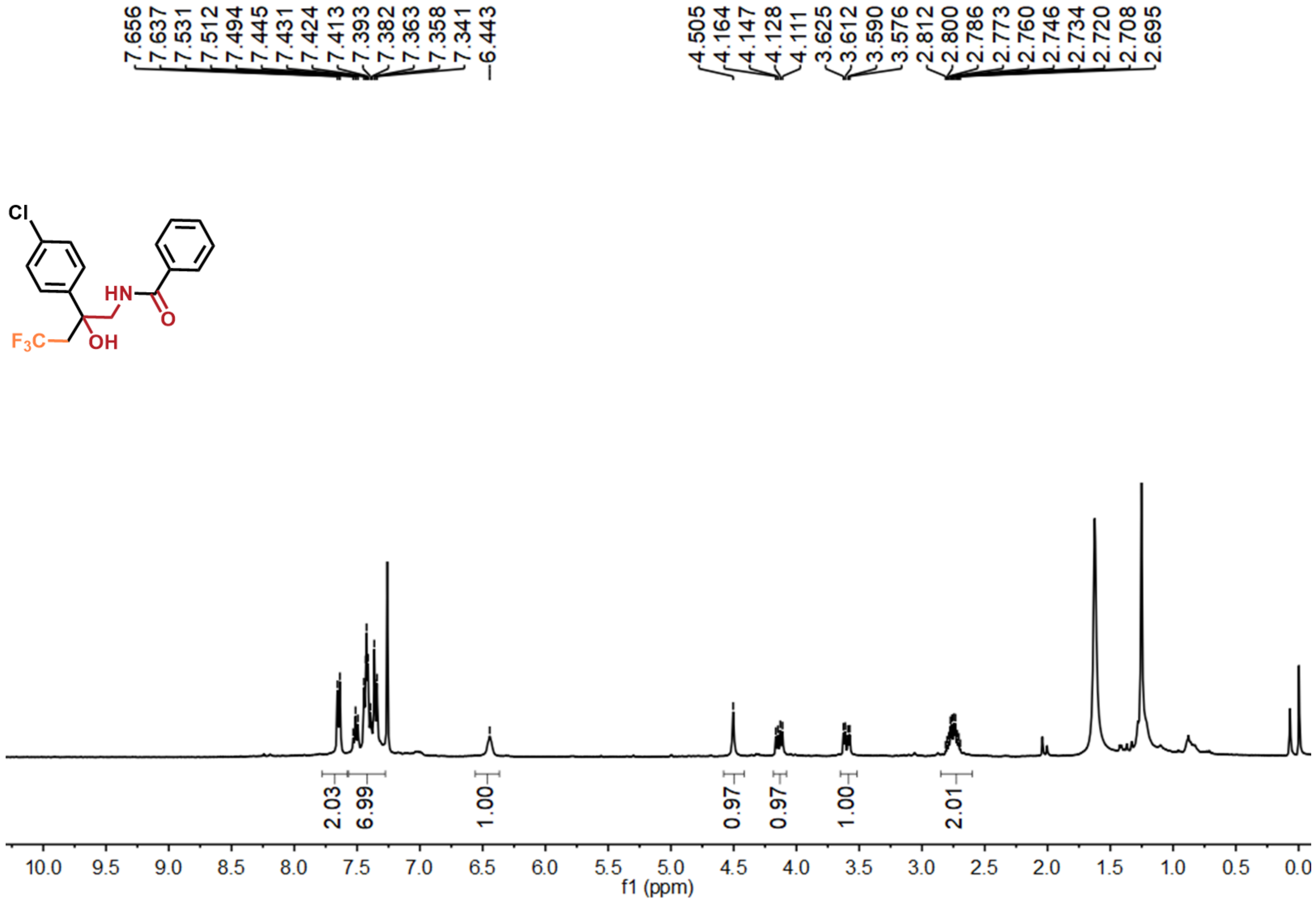

^{13}C NMR (100 MHz, $CDCl_3$) spectrum of **5o**

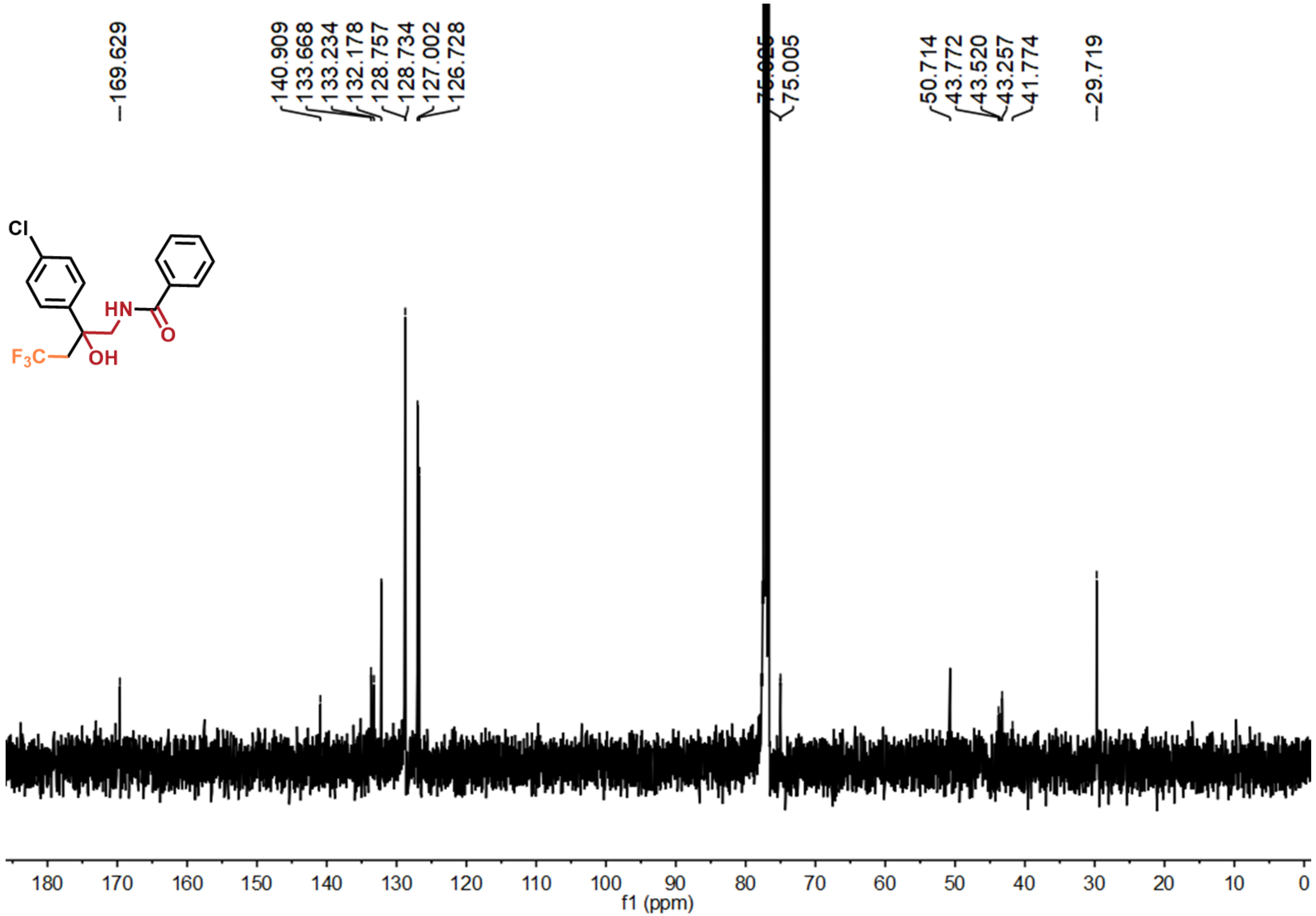

^{19}F NMR (376 MHz, $CDCl_3$) spectrum of **5o**

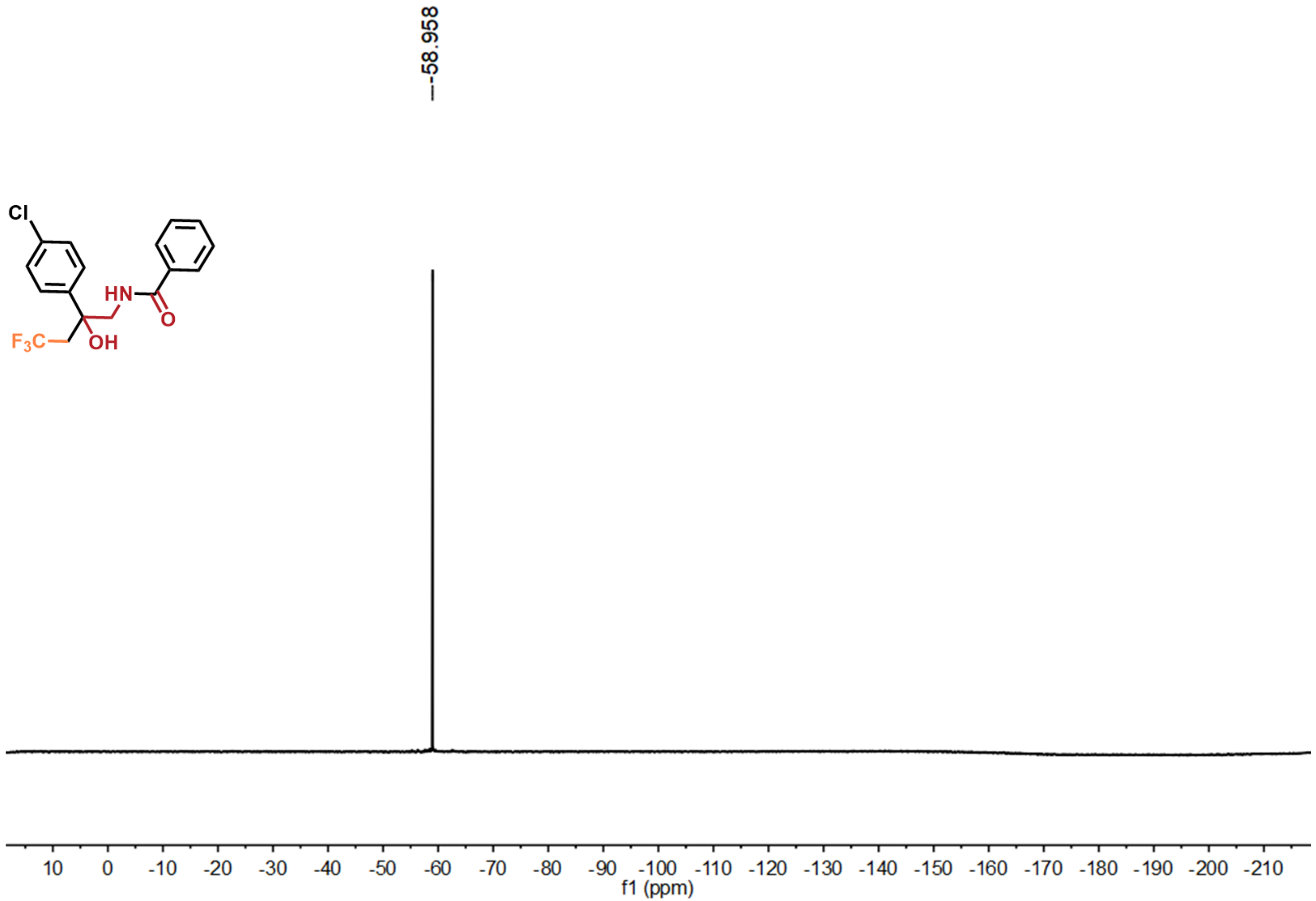

^{1}H NMR (400 MHz, $CDCl_3$) spectrum of **5p**

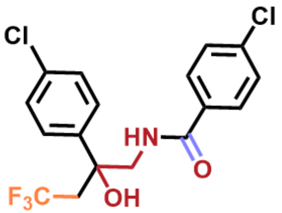

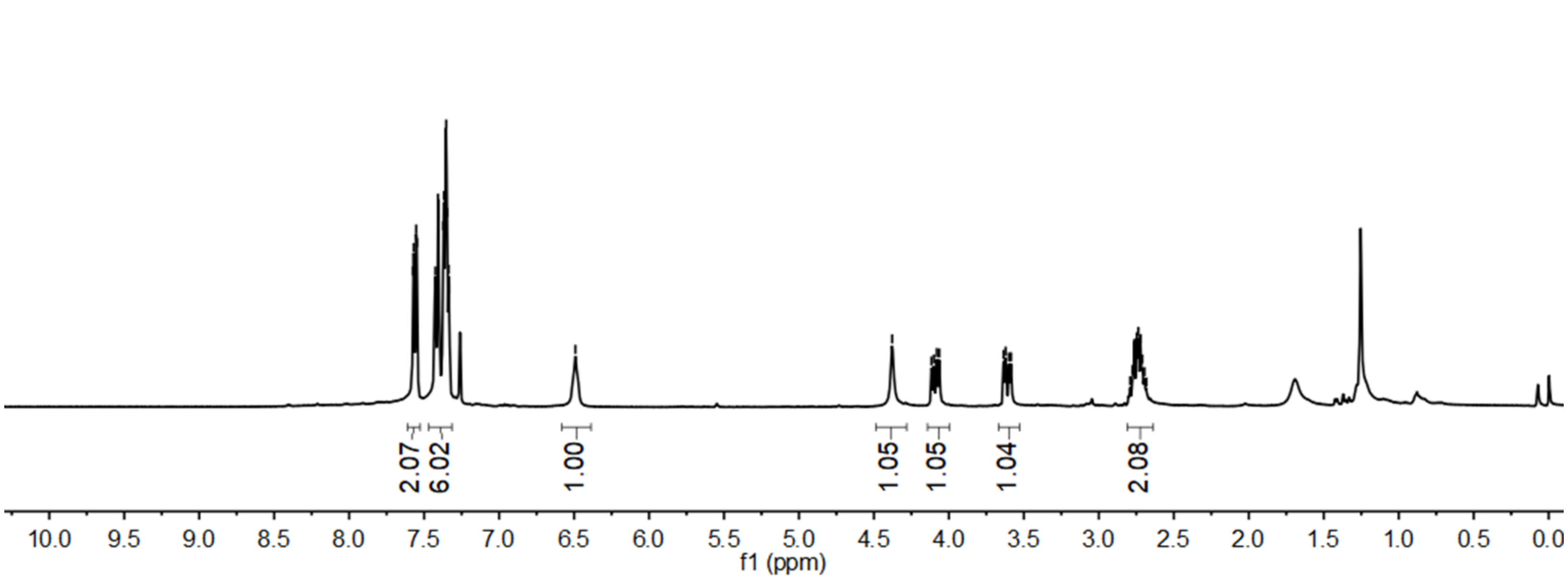

^{13}C NMR (100 MHz, $CDCl_3$) spectrum of **5p**

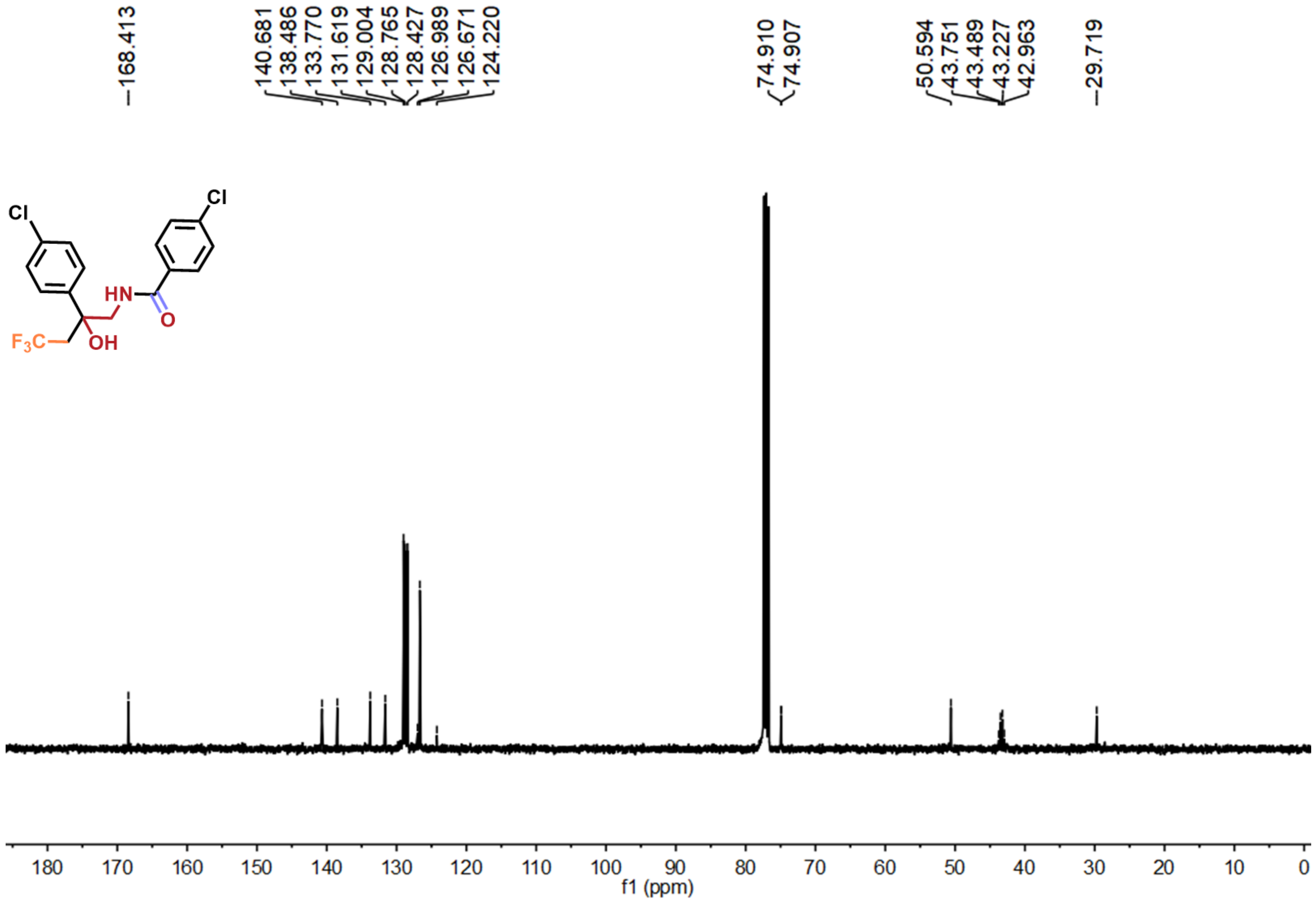

^{19}F NMR (376 MHz, $CDCl_3$) spectrum of **5p**

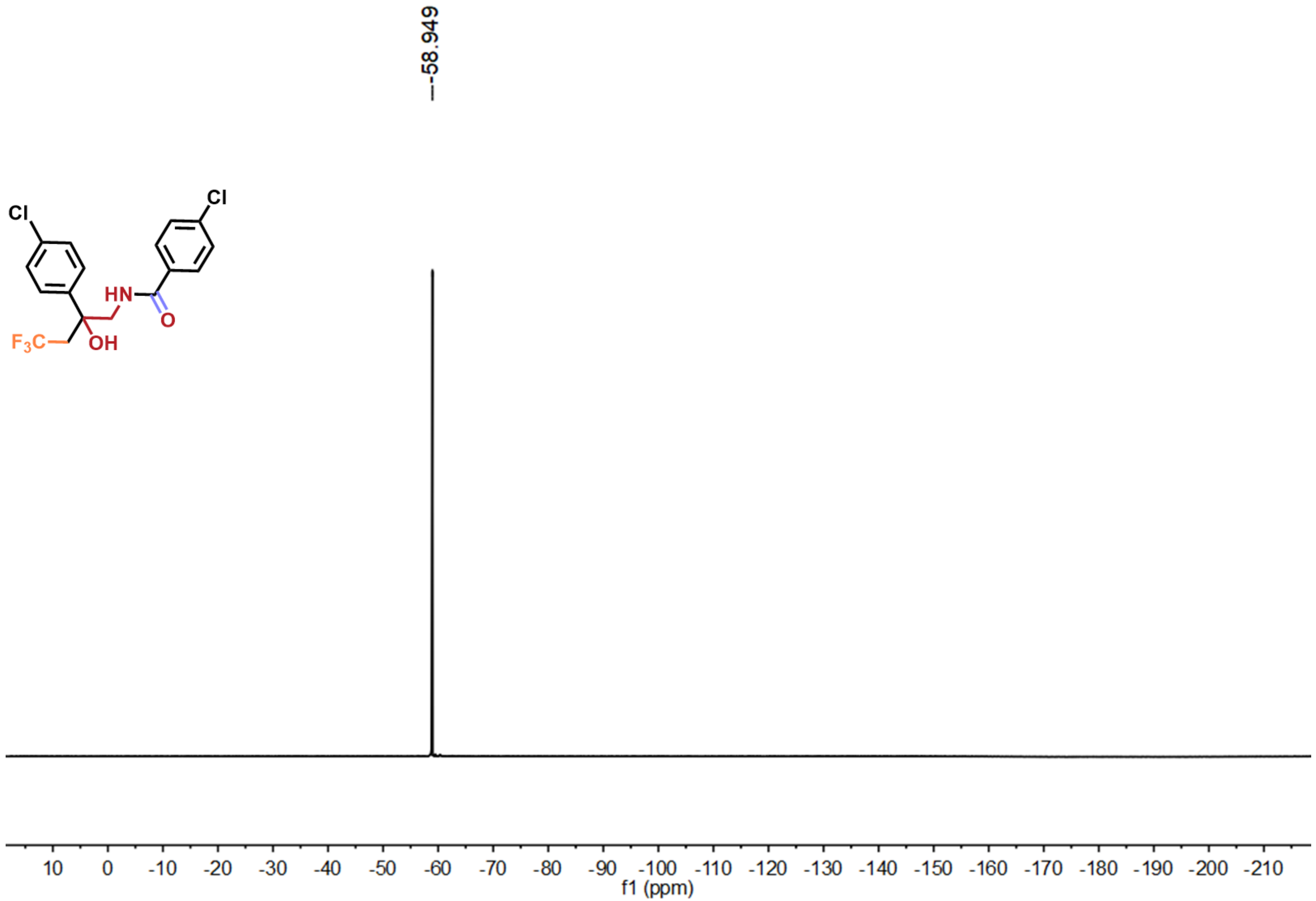

^{1}H NMR (400 MHz, $CDCl_3$) spectrum of **5q**

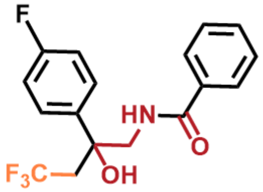

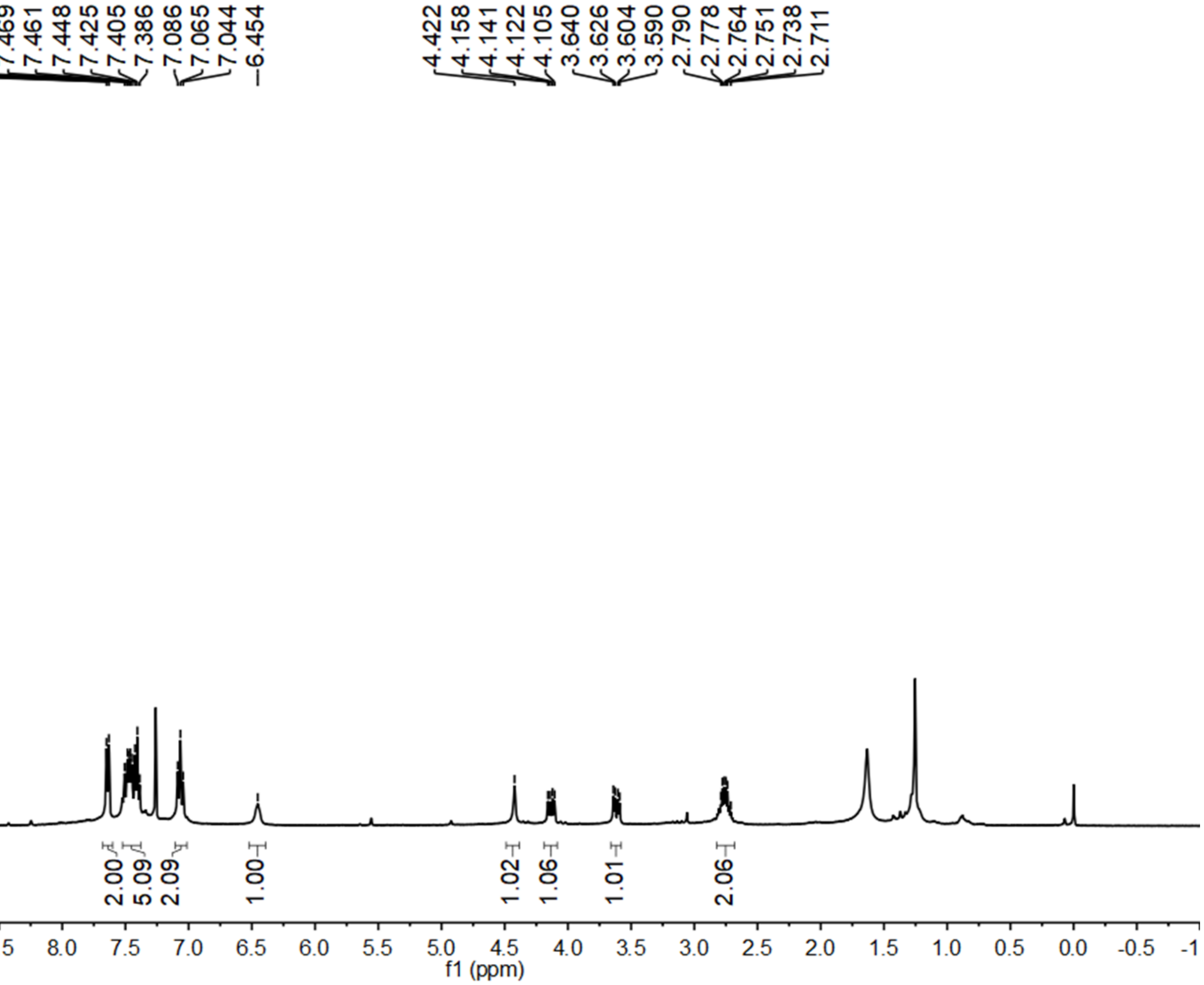

^{13}C NMR (100 MHz, $CDCl_3$) spectrum of **5q**

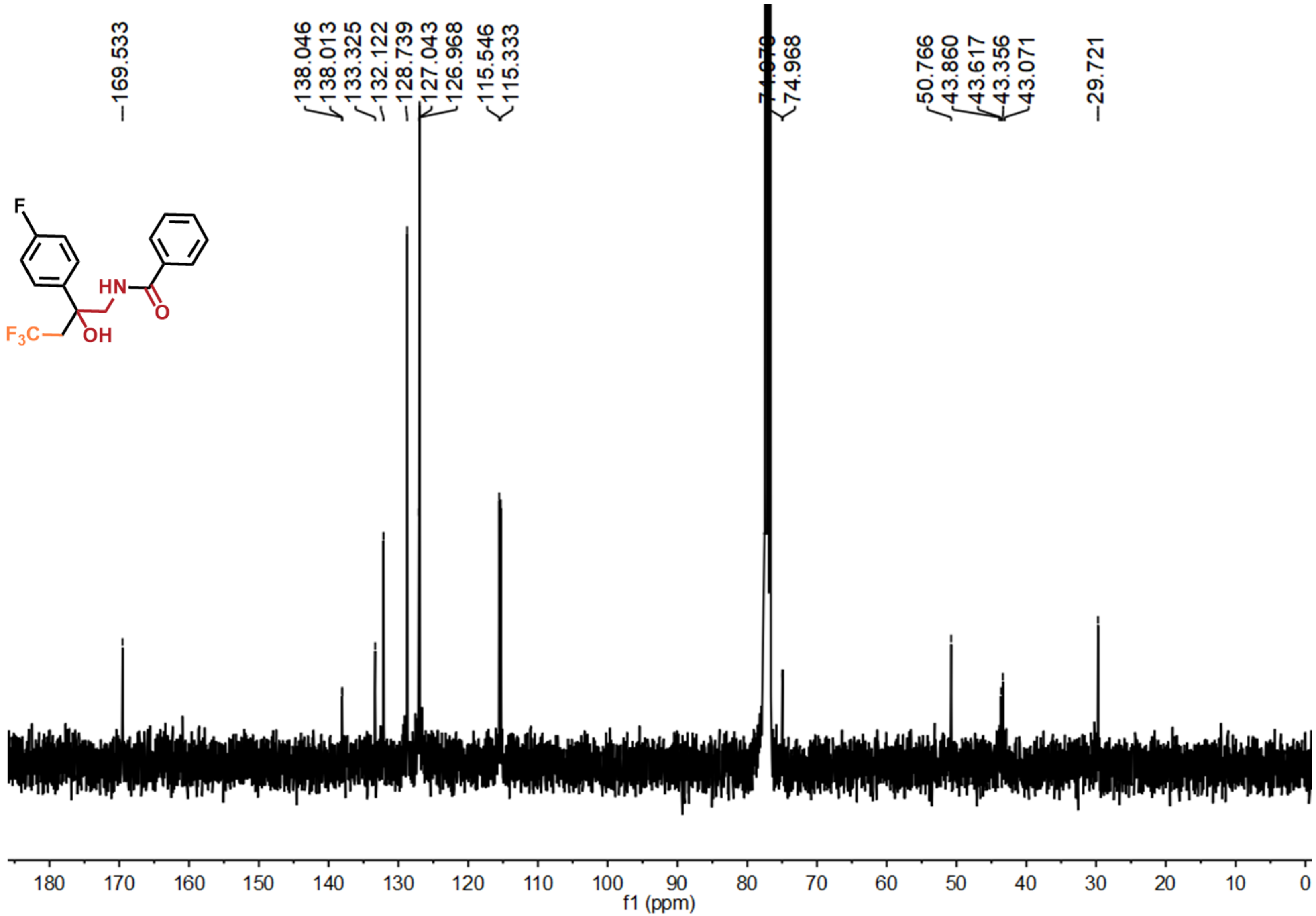

^{19}F NMR (376 MHz, $CDCl_3$) spectrum of **5q**

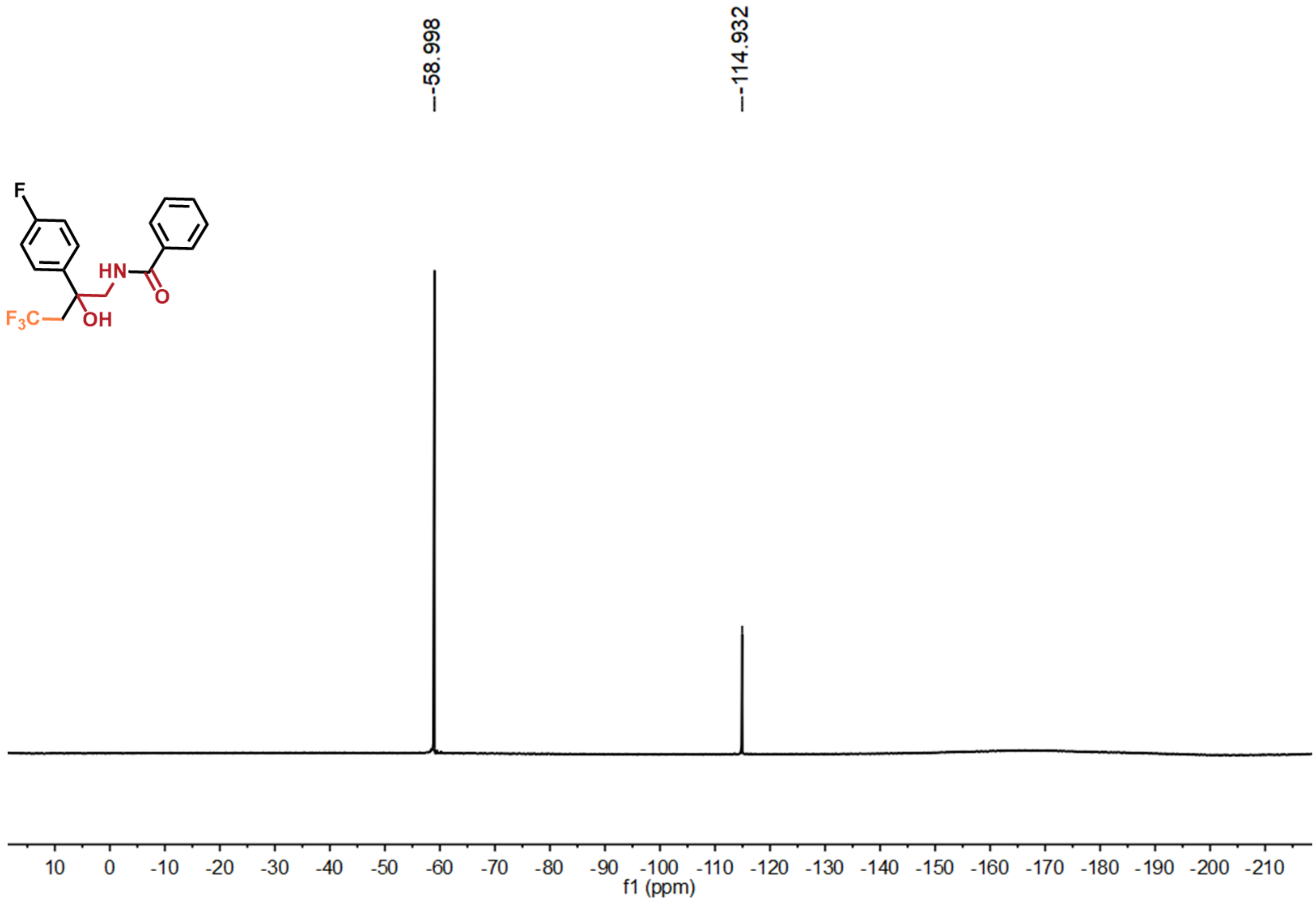

^{1}H NMR (400 MHz, $CDCl_3$) spectrum of **5r**

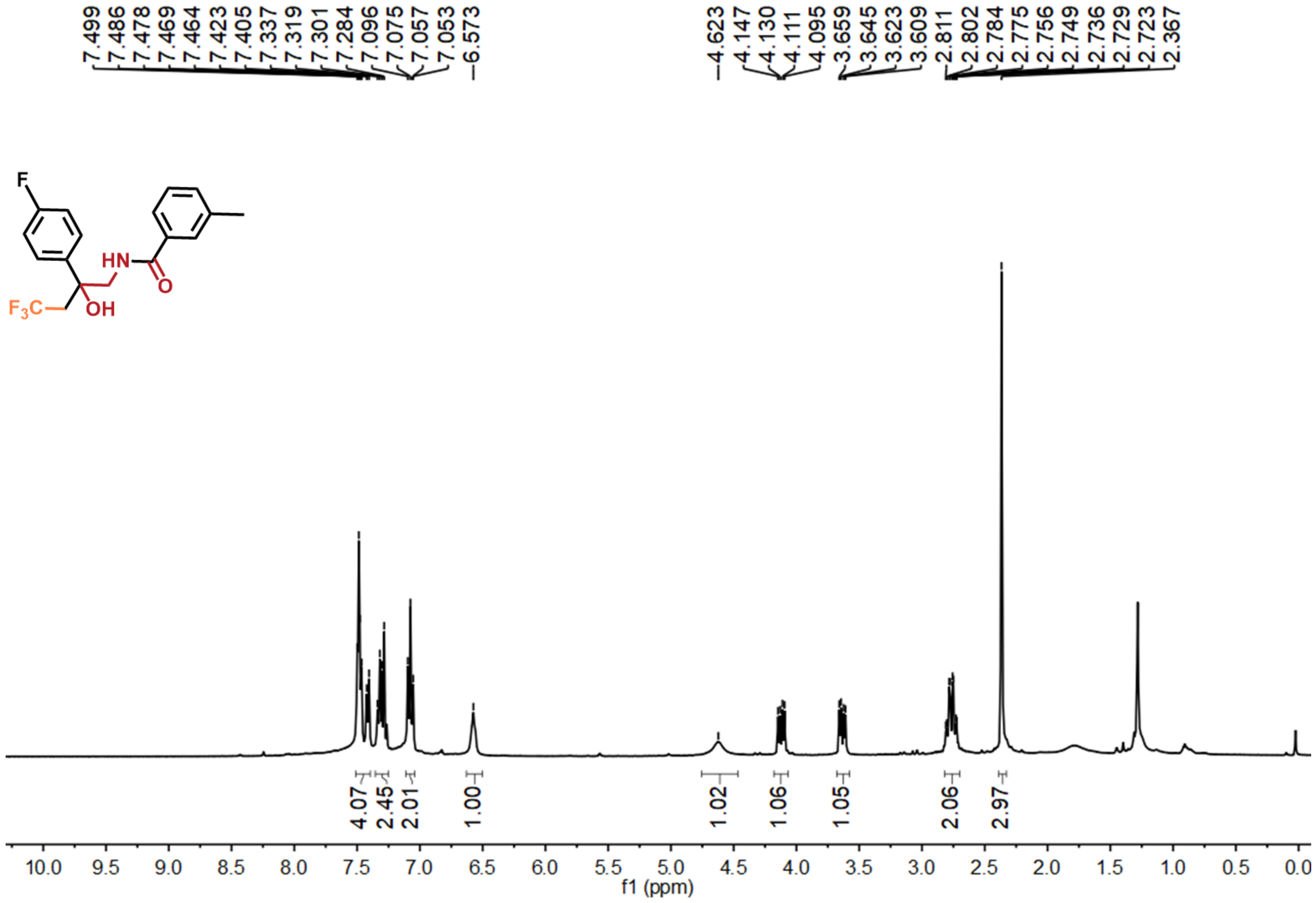

^{13}C NMR (100 MHz, $CDCl_3$) spectrum of **5r**

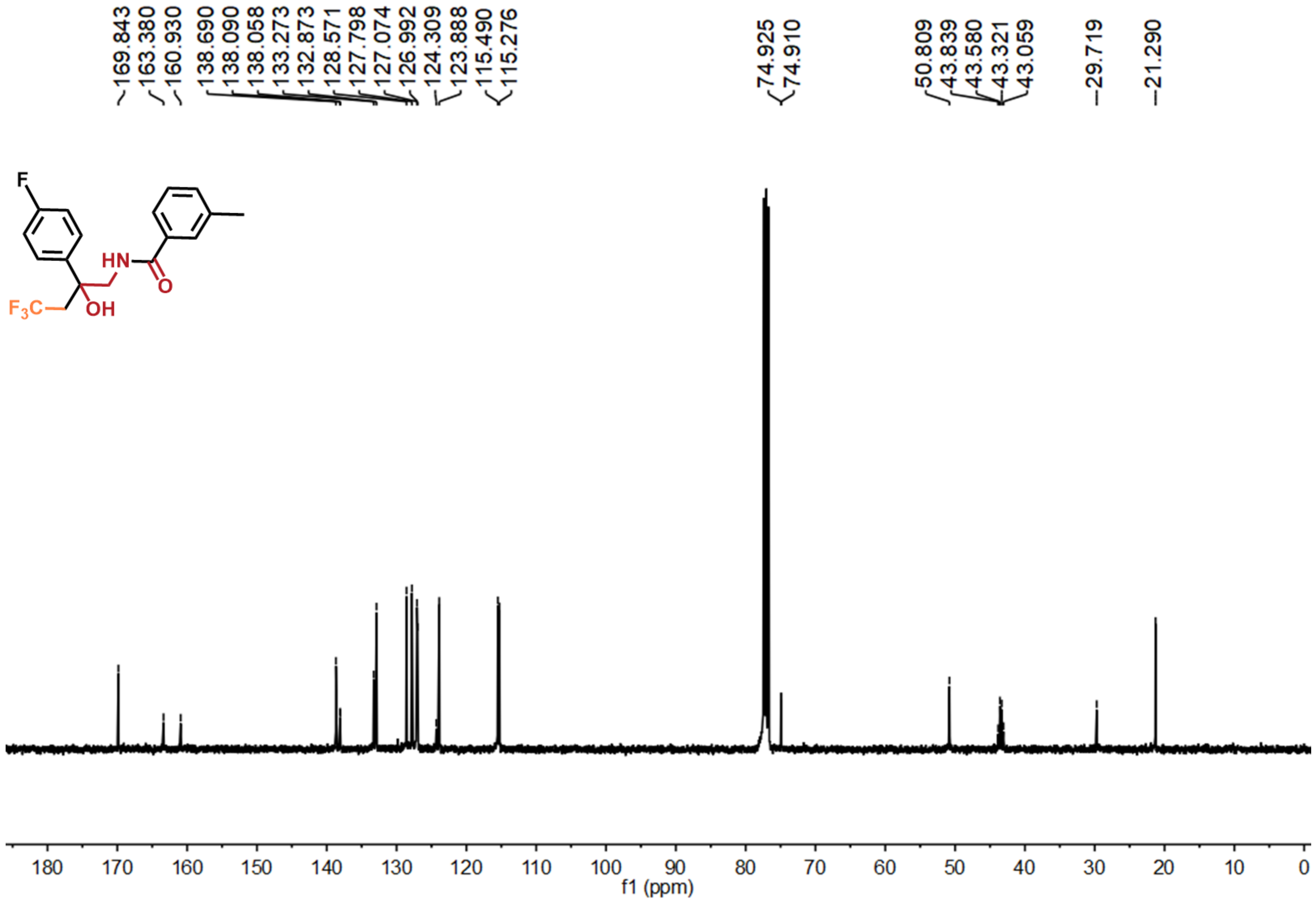

^{19}F NMR (376 MHz, $CDCl_3$) spectrum of **5r**

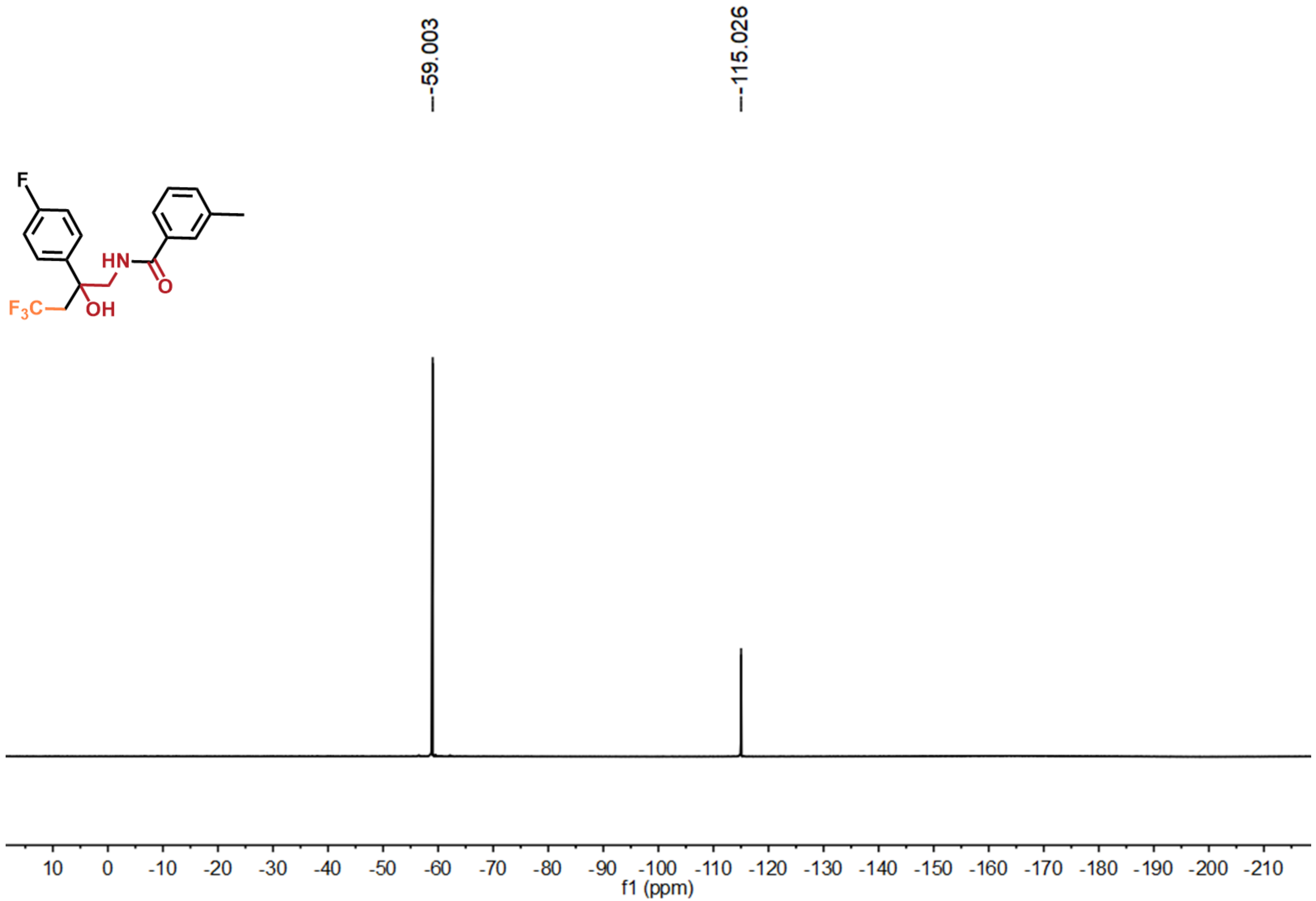

^{1}H NMR (400 MHz, $CDCl_3$) spectrum of **5a-re**

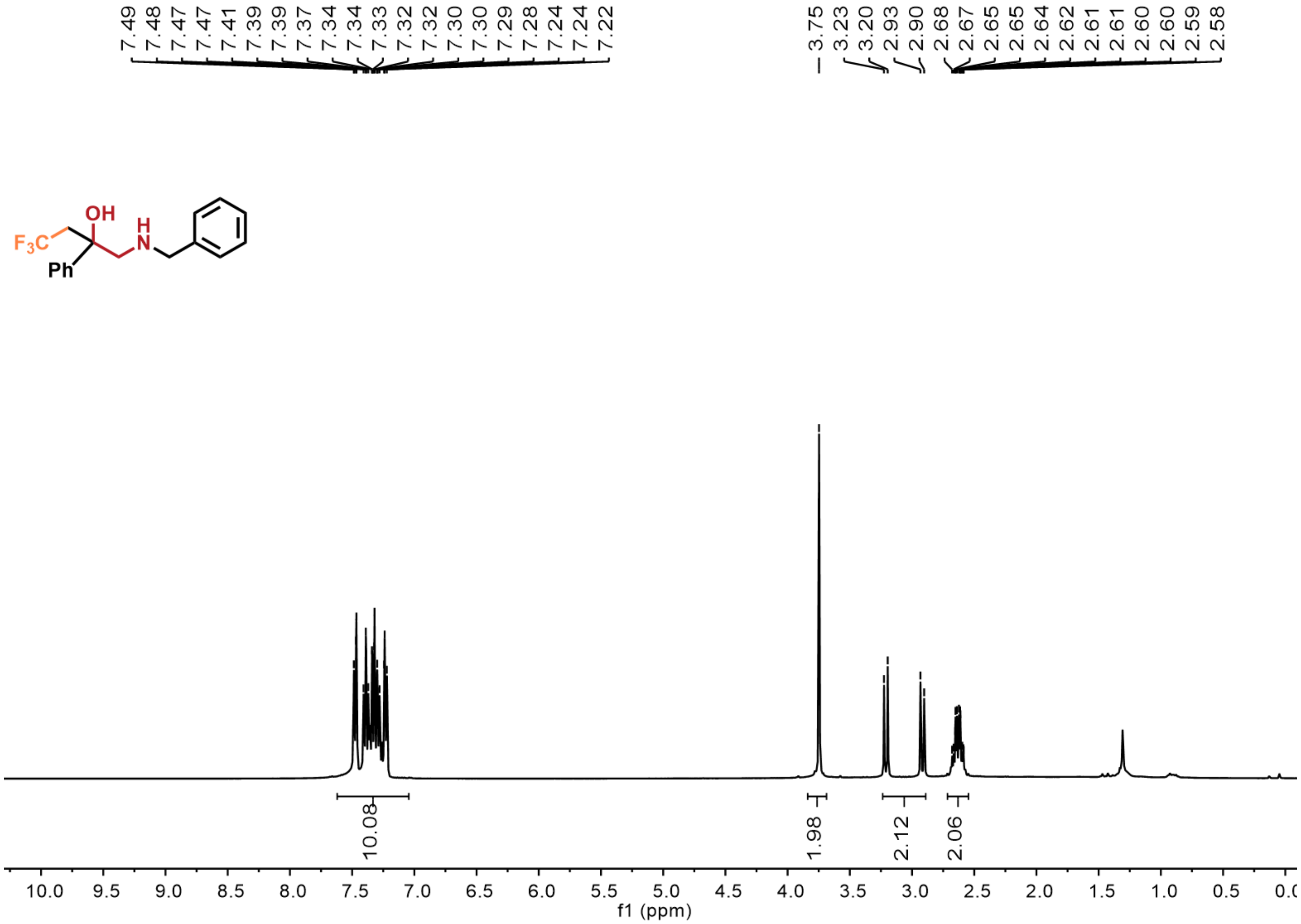

^{13}C NMR (100 MHz, $CDCl_3$) spectrum of **5a-re**

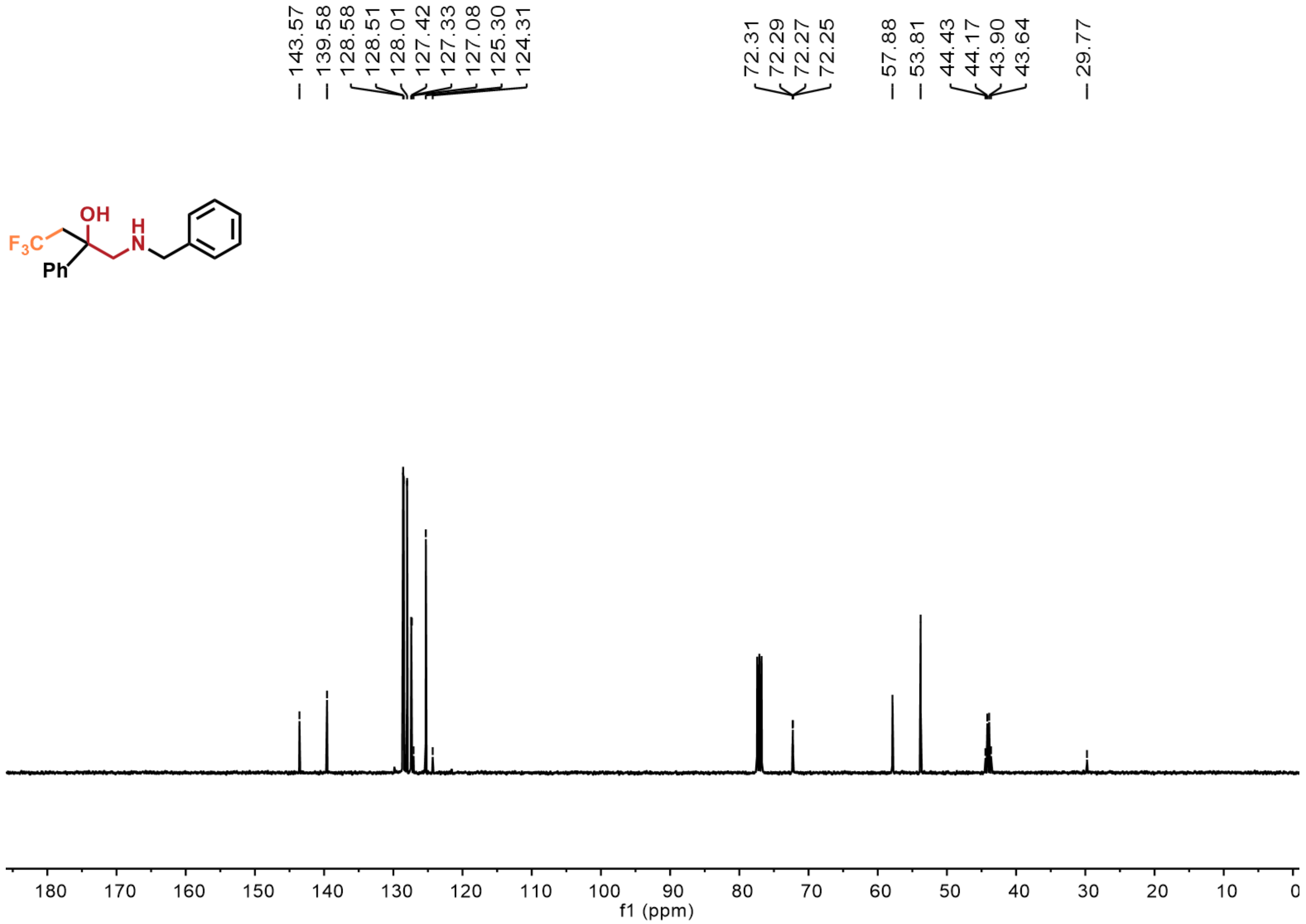

^{19}F NMR (376 MHz, $CDCl_3$) spectrum of **5a-re**

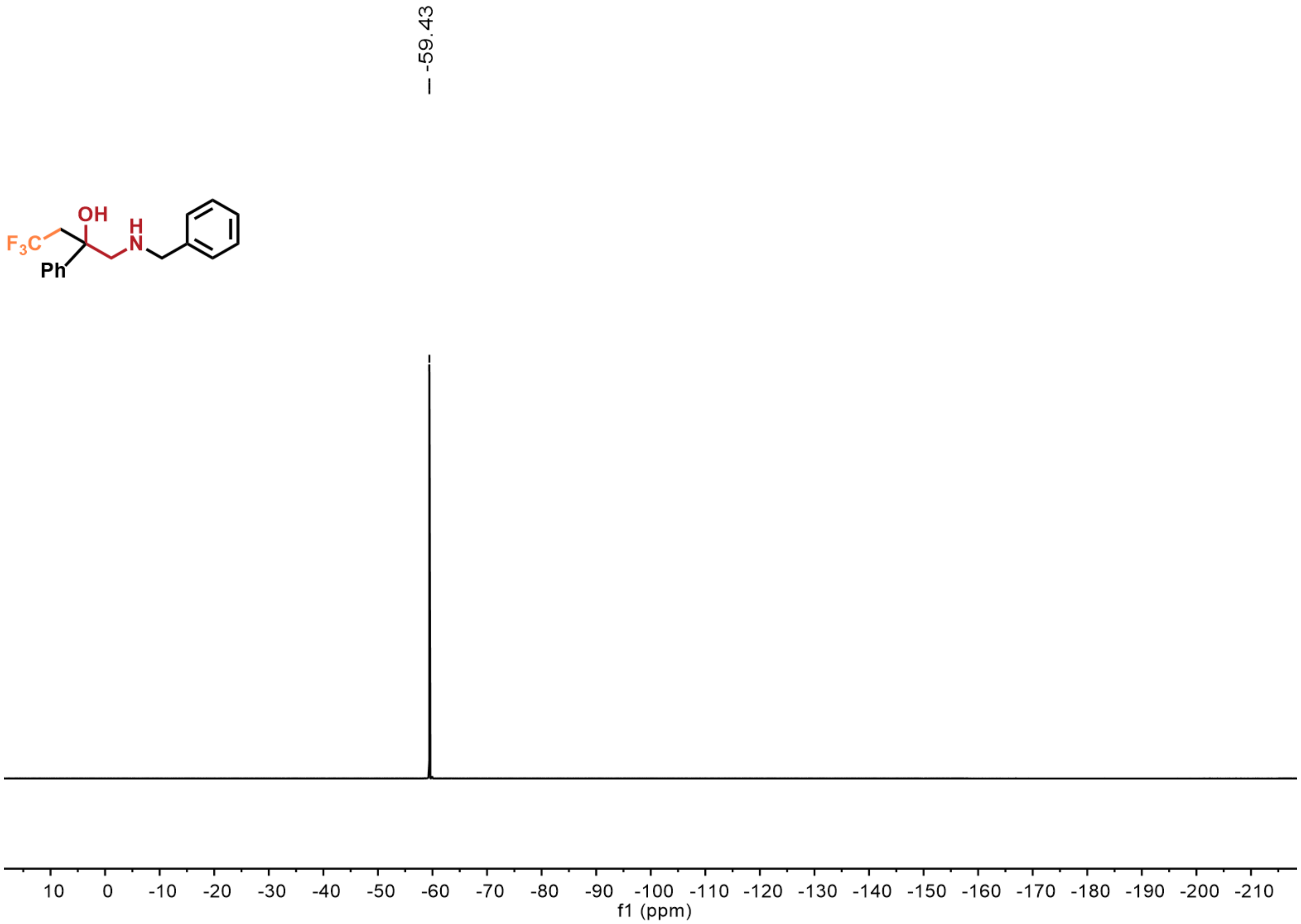

^{1}H NMR (400 MHz, $CDCl_3$) spectrum of **6a**

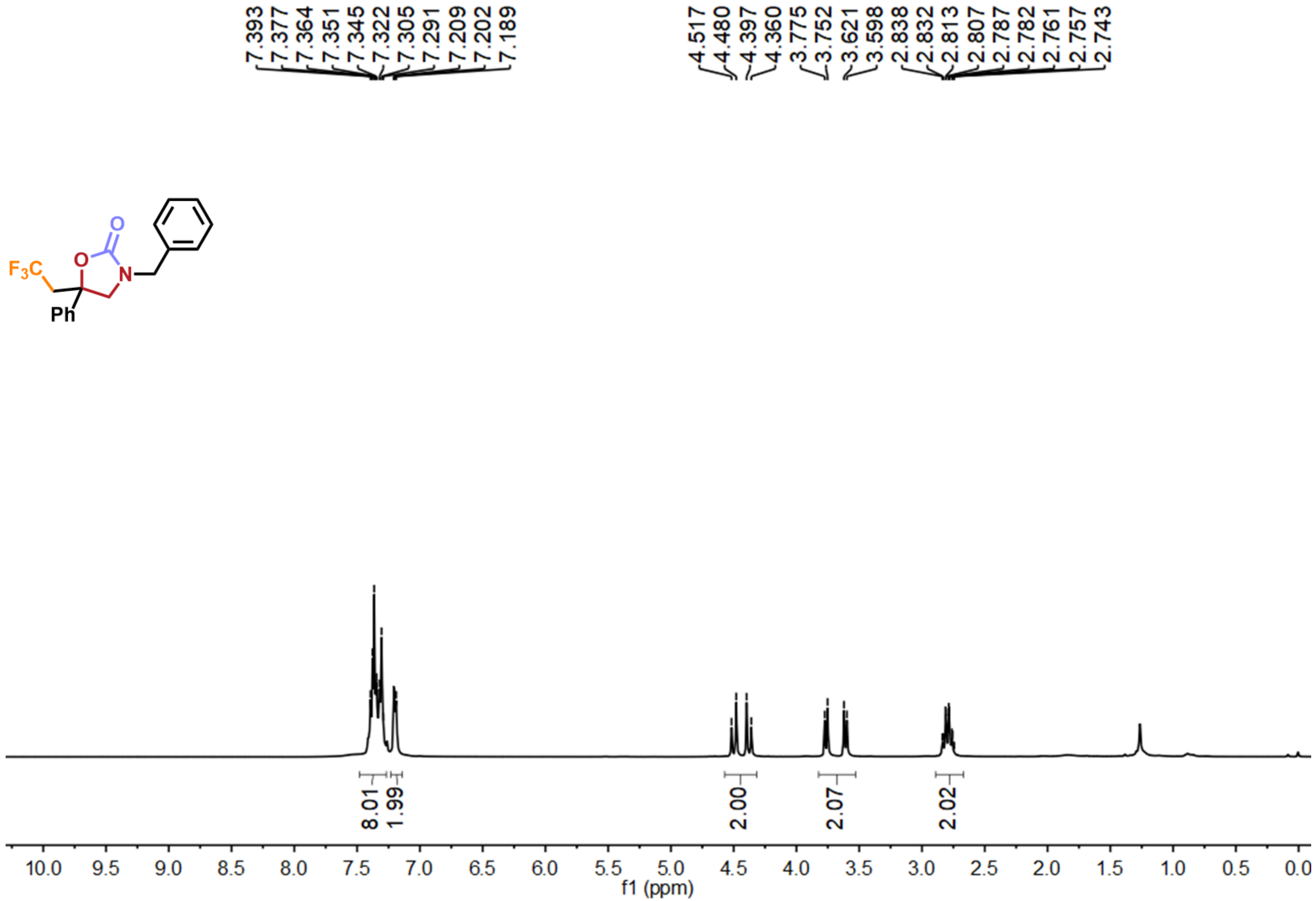

^{13}C NMR (100 MHz, $CDCl_3$) spectrum of **6a**

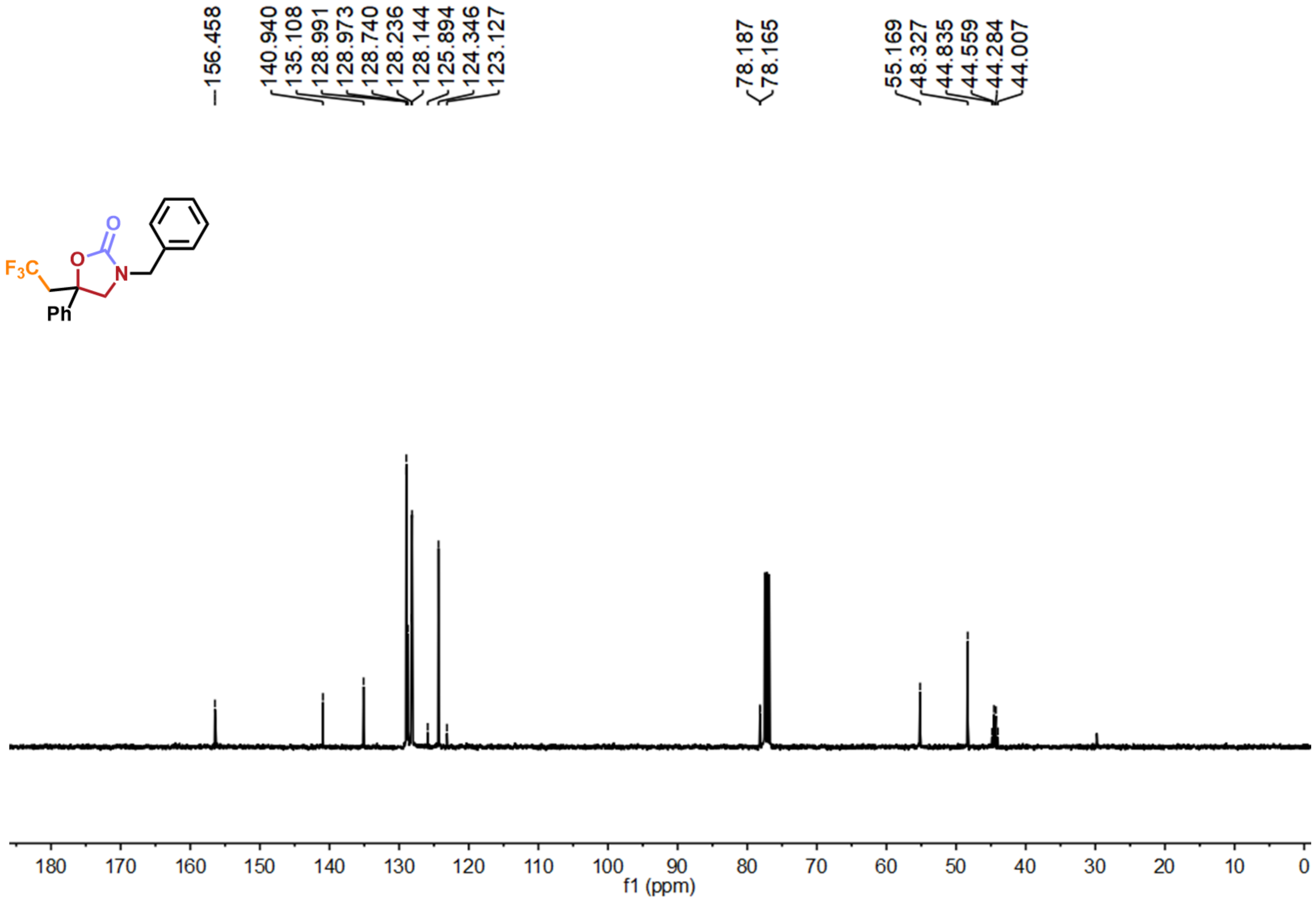

^{19}F NMR (376 MHz, $CDCl_3$) spectrum of **6a**

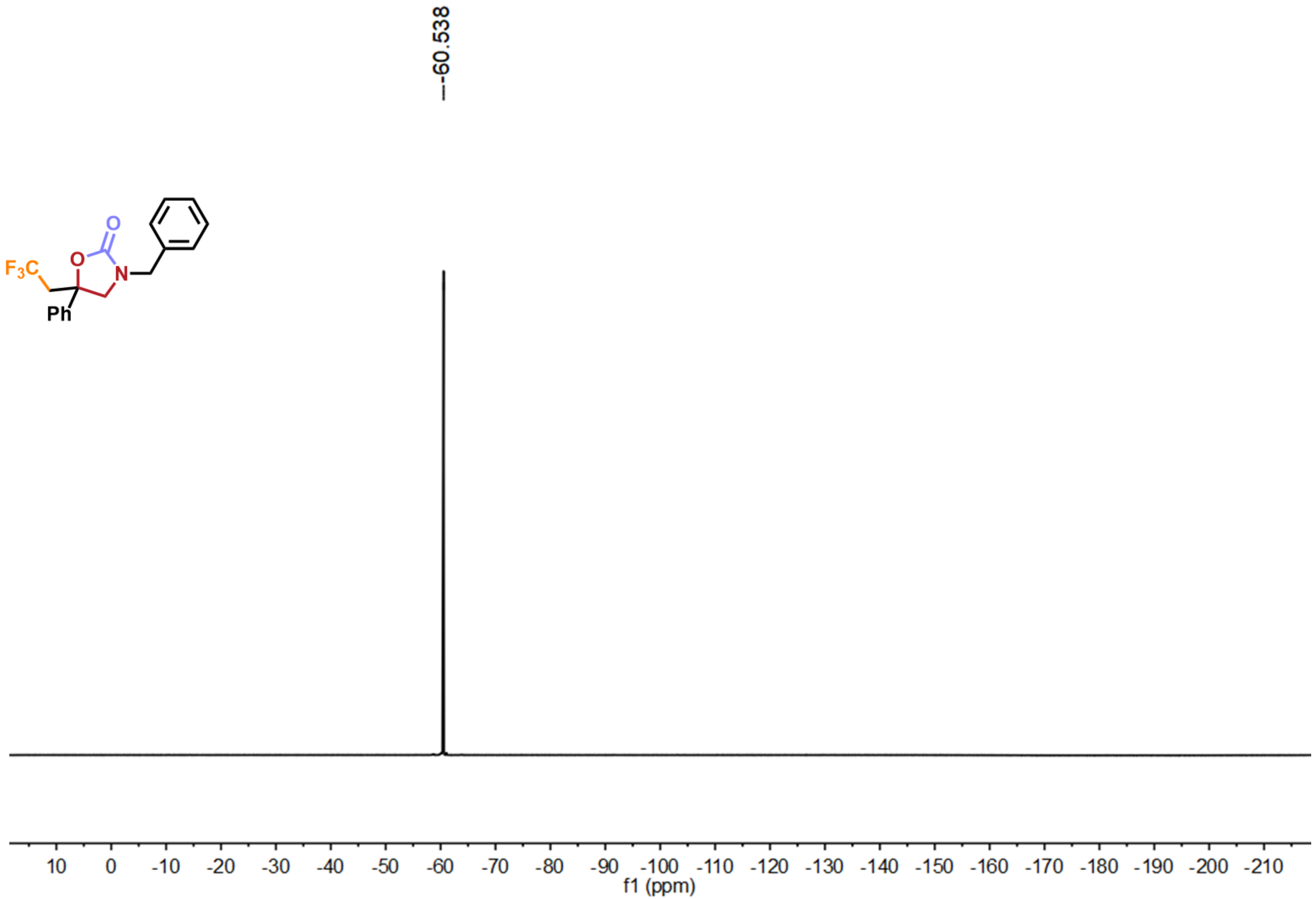

^{1}H NMR (400 MHz, $CDCl_3$) spectrum of **6b**

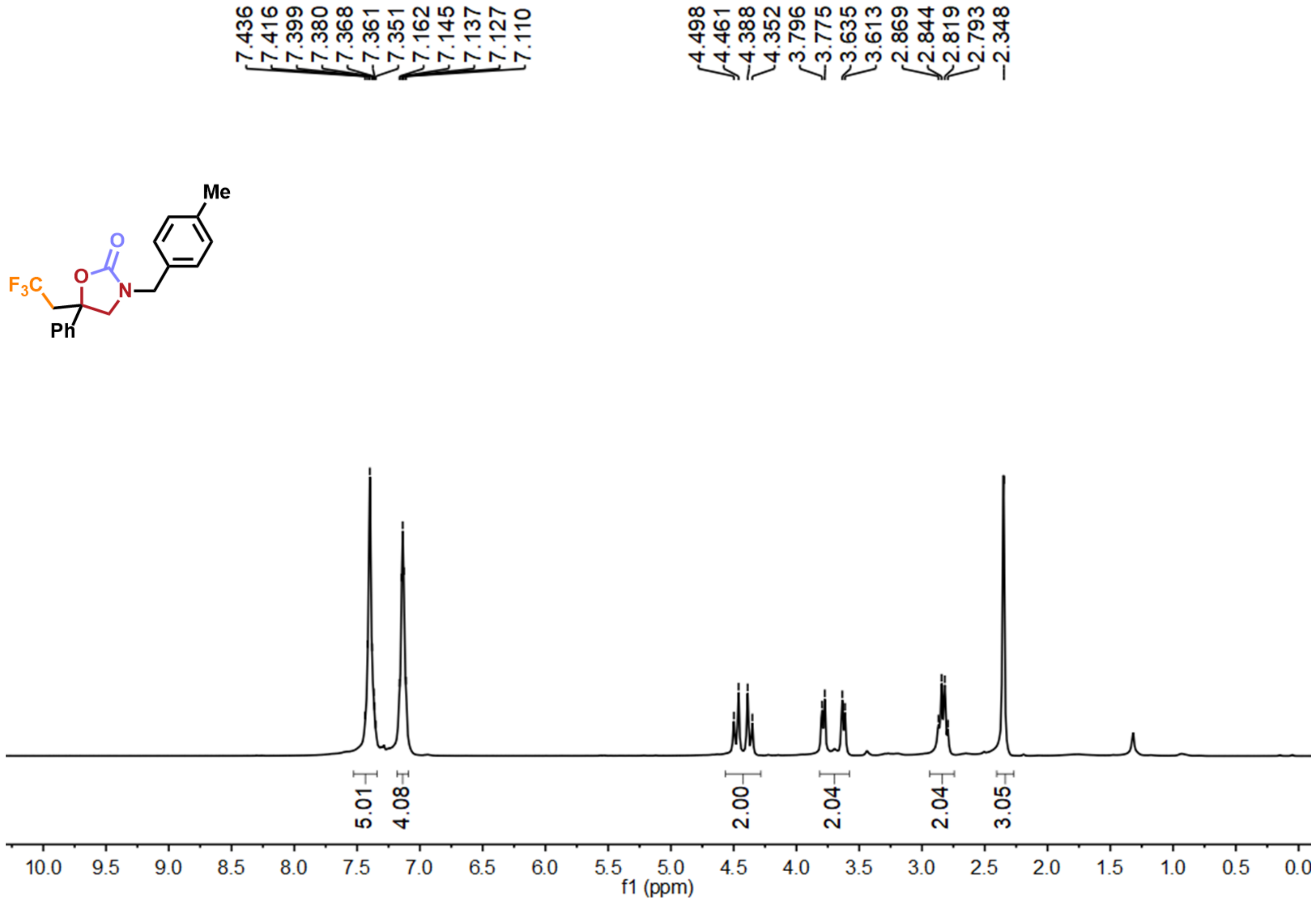

^{13}C NMR (100 MHz, $CDCl_3$) spectrum of **6b**

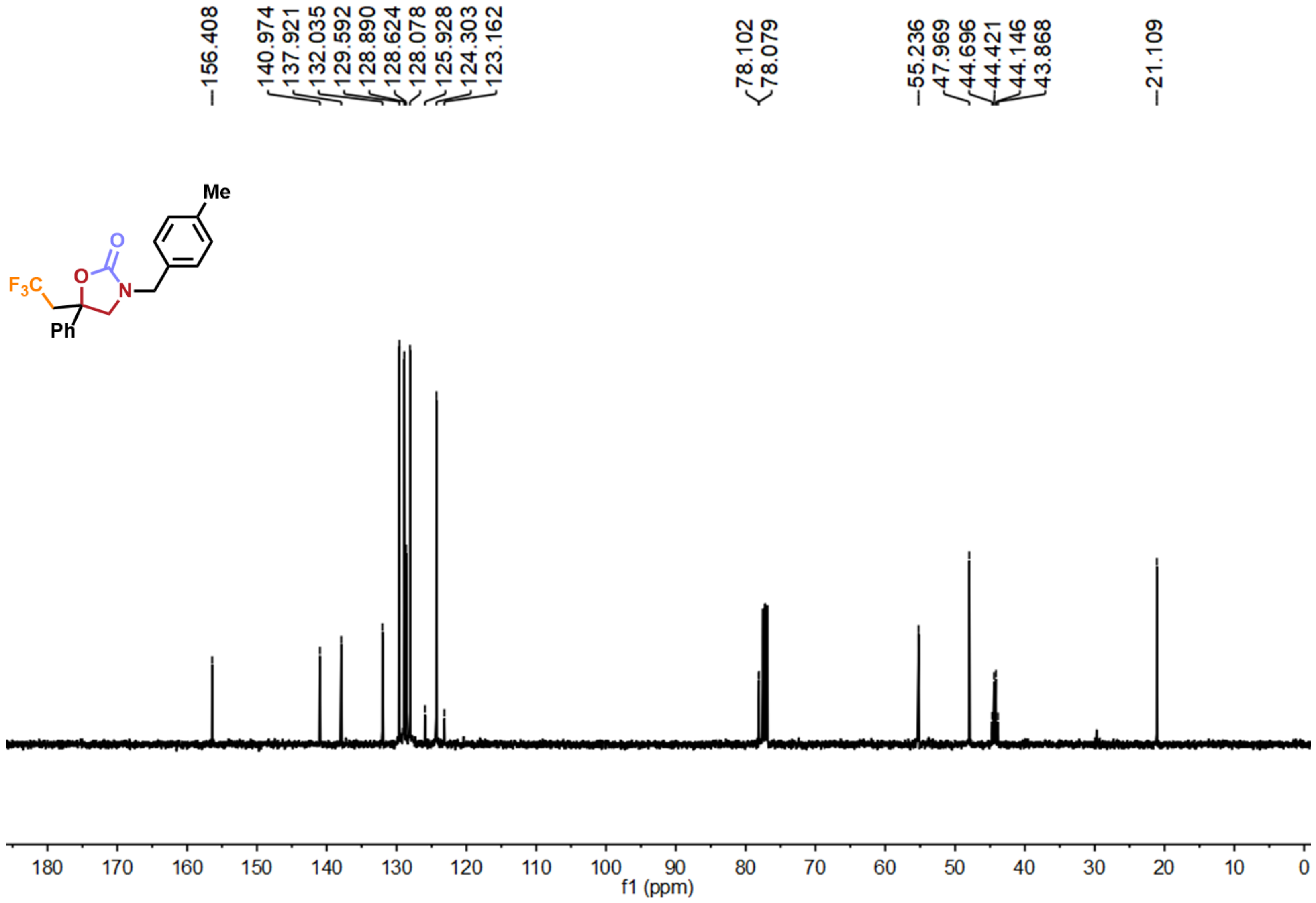

^{19}F NMR (376 MHz, $CDCl_3$) spectrum of **6b**

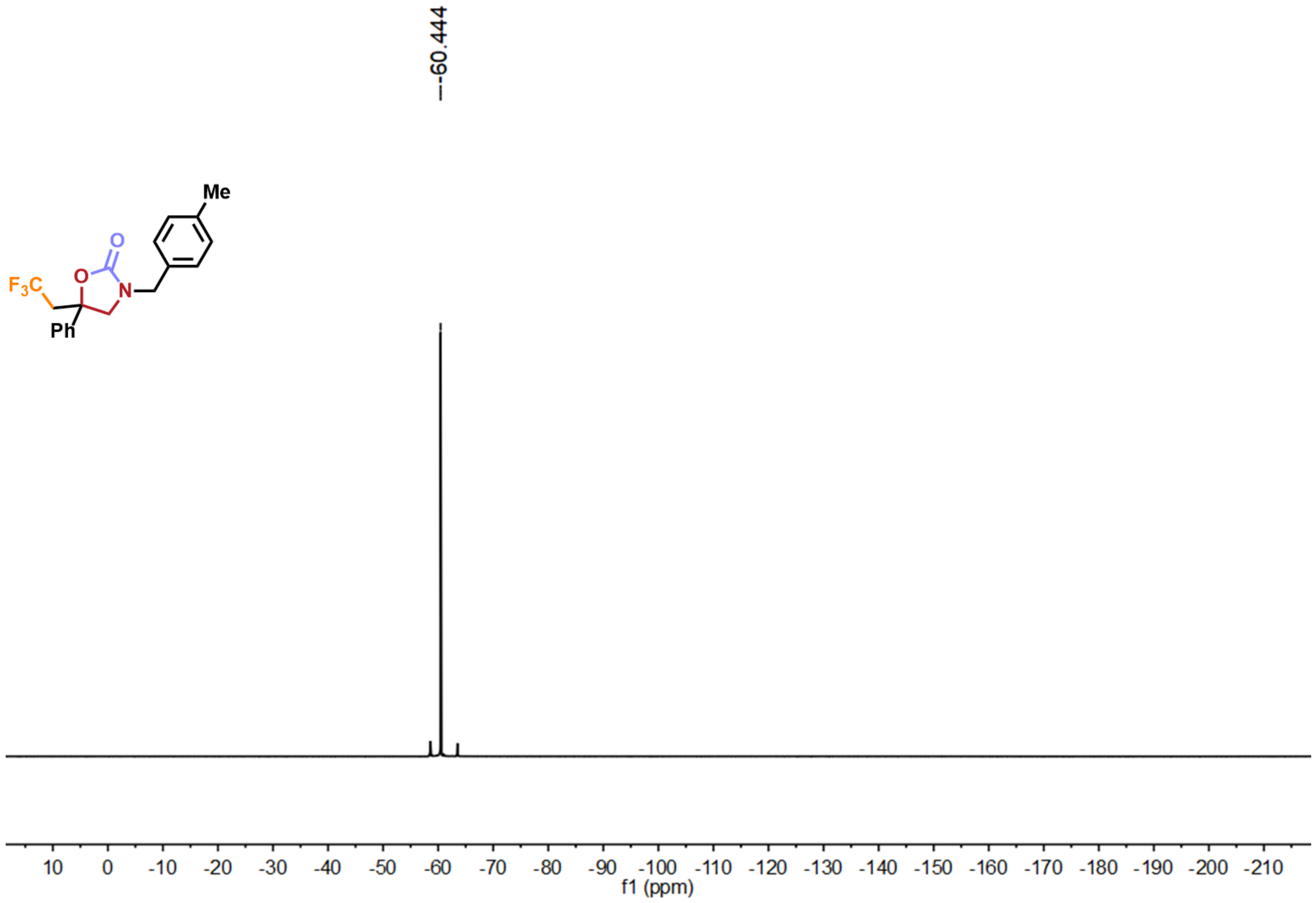

^{1}H NMR (400 MHz, $CDCl_3$) spectrum of **6c**

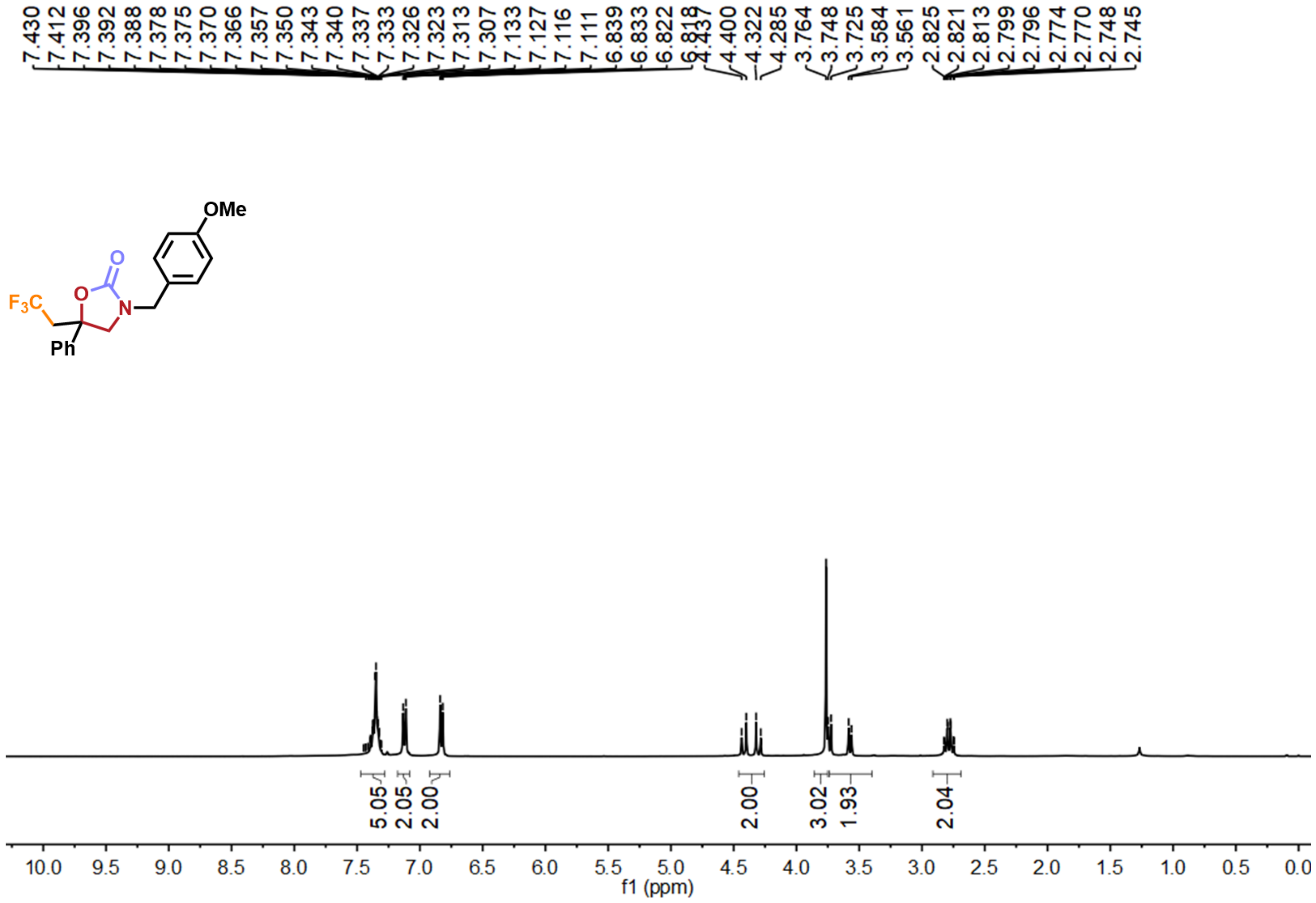

^{13}C NMR (100 MHz, $CDCl_3$) spectrum of **6c**

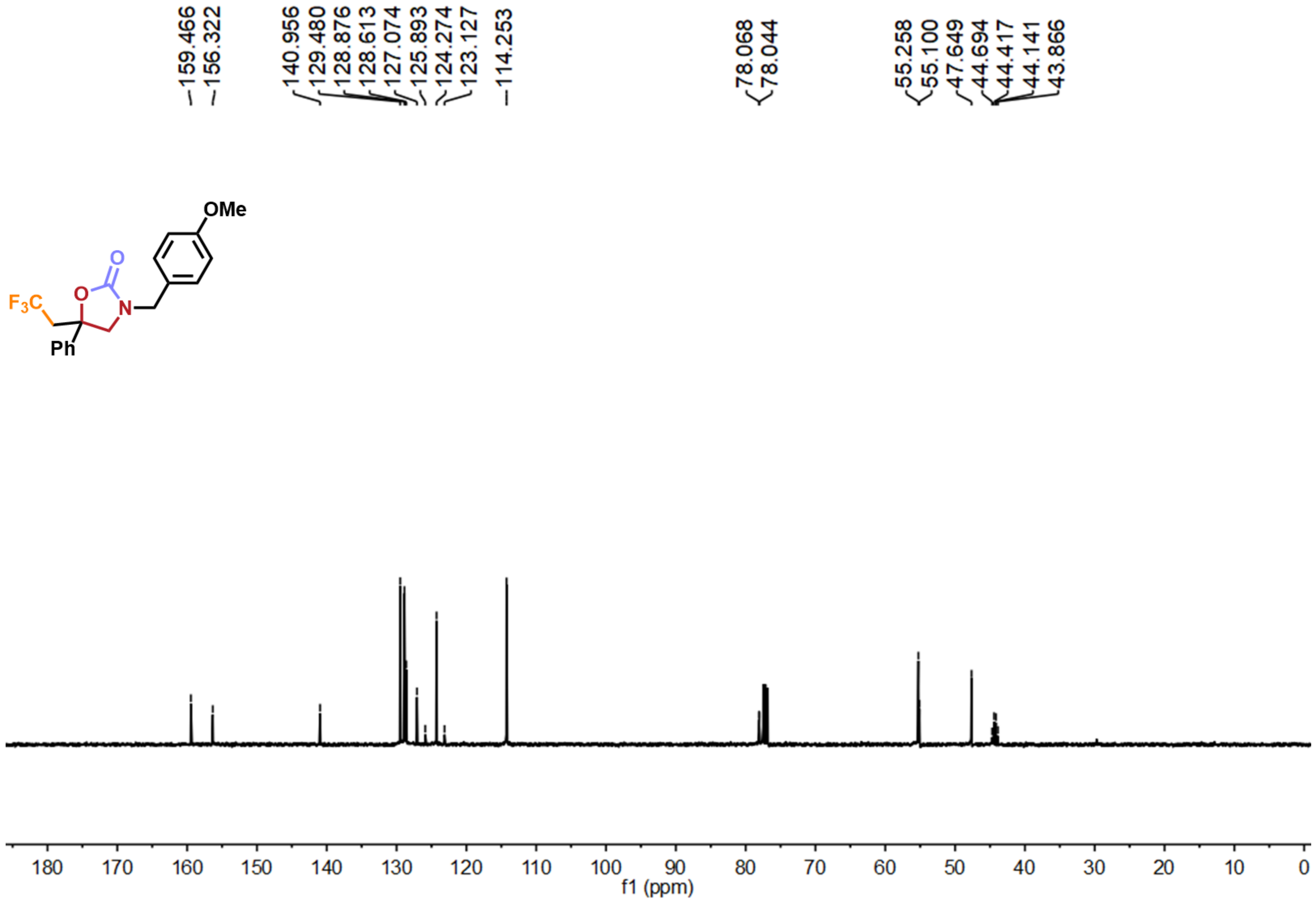

^{19}F NMR (376 MHz, $CDCl_3$) spectrum of **6c**

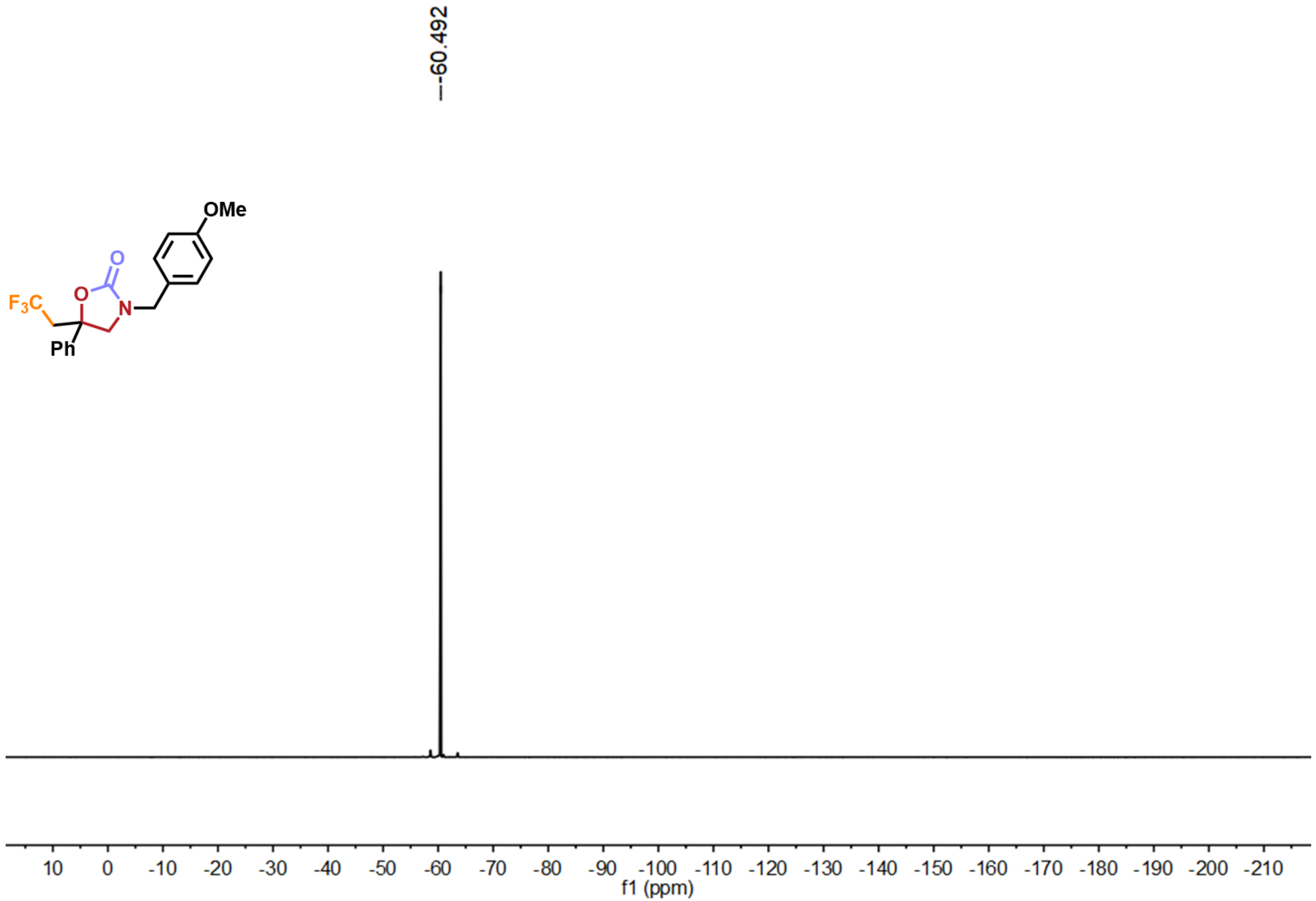

^{1}H NMR (400 MHz, $CDCl_3$) spectrum of **6d**

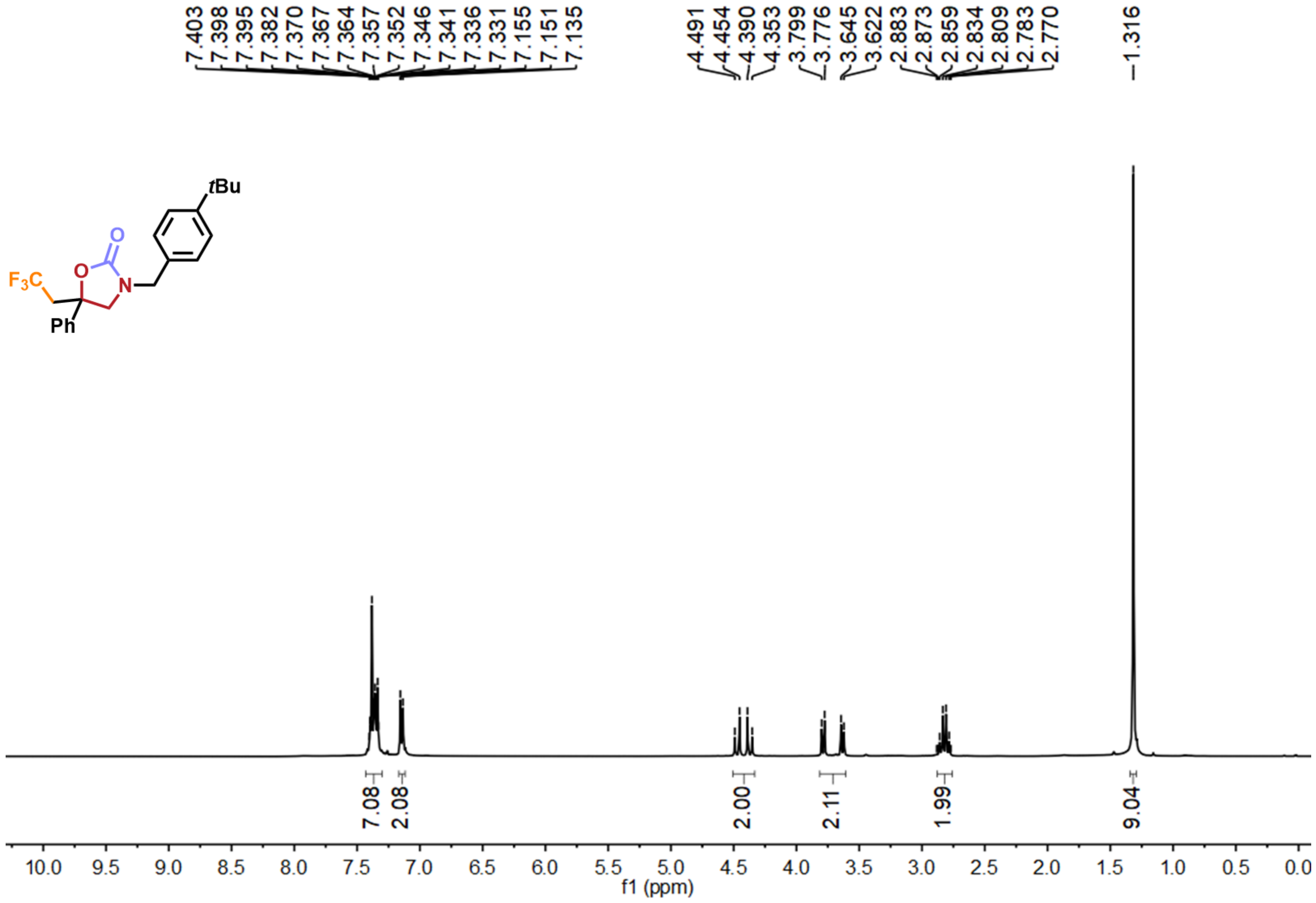

^{13}C NMR (100 MHz, $CDCl_3$) spectrum of **6d**

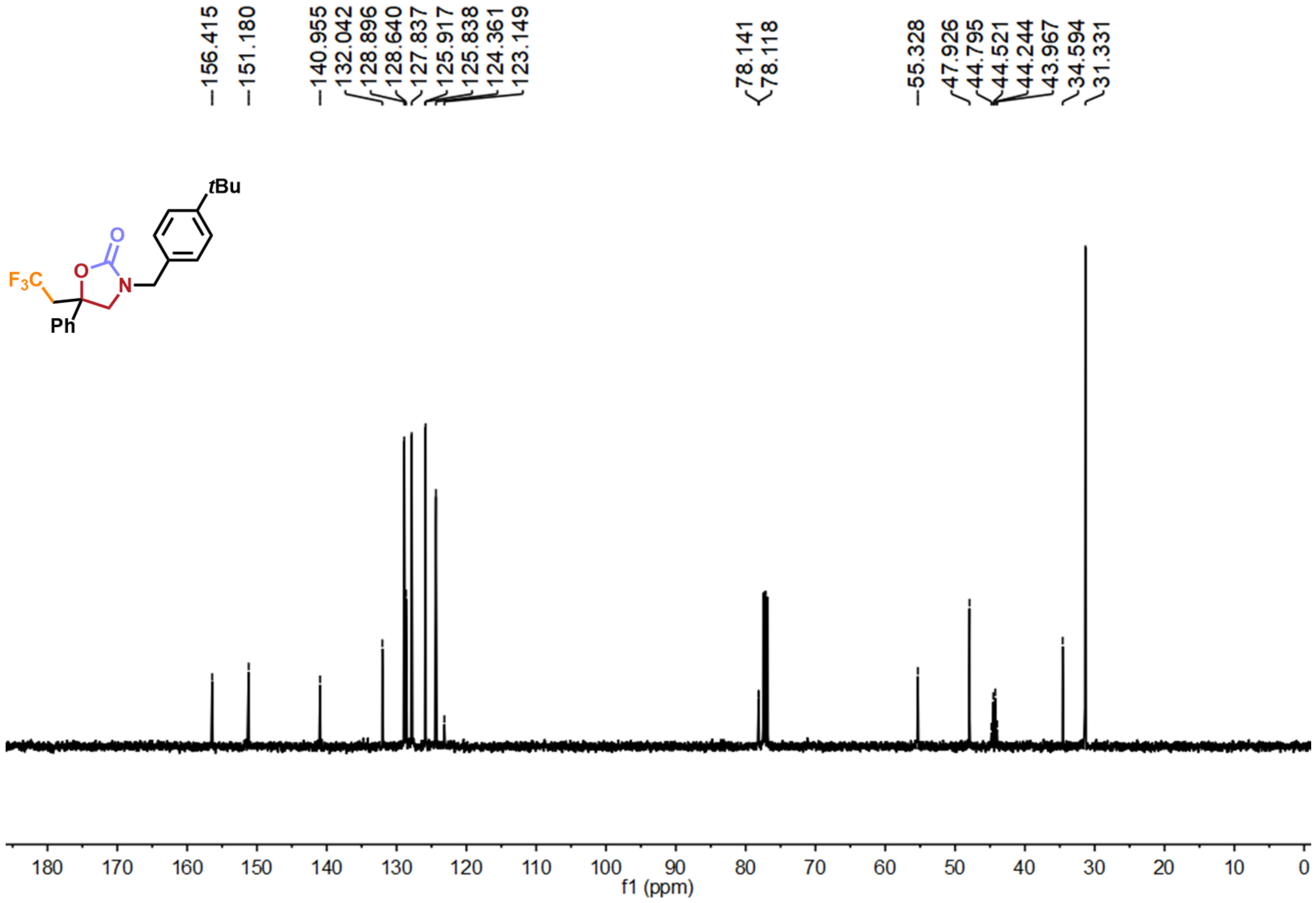

^{19}F NMR (376 MHz, $CDCl_3$) spectrum of **6d**

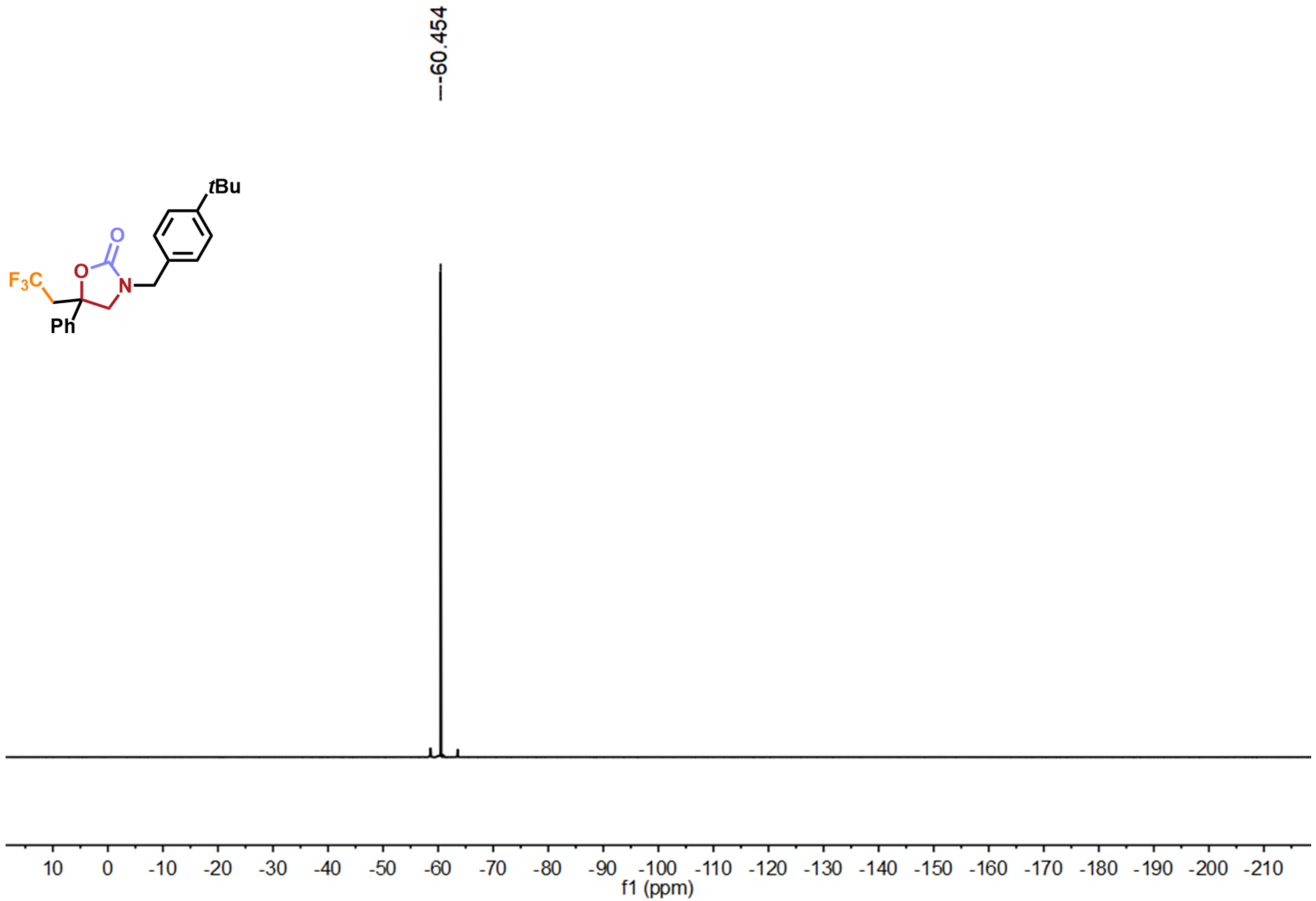

^1H NMR (400 MHz, $CDCl_3$) spectrum of **6e**

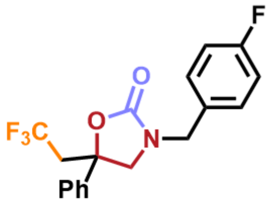

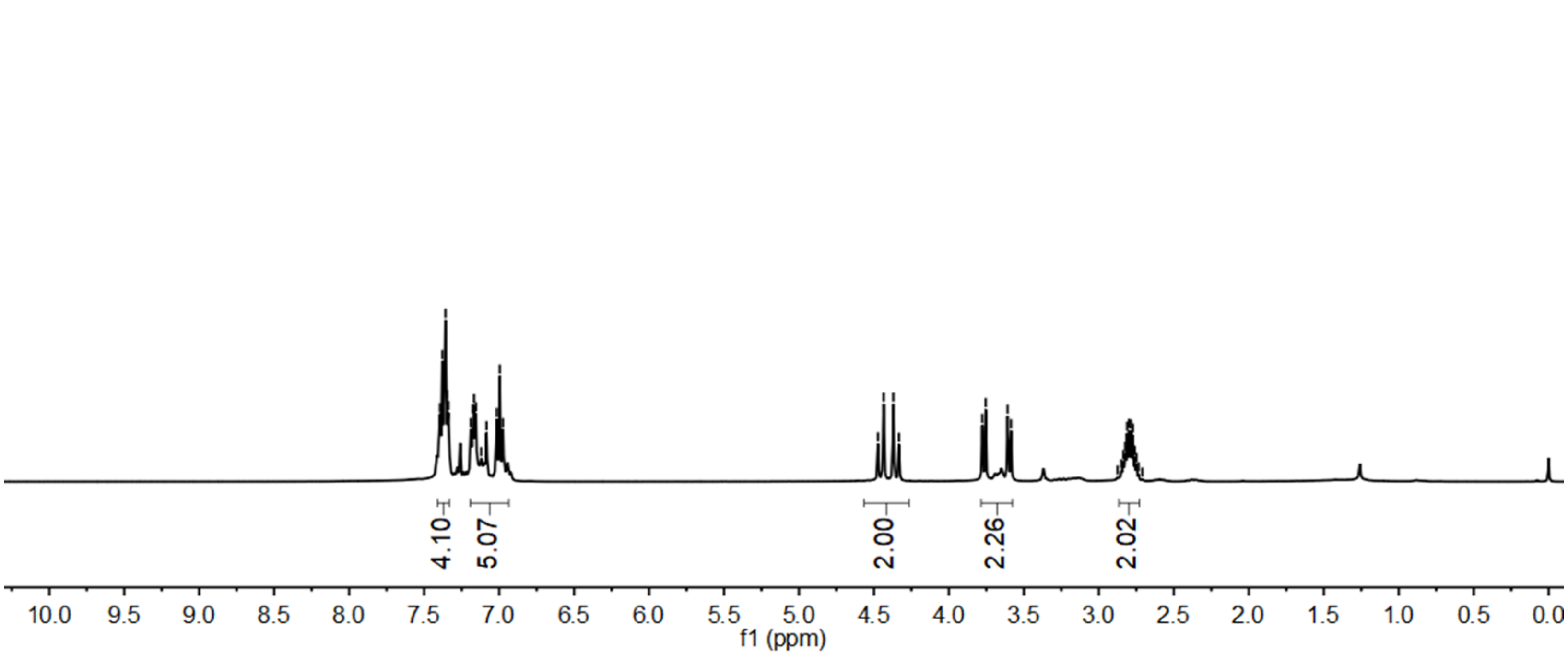

^{13}C NMR (100 MHz, $CDCl_3$) spectrum of **6e**

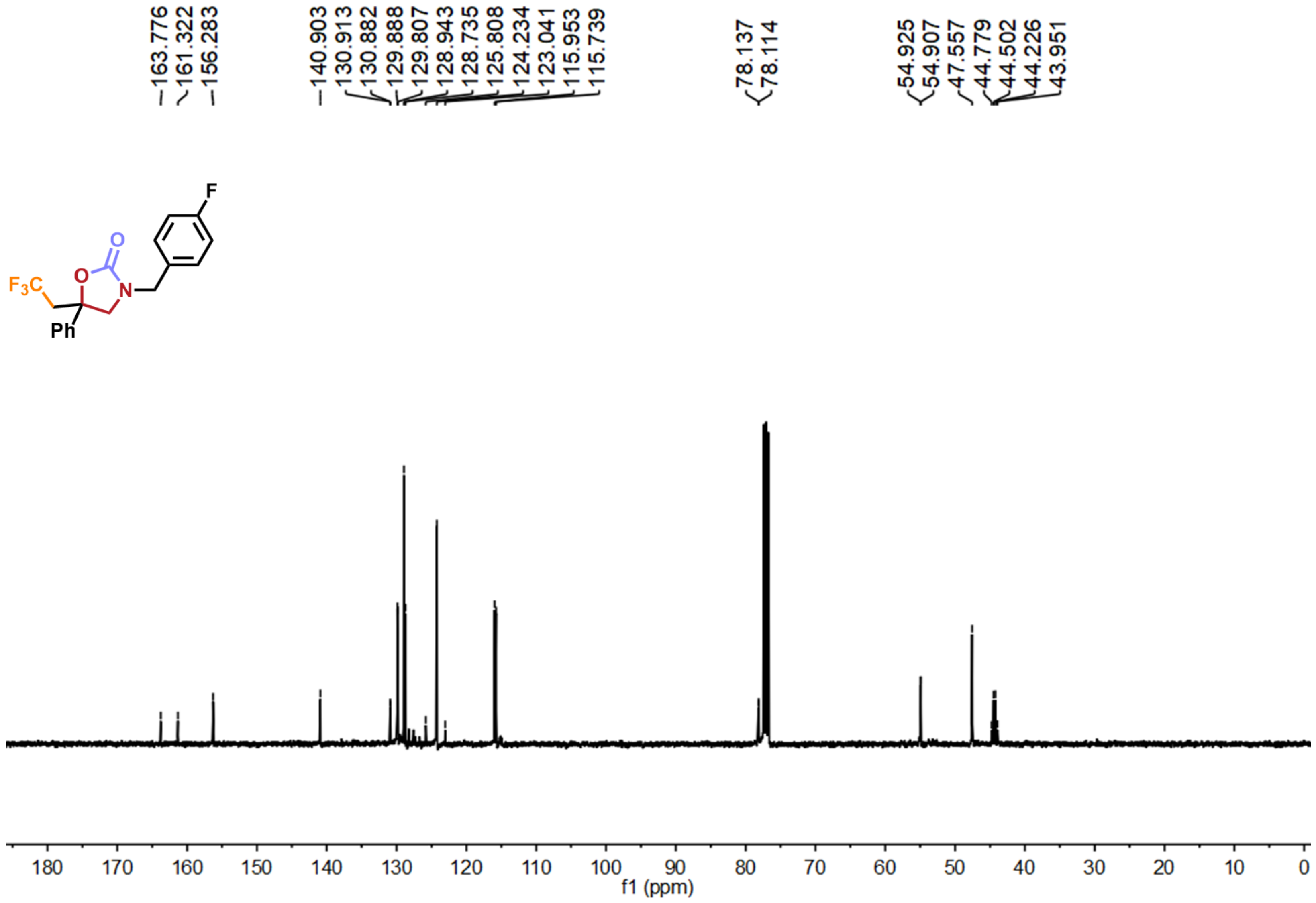

^{19}F NMR (376 MHz, $CDCl_3$) spectrum of **6e**

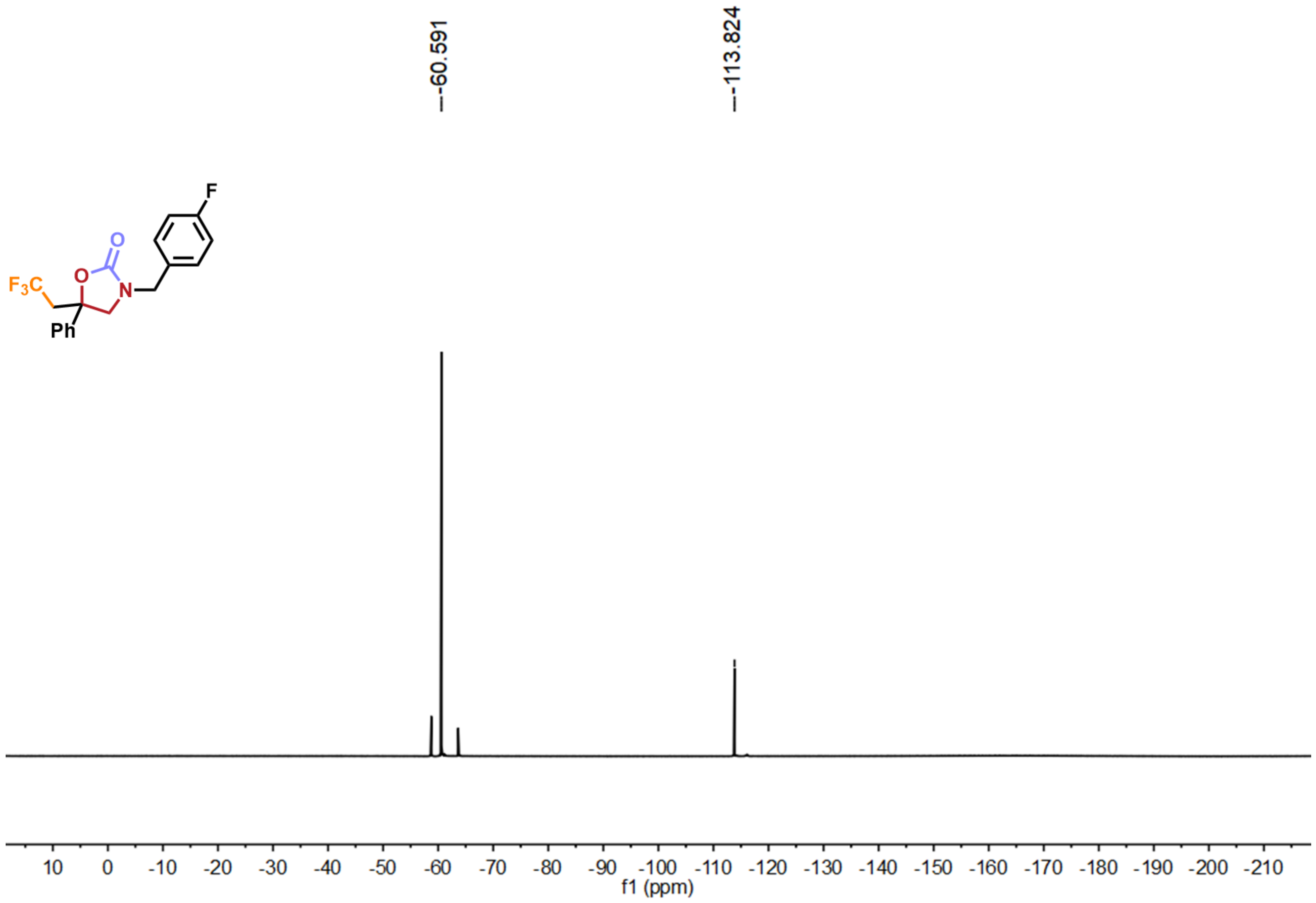

^{1}H NMR (400 MHz, $CDCl_3$) spectrum of **6f**

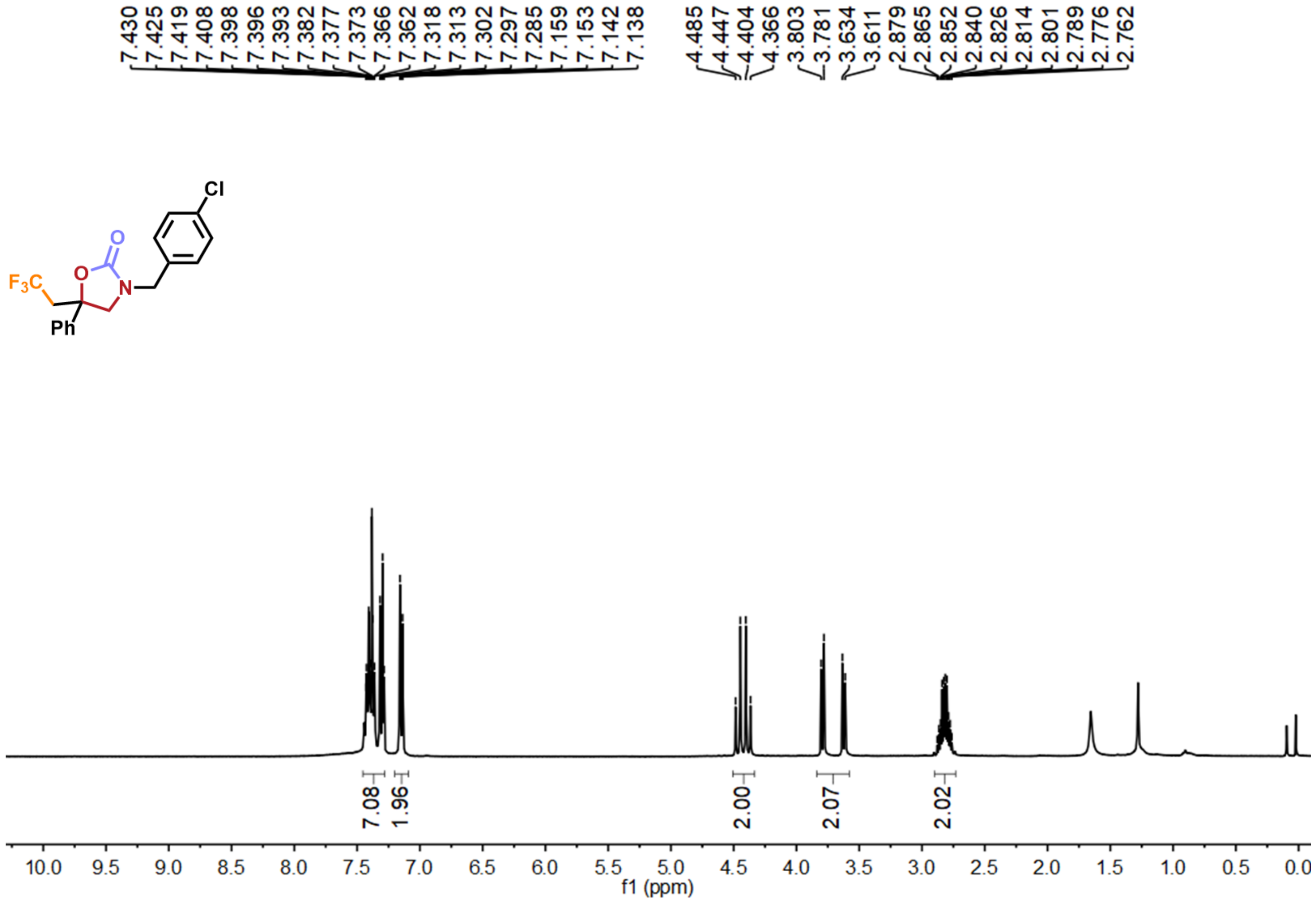

^{13}C NMR (100 MHz, $CDCl_3$) spectrum of **6f**

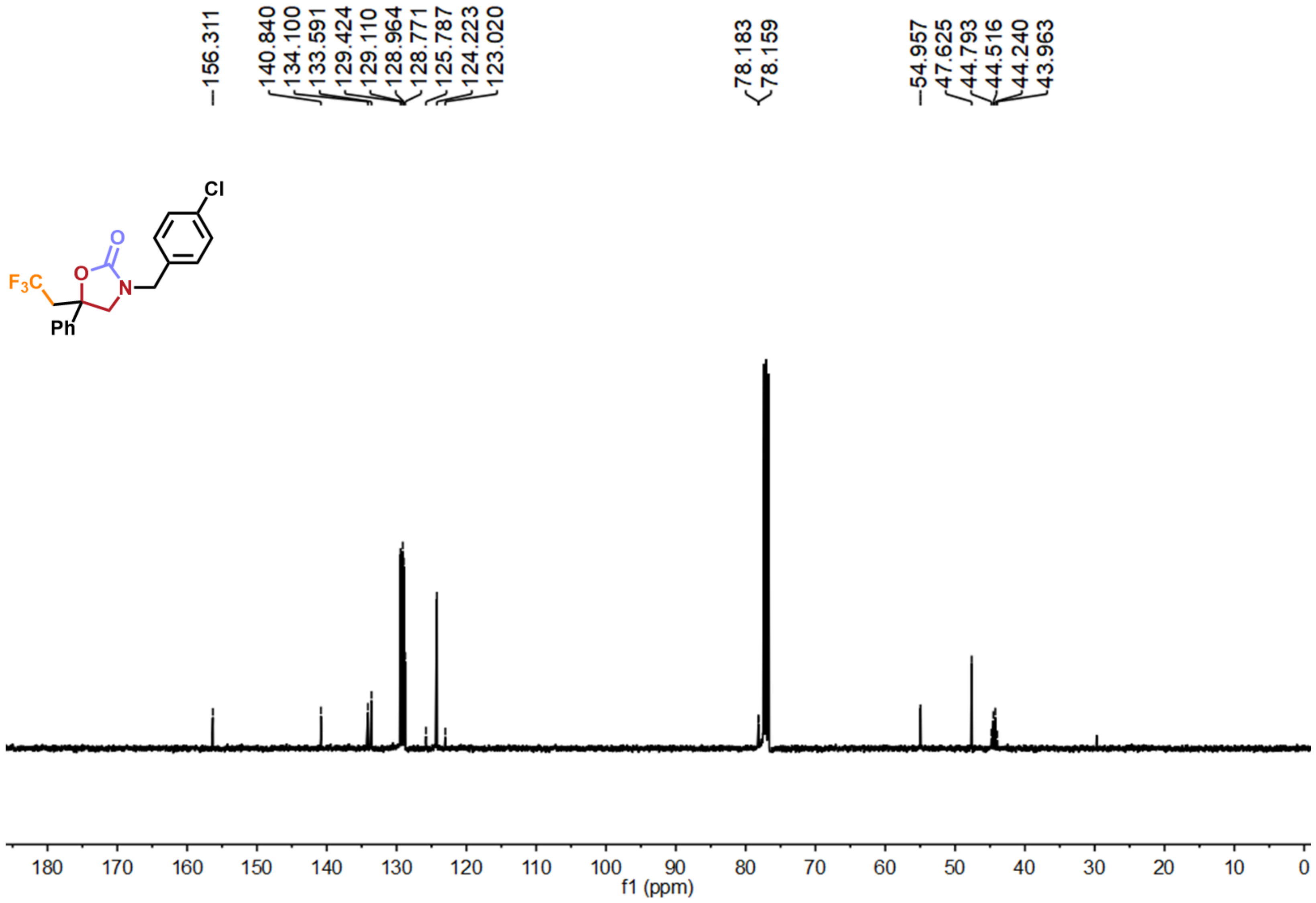

^{19}F NMR (376 MHz, $CDCl_3$) spectrum of **6f**

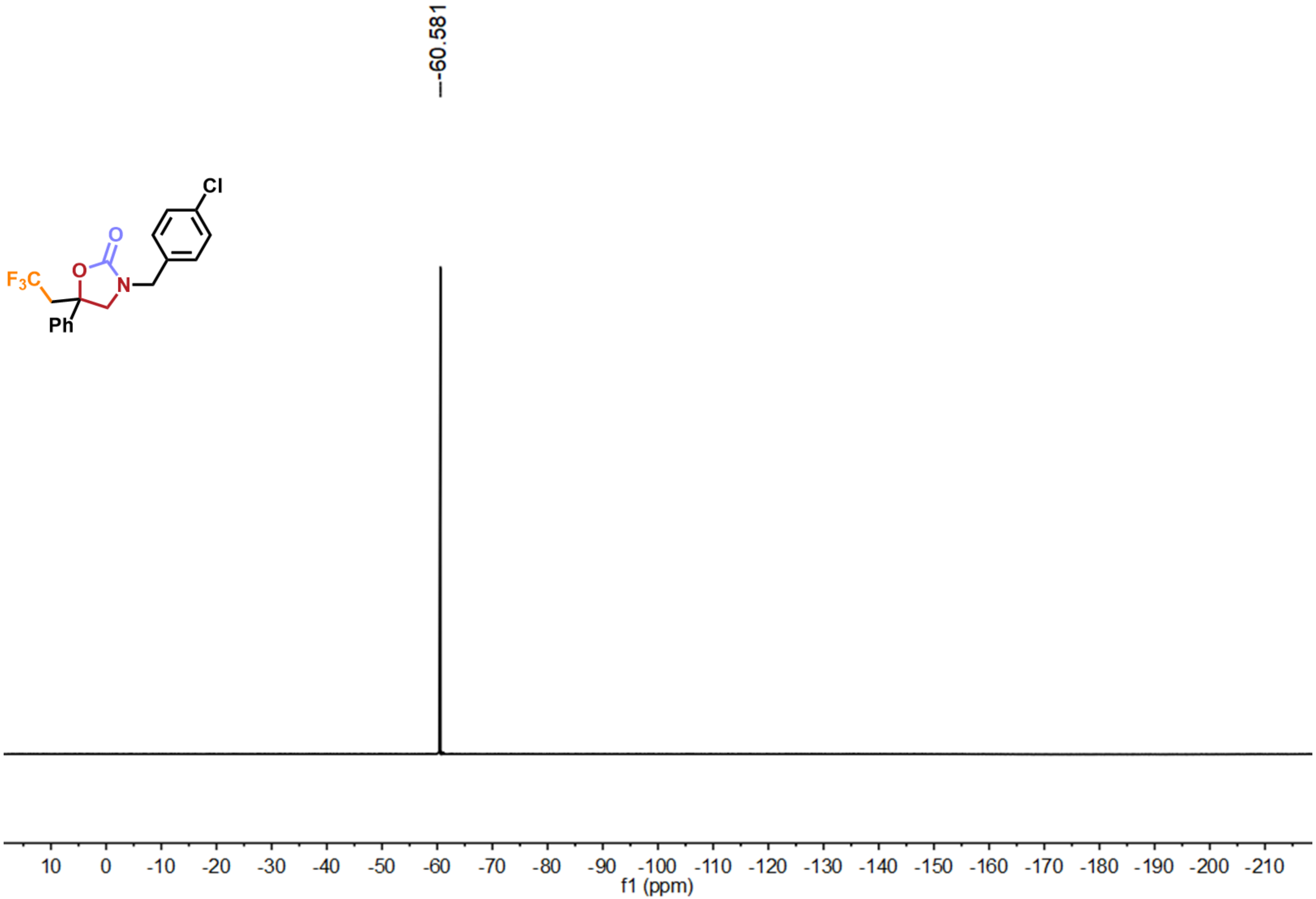

^{1}H NMR (400 MHz, $CDCl_3$) spectrum of **6g**

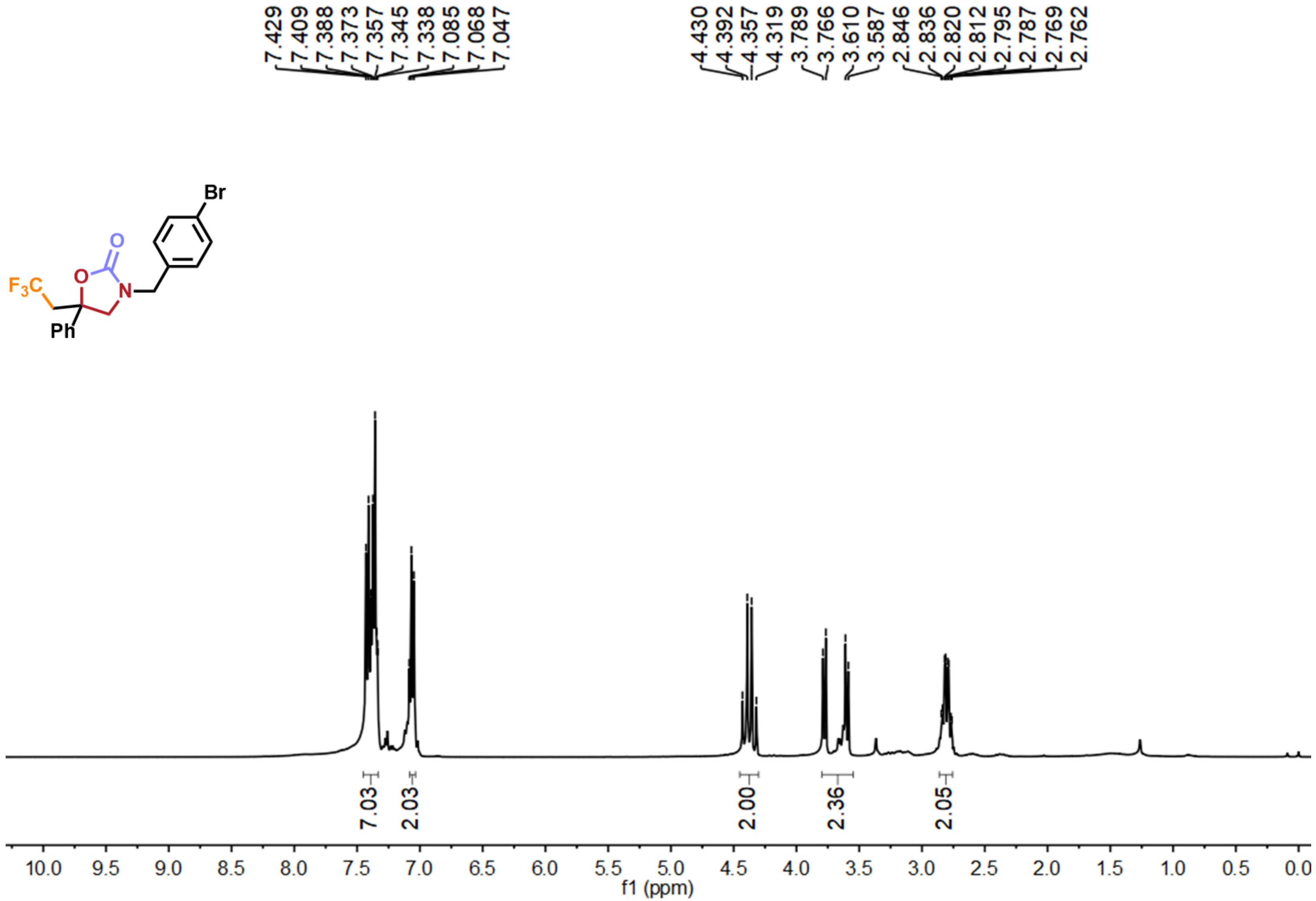

^{13}C NMR (100 MHz, $CDCl_3$) spectrum of **6g**

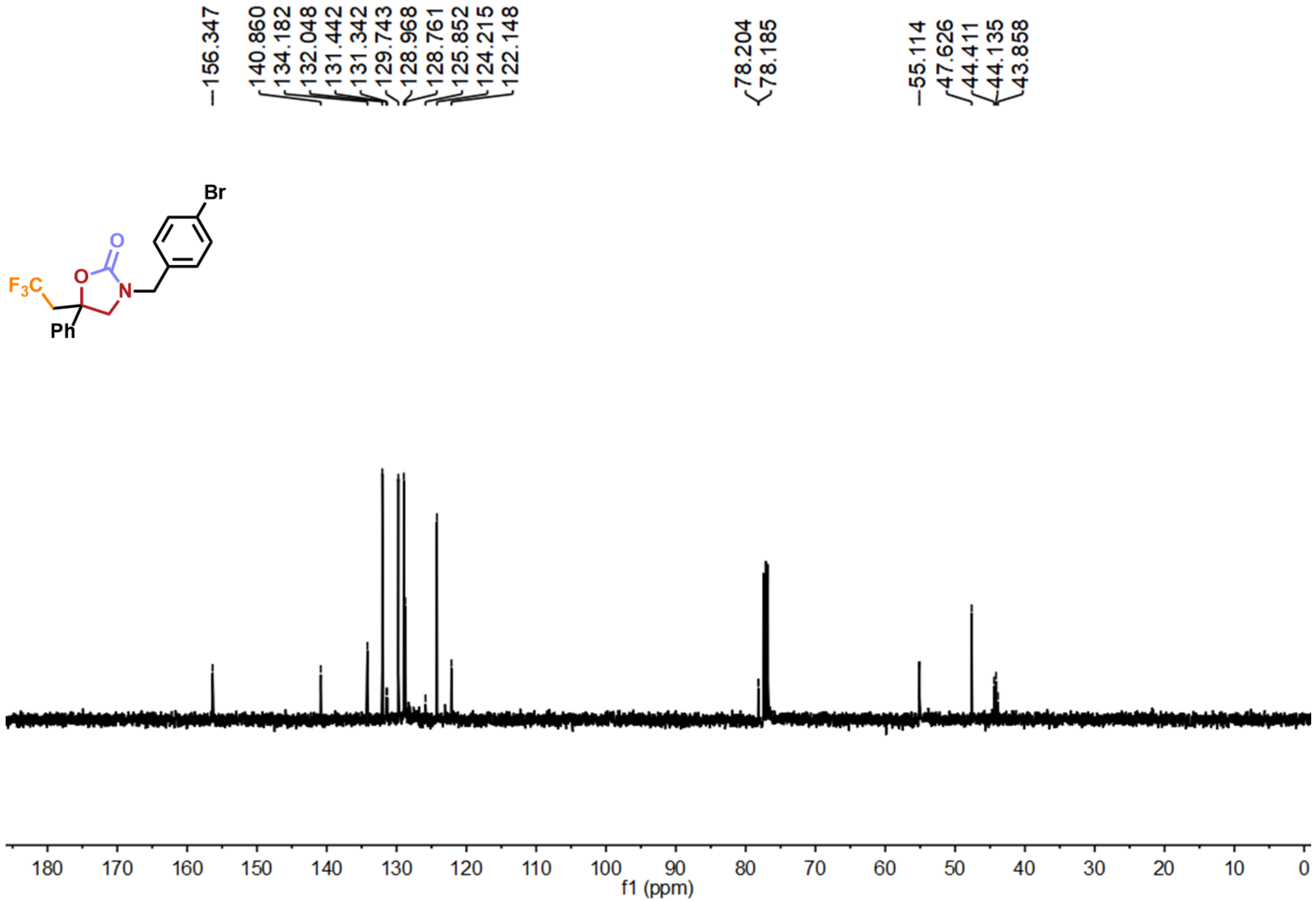

^{19}F NMR (376 MHz, $CDCl_3$) spectrum of **6g**

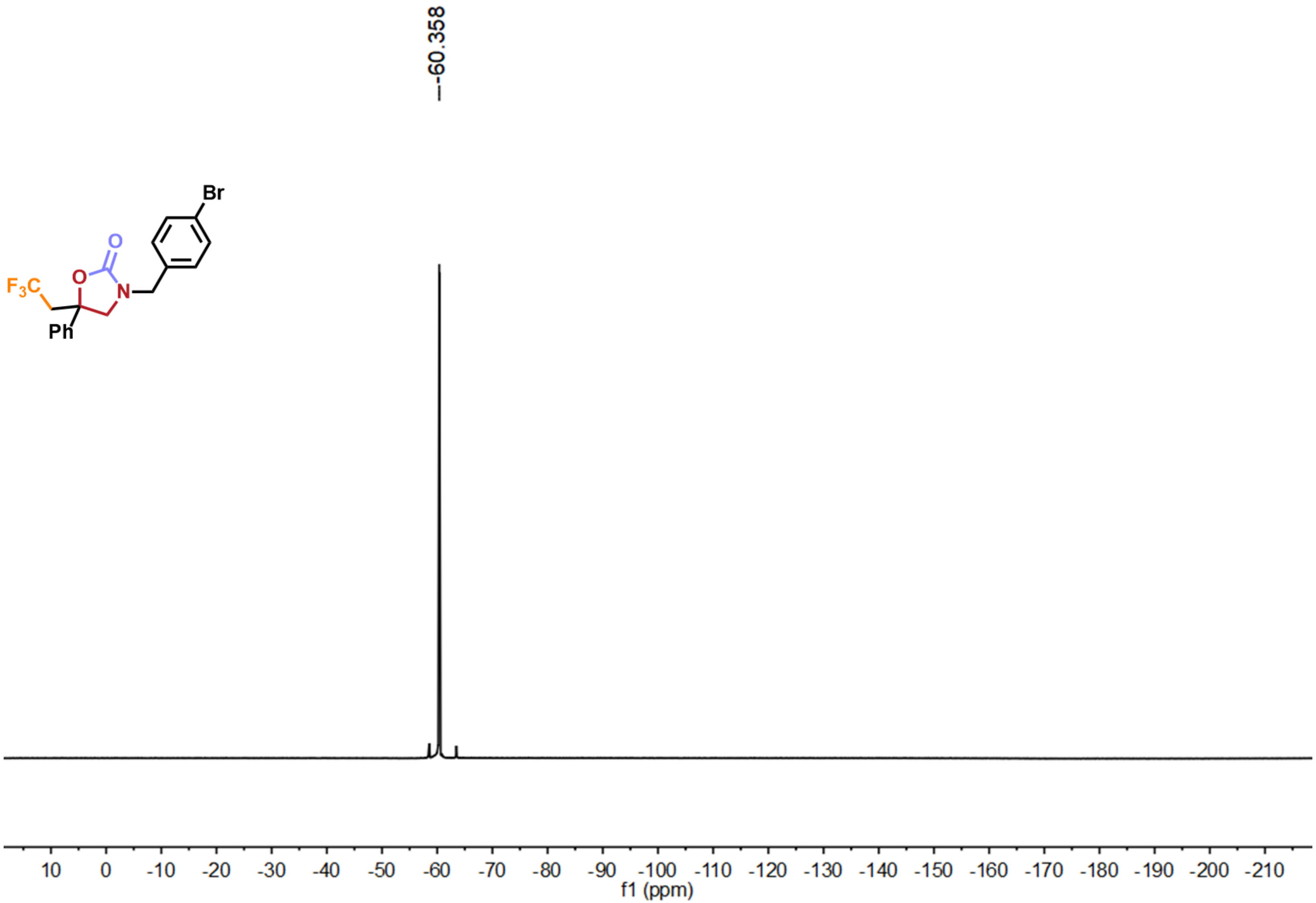

^{1}H NMR (400 MHz, $CDCl_3$) spectrum of **6h**

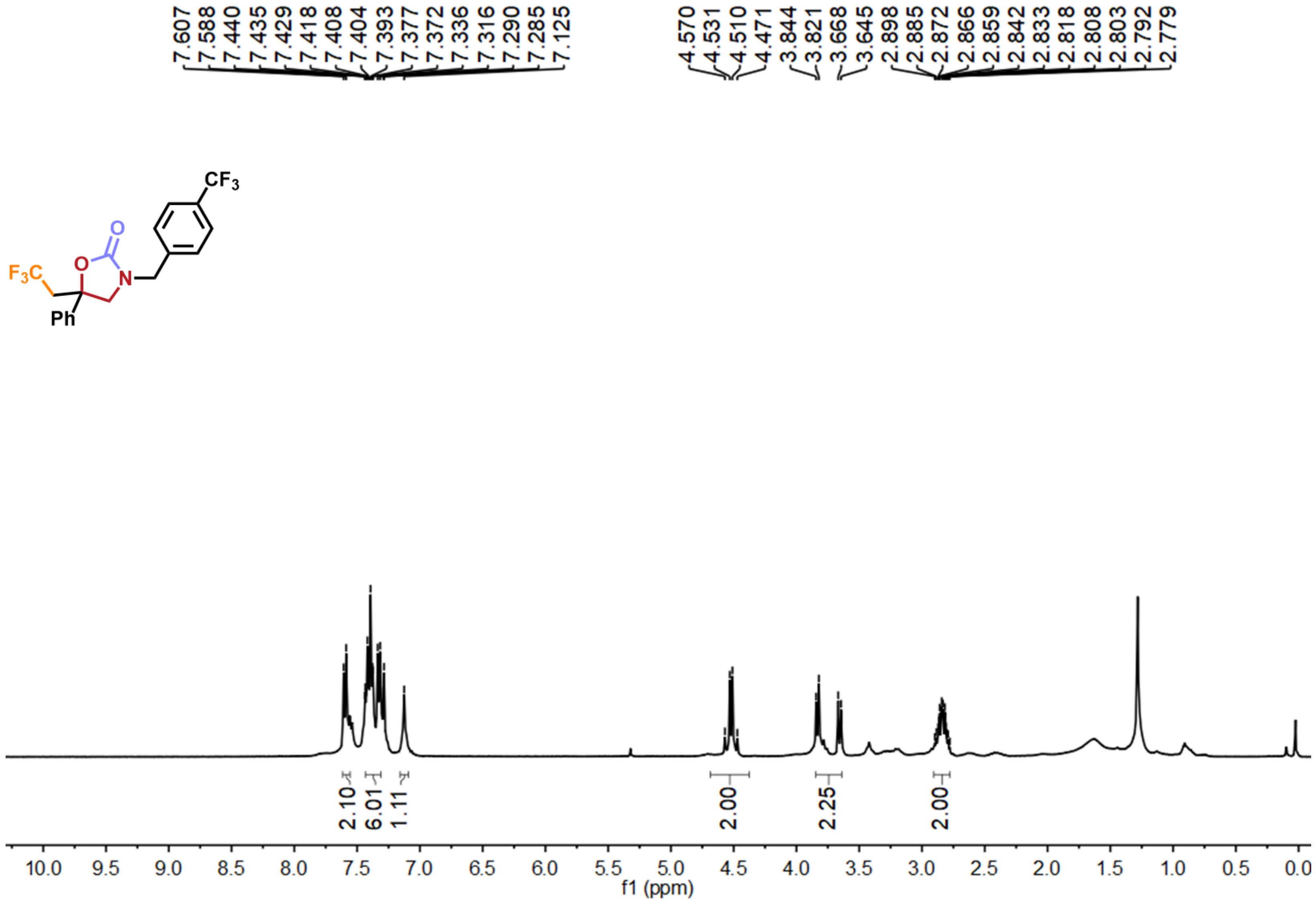

^{13}C NMR (100 MHz, $CDCl_3$) spectrum of **6h**

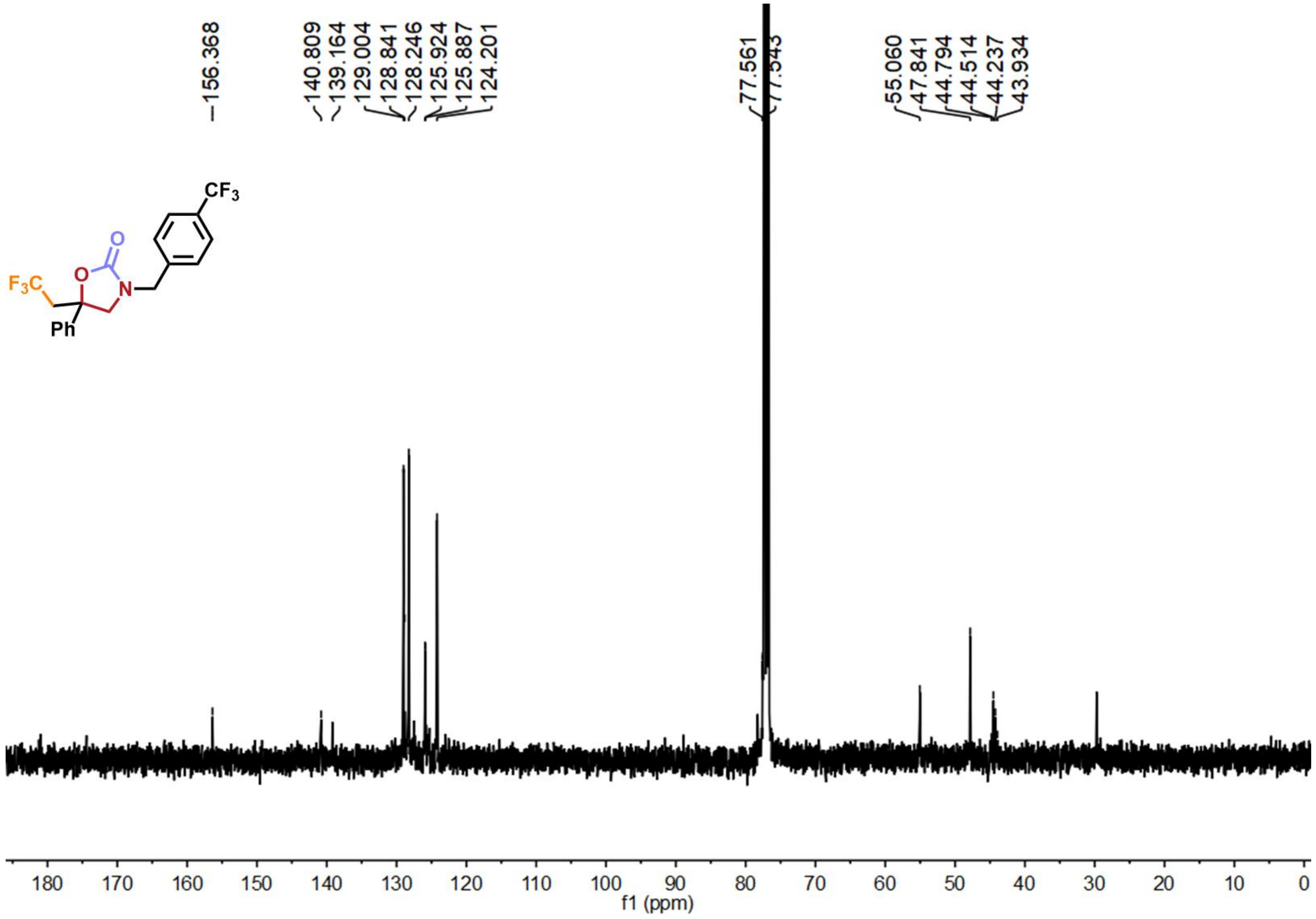

^{19}F NMR (376 MHz, $CDCl_3$) spectrum of **6h**

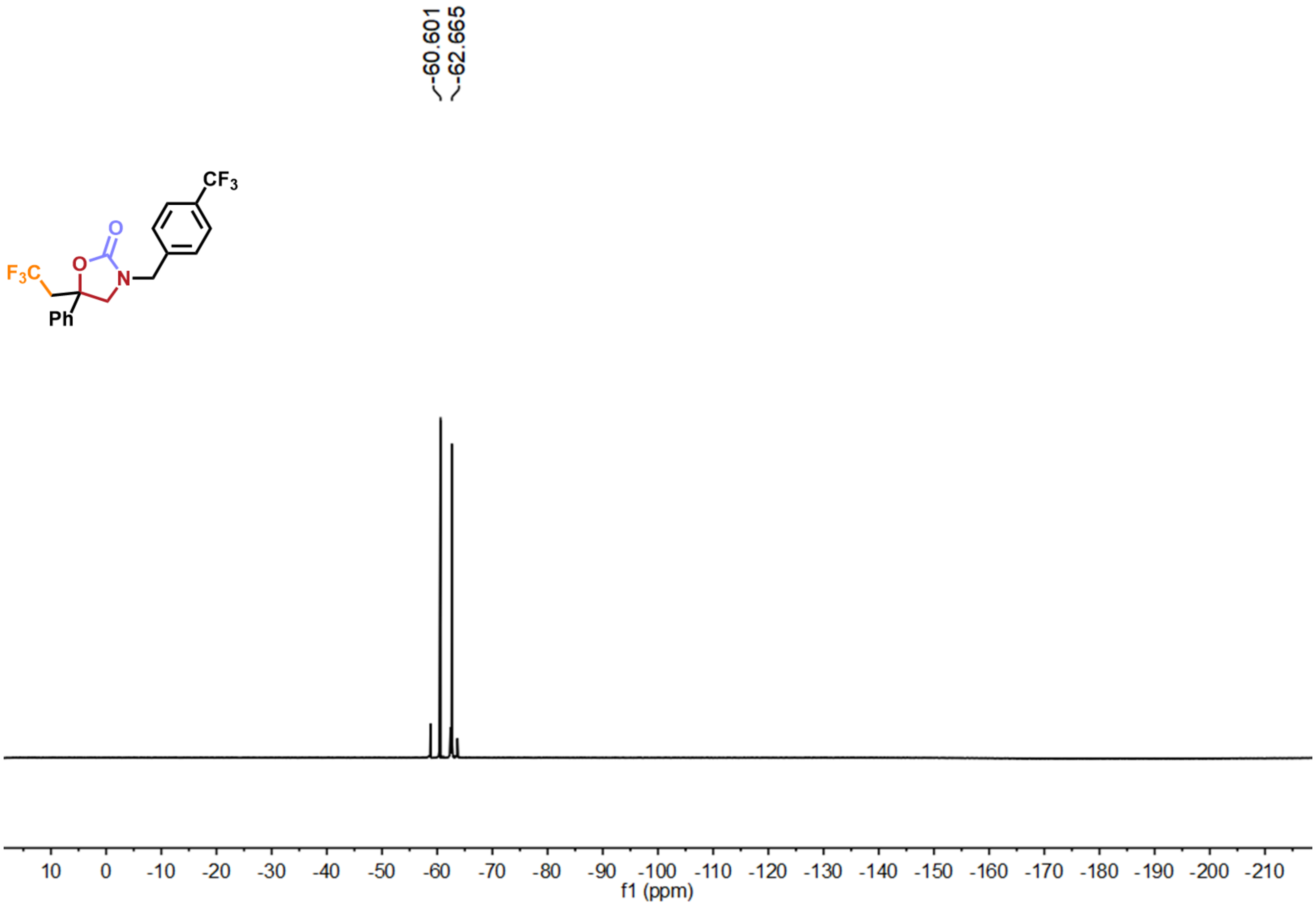

^{1}H NMR (400 MHz, $CDCl_3$) spectrum of **6i**

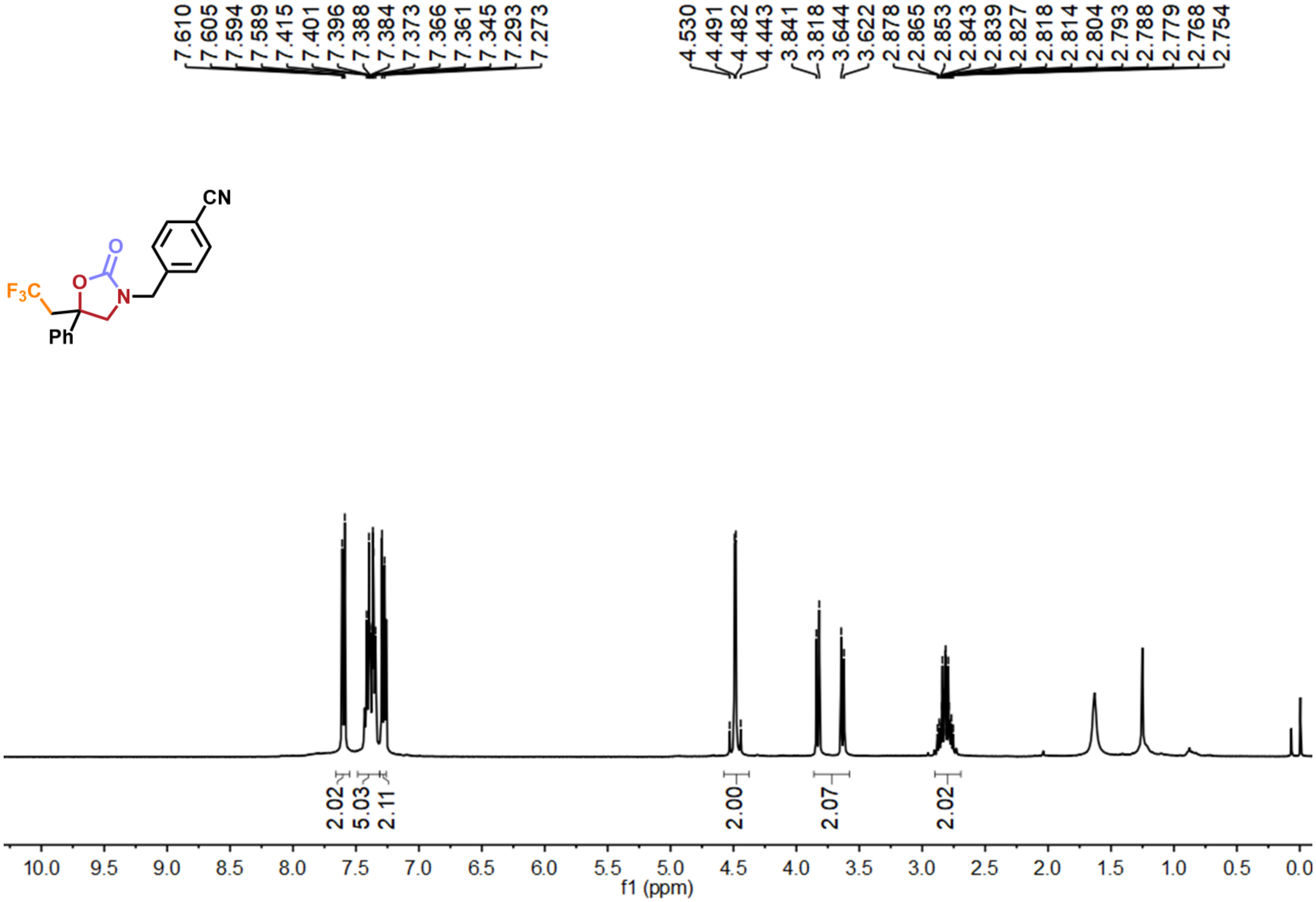

^{13}C NMR (100 MHz, $CDCl_3$) spectrum of **6i**

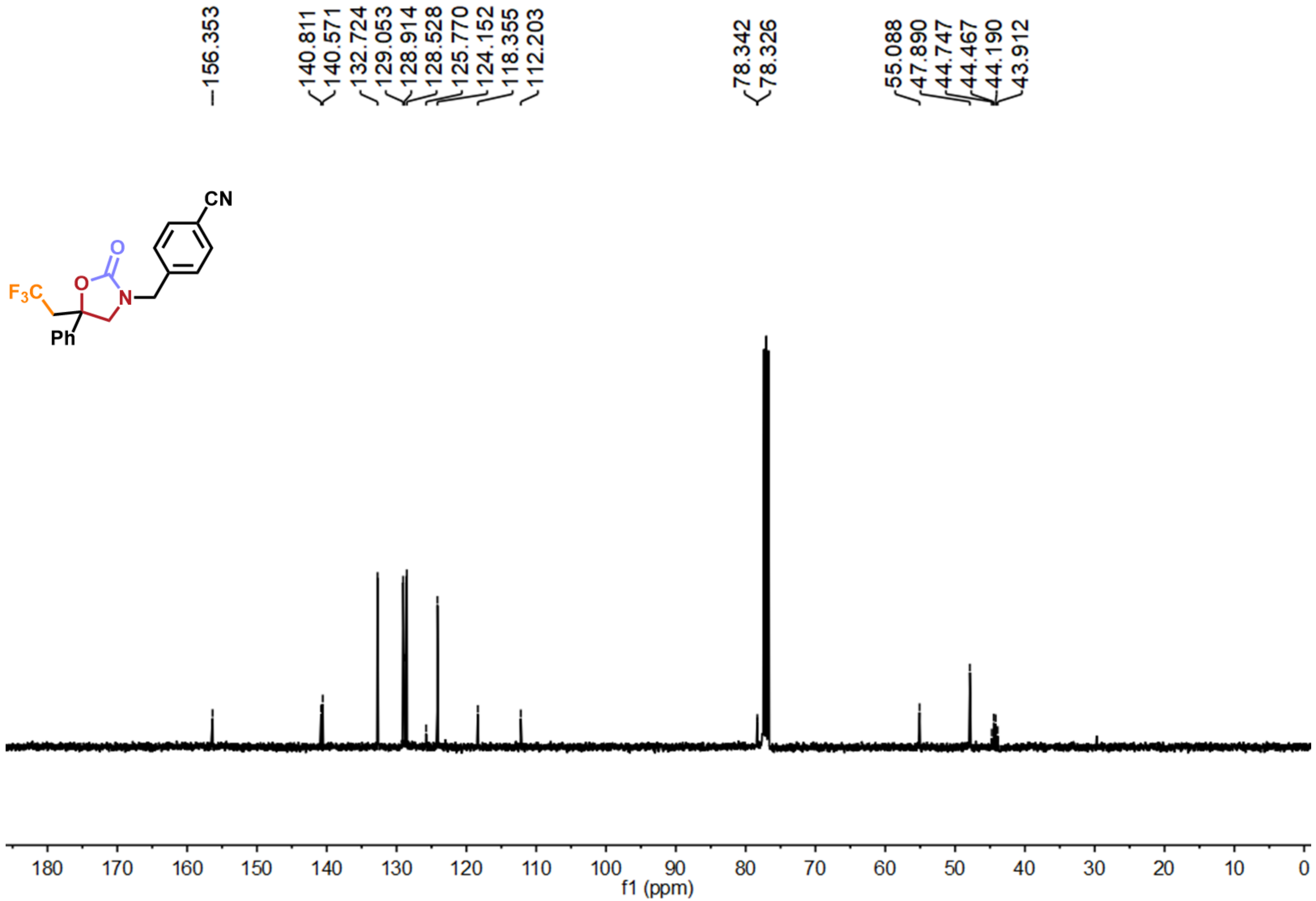

^{19}F NMR (376 MHz, $CDCl_3$) spectrum of **6i**

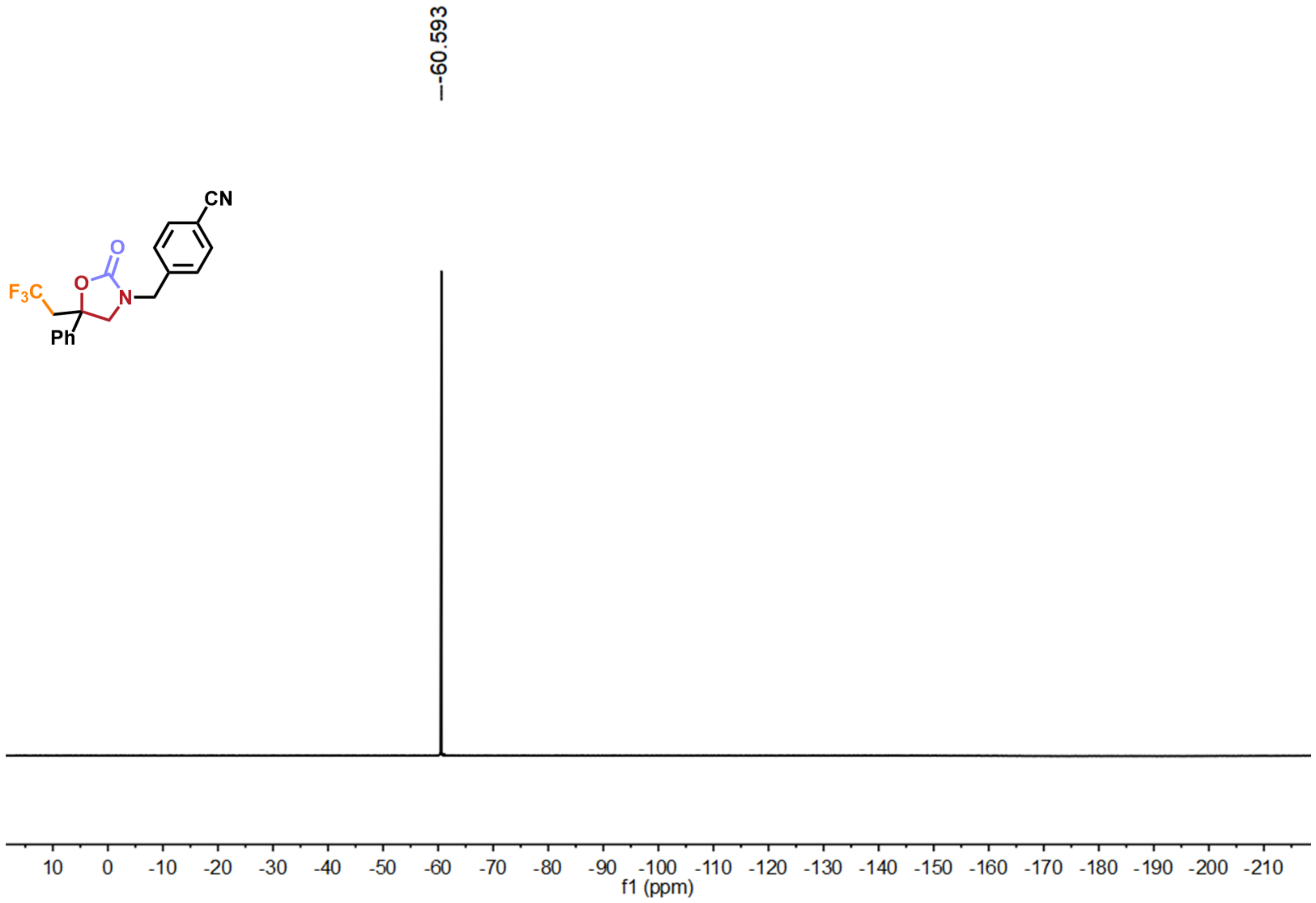

^{1}H NMR (400 MHz, $CDCl_3$) spectrum of **6j**

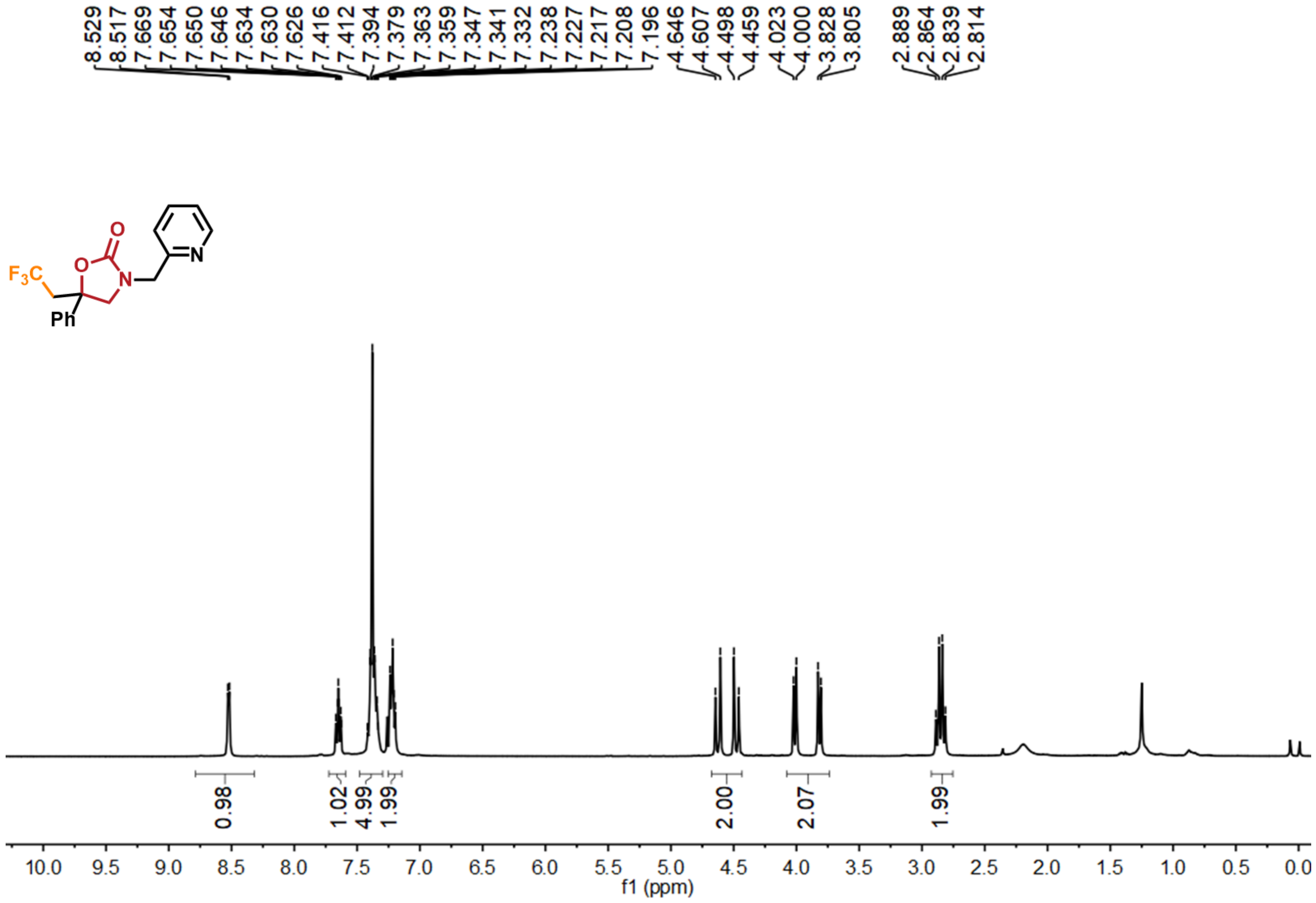

^{13}C NMR (100 MHz, $CDCl_3$) spectrum of **6j**

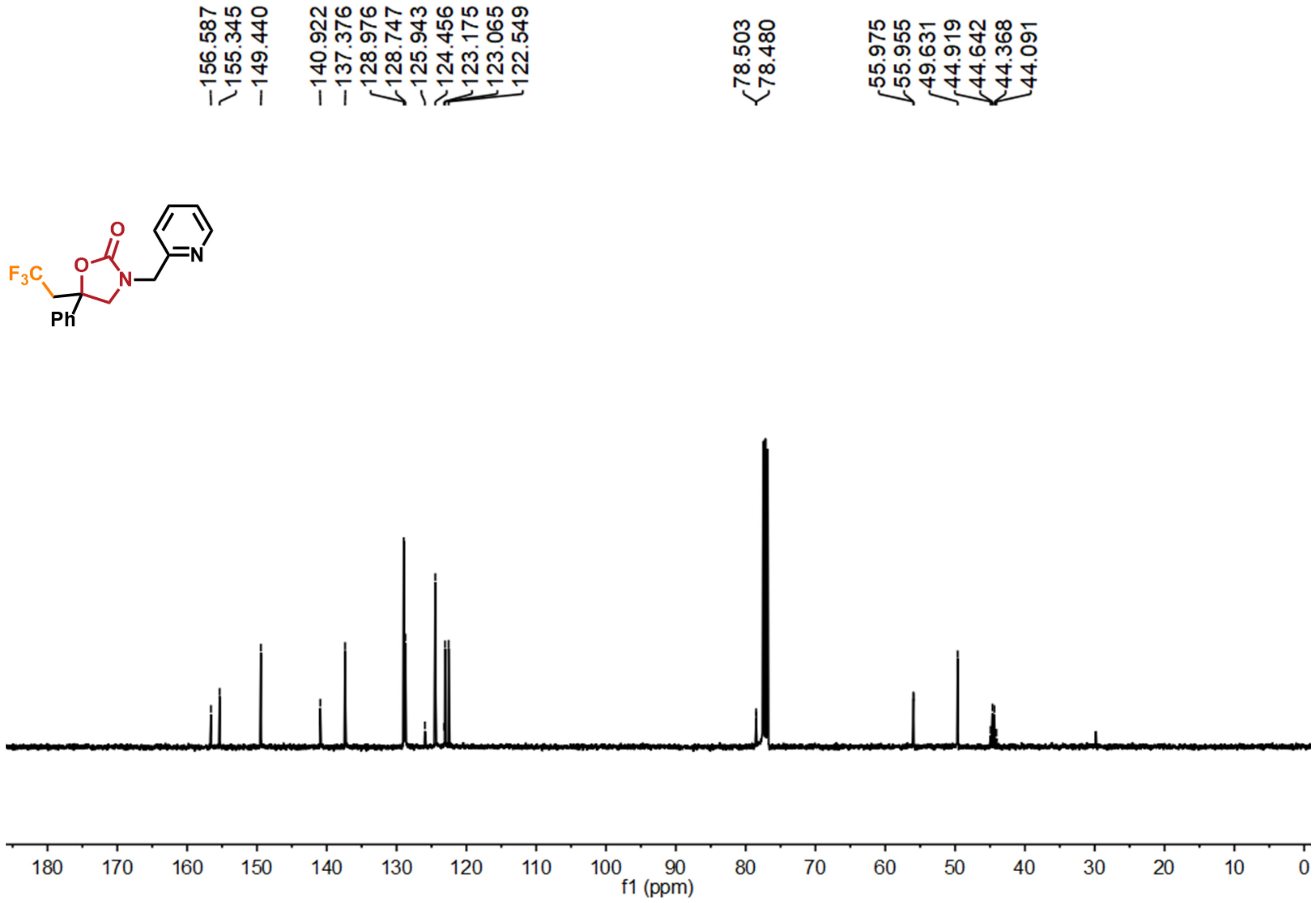

^{19}F NMR (376 MHz, $CDCl_3$) spectrum of **6j**

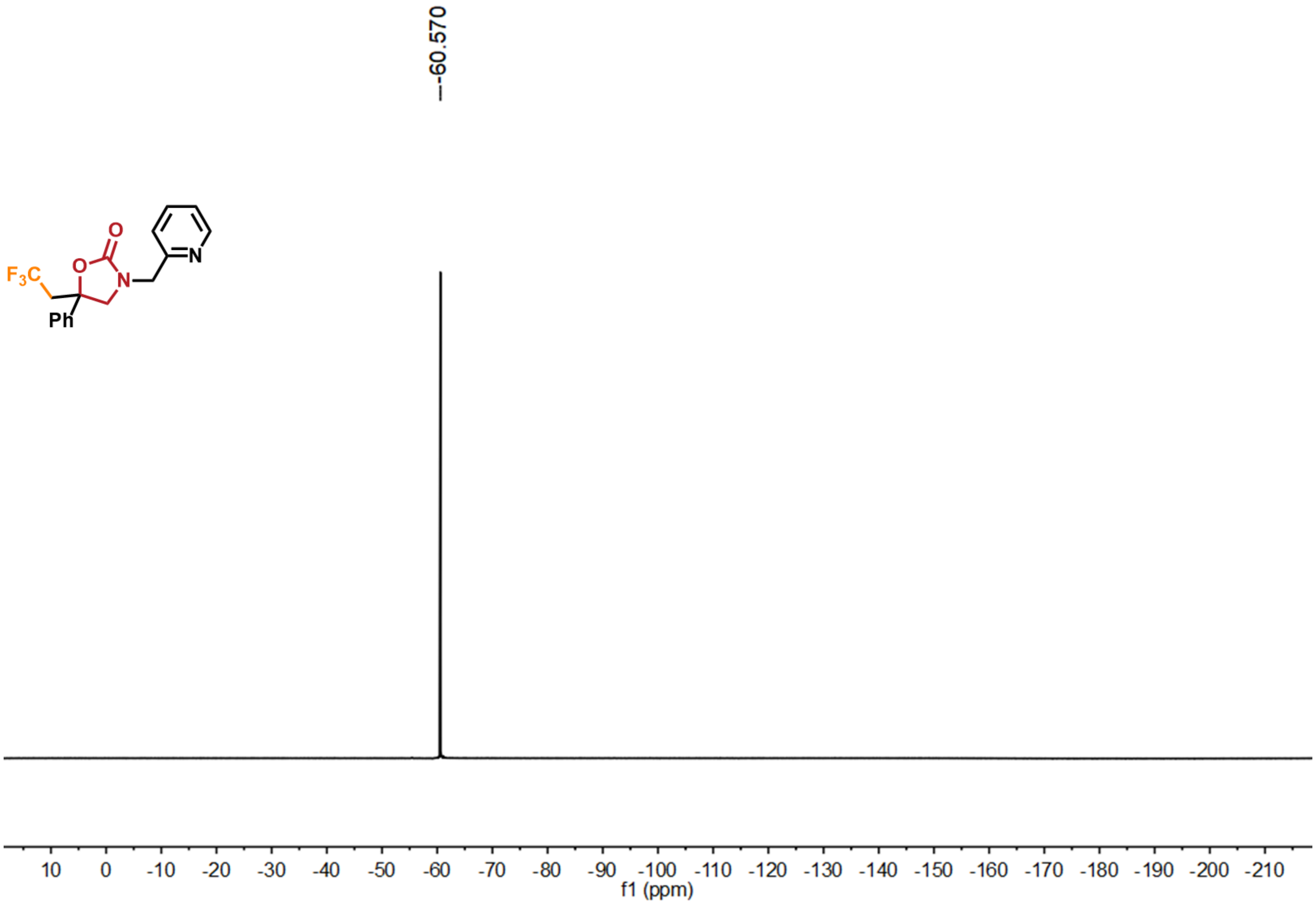

^{1}H NMR (400 MHz, $CDCl_3$) spectrum of **6k**

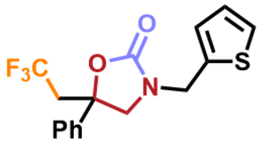

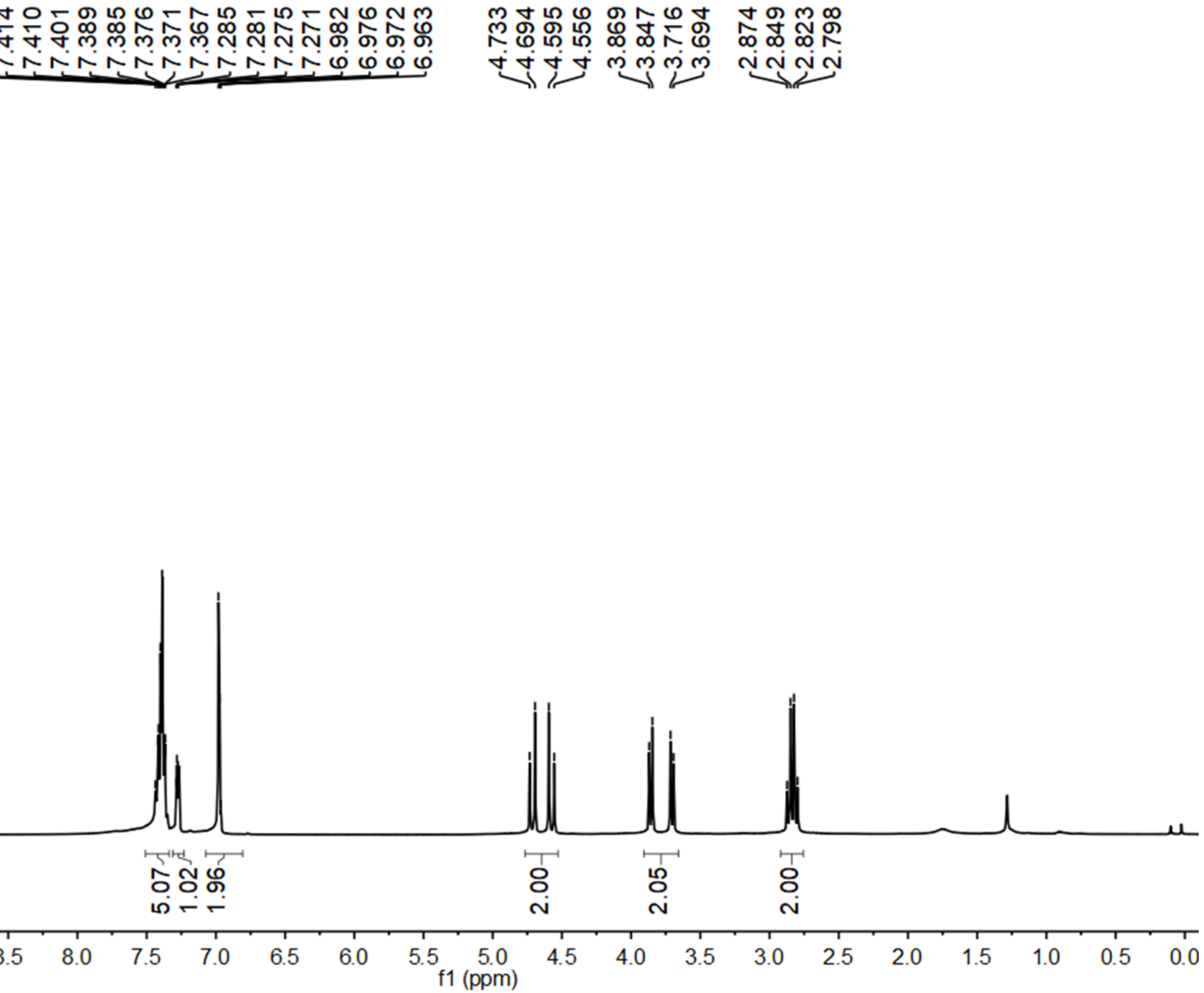

^{13}C NMR (100 MHz, $CDCl_3$) spectrum of **6k**

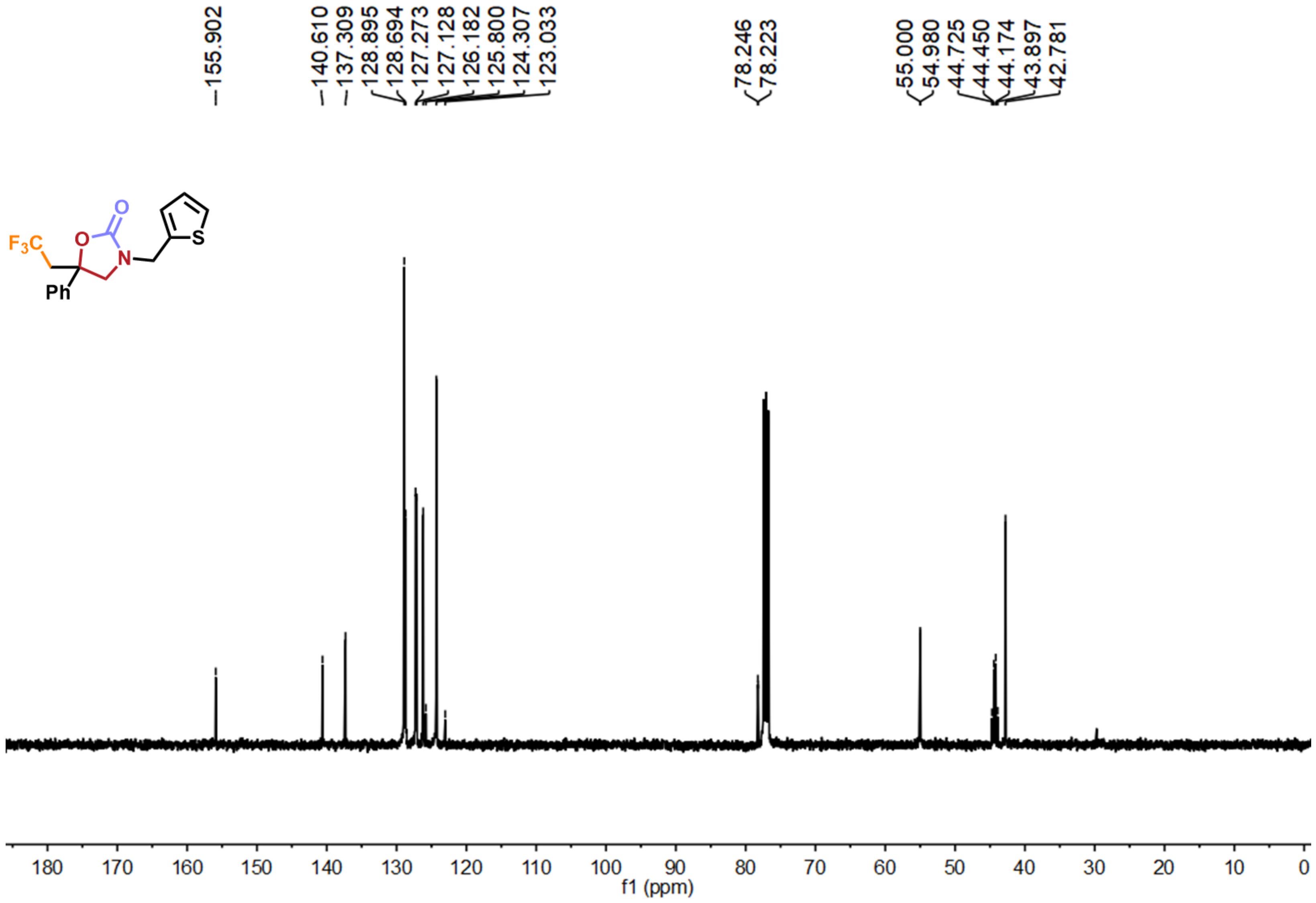

^{19}F NMR (376 MHz, $CDCl_3$) spectrum of **6k**

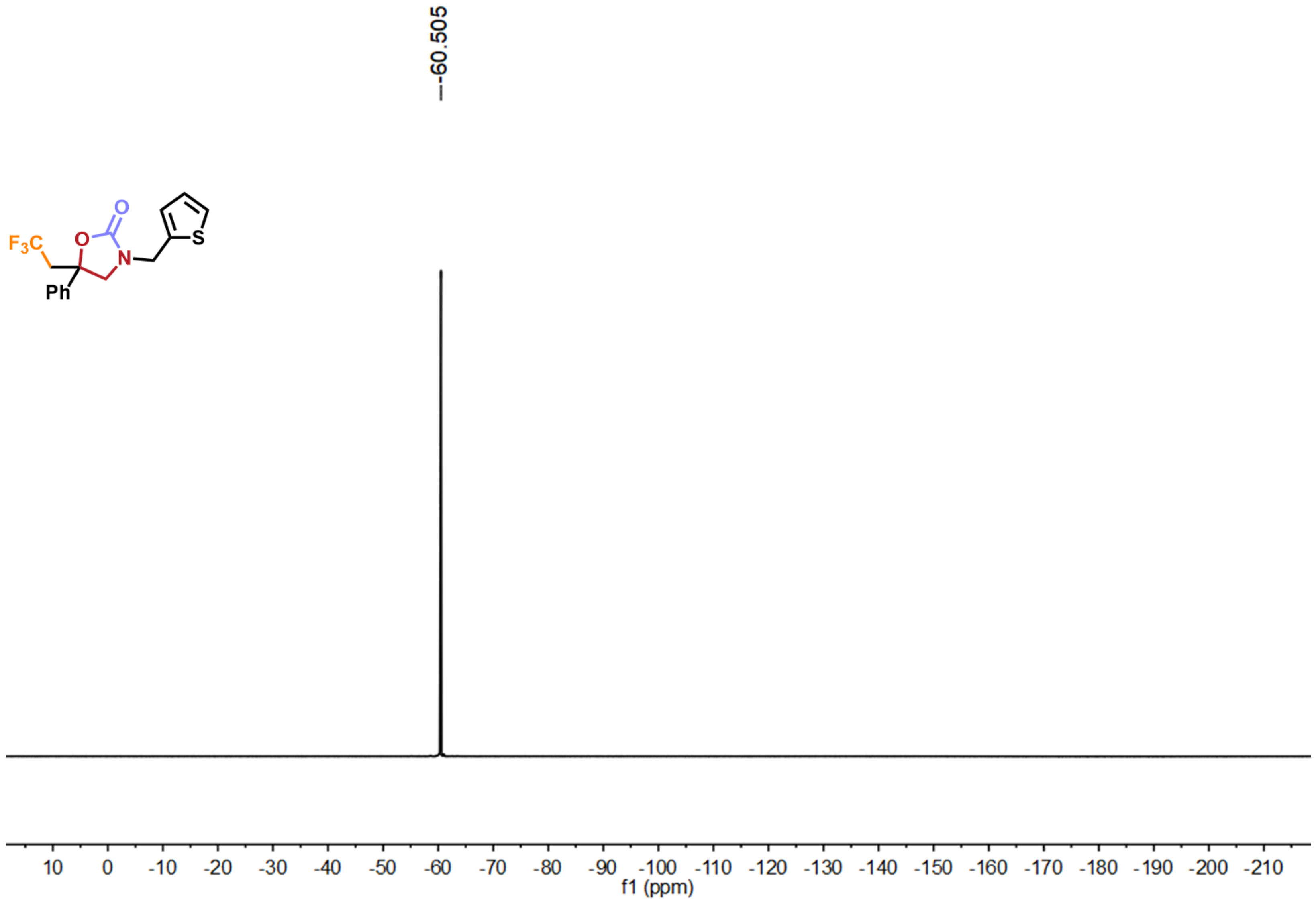

^{1}H NMR (400 MHz, $CDCl_3$) spectrum of **6l**

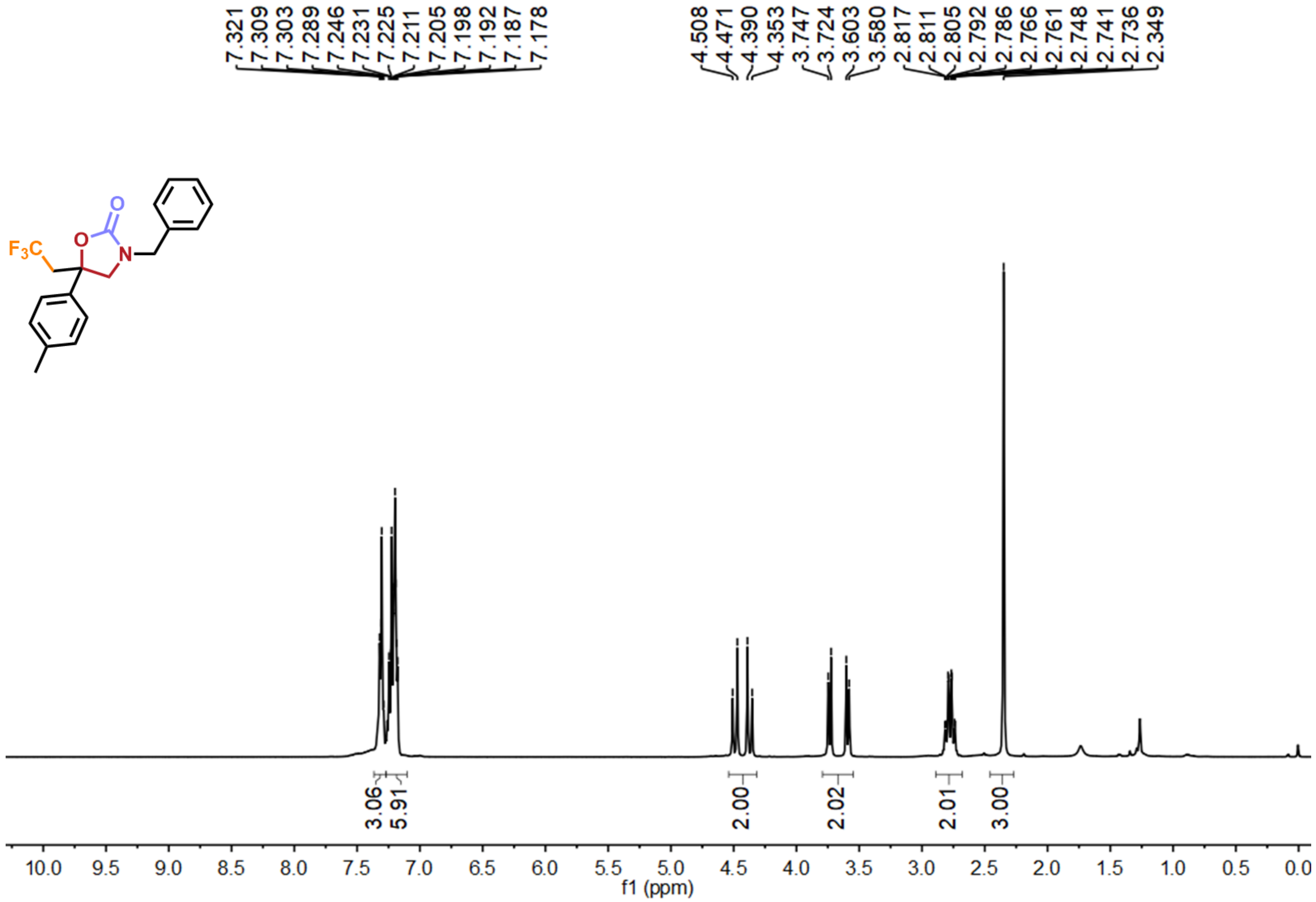

^{13}C NMR (100 MHz, $CDCl_3$) spectrum of **6l**

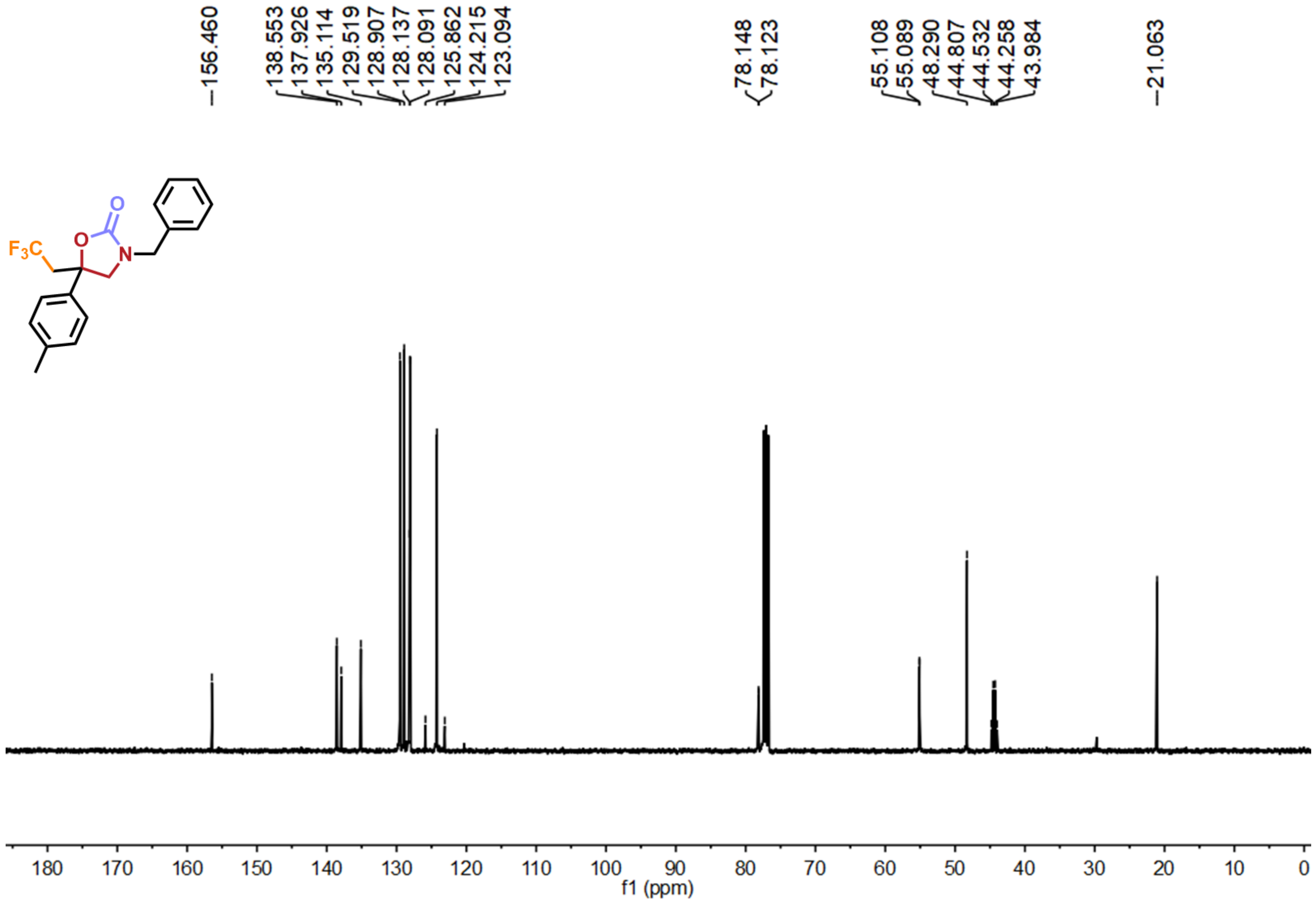

^{19}F NMR (376 MHz, $CDCl_3$) spectrum of **6l**

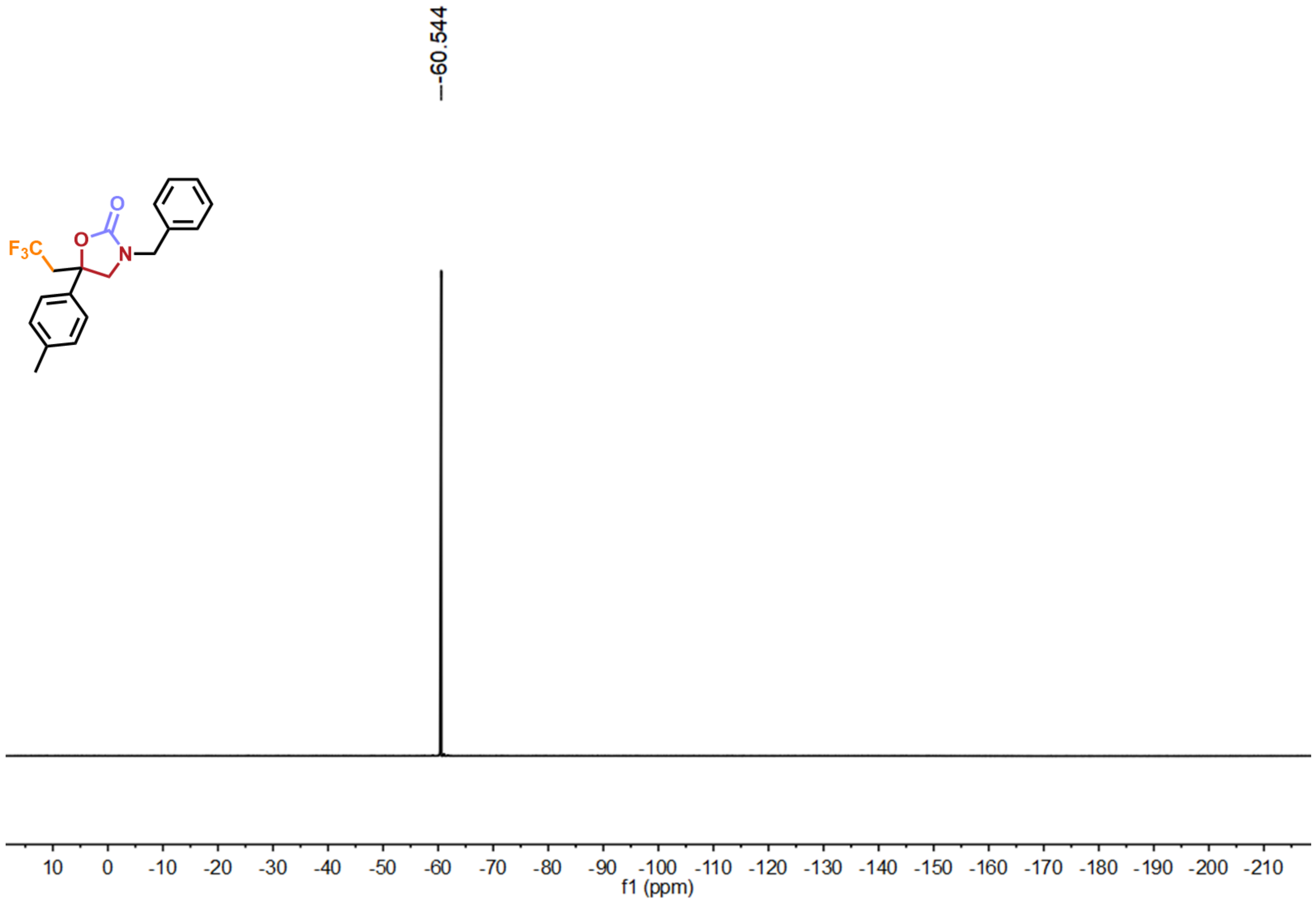

^{1}H NMR (400 MHz, $CDCl_3$) spectrum of **6m**

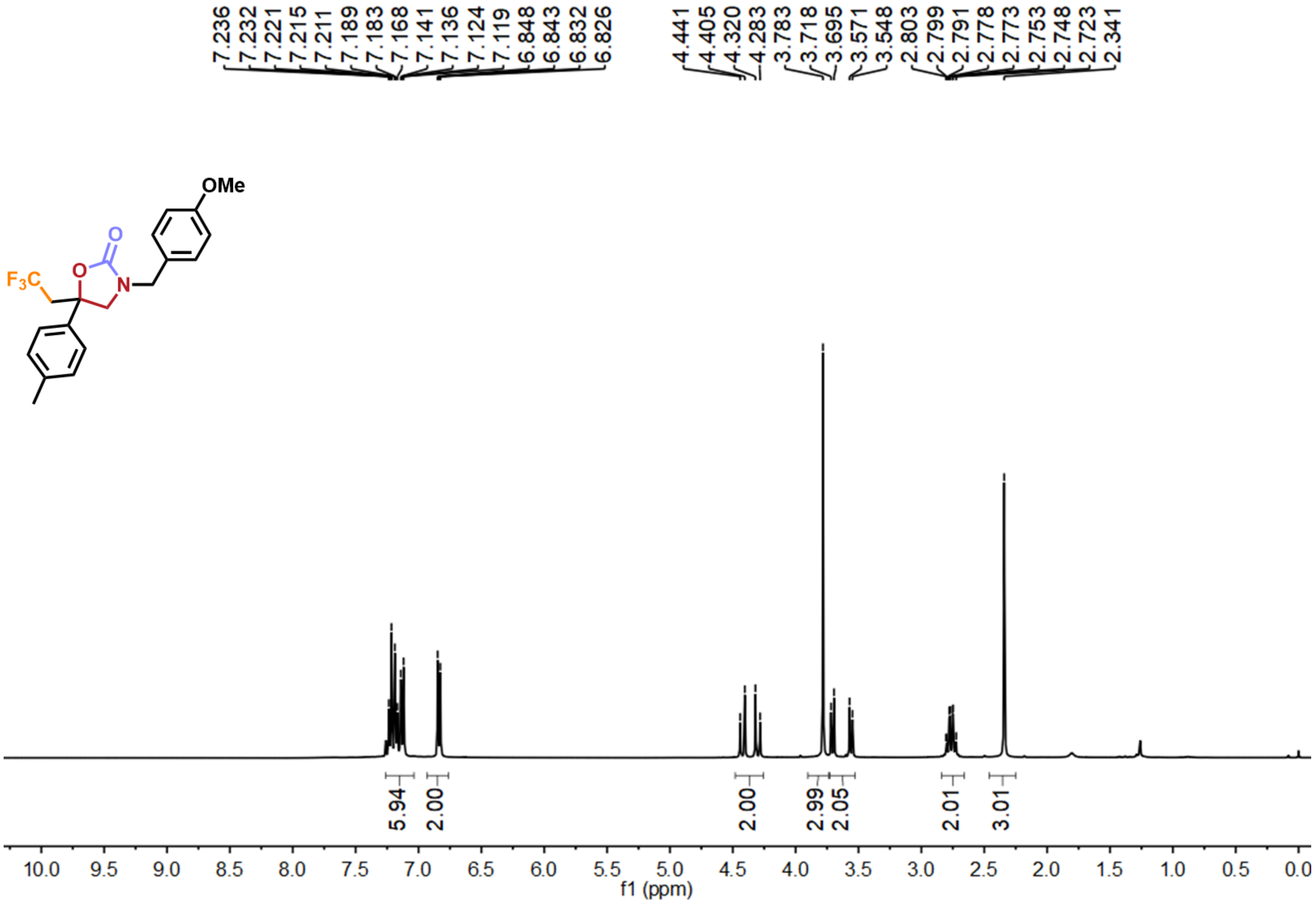

^{13}C NMR (100 MHz, $CDCl_3$) spectrum of **6m**

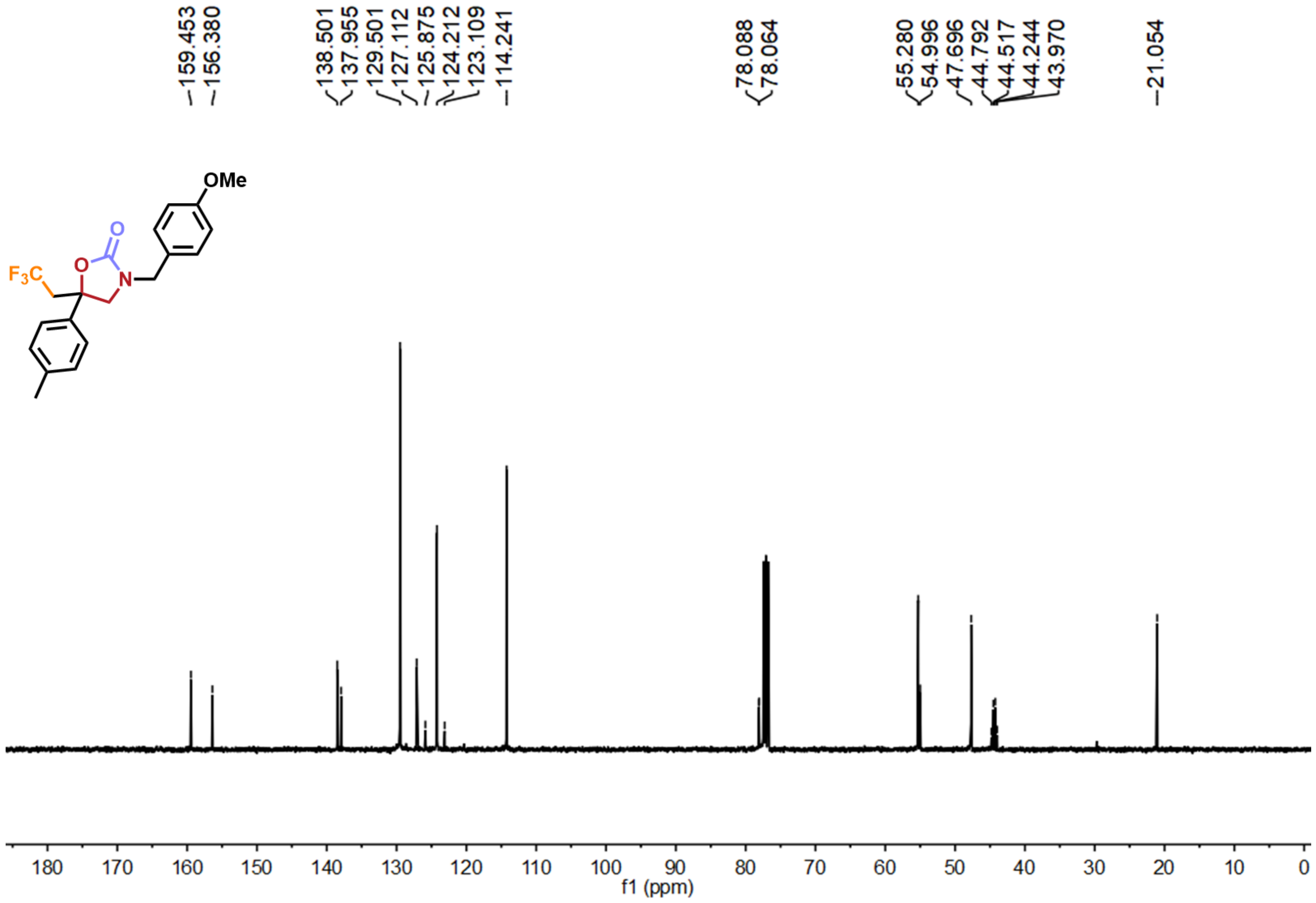

^{19}F NMR (376 MHz, $CDCl_3$) spectrum of **6m**

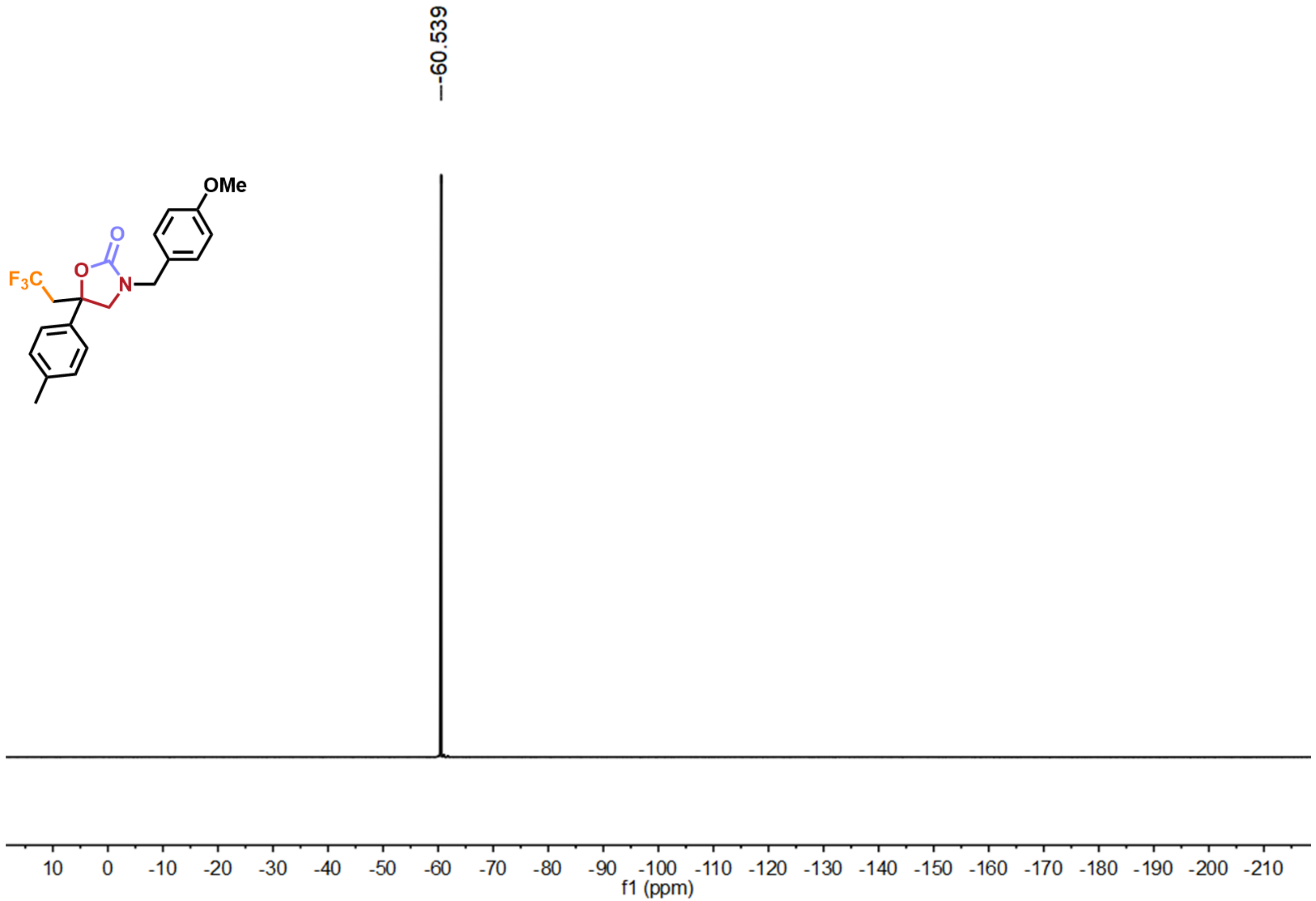

^{1}H NMR (400 MHz, $CDCl_3$) spectrum of **6n**

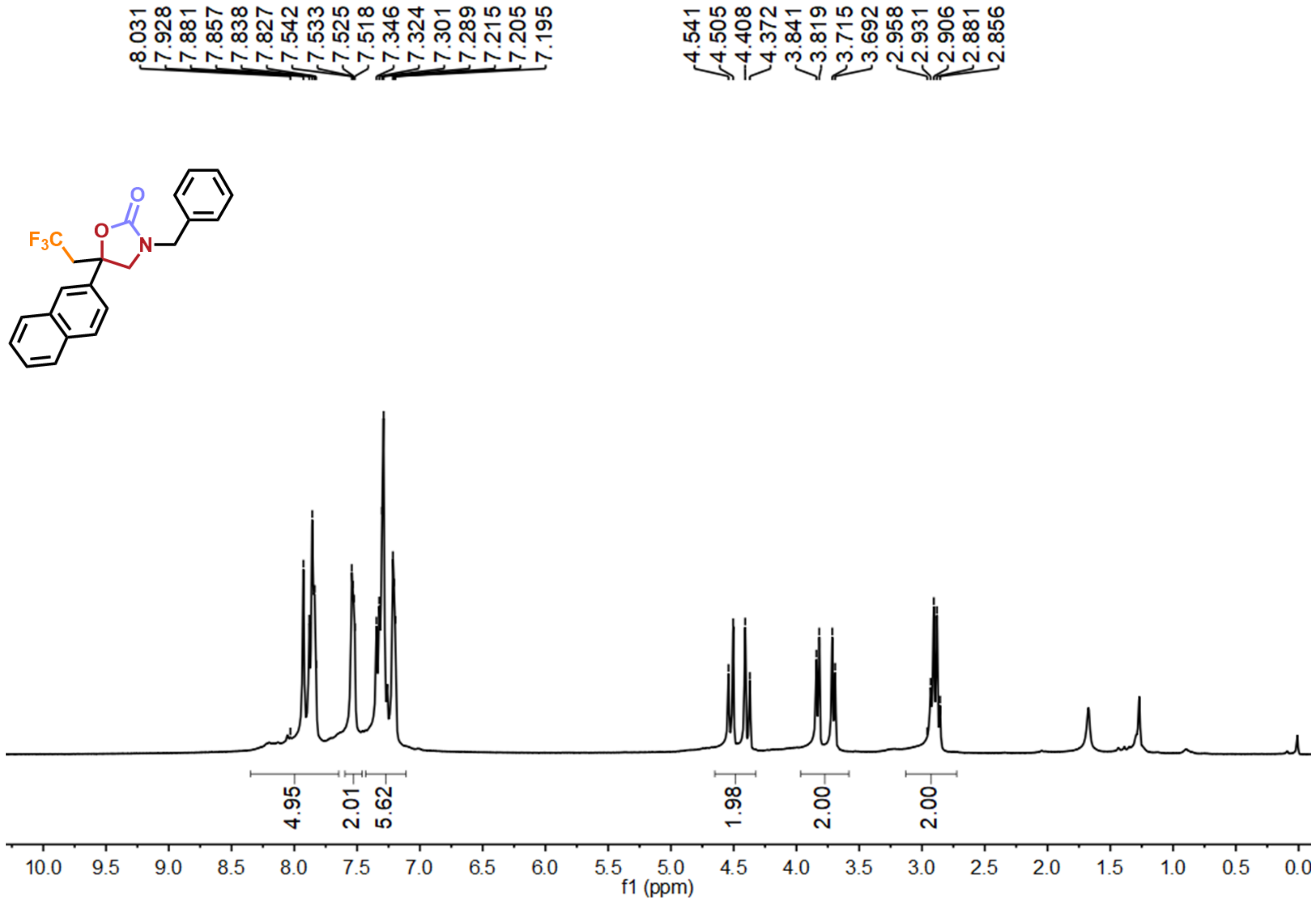

^{13}C NMR (100 MHz, $CDCl_3$) spectrum of **6n**

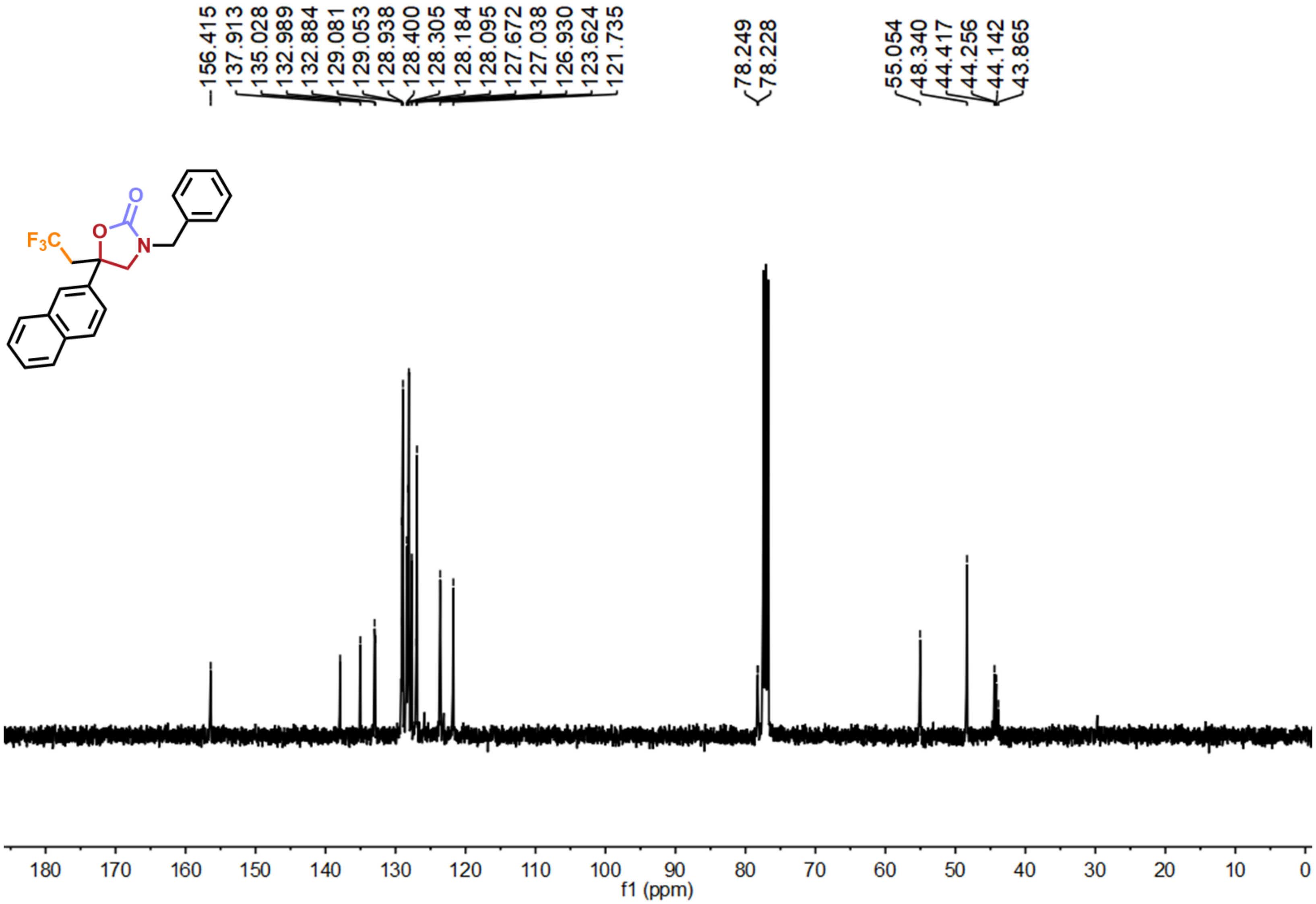

^{19}F NMR (376 MHz, $CDCl_3$) spectrum of **6n**

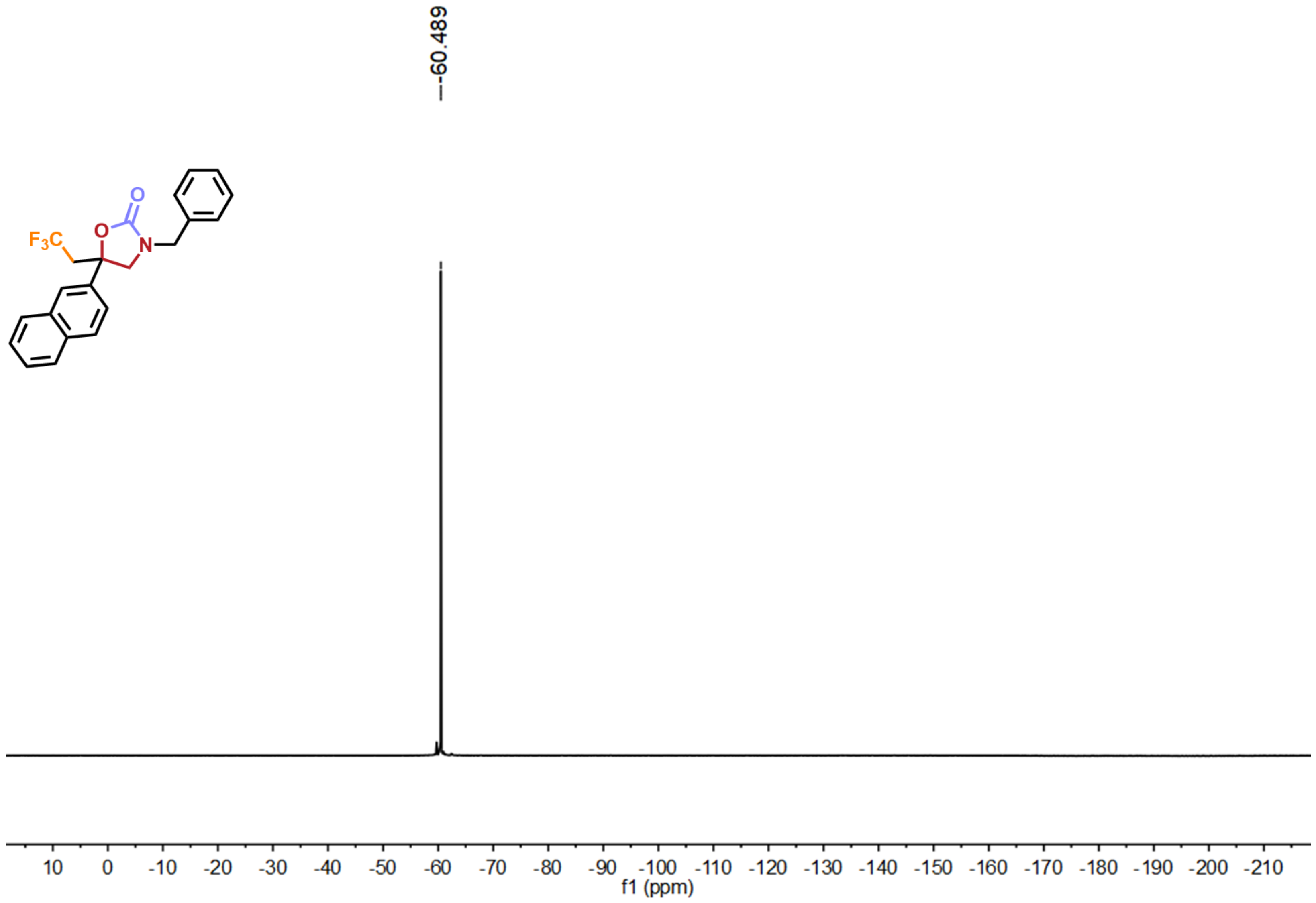

^{1}H NMR (400 MHz, $CDCl_3$) spectrum of **6o**

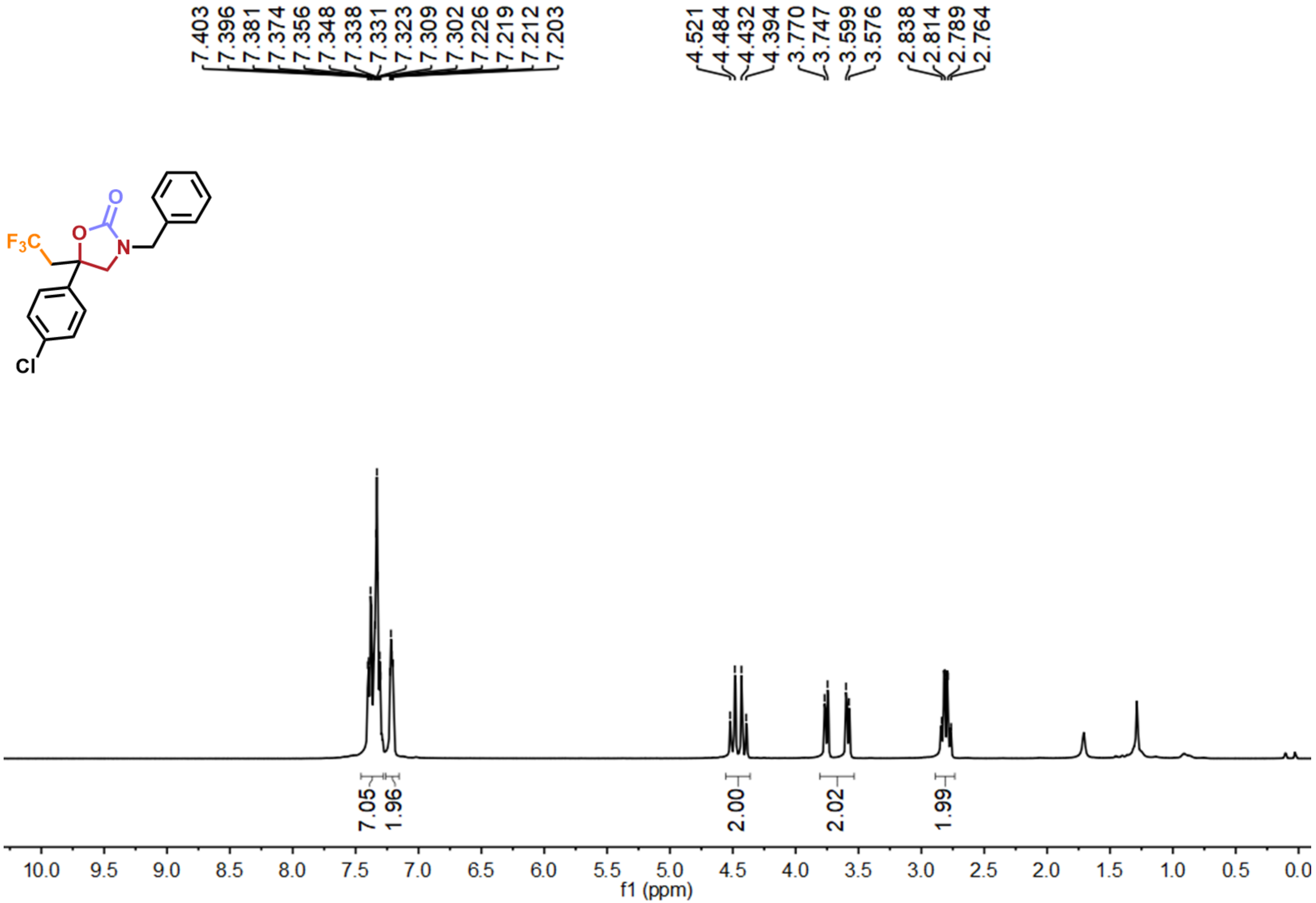

^{13}C NMR (100 MHz, $CDCl_3$) spectrum of **6o**

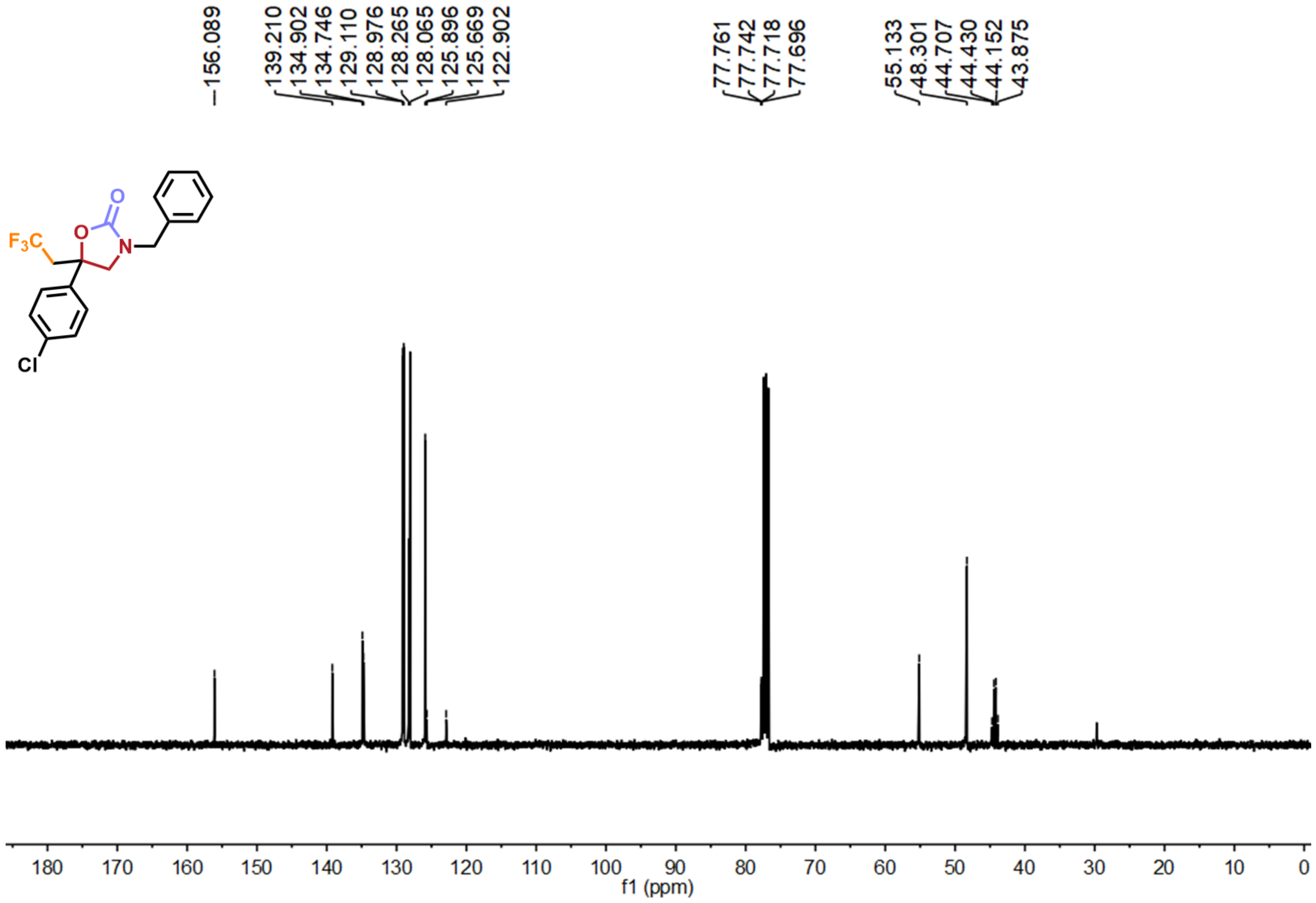

^{19}F NMR (376 MHz, $CDCl_3$) spectrum of **6o**

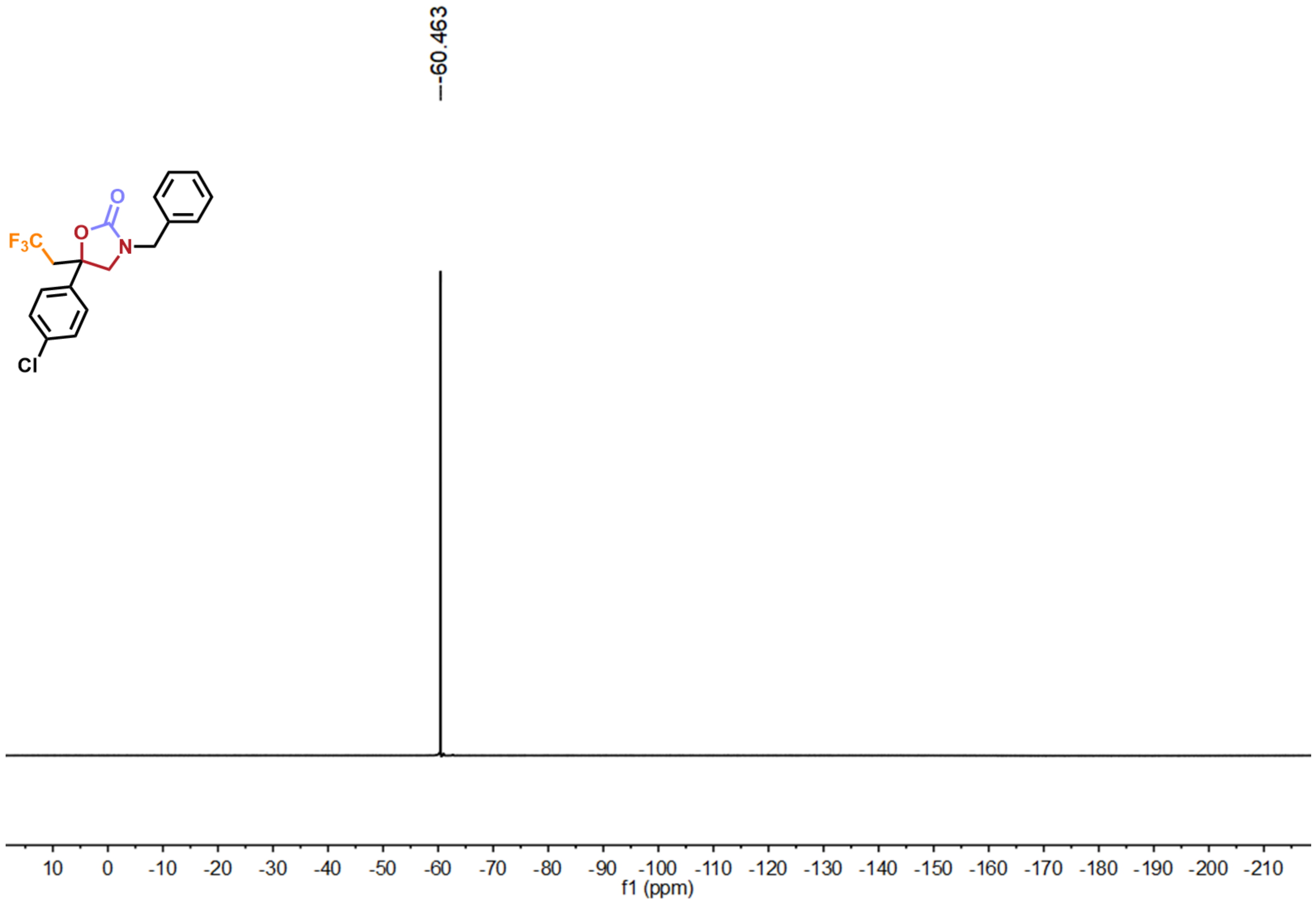

^{1}H NMR (400 MHz, $CDCl_3$) spectrum of **6p**

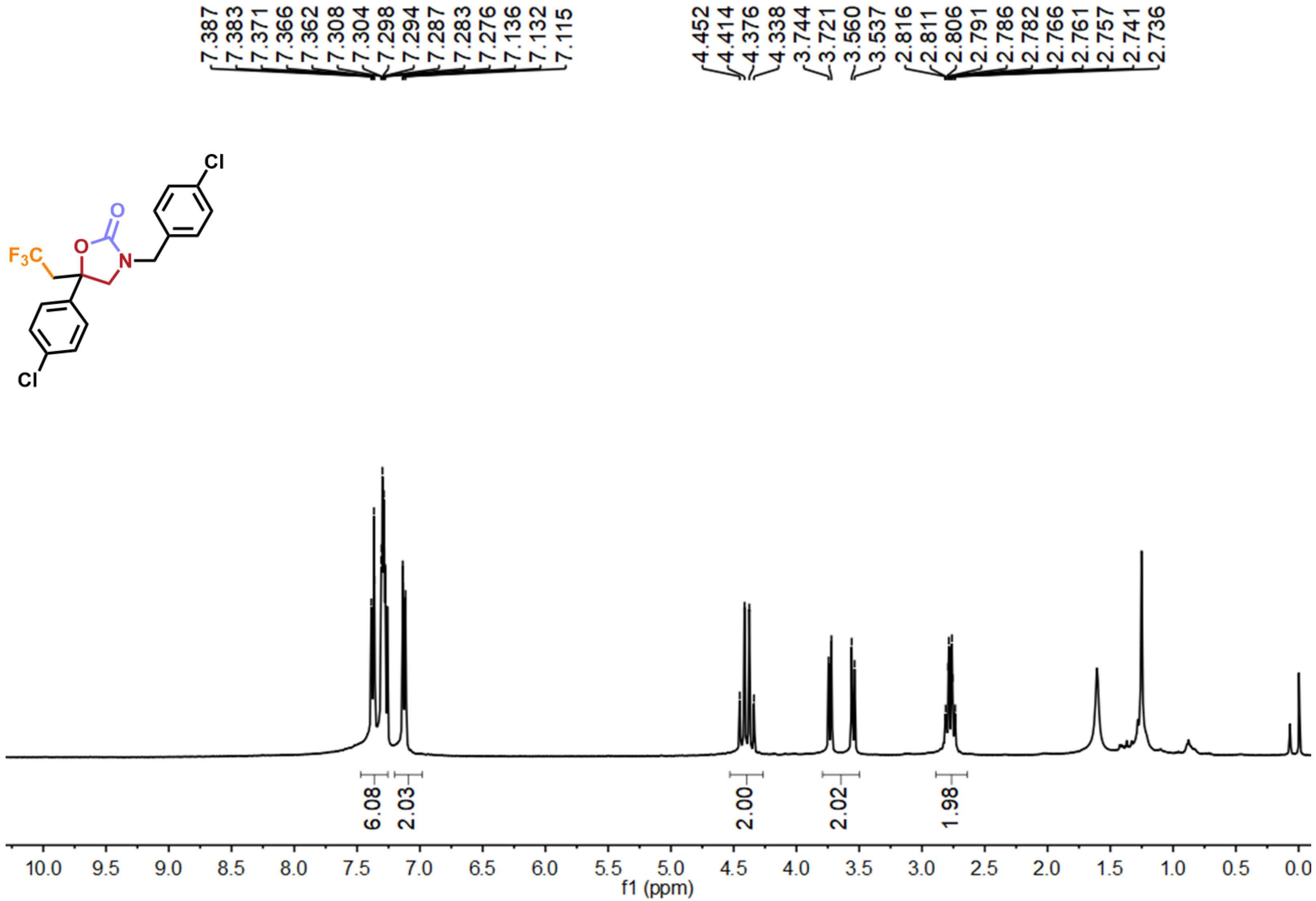

^{13}C NMR (100 MHz, $CDCl_3$) spectrum of **6p**

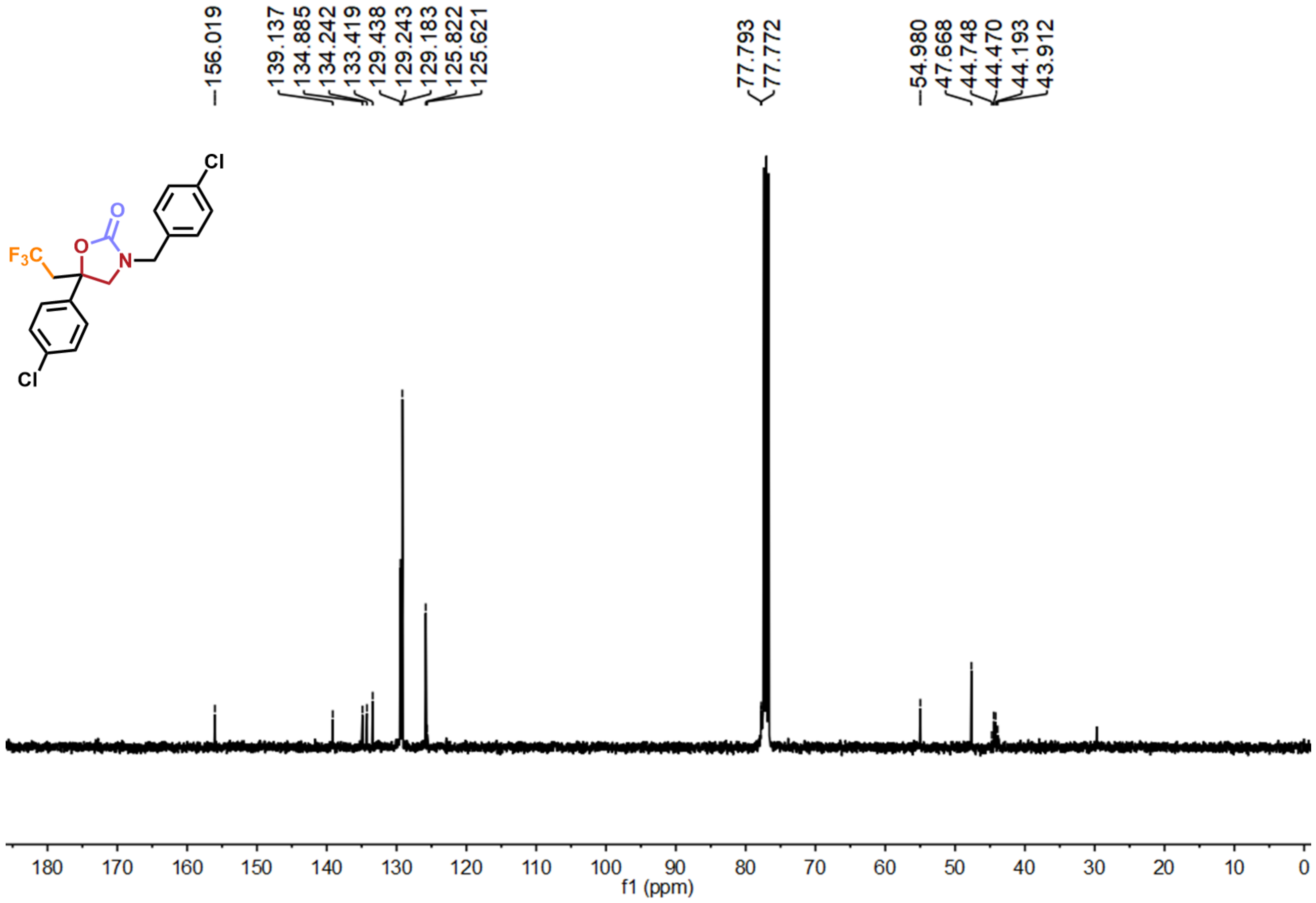

^{19}F NMR (376 MHz, $CDCl_3$) spectrum of **6p**

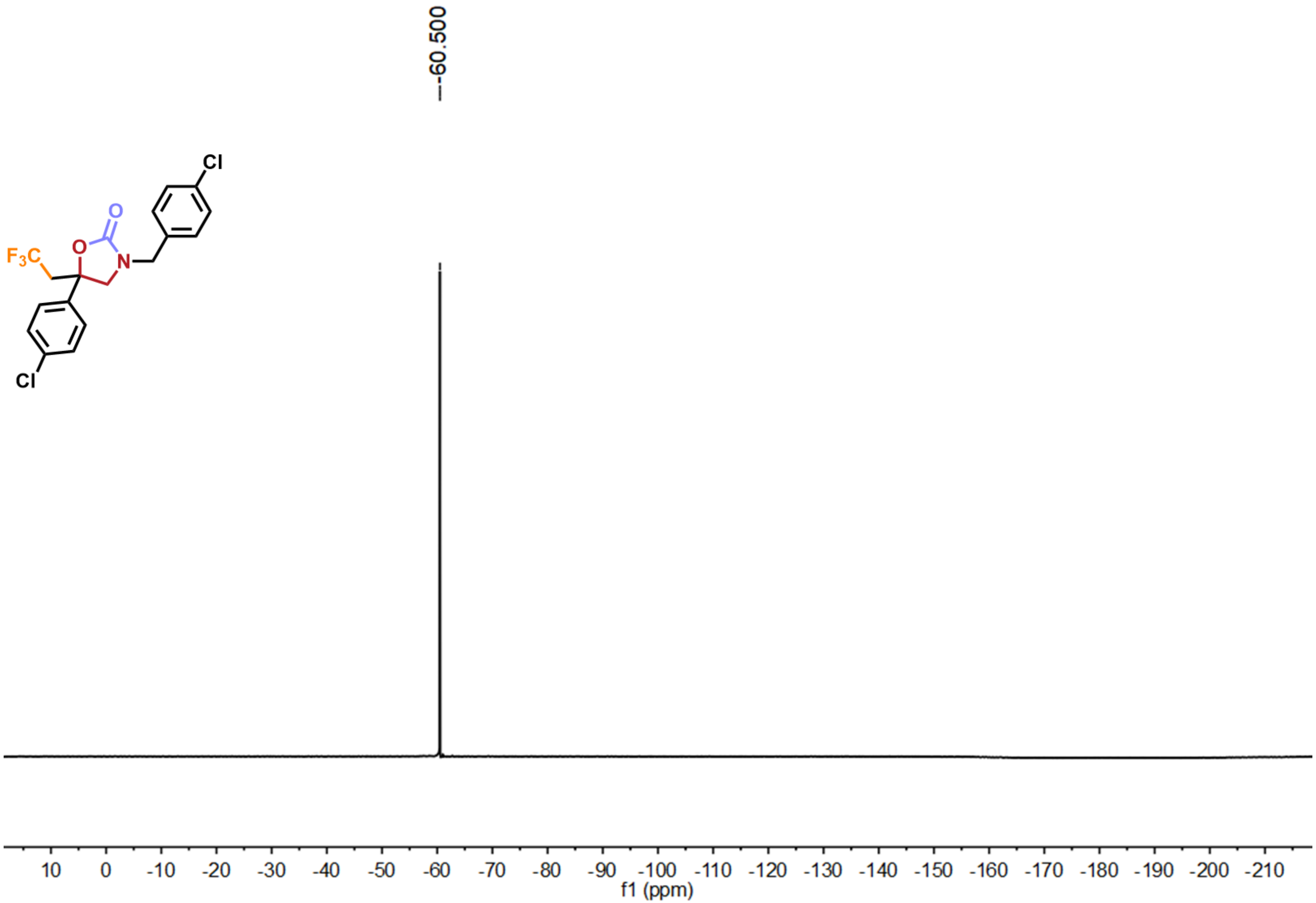

^{1}H NMR (400 MHz, $CDCl_3$) spectrum of **6q**

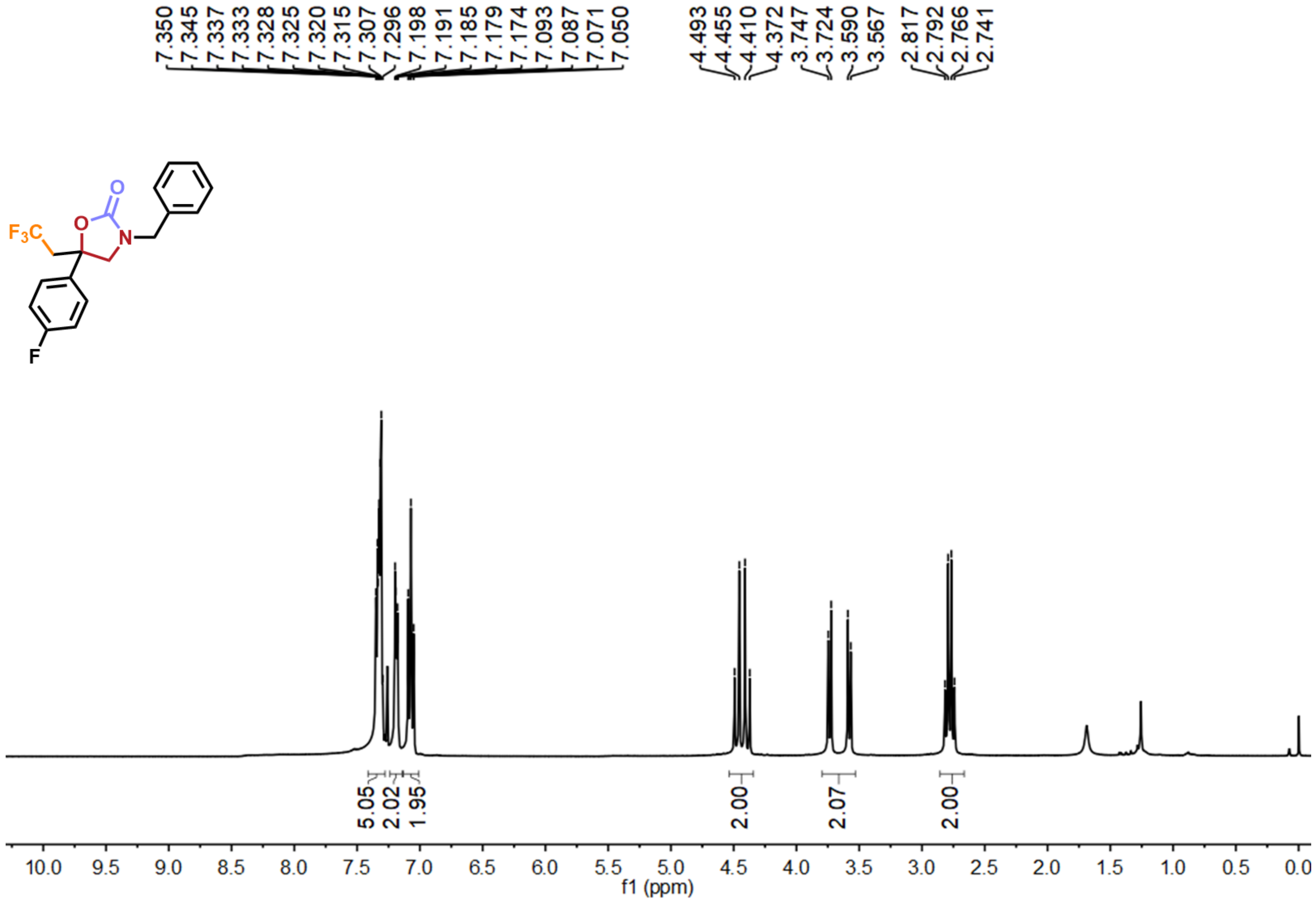

^{13}C NMR (100 MHz, $CDCl_3$) spectrum of **6q**

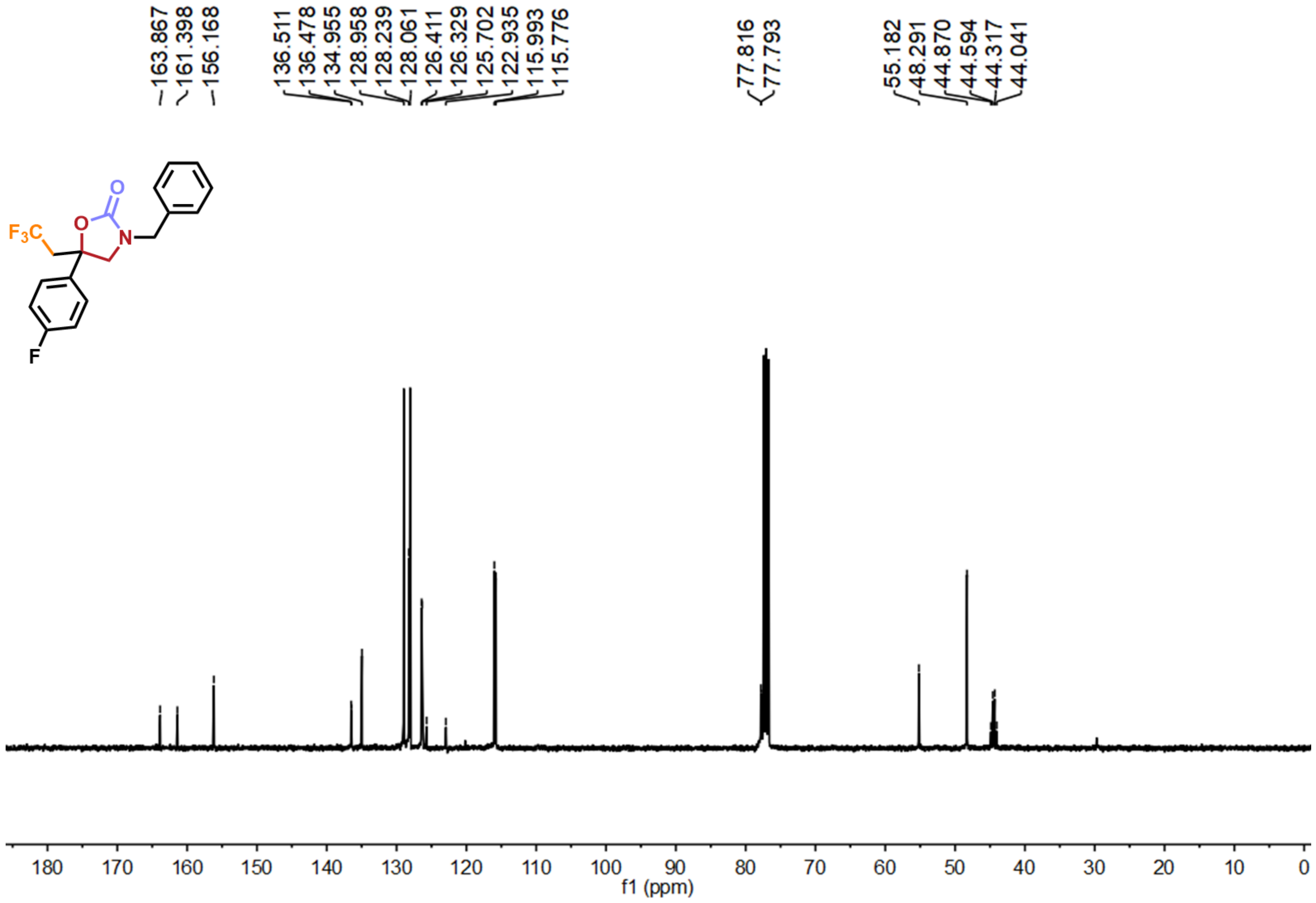

^{19}F NMR (376 MHz, $CDCl_3$) spectrum of **6q**

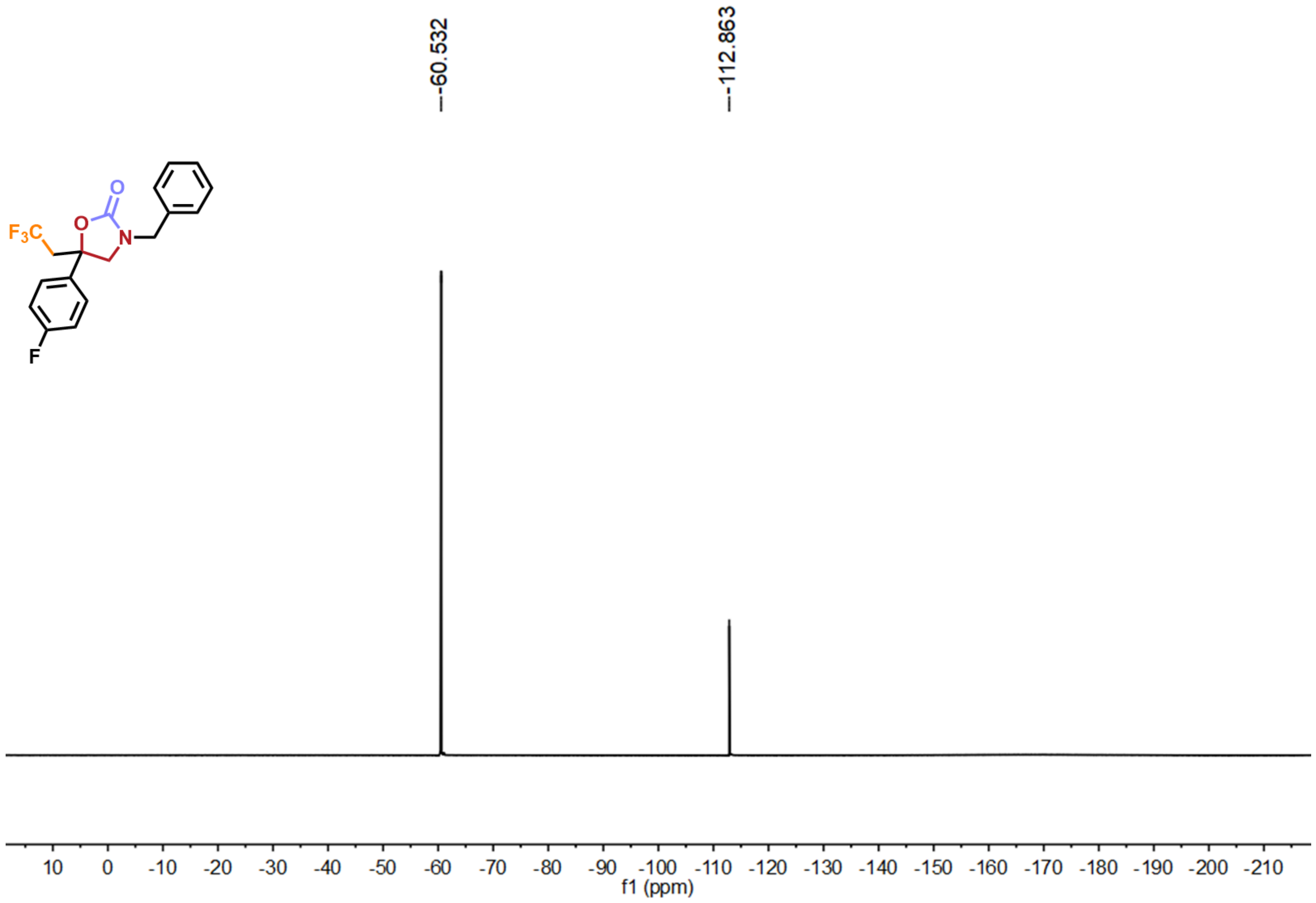

^{1}H NMR (400 MHz, $CDCl_3$) spectrum of **6r**

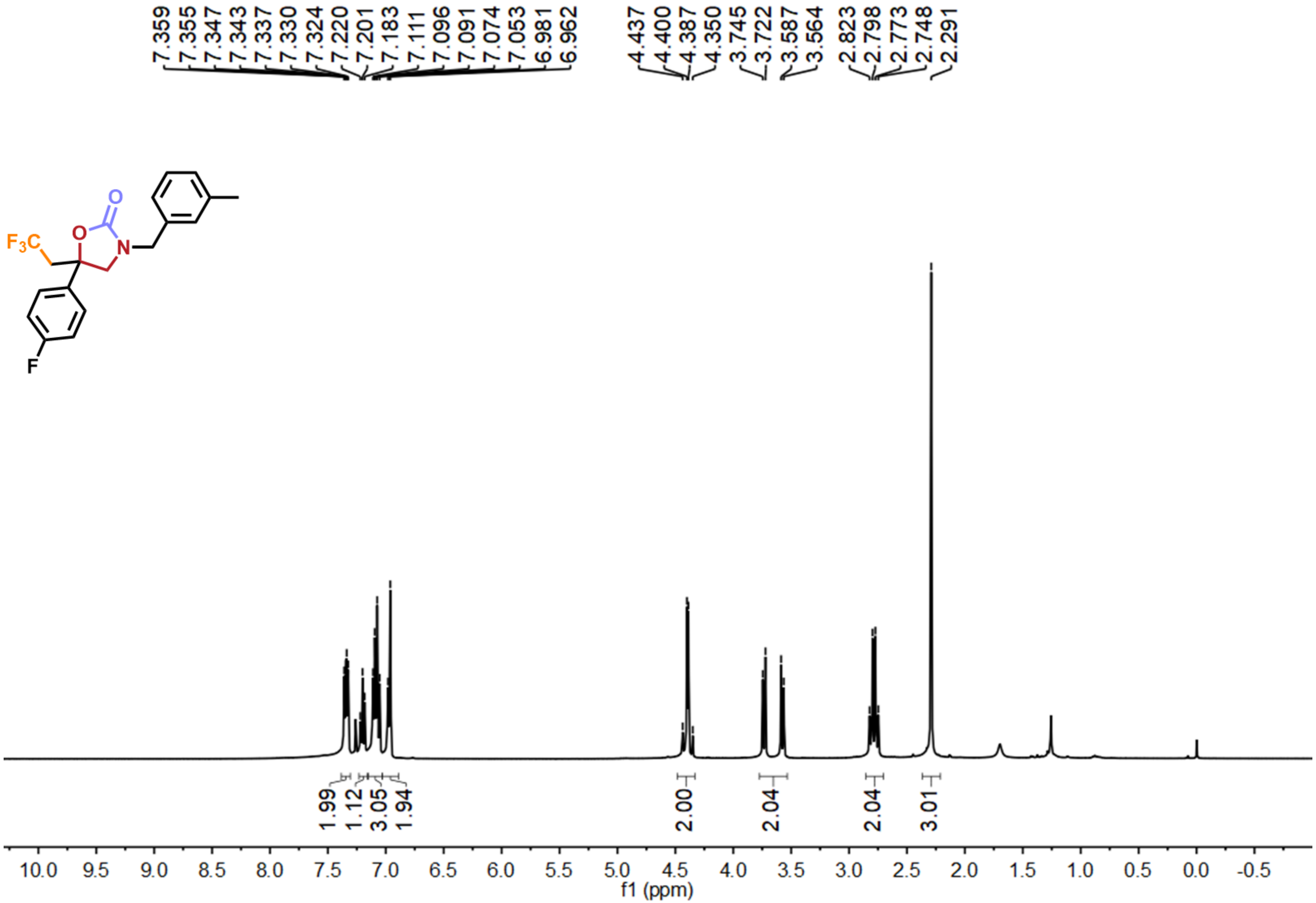

^{13}C NMR (100 MHz, $CDCl_3$) spectrum of **6r**

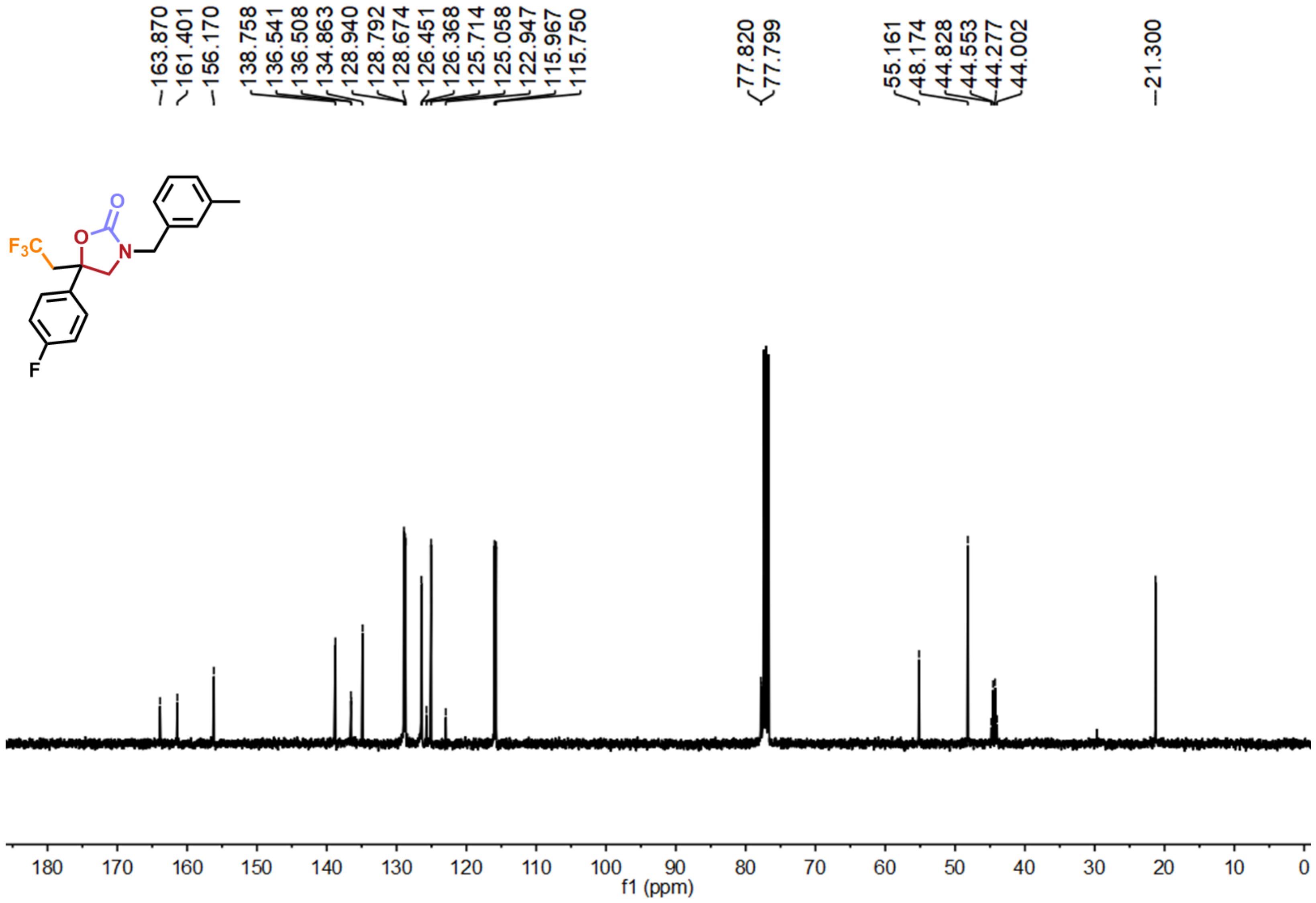

^{19}F NMR (376 MHz, $CDCl_3$) spectrum of **6r**

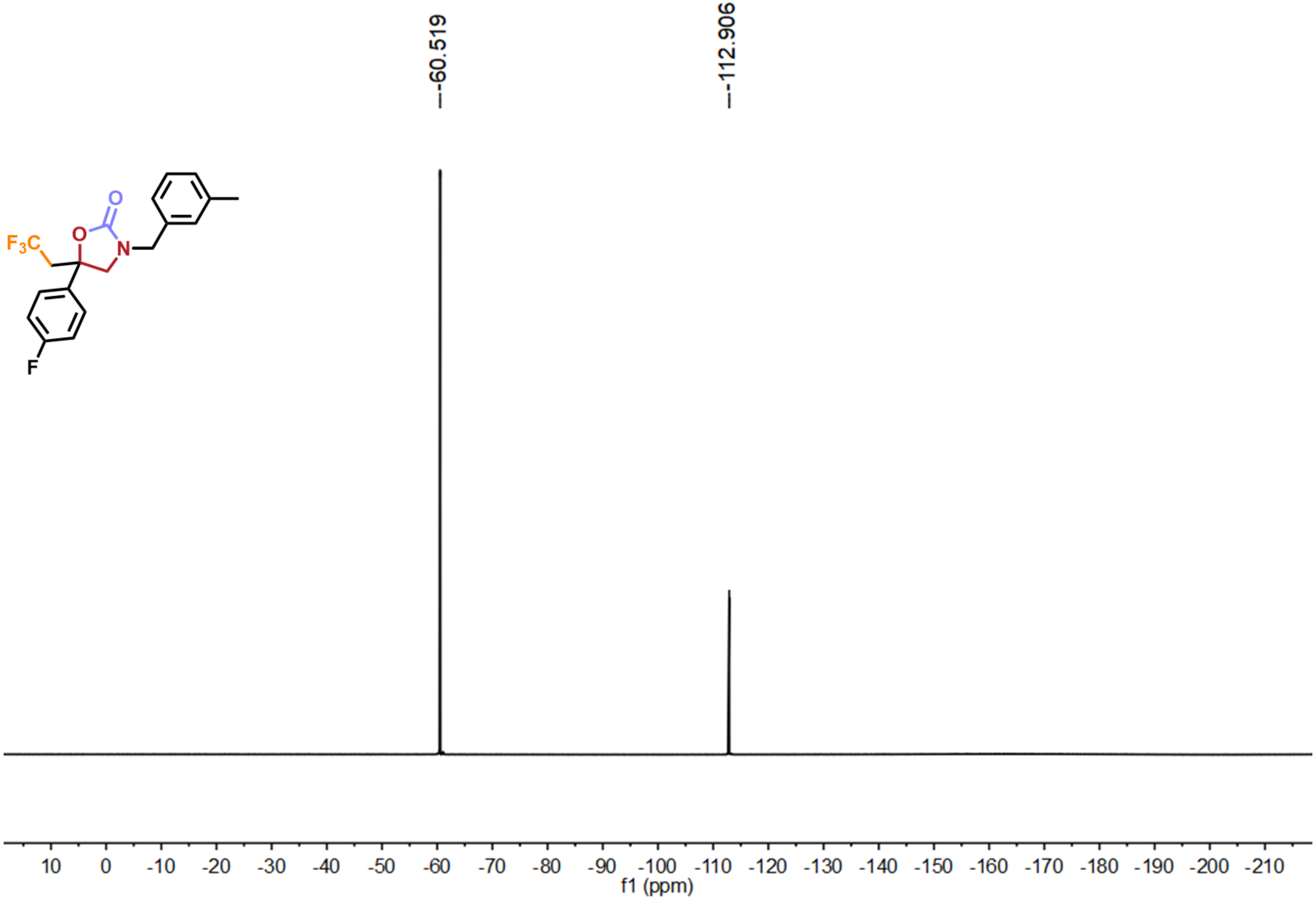

^{1}H NMR (400 MHz, $CDCl_3$) spectrum of **6s**

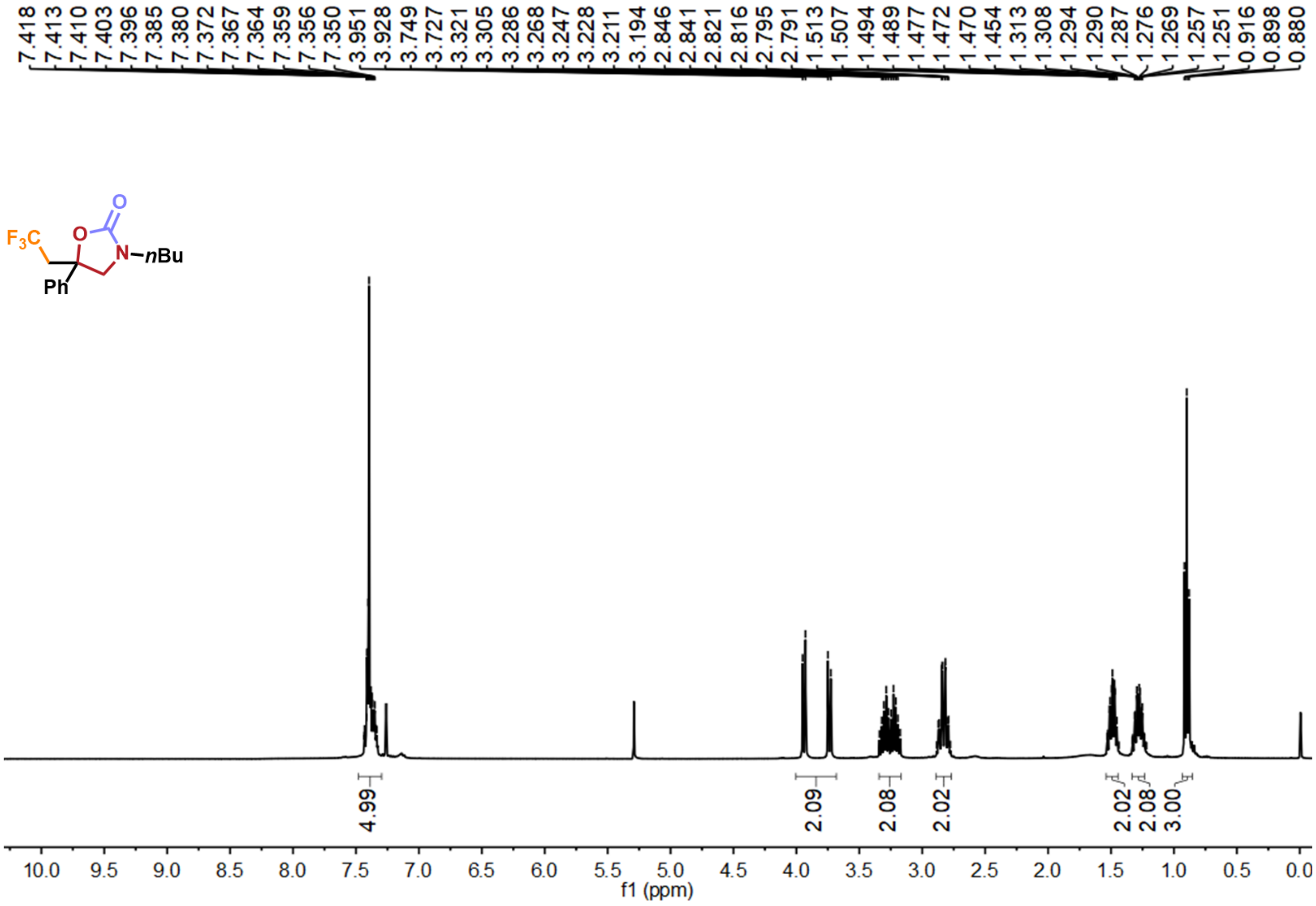

^{13}C NMR (100 MHz, $CDCl_3$) spectrum of **6s**

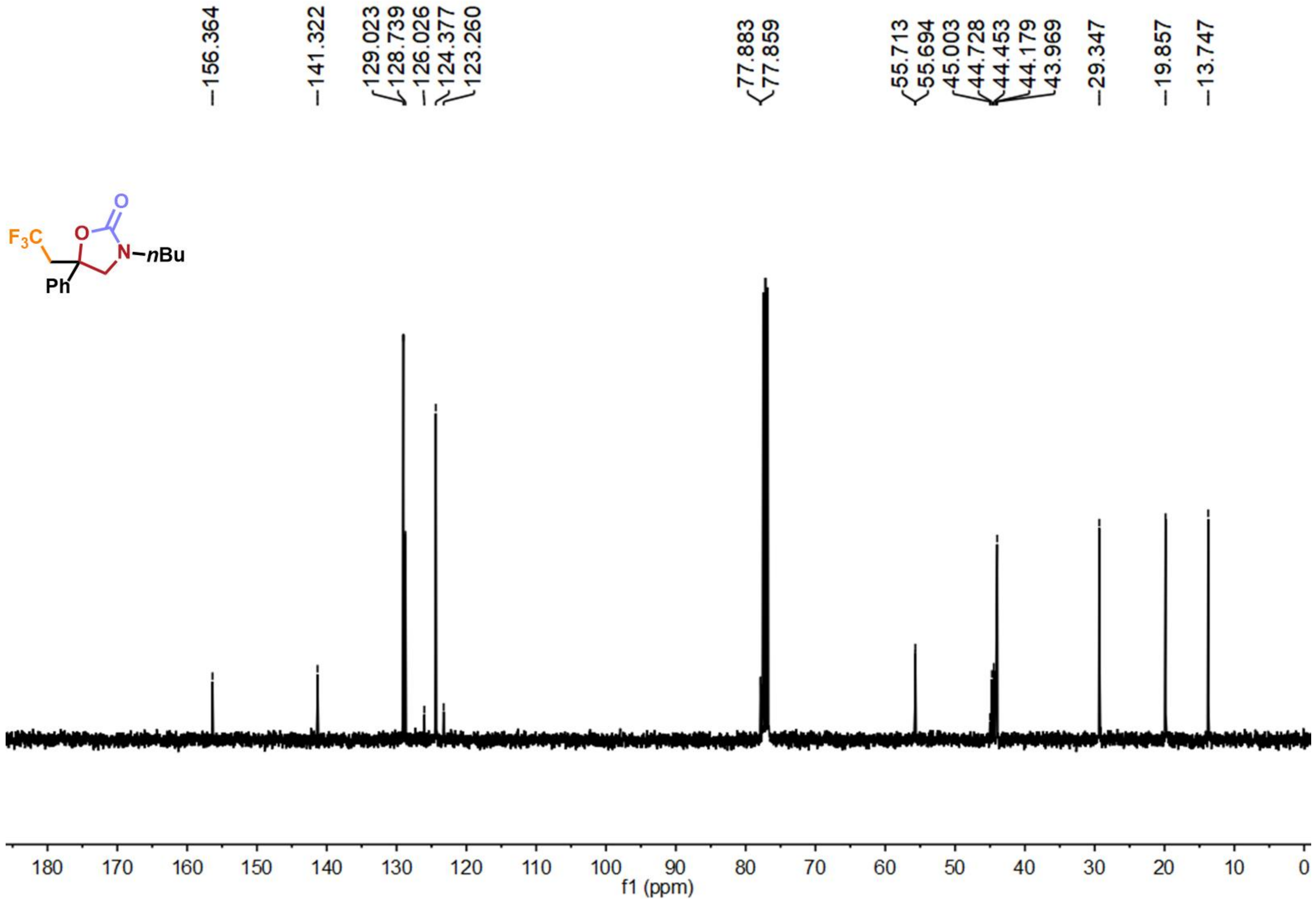

^{19}F NMR (376 MHz, $CDCl_3$) spectrum of **6s**

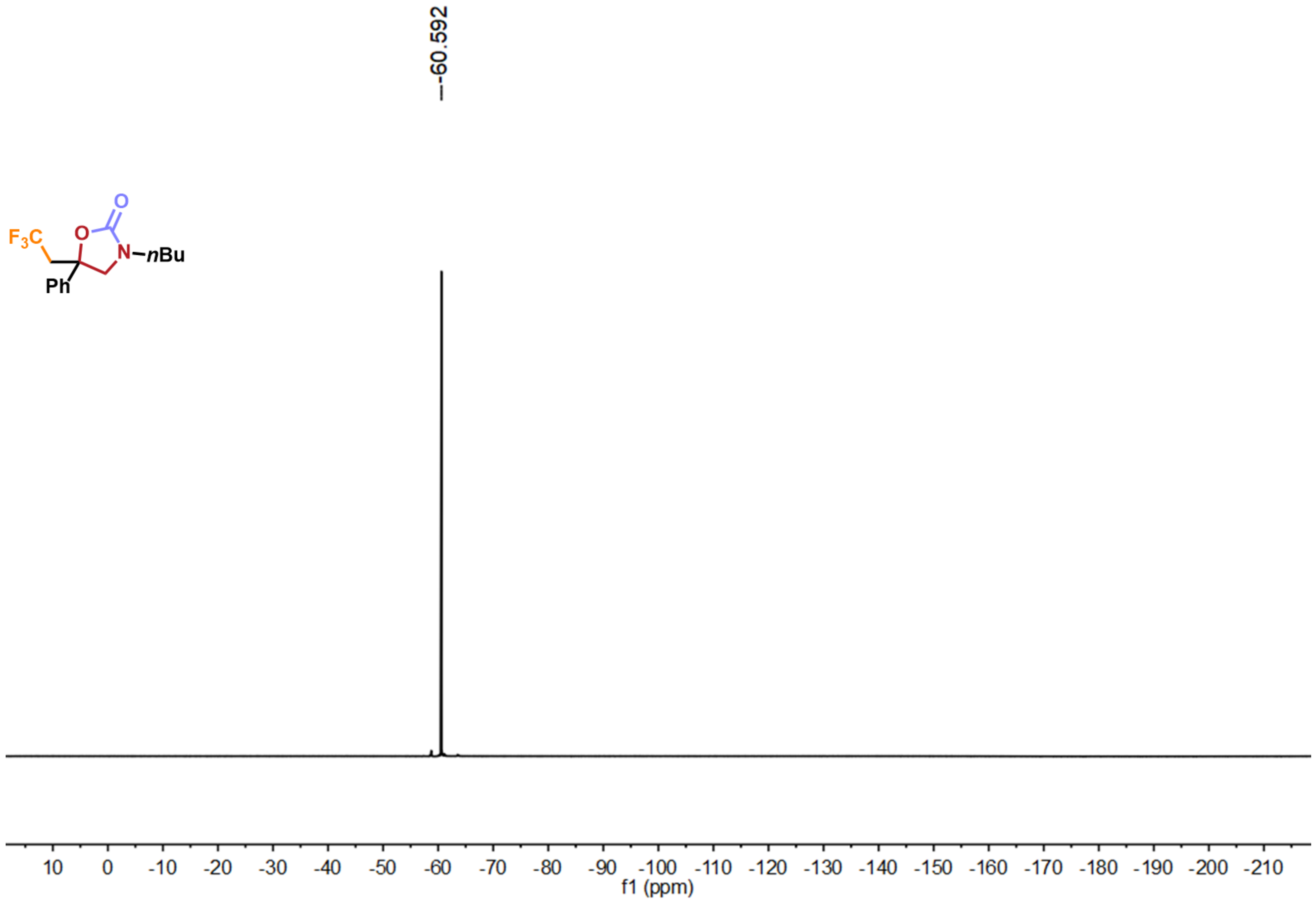

^{1}H NMR (400 MHz, $CDCl_3$) spectrum of **6t**

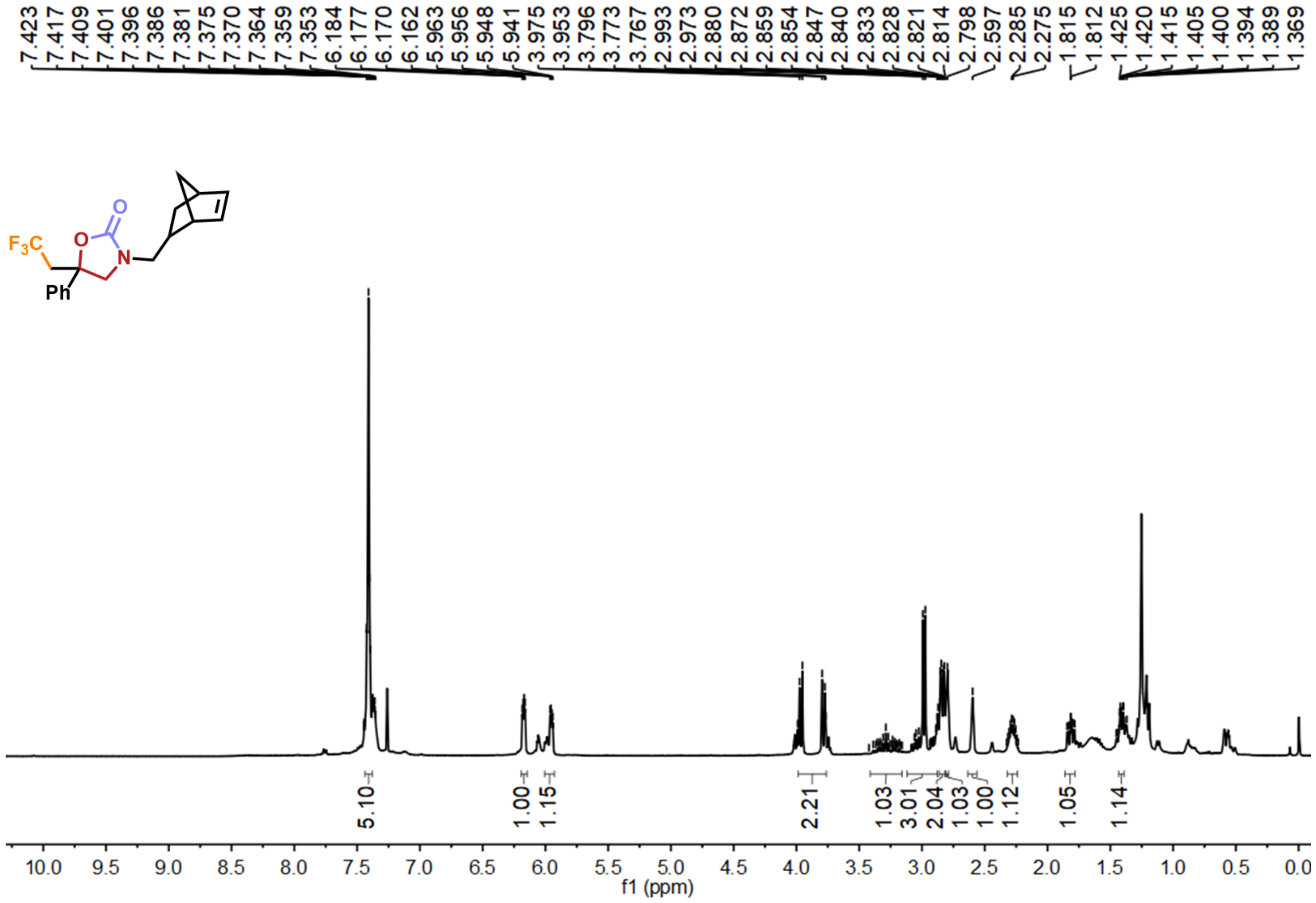

^{13}C NMR (100 MHz, $CDCl_3$) spectrum of **6t**

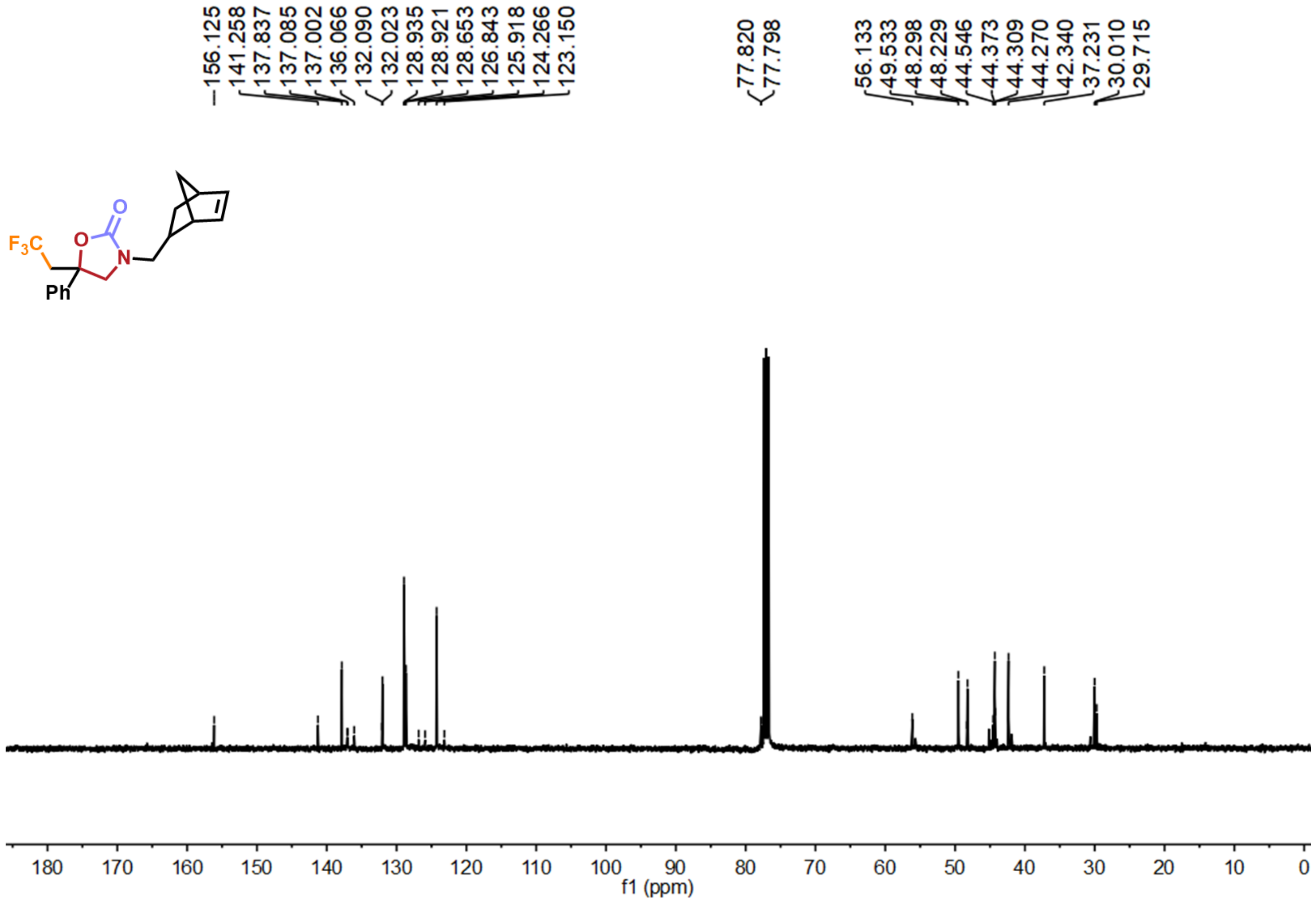

^{19}F NMR (376 MHz, $CDCl_3$) spectrum of **6t**

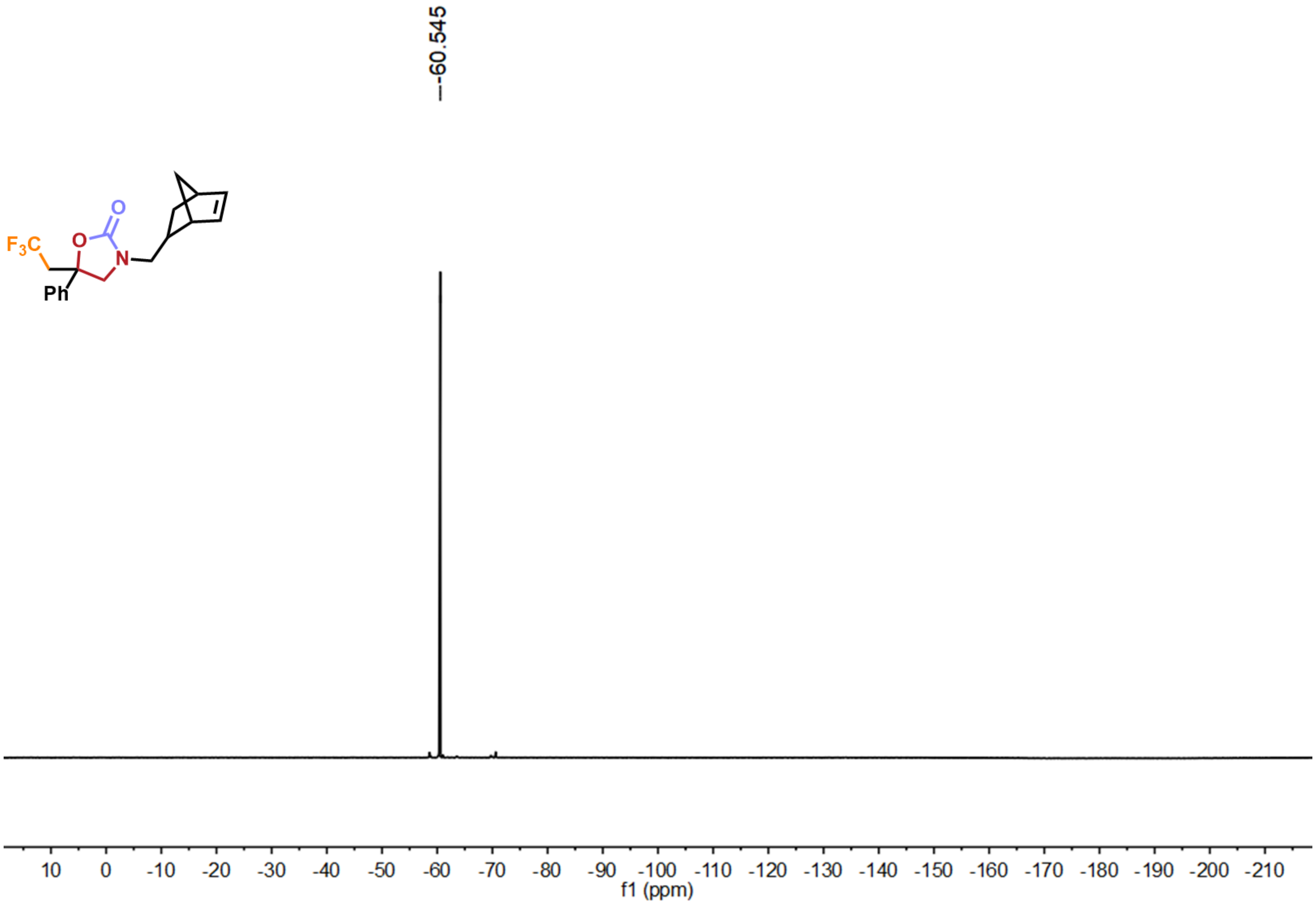

References

(1) Cheng, Y.; Li, Y. X.; Liu, C. H.; Zhu, Y. Y.; Lin, W., Diaryl Dihydrophenazine-Based Porous Organic Polymers Enhance Synergistic Catalysis in Visible-Light-Driven Organic Transformations. *Angew. Chem. Int. Ed.* **2023,** 135, e202310470.

(2) Bao, K.; Wei, J.; Yan, H.; Sheng, R., Visible-light promoted three-component tandem reaction to synthesize difluoromethylated oxazolidin-2-imine. *RSC Adv.* **2020,** 10, 25947-25951.

(3) Ye, J. H.; Song, L.; Zhou, W. J.; Ju, T.; Yin, Z. B.; Yan, S. S.; Zhang, Z.; Li, J.; Yu, D. G., Selective oxytrifluoromethylation of allylamines with CO2. *Angew. Chem. Int. Ed.* **2016,** 128, 10176-10180.

(4) Frisch, M.; Trucks, G.; Schlegel, H.; Scuseria, G.; Robb, M.; Cheeseman, J.; Scalmani, G.; Barone, V.; Mennucci, B.; Petersson, G., Uranyl extraction by N, N-dialkylamide ligands studied by static and dynamic DFT simulations. In *Gaussian 09,* Gaussian Inc Wallingford: 2009.

(5) Perdew, J. P.; Burke, K.; Ernzerhof, M., Generalized gradient approximation made simple. *Phys. Rev. Lett.* **1996,** 77, 3865.

(6) Grimme, S.; Antony, J.; Ehrlich, S.; Krieg, H., A consistent and accurate ab initio parametrization of density functional dispersion correction (DFT-D) for the 94 elements H-Pu. *J. Chem. Phys.* **2010,** 132.

(7) Grimme, S.; Ehrlich, S.; Goerigk, L., Effect of the damping function in dispersion corrected density functional theory. *J. Comput. Chem.* **2011,** 32, 1456-1465.

(8) Hay, P. J.; Wadt, W. R., Ab initio effective core potentials for molecular calculations. Potentials for the transition metal atoms Sc to Hg. *J. Chem. Phys.* **1985,** 82, 270-283.

(9) Hay, P. J.; Wadt, W. R., Ab initio effective core potentials for molecular calculations. Potentials for K to Au including the outermost core orbitals. *J. Chem. Phys.* **1985,** 82, 299-310.

(10) Hariharan, P.; Pople, J. A., Accuracy of AH n equilibrium geometries by single determinant molecular orbital theory. *Mol. Phys.* **1974,** 27, 209-214.

(11) Lu, T.; Chen, F., Multiwfn: A multifunctional wavefunction analyzer. *J. Comput. Chem.* **2012,** 33, 580-592.

(12) Liu, Z.; Lu, T.; Chen, Q., An sp-hybridized all-carboatomic ring, cyclo [18] carbon: Electronic structure, electronic spectrum, and optical nonlinearity. *Carbon* **2020,** 165, 461-467.

(13) Humphrey, W.; Dalke, A.; Schulten, K., VMD: visual molecular dynamics. *J. Mol. Graph.* **1996,** 14, 33-38.

(14) Le Bahers, T.; Adamo, C.; Ciofini, I., A qualitative index of spatial extent in charge-transfer excitations. *J. Chem. Theory Comput.* **2011,** 7, 2498-2506.

(15) D'Avino, G.; Mothy, S.; Muccioli, L.; Zannoni, C.; Wang, L.; Cornil, J.; Beljonne, D.; Castet, F., Energetics of electron–hole separation at P3HT/PCBM heterojunctions. *J. Phys. Chem. C* **2013,** 117, 12981-12990.

(16) Plasser, F.; Thomitzni, B.; Bäppler, S. A.; Wenzel, J.; Rehn, D. R.; Wormit, M.; Dreuw, A., Statistical analysis of electronic excitation processes: Spatial location, compactness, charge transfer, and electron-hole correlation. *J. Comput. Chem.* **2015,** 36, 1609-1620.
